# Design, synthesis, in silico and biological evaluations of novel polysubstituted pyrroles as selective acetylcholinesterase inhibitors against Alzheimer’s disease

**DOI:** 10.1038/s41598-022-18224-6

**Published:** 2022-09-08

**Authors:** Hormoz Pourtaher, Alireza Hasaninejad, Aida Iraji

**Affiliations:** 1 Department of Chemistry, Faculty of Sciences, Persian Gulf University, Bushehr, 75169 Iran; 2 Stem Cells Technology Research Center, Shiraz University of Medical Sciences, Shiraz, Iran; 3 Central Research Laboratory, Shiraz University of Medical Sciences, Shiraz, Iran

**Keywords:** Biochemistry, Drug discovery, Medical research, Chemistry, Mathematics and computing

## Abstract

The objective of this study was to design new polysubstituted pyrrole derivatives as selective acetylcholinesterase (AChE) inhibitors to target Alzheimer's disease. In this context, a highly efficient, one-pot, sequential, multi-component synthesis of a diverse range of polysubstituted pyrroles was developed through a sequential domino strategy by the condensation of amines with 1,1-bis(methylthio)-2-nitroethene (BMTNE), Knovenagle reaction of arylglyoxals with malono derivatives and subsequent Michael addition and intramolecular cyclization reaction in EtOH at reflux*.* Thirty-nine synthesized compounds were evaluated as AChE and butyrylcholinesterase (BChE) inhibitors. Among the synthesized compounds, compound **4ad** (IC_50_ = 2.95 ± 1.31 µM) was the most potent and selective AChE inhibitor with no significant inhibition against butyrylcholinesterase BChE. A kinetic study of **4ad** revealed that this compound inhibited AChE in an uncompetitive mode. Based on a molecular modeling study, compound **4ad** due to its small size properly fitted into the active site of AChE compared to BChE and stabilized by H-bond and hydrophobic interactions with the critical residues of the AChE binding pocket. Consequently, it was proposed that the **4ad** derivative can be an ideal lead candidate against AD with a simple and practical operation of synthetic procedures.

## Introduction

Alzheimer's disease (AD) is the most common form of dementia and progressive neurodegenerative disorder with memory loss, cognitive decline, and language dysfunction in people above the age of 60^1^. AD is characterized by the accumulation of amyloid-β (Aβ) aggregates in the synaptic cleft and intraneuronal aggregations mainly constituted by hyperphosphorylated Tau protein (p-Tau). It is believed that such toxic peptide species cause neuroinflammation, and induce oxidative stress, neuronal dysfunction and, ultimately leading to massive degeneration and neuronal loss in the brain^2,3^.

Historically, the molecular hallmarks of AD have been identified as a result of a reduction in cholinergic neurotransmission named acetylcholine (ACh) due to neuronal distraction and apoptosis^4^. ACh neurotransmitter was hydrolyzed with the cholinesterases including acetylcholinesterase (AChE) and butyrylcholinesterase (BChE)^5^. AChE is presented in the central nervous system (CNS) and plays the most dominant role in the hydrolysis of ACh at a different stage of AD^6^.

Nowadays the most effective treatment for AD is to enhance cholinergic neurotransmission and reduce hydrolysis of ACh in the brain by using AChE inhibitors^7,8^. Donepezil, galantamine, and rivastigmine, as well as huperzine A are approved AChE inhibitors that increase acetylcholine levels at synapses and improve neurotransmission. In this context, search for novel AChE inhibitors ongoing and novel scaffolds including dioxane-4,6-dione^9^, thiourea^10,11^, arylisoxazole^12^, thio methyltriazole were developed^10,13–16^.

Multicomponent reactions (MCRs) have emerged as a powerful tool for the purpose that a series of chemical processes can be controlled in a one-pot operation^17,18^. These reactions involve forming multiple bonds in a single conversion in a one-pot process without separating intermediates^19–22^.

Nitro ketene dithioacetals are known as dicarbon synthons in organic synthesis due to their push–pull electronic nature. Reactions of nitro ketane dithioacetals with a variety of nucleophilic groups have so far been applied to construct various heterocycles^23,24^ with diverse biological activities, including antitumor^25,26^, antianxiety^27^, antileishmanial^28^ and antibacterial^29^.

**Figure 1:**
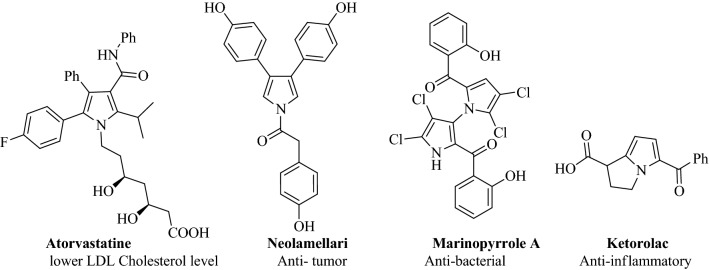
Selected drugs containing pyrrole core.

Compounds containing N-heterocyclic skeletons are known to exhibit various biological and medicinal activities^30–34^. Among these compounds, pyrrole derivatives are generally shown to have several biological activities as antioxidant^35^, antifungal^36^, antituberculosis^37^, anti-inflammatory^38^, analgesic ^39^, antidiabetic ^40^ and anticancer^41^ agents. Some examples of pharmaceutical compounds with pyrrole ring skeletons are shown in Fig. 1^42,43^. In addition, pyrrole is an important core moiety in many biological compounds such as chlorophyll, heme, vitamin B_12_, bile pigments, and alkaloids.

A number of synthetic strategies for the construction of substituted pyrrole derivatives have been reported such as the Knorr reaction^44^, the Paal-Knorr reaction^45^, the Hantzsch synthesis^46^ and different cycloaddition methods^47^. Some of these methods have disadvantages such as long reaction time, low yields, expensive reagents or catalysts, harsh reaction conditions, the use of toxic solvents, and methods that do not comply with the principles of green chemistry.

Owing to the attractive medicinal properties of pyrrole derivatives, the development of a new and simple synthetic strategy for the efficient synthesis of novel polysubstituted pyrroles will be a useful and attractive challenge. In continuation of our laboratory efforts on the development of multicomponent reactions for the synthesis of biologically important heterocyclic compounds^48–51^ herein, an efficient and simple method was developed to synthesize a library of polysubstituted pyrroles as AChE and BChE inhibitors. Furthermore, the kinetic studies of the most potent derivative were performed. The most potent compound was then subjected to molecular docking and molecular dynamic (MD) studies to evaluate its binding affinity and mode of action within the binding site of the enzyme. Finally, drug-likeness predictions were performed to address the pharmacokinetic properties of the most interesting compounds.

## Result and discussion

### Design of new scaffold as AChE inhibitor

According to crystallographic studies, the binding pocket of AChE comprises a narrow gorge approximately 20 Å deep, consisting of two binding sites: catalytic activity site (CAS) at the bottom of the gorge and peripheral anion site (PAS) near the entrance of the gorge. According to several studies, dual binding site inhibitors of AChE occupy CAS and PAS pockets and exert their effects more efficiently. Generally, dual binding site AChE inhibitors may not only improve cognition by inhibiting AChE but also slow down the aggregation of Aβ to generate toxic plaques^52^.

**Figure 2:**
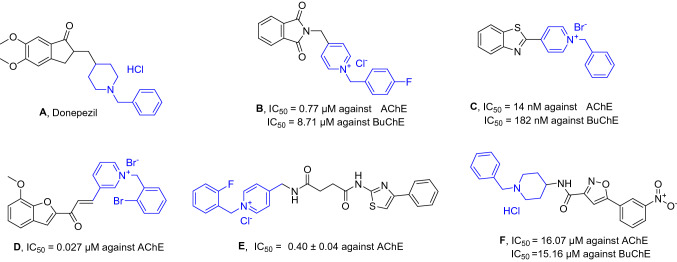
Traditional AChE inhibitors including donepezil hydrochloride as an FDA-approved AChE inhibitor and related structures.

The traditional AChE inhibitors comprise three parts including a core ring that interacts with amino acid residues of PAS, a basic moiety to interact with the aromatic residues of CAS, and a linker to link these two parts, which lies in a narrow part of the active site of AChE. Compounds **A-F** (Fig. 2) are some AChE examples^53–59^. However, those inhibitors that possess a positive charge (quaternary amines) are mostly unable to cross the blood–brain barrier (BBB) and show poor efficiency in CNS.

**Figure 3:**
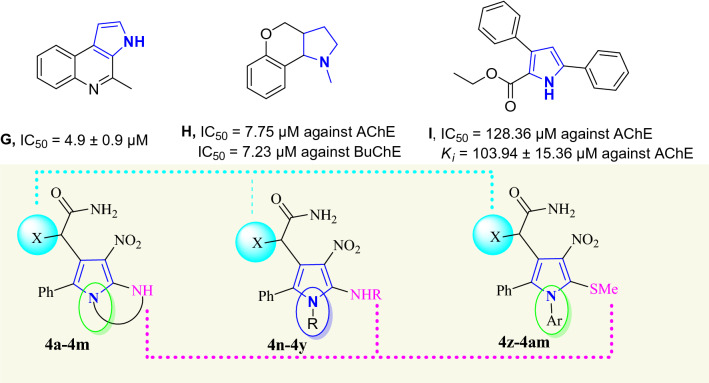
Structures of previously studied pyrrole derivatives as ChE inhibitors and the newly designed compounds (**4a-m**, **4n-y,** and **4z-am)**.

There are limited data available about the ChE inhibitory activities of polysubstituted pyrroles. Amongst compounds **G**^60^, **H**^61^, and **I**^62^ with moderate to good activity against AChE were presented in Fig. 3. Recently, compound bearing pyrrole-sulfonyl amine was reported as an example of pyrrole derivatives with IC_50_ values ranging from 6.50 to 37.46 nm against AChE^63^.

It was assumed that the pyrrole ring can play a role as core moiety to interact with the aromatic residues of the AChE binding pocket while different substitutions on the ring might improve its potencies. In this context, a library of 39 molecules was designed and synthesized to properly evaluate the structure–activity relationships (SARs) of pyrrole derivatives against AChE and BChE.

### Chemistry

**Figure 4:**
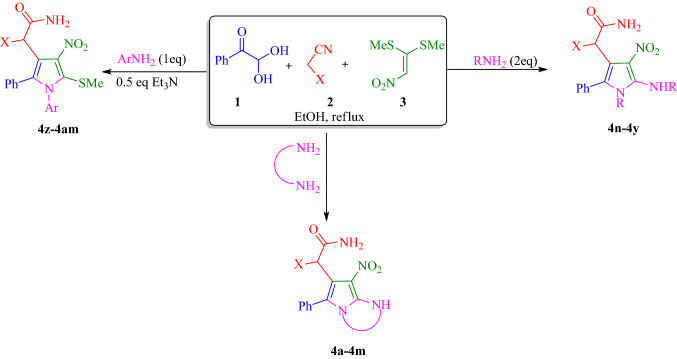
Synthesis of polysubstituted pyrroles **4a-4am**.

The synthetic route of the polysubstituted pyrroles is shown in Fig. 4. Initially, BMTNE **3** (1 mmol) and amine (2 eq) or diamine (1 eq) were reacted in EtOH at reflux for 6 h to form the corresponding intermediate **A**. Then arylglyoxal **1** (1 mmol) and malono derivative **2** (1 mmol) were added to obtain the desired product **4**. It should be mentioned that some other solvents such as H_2_O, CH_3_CN, CHCl_3_, THF, and DMF were tested and the results showed that EtOH was the best solvent. Having optimized the reaction conditions a series of polysubstituted pyrroles were successfully synthesized under the given reaction conditions and the results are summarized in Table 1.

**Table 1:**
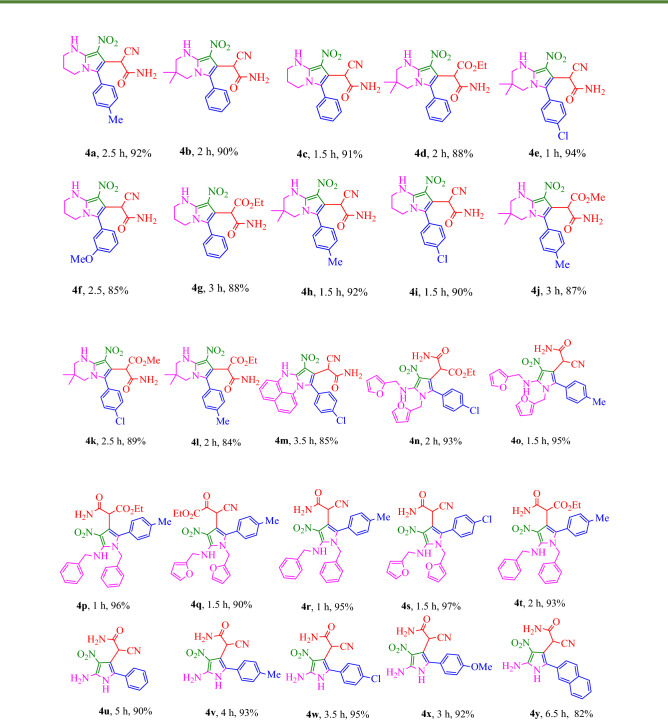

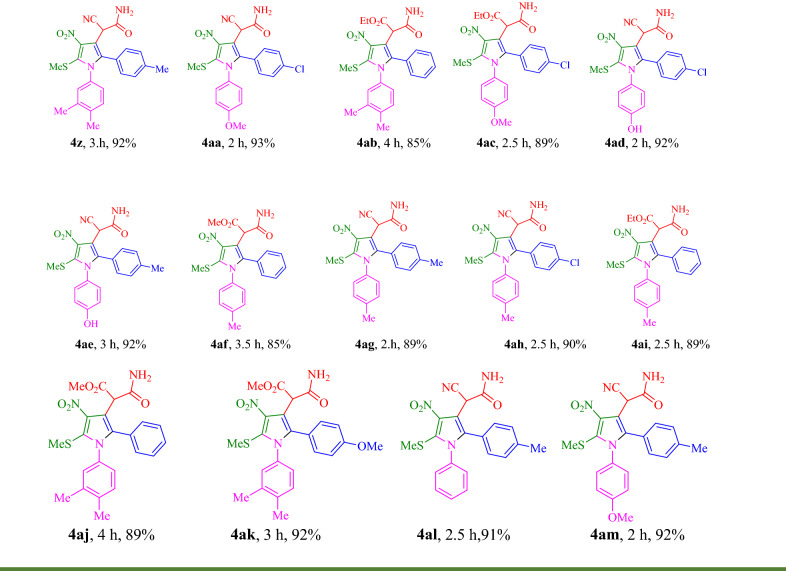
Multicomponent synthesis of polysubstituted pyrrole﻿ derivatives^a,b^.

^a^Isolated yield. ^b^Reaction conditions: BMTNE **3** (1 mmol) and amine (1 or 2 mmol) were added to EtOH (5 mL) at reflux and after 6 h arylglyoxal **1** (1 mmol) and malono derivative **2** (1 mmol), were added to obtain the desired product **4.**

As shown in Table 1, a diverse range of different arylglyoxals, amines and malono derivatives were successfully employed. In this study, methoxy, methyl and chloro substituents on the phenylglyoxal ring showed similar productivity in comparison with phenylglyoxal. In addition, different malono derivatives such as malononitrile and alkyl cyanoacetates were used in these reactions. However, the use of malono nitrile leads to a shorter reaction time and higher yields compared to ethyl or methyl cyanoacetate. Diamines such as 1,3-propyl diamine, 2,2-dimethyl-1,3-propane-diamine and 1,8-diaminonaphthalene were used for the effectient synthesis of fully substituted pyrroles in good to excellent yields (compounds 4a-4 m). Also, two equivalents of alkylamines such as benzylamine and furfuralamine were used and the corresponding products were synthesized in excellent yield (compounds 4n-4t). In this study, aqueous ammonia solution (25%) was used as a source of amine to obtain N-substituted-free pyrroles and these type of pyrroles were prepared efficiently (compounds 4u-4y). It should be noted that in this case, ammonia was used in excess amount (1.5 mL for 1 mmol of BMTNE). Aromatic amines were also used and studies shown that only one equivalent of amine is presented in the final product even when we used two equivalents of the amine. On the other hand, this method has successfully led to the formation of polysubstituted pyrroles haveing thiomethyl substituent in their structure, which can be potentially biologically important compounds (4z-4am). When we used 1,2- Phenylenediamine derivatives as the source of amine, the reaction proceeded in another direction that is currently being investigated in our laboratory.

**Figure 5:**
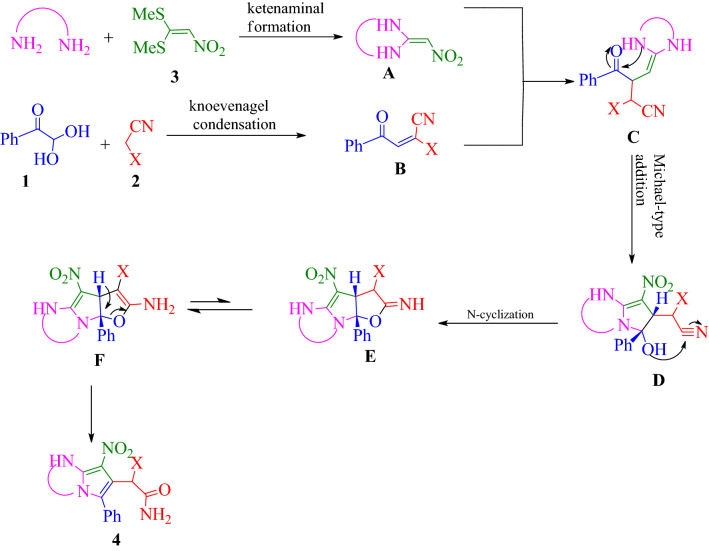
Proposed mechanism for the synthesis of polysubstituted pyrrole derivatives.

The most probable reaction mechanism for the formation of compounds **4** depicted in Fig. 5). The initial reaction between diamine and BMTNE** 3** lead to intermediate **A**. In the next step, the Knoevenagel condensation of arylglyoxal **1** and malono derivative **2** lead to intermediate **B**. Also, Michael addition of intermediate **A** to intermediate **B** will provide intermediate **C**, which next undergoes cyclization to give **D** and then **E**. The bicyclic intermediate **E** further undergoes ring opening through intermediate **F** and finally leading to the formation of **4**.

### In vitro ChE inhibitory activities

First derivatives **4a-l** were synthesized to evaluate their potency as AChE and BChE inhibitors. Results are summarized in Table 2 in terms of % inhibition at 50 µM and IC_50_s. According to IC_50_ values of pyrrolo[1,2-a]pyrimidine analogs, this set of compounds recorded, mostly, better inhibitory activity toward AChE compared to BChE. The exception in this trend came back to **4a** and **4j.**

**Table 2:** IC_50_ values and percent inhibitory activity of polysubstituted pyrroles against AChE and BChE enzymes.


Compound	R^1^	R^2^	R^3^	AChE^a^		BChE^a^	
				% inhibition at 50 µM	IC_50_ µM	% inhibition at 50 µM	IC_50_ µM
**4a**	H	CN	4-CH_3_	14.71 ± 2.26	–	26.07 ± 4.09	–
**4b**	Methyl	CN	H	52.01 ± 4.43	37.15 ± 3.52	NA	–
**4c**	H	CN	H	39.75 ± 3.69	–	NA	–
**4d**	Methyl	COOEt	H	13.50 ± 1.58	–	13.50 ± 2.96	–
**4e**	Methyl	CN	4-Cl	57.05 ± 5.46	36.30 ± 4.85	18.14 ± 2.89	–
**4f**	H	CN	3-OCH_3_	33.49 ± 5.19	–	17.78 ± 2.79	–
**4g**	H	COOEt	H	30.78 ± 5.17	–	NA	–
**4h**	Methyl	CN	4-CH_3_	45.81 ± 2.81	67.60 ± 7.54	NA	–
**4i**	H	CN	4-Cl	33.94 ± 6.87	–	NA	–
**4j**	Methyl	COOMe	4-CH_3_	14.92 ± 2.45	–	28.18 ± 3.83	–
**4k**	Methyl	COOMe	4-Cl	13.35 ± 4.88	–	13.66 ± 2.13	–
**4l**	Methyl	COOEt	4-CH_3_	14.18 ± 2.12	–	NA	–
**Donepezil**					0.079 ± 0.05		10.6 ± 2.1

^a^Data represented in terms of mean ± SD.

Assessment of SARs indicated interesting results so that type of substitution at R^1^ and R^2^ positions played the most dominant role in AChE inhibitory activity compared to R^3^ substitution. As can be seen compounds **4b** (IC_50_ = 37.15 µM), **4e** (IC_50_ = 36.30 µM), and **4h** (IC_50_ = 67.60 µM) bearing 3,3-dimethyl at the R^1^ with CN moiety at R^2^ position significantly improved the inhibitory potencies and resulted in the most potent derivatives in this set compared to the other derivatives.

Exploration the inhibitory activity based on R^2^ substitation exhibited that derivitives bearing CN moiety at R^2^ showed better activity against AChE compared to their counterparts bearing COOEt and COOMe. This is the case in **4b** (% inhibition at 50 µM = 52.01) versus **4d** (% inhibition at 50 µM = 13.50), **4e** (% inhibition at 50 µM = 57.05) versus **4k** (% inhibition at 50 µM = 13.35) and **4h** (% inhibition at 50 µM = 45.81) versus **4l** (% inhibition at 50 µM = 14.18).

The substituted groups at the R^3^ position did not disclose straightforward inhibition and significant differences. It seems that this position had the least effect on the AChE inhibitory activity.

Next to better extract the SARs of pyrrole-acetamide derivatives **4n-ak** were also synthesized. The activity of **4n-t** bearing aromatic rings at R^1^ and R^4^ positions showed different results so these analogs were more potent against BChE compared to AChE (Table 3). It seems that the increase bulkiness in this position makes the derivatives more potent as BChE inhibitors. The exception in this trend came back to **4p**. The activity of BChE changes in the following order so that **4q** (R^1^: -aminomethylfuran; R^2^: COOEt; R^3^: 4-CH_3_ and R^4^: -methylfuran) showed IC_50_ values of 18.62 µM followed by **4o** (R^1^: -aminomethylfuran; R^2^: CN; R^3^: 4-CH_3_; R^4^: -methyl-furan; IC_50_ = 69.18 µM).

**Table 3:**
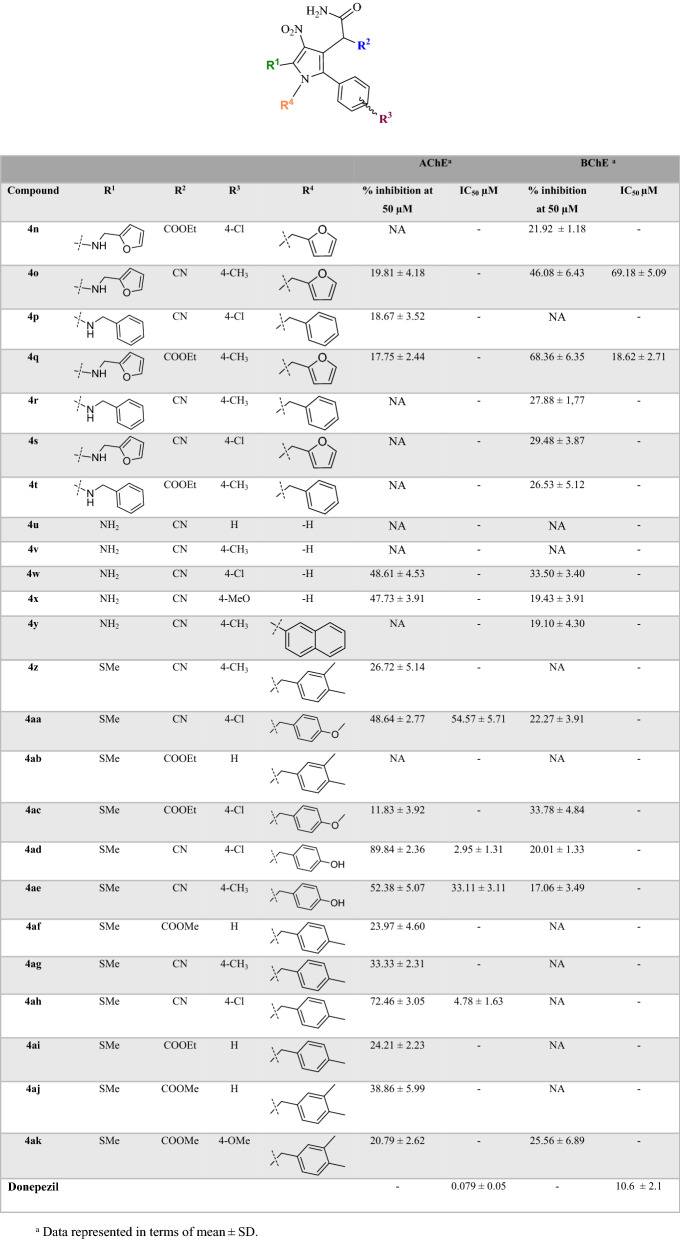
IC_50_ values and percent inhibitory activity of polysubstituted pyrroles against AChE and BChE enzymes^a^.

Changing the type of R^3^ position while R^1^ and R^4^ are the same exhibited that the presence of *para*-methyl as an electron-donating group is more favorable compared to *para* chlorine as a halogen-substituted group as BChE inhibitor. In this case, **4o** and **4q** recorded better activities compared to **4n** and **4s** as well as **4r** and **4t** versus **4p**.

Based on the obtained biological results related to **4u-y** (R^1^ = NH_2,_ R^2^ = CN), three derivatives were almost inactive against both enzyme. **4w** (R^3^ = 4-Cl) as the most active compound in this set showed an inhibition percent of 48.61 against AChE and 33.50 against BChE at 50 M followed by **4X** (R^3^ = 4-MeO) with 47.73% and 19.43% inhibition percent against AChE and BChE, respectively.

In the last set (**4z-4ak**), NH_2_ of R^1^ position was replaced with SMe. The most potent compounds with high selectivity against AChE were presented in this set. The most active compounds among all tested derivatives were **4ad** (R^2^ = CN; R^3^ = 4-Cl; R^4^: 4-hydroxyphenyl) with an IC_50_ value of 2.95 ± 1.31 µM followed by **4ah** (R^2^ = CN; R^3^ = 4-Cl; R^4^ = 4-methylphenyl) with IC_50_ value of 4.78 ± 1.63 µM. In this group COOEt or COOMe substitutions at R^2^ inferior the activity (% inhibition at 50 M ranging from not active to 38.86) compared to the CN (% inhibition at 50 M ranging from 26.72 to 89.84) substituted counterparts.

**Figure 6:**
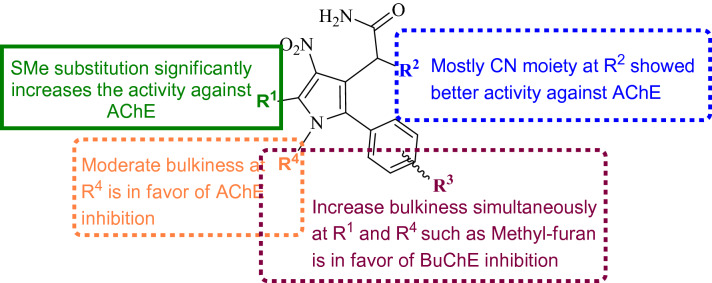
Summary of SARs of polysubstituted pyrroles as ChEs inhibitors.

The summary of SAR was presented in Fig. 6. The highest potency to inhibit AChE was observed in compounds bearing SMe group at R^1^, CN moiety at R^2^ and 4-Cl at R^3^ position. Compounds **4ad** and **4ah** with IC_50_ values of 2.95 ± 1.31 µM and 4.78 ± 1.6 were the most potent AChE inhibitors, respectively.

Regarding the BChE, the presence of -methylfuran ring at R^1^ and R^4^ (**4q**, IC_50_ = 18.62 ± 2.71 µM and **4O**, IC_50_ = 69.18 ± 5.09 µM) make the derivatives more ideal as BChE inhibitors.

### Kinetic studies of AChE inhibition

**Figure 7:**
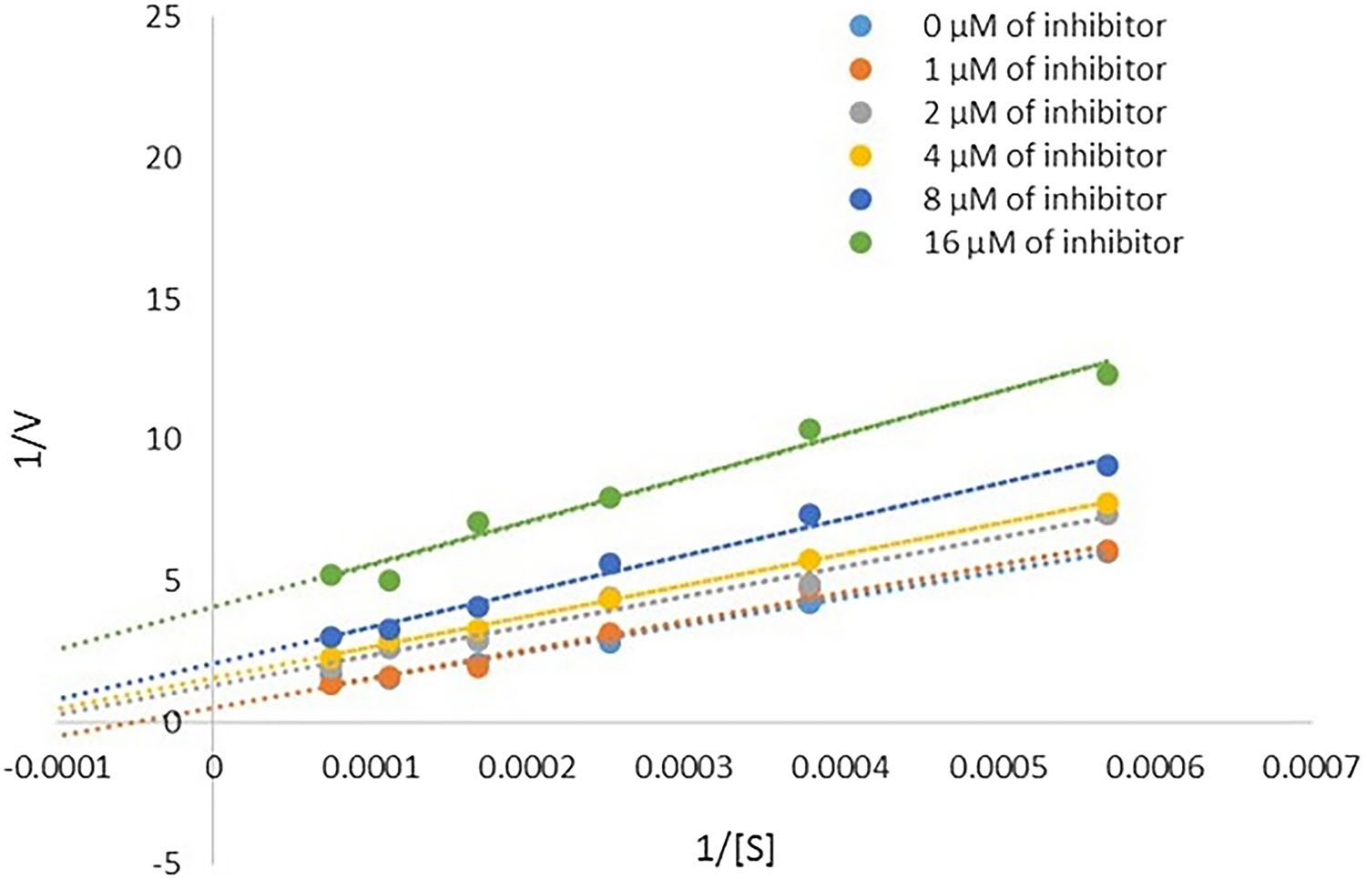
Kinetic study on the mechanism of AChE inhibition by **4ad**. Overlaid Lineweaver − Burk reciprocal plots of AChE initial velocity at increasing substrate concentration (0.1–1 mM) in the absence or presence of **4ad** are shown.

To determine the mechanism of inhibition of AChE, a kinetic study of **4ad** was done against AChE. The reciprocal Lineweaver–Burk plots (Fig. 7) illustrate that *K*_*m*_ and *V*_*max*_ reduced with the increasing concentration of **4ad** which indicates that **4ad** is an uncompetitive type inhibitor.

**Figure 8:**
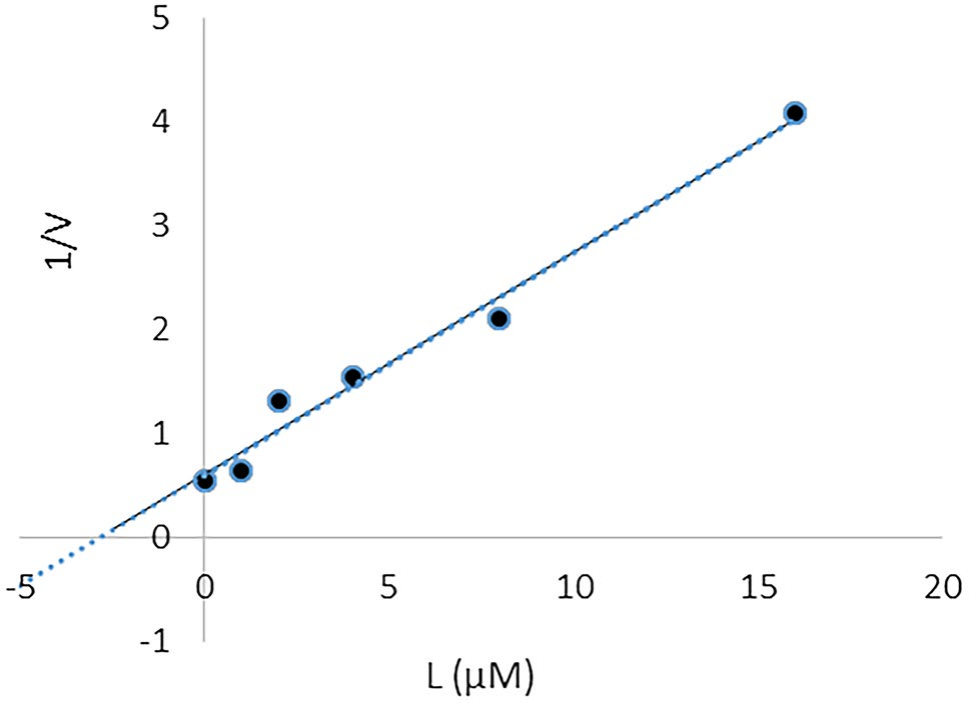
Double reciprocal Lineweaver–Burk plot of **4ad** against AChE.

Furthermore, the plot of the *K*_*m*_ versus different concentrations of **4ad** gave an estimate of the inhibition constant, *K*_*i*_ of 2.84 µM which is in accordance with the IC_50_ value of 2.95 ± 1.31 µM (Fig. 8).

### Molecular modeling studies of 4ad in AChE and BChE active site

Given the uncompetitive-type inhibitory nature of **4ad**, its interaction with the active site of AChE was subsequently investigated. First, the docking procedures were validated by accurately redocking the co-crystallized ligands into the AChE and BChE models. CAS located at the bottom of the gorge consist of two sub-units namely, the catalytic triad (esteratic sub-site) and the anionic subsite and the second is PAS. Glutamic acid (E202), serine (S203) and histidine (H447) are the main residues of the catalytic triad while the anionic subsite consists of tryptophan (W86). PAS consists of amino acids of tryptophan (W286), tyrosine (Y337), and phenylalanine (F338) which guide molecules to the CAS^10,64,65^.

**Figure 9:**
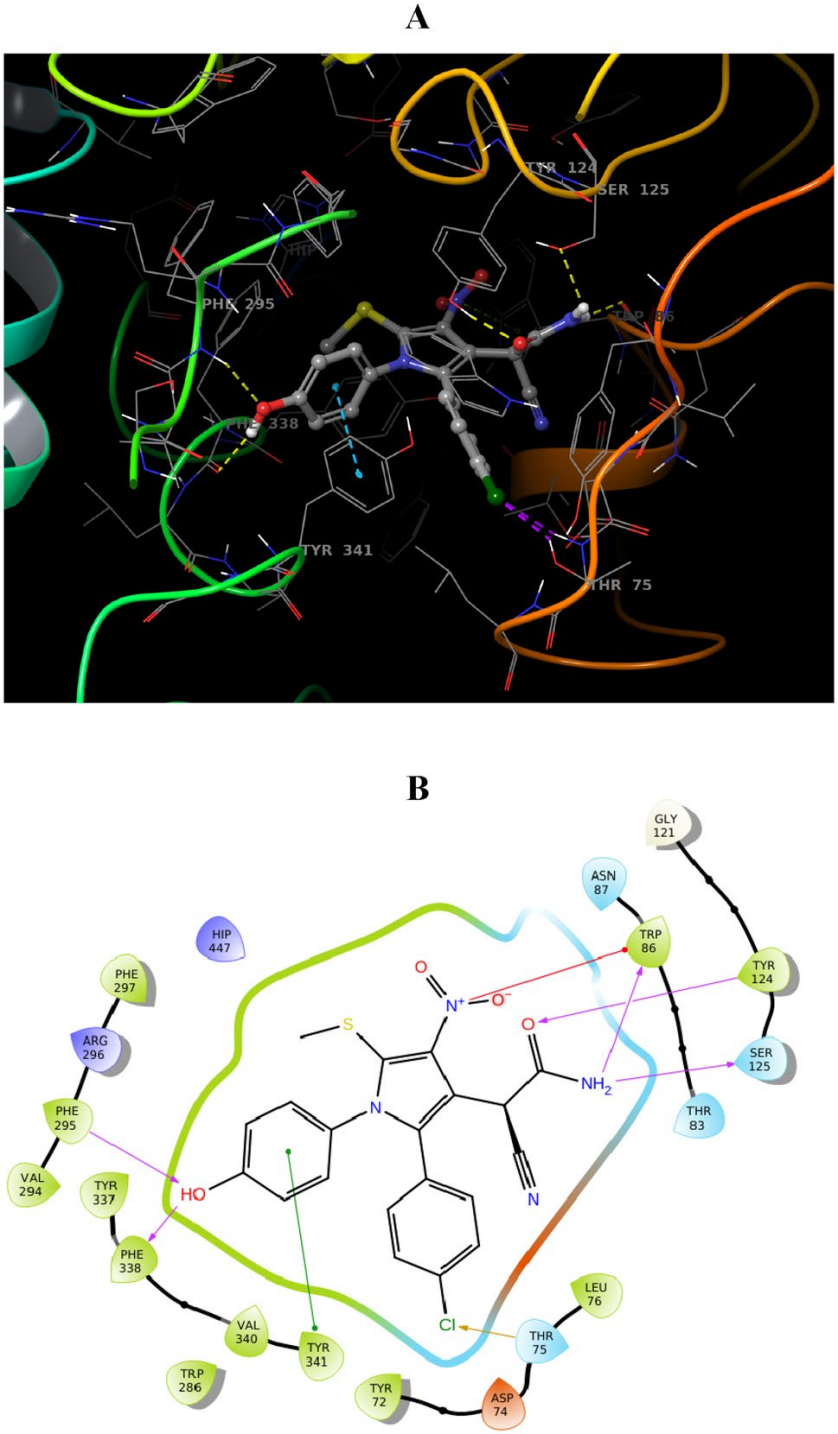
(**A**) 3D Binding pattern of **4ad** within the active site of AChE, (**B**) 2D binding interactions **4ad** in AChE active site.

Computational docking studies were performed to model the interaction of **4ad** with AChE (PDB ID: 4BDS) at the target-binding site (Fig. 9). The nitro and acetamide moieties of **4ad** are accommodated in CAS. In detail, the acetamide group showed three hydrogen bound interactions with Trp86 (anionic subsite) and Tyr124, and also the nito group recorded interaction with Trp 86. On the other side of the molecule, 4OH-benzyl is oriented towards PAS and displayed two hydrogens bound interactions with Phe295 and Tyr341 of PAS. This ring also exhibited interaction with Tyr341. *Para* chlorophenyl also demonstrated hydrophobic interaction with Thr75. These interactions were in line with the results of biological assessments and confirmed the high potency of this derivative.

**Figure 10:**
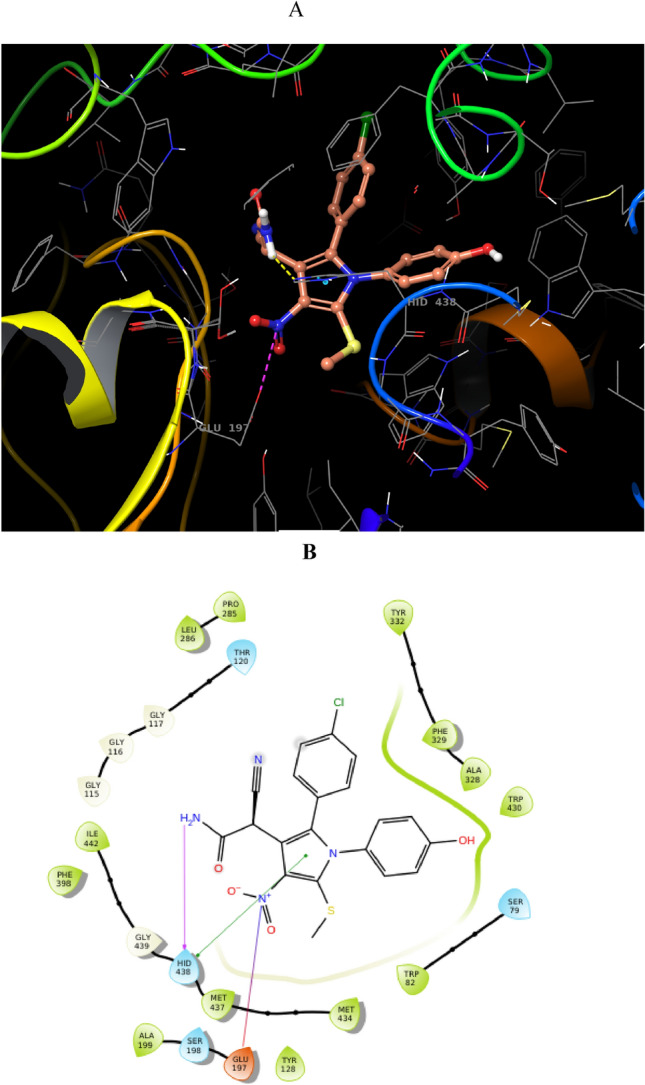
(**A**) 3D Binding pattern of **4ad** within the active site of BChE, (**B**) 2D binding interactions **4ad** against BChE.

Also, the molecular docking study of **4ad** as one of the inactive compounds was performed against BChE. **4ad** did not tightly interact with BChE active site and just showed interactions with His438 and His438. A salt bridge interaction of the nitro group with Glu197 was observed (Fig. 10).

These results justify the high potencies of this derivative against AChE compared to BChE. It should be noted that as the active site of AChE is smaller than BChE, the smaller molecules, in this case, **4ad**, properly fitted in the binding site of the enzyme and exhibited interaction with PAS and CAS pocket. However **4ad** derivative could not effectively occupy all pockets of BChE where this compound lacks hydrogen bonds or pi–pi interactions with active site residue.

### Molecular dynamics simulation

**Figure 11:**
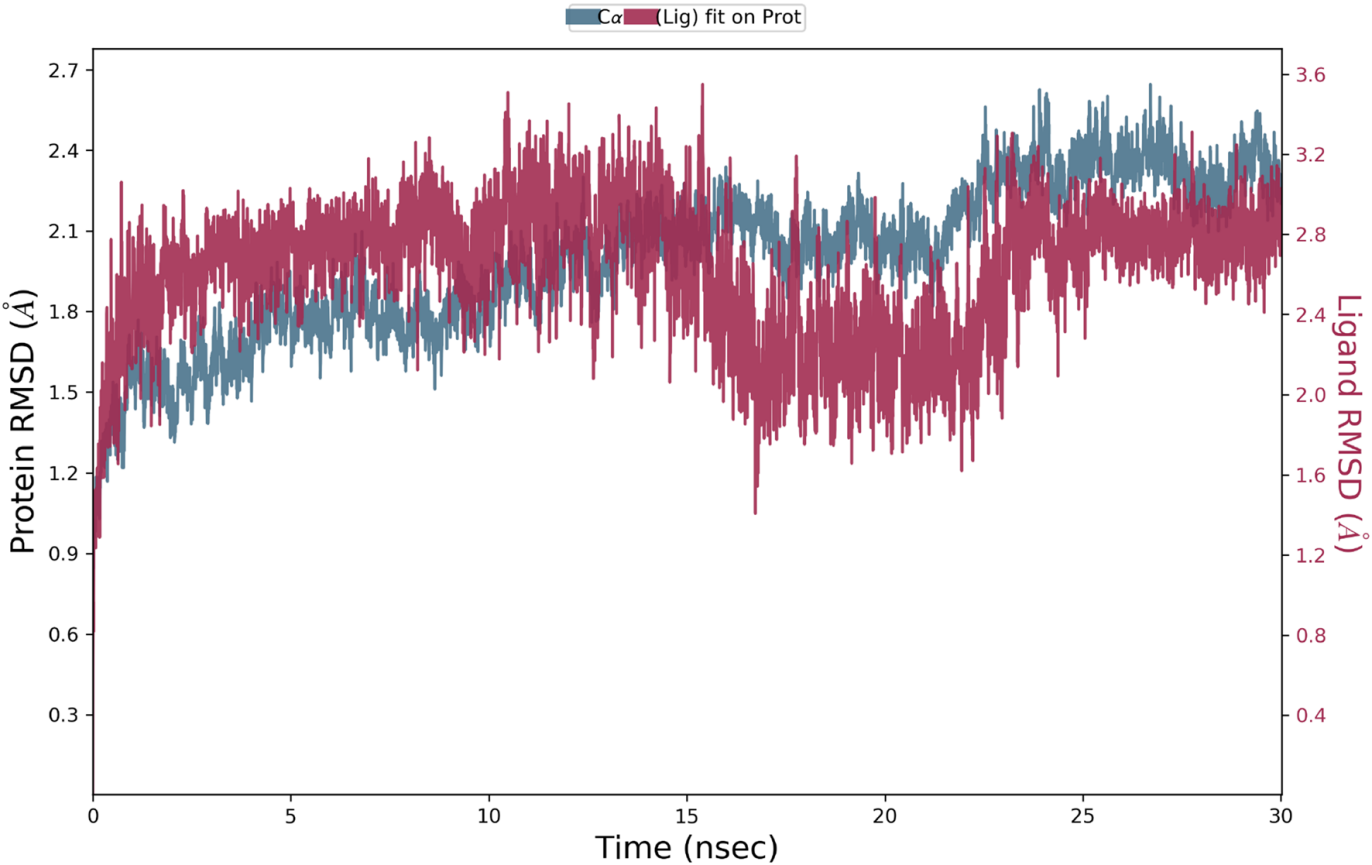
RMSD plot of the AChE in complex with compound **4ad** in the MD simulation time. RMSD values of the Ca atoms of the protein are depicted in blue, and ligands fitted in protein are exhibited in red.

MD simulation was performed to model the time-dependent motions and stabilization of the **4ad**-AChE complex. The root mean square deviation (RMSD) showed the average change in displacement of a selection of atoms for a particular frame. The RMSD value of less than 3 Å is perfectly acceptable for small, globular proteins. As can be seen, the RMSD stabilizes around 2.1 Å indicating the simulation is in equilibration. Also, it can be seen that after 15 s the RMSD of the ligand was slightly fewer than the RMSD protein which confirms that the ligand is fixed in the binding site (Fig. 11).

**Figure 12:**
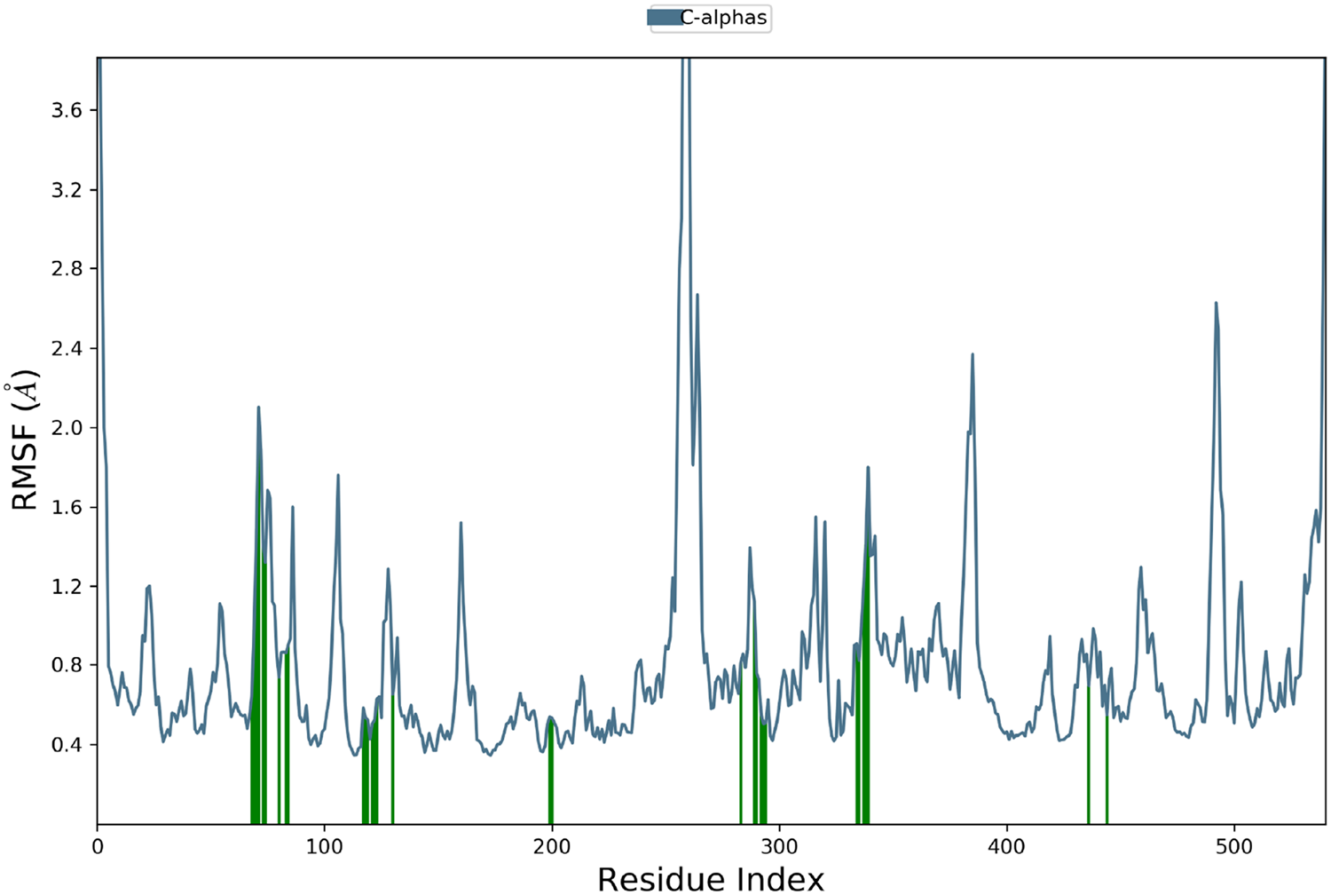
RMSF plot of the AChE residues in complexed with **4ad**.

The root mean square fluctuation (RMSF) is useful for characterizing local changes along the protein chain. As can be seen, residues between 270 and 285 (PAS pocket) showed the most fluctuation which showed the highest movement. Also, the residues that more effectively interact with the ligand are marked with green-colored vertical bars (Fig. 12).

**Figure 13:**
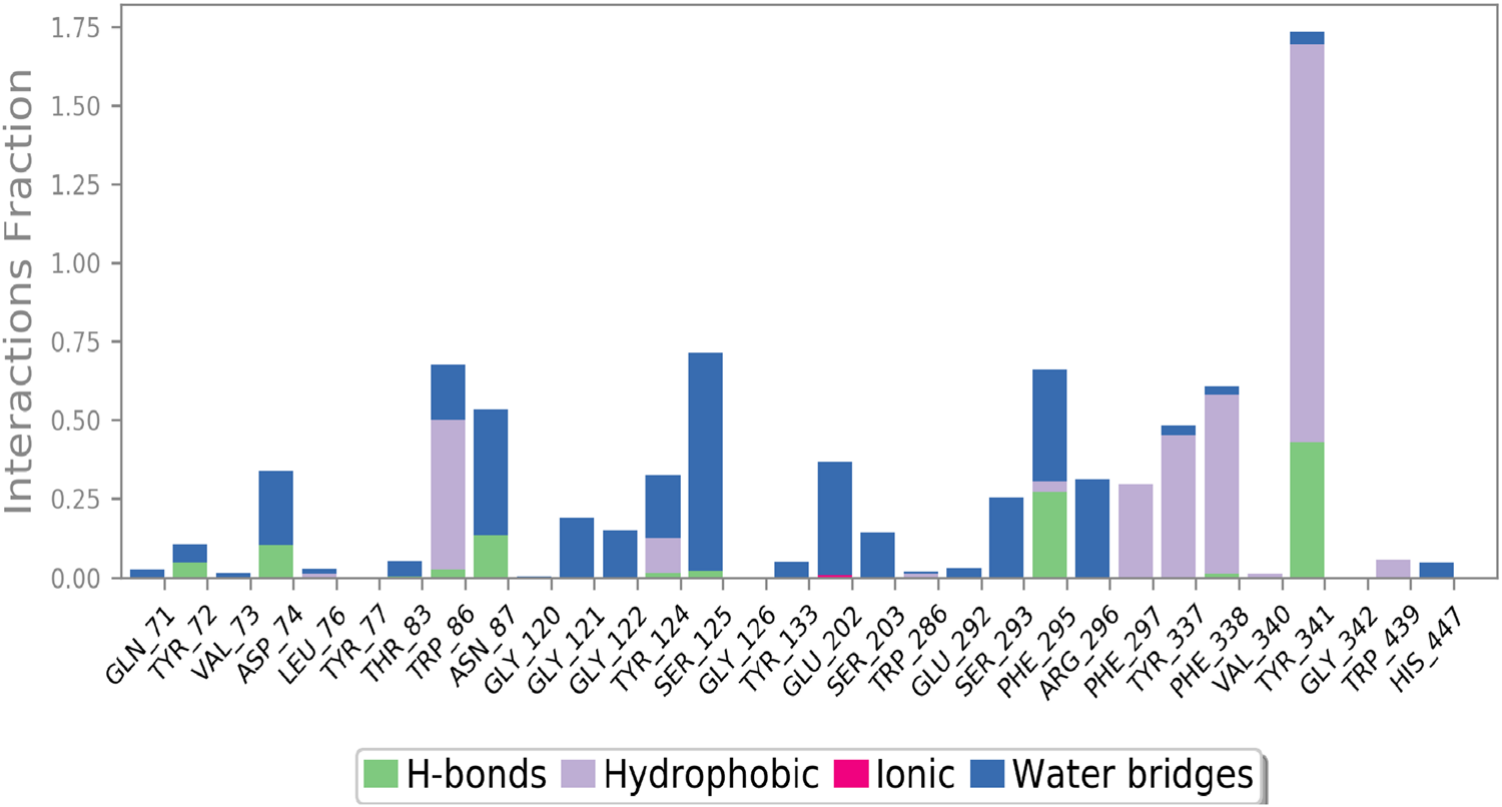
Protein–ligand contacts during the MD run.

**Figure 14:**
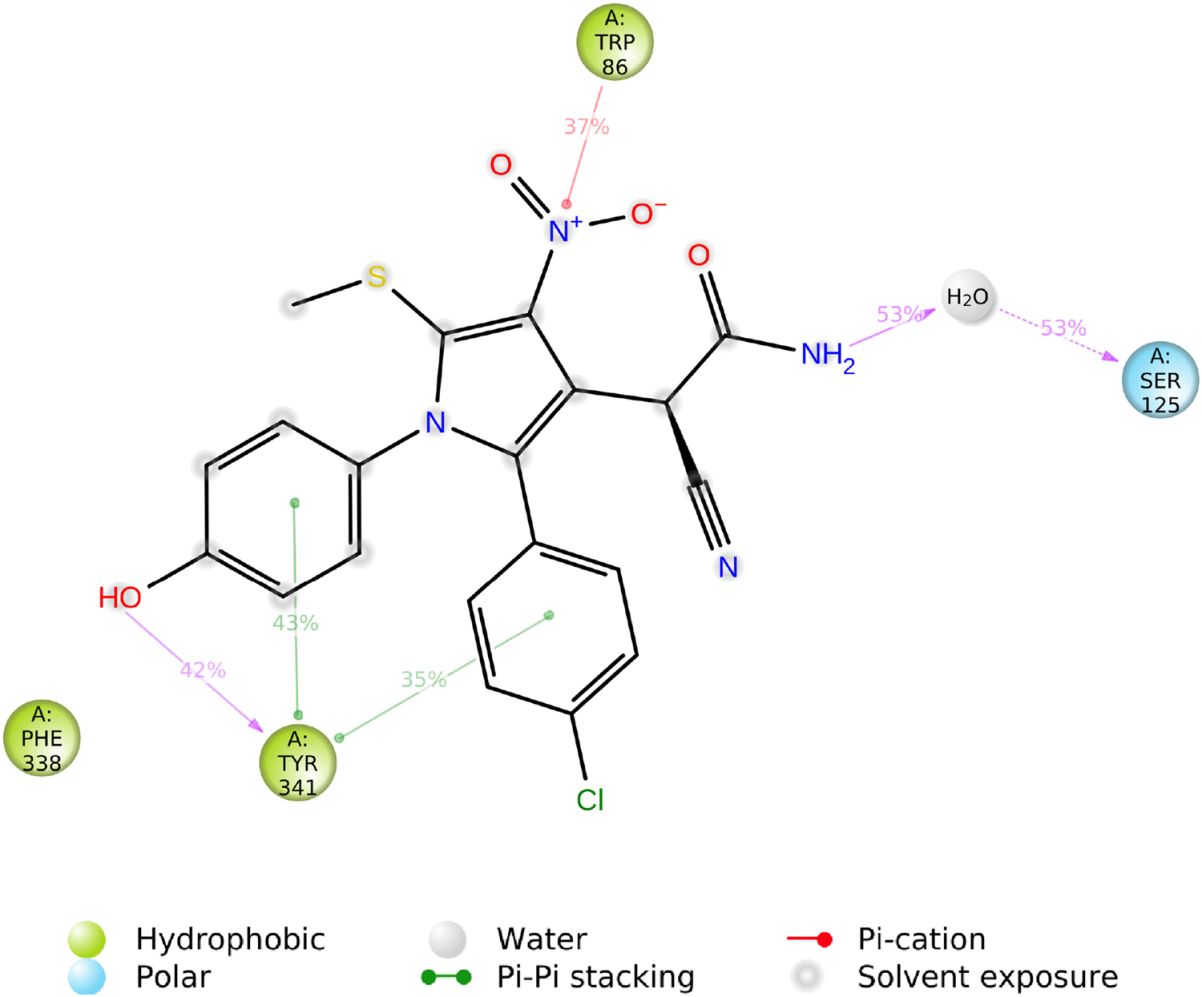
A schematic of detailed ligand atom interactions with the protein residues.

The protein interactions with the ligand can be monitored throughout the simulation. The interactions were categorized by type are summarized in Figs. 13 and 14. As shown, **4ad** exhibited interaction with Tyr341 of PAS (100% of all time) and seems to be close to the entrance of the active site gorge. Also, interaction with the Trp86 of the anionic site was recorded (75% of all time).

The molecular interactions of AChE with **4ad** over the binding site of AChE at the equilibrated phase of MD simulation were represented in Fig. 14. As can be seen, **4ad** formed H-bound interaction with Ser125, and Tyr341, through both of its NH_2_ and OH groups during the equilibrated phase. Pi-cation interaction between Trp86 and NO_2_ plus two pi–pi stacked interactions between phenyl ring and Tyr341 were also recorded.

### Physicochemical properties

In order to assess the drug-like characteristics of the most active compounds (**4ad** and **4ah**), various physicochemical parameters of these compounds were evaluated using the pkCSM and swissADME server.

Both the compounds showed good drug-like properties and follow Lipinski's rule of five (see Table 4). The logP value of both the compounds is less than five and hydrogen bond donor and acceptor atoms are in the optimum range. Both the compounds pass the drug-likeness criteria of ***Pfizer*** and ***Amgen***. Thus, it can be concluded that **4ab** and **4ah** compounds have an optimum chemical skeleton that can be developed as potential drug molecules.

**Table 4:** In silico prediction of pharmacokinetic properties of the synthesized compound.

Compounds		**4ad**	**4ah**
Physicochemical properties	MW (g/mol)	442.88	440.90
	Num. rotatable bonds	6	6
	Num. H-bond acceptors	5	4
	Num. H-bond donors	2	1
	Log P o/w (iLOGP)	1.59	2.21
	Molar Refractivity	116.02	118.96
	TPSA (Å^2^)	163.16 Å^2^	142.93
Drug-likeness rules	Intestinal absorption (human)	92.378	95.088
	Oral Rat Acute Toxicity (LD_50_)	3.245	2.777
	Lipinski (*Pfizer*)	Yes	Yes
	Ghose (*Amgen*)	Yes	Yes
	Abbott Bioavailability score	0.55	0.55

## Conclusion

In summary, a novel series of polysubstituted pyrroles derivatives **4a–4ak** were synthesized via simple multi-component chemical reactions, furnishing excellent yields and producing diverse libraries of polysubstituted pyrroles. Four sets of compounds (39 derivatives) were synthesized and the inhibitory activity of all derivatives was evaluated against AChE and BChE. SARs studies showed that SMe group at R^1^, and CN moiety at R^2^ significantly improve the AChE inhibition compared to BChE. Among them, compound **4ad** exhibited an IC_50_ value of 2.95 ± 1.31 μM against AChE.

In addition, compound **4ad** possessed an uncompetitive type of inhibition in the enzymatic assay. Furthermore, the IFD docking study of the **4ad** in the AChE active site showed key interacting residues with the PAS and CAS pocket of the enzyme. Also, MD simulations revealed compound **4ad** interacted with Tyr341 of PAS as well as Trp86 which are of the key residues anionic site. In summary, **4ad** was presented as a suitable anti-AChE agent with simple multi-components synthetic procedures that are ideal for lead optimization studies.

## Materials and method

All chemicals were purchased from Merck or Fuluka chemical companies. ^1^H-NMR (500 and 300 MHz) and ^13^C-NMR (125 and 75 MHz) spectra were run on a Varian—Inova 500 and Bruker Avance 300 MHz instrument in DMSO-*d*_*6*_. IR spectra were recorded using an FTIR apparatus. Melting points were recorded as a Buchi B-545 apparatus in open capillary tubes. Mass spectra were recorded with an Agilent-5973 C insert XL MSD mass spectrometer (Ringoes, NJ) operating at an ionization potential of 70 eV. Reaction progress was screened by TLC using silica gel polygram SIL G/UV254 plates.

### General chemistry methods

#### Typical procedure for the synthesis of polysubstituted pyrrole derivatives

A mixture of BMTNE **3** (1 mmol) with amines (1 mmol for arylamine or diamine or 2 mmol for monoalkylamine or 1.5 mL of aqueous ammonia solution) in ethanol (5 mL) was heated at reflux for 6 h. Then to the above mixture, aryl glioxal **1** (1 mmol) and malono derivative **2** (1 mmol) were added to it and were heated for the time given in Table 1. After reaction completion was confirmed by TLC, the precipitate formed was filtered and washed with cold ethanol to afford the pure product **4a-4am**. The ^1^H and ^13^C NMR spectra are available as supplementary data.

#### 2-cyano-2-(8-nitro-6-(p-tolyl)-1,2,3,4-tetrahydropyrrolo[1,2-a]pyrimidin-7-yl)acetamide (4a)

Yellow powder; Yield: 92%, m.p: 238–240 °C; (TLC; n-hexane:EtOAc, 1:5, R_f_ = 0.22); IR (KBr): 3432, 3309 (NH), 3012, 20,877, 2341 (CN), 1739 (CO), 1670, 1623, 1877, 1511, 1403, 1365, 1353, 1211, 1145, 1068, 979, 821, 586. ^1^H NMR (500 MHz, DMSO*-d*_*6*_) δ: 8.30 (1H, s, NH), 7.41 (1H, s, NH), 7.32 (2H, d, *J* = 7.7 Hz, Ar), 7.26 (2H, d, *J* = 7.7 Hz, Ar), 7.18 (1H, s, NH), 4.66 (1H, s, CH), 3.57 (2H, m, CH_2_), 3.32 (2H, s CH_2_), 2.36 (3H, s, Me), 1.93 (2H, m, CH_2_); ^13^C NMR (125 MHz, DMSO*-d*_*6*_) δ: 166.0, 144.0, 139.2, 130.5, 129.9, 129.8, 125.0, 117.6, 114.7, 102.9, 41.9, 38.6, 36.3, 21.3, 20.4; MS (m/z): 339 [M^+^], 279, 249, 221, 193, 154, 131, 68, 44.

#### 2-cyano-2-(3,3-dimethyl-8-nitro-6-phenyl-1,2,3,4-tetrahydropyrrolo[1,2-a]pyrimidin-7-yl)acetamide (4b)

Pale yellow powder; Yield: 90%, m.p: 215–217 °C; (TLC; n-hexane:EtOAc, 1:5, R_f_ = 0.24); IR (KBr): 3386 (NH), 3181, 2965, 2886, 2341 (CN), 1739(CO), 1704, 1631, 1515, 1411, 1365, 1222, 1130, 1045, 933, 694; ^1^H NMR (500 MHz, DMSO*-d*_*6*_) δ: 8.38 (1H, s, NH), 7.47–7.54 (3H, m, Ar), 7.42 (1H, s, NH), 7.35 (2H, d, *J* = 7.2 Hz, Ar), 7.24 (1H, s, NH), 4.69 (1H, s, CH), 3.35 (1H, d, *J* = 12.3 Hz, CH), 3.26 (1H, d, *J* = 12.4 Hz, CH), 3.07–3.17 (2H, m, CH_2_), 0.97 (6H_,_ d, *J* = 9.0 Hz, Me); ^13^C NMR (125 MHz, DMSO*-d*_*6*_) δ: 165.9, 143.0, 130.6, 129.6, 129.6, 129.4, 127.9, 117.6, 114.5, 103.4, 52.6, 50.0, 36.5, 28.0, 24.0.

#### 2-cyano-2-(8-nitro-6-phenyl-1,2,3,4-tetrahydropyrrolo[1,2-a]pyrimidin-7-yl)acetamide (4c)

Yellow powder; Yield: 91%, m.p: 232–234 °C; (TLC; n-hexane:EtOAc, 1:5, R_f_ = 0.21); IR (KBr): 3428, 3328 (NH), 3154, 3016, 2873, 2341(CN), 1739 (CO), 1670, 1631, 1573, 1511, 1411, 1369, 1211, 1145, 1072, 636; ^1^H NMR (500 MHz, DMSO*-d*_*6*_) δ: 8.31 (1H, s,NH), 7.53–7.38 (6H, m, Ar), 7.21 (1H, s, NH), 4.69 (1H, s, CH), 3.66–3.61 (2H, m, CH_2_), 3.44–3.41 (2H, m, CH_2_), 1.93 (2H, m, CH_2_); ^13^C NMR (125 MHz, DMSO*-d*_*6*_) δ: 166.0, 144.0, 130.6, 129.8, 129.6, 129.3, 127.9, 117.6, 114.7, 103.1, 42.0, 38.6, 36.4, 20.3.

#### Ethyl3-amino-2-(3,3-dimethyl-8-nitro-6-phenyl-1,2,3,4-tetrahydropyrrolo[1,2-a]pyrimidin-7-yl)-3-oxopropanoate (4d)

Yellow powder; Yield: 88%, m.p: 200–202 °C; (TLC; n-hexane:EtOAc, 1:5, R_f_ = 0.28); IR (KBr): 3725, 3625, 3266 (NH), 3162, 2969, 2854, 1731 (CO), 1685 (CO), 1623, 1511, 1400, 1276, 1211, 1122, 1033, 647; ^1^HNMR (500 MHz, DMSO*-d*_*6*_) δ: 8.38 (1H, s, NH), 7.46–7.41 (3H, m, Ar),7.37–738 (2H, m, Ar), 7.11 (1H, s, NH), 4.47 (1H, s, CH), 3.85–3.76 (2H, m, OCH_2_), 3.36 (1H, d, *J* = 12.3 Hz, CH_2_), 3.28 (1H, d, *J* = 12.1 Hz, CH_2_), 3.14 (2H, s, CH_2_), 1.02 (3H, t, *J* = 7.1 Hz, Me), 0.93 (6H, d, *J* = 12.0 Hz, Me); ^13^C NMR (125 MHz, DMSO*-d*_*6*_) δ: 169.2, 168.9, 143.1, 130.8, 130.0, 128.9, 128.7, 128.6, 115.5, 107.5, 60.9, 52.7, 50.1, 49.8, 27.9, 23.9, 23.8, 14.3.

#### 2-(6-(4-chlorophenyl)-3,3-dimethyl-8-nitro-1,2,3,4-tetrahydropyrrolo[1,2-a]pyrimidin-7-yl)-2-cyanoacetamide (4e)

Yellow powder; Yield: 94%, m.p: 144–146 °C; (TLC; n-hexane:EtOAc, 1:5, R_f_ = 0.23); ^1^H NMR (500 MHz, DMSO*-d*_*6*_) δ: 8.38 (1H, s, NH), 7.59–7.57 (2H, m, Ar), 7.42 (1H, s, NH), 7.39–7.33 (2H, m, Ar), 7.26 (1H, s, NH), 4.82 (1H, s, CH), 3.35–3.3.24 (2H, m, CH_2_), 3.15–3.08 (2H, m, CH_2_), 0.96 (6H, d, *J* = 5 Hz, Me); ^13^C NMR (125 MHz, DMSO*-d*_*6*_) δ: 165.89, 143.04, 134.54, 132.59, 129.50, 128.31, 126.87, 117.62, 114.60, 103.87, 52.58, 49.95, 36.27, 28.00, 24.02, 24.01.

#### 2-cyano-2-(6-(3-methoxyphenyl)-8-nitro-1,2,3,4-tetrahydropyrrolo[1,2-a]pyrimidin-7-yl)acetamide (4f.)

Yellow powder; Yield: 85%, m.p: 230–232 °C; (TLC; n-hexane:EtOAc, 1:5, R_f_ = 0.23); IR (KBr): 3440, 3370, 3278, (NH), 3154, 3016, 2965, 2881, 2136 (CN), 1739(CO), 1685, 1623, 1515, 1407, 1365, 1218, 1153, 1091, 1029, 871, 790, 694; ^1^H NMR (500 MHz, DMSO*-d*_*6*_) δ: 8.31 (1H, s, NH), 7.38–7.46 (2H, m, Ar), 7.20 (1H, s, NH), 7.05 (1H, dd, *J* = 8.4, 2.5 Hz, Ar), 6.98–6.92 (2H, m, Ar), 4.75 (1H, s, CH), 3.78 (3H, s, OMe), 3.67–354 (2H, m, CH_2_), 3.43–3.32 (2H, m, CH_2_), 2.0–1.87 (2H, m, CH_2_); ^13^C NMR (125 MHz, DMSO*-d*_*6*_) δ: 166.0, 159.7, 143.9, 130.5, 129.6, 129.2, 122.7, 117.6, 116.1, 115.2, 114.7, 103.1, 55.6, 42.0, 38.6, 36.3, 20.3.

#### Ethyl 3-amino-2-(8-nitro-6-phenyl-1,2,3,4-tetrahydropyrrolo[1,2-a]pyrimidin-7-yl)-3-oxopropanoate (4 g)

Yellow powder, Yield: 88%, m.p: 215–217 °C; (TLC; n-hexane:EtOAc, 1:5, R_f_ = 0.28); IR (KBr): 3367, 3193 (NH), 2996, 2954, 2896, 1731 (CO), 1673, 1623, 1508, 1396, 1276, 1222, 1103, 1072, 748, 593, 435; ^1^H NMR (500 MHz, DMSO*-d*_*6*_) δ: 8.31 (1H, s, NH), 7.40–7.46 (6H, m, Ar), 7.34 (1H, s, NH), 7.13 (1H, s, NH), 4.46 (1H, s, CH), 3.87–377 (2H, m, CH_2_), 3.64–3.59 (2H, m, CH_2_), 3.44–342 (2H, q, *J* = 4.3 Hz, OCH_2_), 1.94–1.89 (2H, m, CH_2_), 1.03 (3H, t, *J* = 7.0 Hz, Me); ^13^C NMR (125 MHz, DMSO*-d*_*6*_) δ: 169.3, 169.0, 144.0, 130.8, 129.0, 128.8, 128.8, 128.7, 115.7, 107.2, 60.9, 50.0, 41.9, 38.5, 20.5, 14.3.

#### 2-cyano-2-(3,3-dimethyl-8-nitro-6-(p-tolyl)-1,2,3,4-tetrahydropyrrolo[1,2-a]pyrimidin-7-yl)acetamide (4 h)

Yellow powder; yield: 92%, m.p: 236–238 °C; (TLC; n-hexane:EtOAc, 1:5, R_f_ = 0.22); IR (KBr): 3390, 3313, 3178, 3019, 2965, 2923, 2345 (CN), 1739 (CO), 1704, 1631, 1515, 1411, 1365, 1222, 1133, 1045, 601, 416; ^1^H NMR (500 MHz, DMSO*-d*_*6*_) δ: 8.36 (1H, s, NH), 7.41 (1H, s, NH), 7.32 (2H, d, *J* = 7.6 Hz, Ar), 7.23 (3H, d, *J* = 8.5 Hz, Ar), 4.67 (1H, s, CH), 3.32 (1H, s, CH_2_), 3.24 (1H, d, *J* = 12.3 Hz, CH_2_), 3.07–3.17 (2H, m, CH_2_), 2.36 (3H, s, Me), 0.96 (6H, d, *J* = 8.2 Hz, Me); ^13^C NMR (125 MHz, DMSO*-d*_*6*_) δ: 165.9, 143.0, 139.2, 139.2, 130.5, 130.0, 129.7, 124.9, 117.7, 114.5, 103.1, 52.6, 49.9, 36.4, 28.0, 24.0, 21.4.

#### 2-(6-(4-chlorophenyl)-8-nitro-1,2,3,4-tetrahydropyrrolo[1,2-a]pyrimidin-7-yl)-2-cyanoacetamide(4i)

Yellow powder; Yield: 90%, m.p: 218–220 °C; (TLC; n-hexane:EtOAc, 1:5, R_f_ = 0.23); IR (KBr): 3432, 3347, 3301 (NH), 3162, 2969, 2144 (CN), 1739 (CO), 1677, 1637, 1573, 1511, 1365, 1295, 1214, 1137, 821, 601, 532; ^1^H NMR (499 MHz, DMSO*-d*_*6*_) δ: 8.32 (1H, s, NH), 7.58 (2H, d, *J* = 10 Hz, Ar), 7.44–7.37 (3H, m, Ar), 7.21 (1H, s, NH), 4.81 (1H, s, CH), 3.62 -3.53 (2H, m, CH_2_), 3.41 (2H, q, *J* = 4.7, 4.1 Hz, CH_2_), 1.99–1.87 (2H, m, CH_2_); ^13^C NMR (126 MHz, DMSO*-d*_*6*_) δ: 165.94, 143.98, 134.51, 132.57, 129.41, 128.47, 126.96, 117.57, 114.78, 103.64, 41.92, 38.61, 36.19, 20.36.

#### Methyl3-amino-2-(3,3-dimethyl-8-nitro-6-(p-tolyl)-1,2,3,4-tetrahydropyrrolo[1,2-a]pyrimidin-7-yl)-3-oxopropanoate (4j)

Yellow powder; Yield: 87%, m.p: 229–231 °C; (TLC; n-hexane:EtOAc, 1:5, R_f_ = 0.29); IR (KBr): 3448, 3332 (NH), 3016, 2965, 1735 (CO), 1631, 1515, 1419, 1365, 1214, 1114, 1041, 532; ^1^H NMR (500 MHz, DMSO*-d*_*6*_) δ: 8.36 (1H, s, NH), 7.31 (1H, s, NH), 7.27–7.23 (4H, m, Ar), 7.11 (1H, s, NH), 4.44 (1H, s, CH), 3.39 (3H, s, OMe), 3.35 (1H, s, CH_2_), 3.24 (1H, d, *J* = 12.3 Hz, CH_2_), 3.13 (2H, d, *J* = 3.0 Hz, CH_2_), 2.34 (3H, s, Me), 0.95 (3H, s, Me), 0.91 (3H, s, Me); ^13^C NMR (125 MHz, DMSO*-d*_*6*_) δ: 169.8, 168.9, 143.1, 138.6, 130.7, 130.2, 130.1, 129.6, 128.8, 125.6, 115.4, 107.1, 52.6, 52.3, 49.9, 49.8, 27.9, 24.0, 23.8, 21.3; MS (m/z): 400 [M^+^], 324, 280, 251, 221, 194, 167, 142, 119, 91, 69, 44.

#### Methyl3-amino-2-(6-(4-chlorophenyl)-3,3-dimethyl-8-nitro-1,2,3,4-tetrahydropyrrolo[1,2-a]pyrimidin-7-yl)-3-oxopropanoate (4 k)

Yellow powder; Yield: 89%, m.p: 220–222 °C; (TLC; n-hexane:EtOAc, 1:5, R_f_ = 0.28); IR (KBr): 3448, 3332 (NH), 3016, 2965, 1735 (CO), 1631, 1515, 1419, 1365, 1214, 1114, 1045, 860, 740, 532; ^1^H NMR (500 MHz, DMSO*-d*_*6*_) δ: 8.38 (1H, s, NH), 7.51–7.36 (4H, m, Ar), 7.36 (1H, s, NH), 7.13 (1H, s, NH), 4.61 (1H, s, CH), 3.85–3.76 (2H, m, CH_2_), 3.38 (3H, s, OMe), 3.33–3.26 (2H, m, CH_2_), 3.12 (2H, s, CH_2_), 0.93 (6H, d, *J* = 12.0 Hz, Me); ^13^C NMR (125 MHz, DMSO*-d*_*6*_) δ: 169.2, 168.9, 143.1, 130.8, 129.0, 128.9, 128.7, 128.6, 115.5, 107.5, 60.9, 52.7, 50.1, 49.8, 27.9, 23.9, 23.8, 14.3; MS (m/z): 420 [M^+^], 377, 331, 300, 258, 216, 189, 162, 138, 111, 69, 44.

#### Ethyl3-amino-2-(3,3-dimethyl-8-nitro-6-(p-tolyl)-1,2,3,4-tetrahydropyrrolo[1,2-a]pyrimidin-7-yl)-3-oxopropanoate (4 l)

Pale yellow powder; Yield: 84%, m.p: 240–242 °C; (TLC; n-hexane:EtOAc, 1:5, R_f_ = 0.29); IR (KBr): 3445, 3326 (NH), 3020, 2980, 1739 (CO), 1635, 1515, 1430, 1368, 1212, 1122, 1041, 866, 740, 532; ^1^H NMR (500 MHz, DMSO*-d*_*6*_) δ: 8.37 (1H, s, NH), 7.31 (1H, s, NH), 7.28–723 (4H, m, Ar), 7.10 (1H, s, NH), 4.43 (1H, s, CH), 3.87–383 (2H, m, CH_2_), 3.36–3.24 (2H, m, CH_2_), 3.13 (2H, s, CH_2_), 2.33 (3H, s, Me), 1.08–1.00 (3H, t, *J* = 7 Hz, Me), 0.93 (6H, d, *J* = 16.2 Hz, Me); ^13^C NMR (125 MHz, DMSO*-d*_*6*_) δ: 169.3, 169.0, 143.1, 138.6, 130.7, 129.5, 128.8, 125.7, 115.5, 107.2, 60.9, 52.7, 50.1, 49.8, 27.9, 24.0, 23.8, 21.3, 14.3.

#### 2-(10-(4-chlorophenyl)-8-nitro-7H-pyrrolo[1,2-a]perimidin-9-yl)-2-cyanoacetamide (4 m)

yellow powder; Yield: 85%, m.p: 248–250 °C; (TLC; n-hexane:EtOAc, 1:5, R_f_ = 0.24); IR (KBr): 3428, 3289 (NH), 3046, 2904, 2194 (CN), 1735 (CO), 1646, 1604, 1519, 1446, 1403, 1272, 1222, 1099, 1014, 968, 890, 817, 763; ^1^H NMR (500 MHz, DMSO*-d*_*6*_) δ: 11.42 (1H, s, NH), 7.69 (2H, s, Ar), 7.58–7.51 (5H, m, Ar), 7.40 (2H, d, *J* = 4.3 Hz), 7.32 (1H, d, *J* = 8.3 Hz), 7.13 (1H, t, *J* = 8.1 Hz, Ar), 6.16 (1H, d, *J* = 7.7 Hz), 4.43 (1H, s, CH); ^13^C NMR (125 MHz, DMSO*-d*_*6*_) δ: 167.6, 147.7, 135.4, 135.0, 134.4, 132.2, 130.6, 130.2, 128.6, 127.4, 127.1, 121.9, 121.5, 118.4, 116.5, 110.2, 108.1, 107.7, 101.4, 55.3, 51.8; MS (m/z): 443 [M^+^], 414, 396, 368,332, 299, 382, 210, 193, 166, 140, 113, 91, 63, 44.

#### Ethyl3-amino-2-(2-(4-chlorophenyl)-1-(furan-2-ylmethyl)-5-((furan-2-ylmethyl)amino)-4-nitro-1H-pyrrol-3-yl)-3-oxopropanoate (4n)

Yellow powder; Yield: 93%, m.p: 154–156 °C; (TLC; n-hexane:EtOAc, 1:5, R_f_ = 0.38); IR (KBr): 3397, 3266, 3201 (NH), 2981, 2877, 2773, 1724 (CO), 1677, 1873, 1527, 1415, 1353, 1253, 1207, 1133, 1014, 833, 740, 663; ^1^H NMR (500 MHz, DMSO*-d*_*6*_) δ: 7.68–7.63 (2H, m, Ar), 7.54–7.47 (3H, m, Ar), 7.35–7.28 (3H, m, Ar), 7.11 (1H,s, NH), 6.43 (1H, s, NH), 6.33 (2H, s, CH_2_), 5.91 (1H, s), 5.01 (2H, s, CH_2_), 4.66 (2H, d, *J* = 6.3 Hz), 4.50 (1H, s), 3.91–3.83 (2H, m, CH_2_), 1.05 (td, *J* = 7.1, 1.5 Hz, 3H); ^13^C NMR (125 MHz, DMSO*-d*_*6*_) δ: 169.2, 168.1, 151.8, 149.1, 145.2, 143.4, 143.4, 134.3, 133.2, 128.9, 128.3, 127.9, 120.2, 111.0, 111.0, 110.7, 108.5, 108.3, 61.0, 49.6, 43.0, 42.2, 14.3.

#### 2-cyano-2-(1-(furan-2-ylmethyl)-5-((furan-2-ylmethyl)amino)-4-nitro-2-(p-tolyl)-1H-pyrrol-3-yl)acetamide (4o)

Yellow powder; Yield: 95%, m.p: 159–161 °C; (TLC; n-hexane:EtOAc, 1:5, R_f_ = 0.33); IR (KBr): 3482, 3359, 3274 (NH), 3124, 3023, 2969, 2892, 1704 (CO), 1589, 1523, 1415, 1353, 1199, 1114, 1010, 825, 659, 485; ^1^H NMR (500 MHz, DMSO*-d*_*6*_) δ: 7.76 (1H, t, *J* = 6.3 Hz), 7.64 (1H, s), 7.5 (1H, s), 7.43 (1H, s), 7.30–7.19 (5H, m, Ar), 6.46–6.41 (1H, s), 6.36 (2H, s), 6.08 (1H, s), 5.07–4.94 (2, m), 4.65 (2H, d, *J* = 6.3 Hz), 4.58 (1H, s), 2.36 (3H, s, Me); ^13^C NMR (125 MHz, DMSO*-d*_*6*_) δ: 165.4, 151.7, 149.1, 145.2, 143.5, 143.4, 139.7, 138.5, 131.2, 131.1, 129.9, 125.0, 118.8, 117.4, 111.1, 111.1, 108.7, 108.3, 105.9, 42.7, 42.2, 36.5, 21.4.

#### 2-(1-benzyl-5-(benzylamino)-2-(4-chlorophenyl)-4-nitro-1H-pyrrol-3-yl)-2-cyanoacetamide (4p)

Yellow powder; Yield: 96%, m.p: 138–140 °C; (TLC; n-hexane:EtOAc, 1:5, R_f_ = 0.36); IR (KBr): 3455, 3378, 3259 (NH), 3189, 3023, 2969, 1739 (CO), 1689, 1619, 1523, 1407, 1361, 1226, 1103, 1018, 848, 690; ^1^H NMR (500 MHz, DMSO*-d*_*6*_) δ: 8.16 (1H, t, *J* = 6.5 Hz), 7.50–7.43 (3H, m, Ar), 7.37–7.24 (10H, m, Ar), 7.20 (2H, d, *J* = 7.5 Hz, Ar), 6.99 (2H, d, *J* = 7.4 Hz, Ar), 5.04 (1H, d, *J* = 18.0 Hz), 4.96 (1H, d, *J* = 18.0 Hz), 4.81 (1H, s), 4.50 (2H, d, *J* = 6.5 Hz); ^13^C NMR (125 MHz, DMSO*-d*_*6*_) δ: 165.5, 146.2, 138.8, 136.9, 135.0, 133.1, 129.9, 129.3, 129.2, 129.1, 128.0, 127.9, 127.3, 126.9, 126.0, 118.1, 117.4, 106.3, 48.8, 48.4, 36.6.; MS (m/z): 499 [M^+^], 436, 409, 383, 347, 320, 292, 256, 187, 163, 140, 116, 91, 65.

#### Ethyl3-amino-2-(1-(furan-2-ylmethyl)-5-((furan-2-ylmethyl)amino)-4-nitro-2-(p-tolyl)-1H-pyrrol-3-yl)-3-oxopropanoate (4q)

Yellow powder; Yield: 90%, m.p: 170–172 °C; (TLC; n-hexane:EtOAc, 1:5, R_f_ = 0.38); IR (KBr): 3397, 3270 (NH), 3193, 2977, 2931, 2874, 1720 (CO), 1677, 1573, 1407, 1353, 1126, 1014, 917, 825, 744, 655; ^1^H NMR (500 MHz, DMSO*-d*_*6*_) δ: 7.74 (1H, t, *J* = 6.4 Hz), 7.63 (1H, s), 7.54 (1H, s), 7.31 (1H, s), 7.24 (2H, d, *J* = 7.6 Hz), 7.17 (2H, d, *J* = 7.7 Hz), 7.12 (1H, s), 6.43 (2H, q, *J* = 2.2 Hz), 6.34 (2H, q, *J* = 2.9, 2.3 Hz), 5.91 (1H, d, *J* = 3.3 Hz), 5.01 (2H, s), 4.66 (2H, d, *J* = 6.3 Hz), 4.37 (1H, d, *J* = 1.6 Hz), 3.90 (2H, q, *J* = 7.0 Hz), 1.06 (3H, td, *J* = 7.2, 1.6 Hz); ^13^C NMR (125 MHz, DMSO*-d*_*6*_) δ: 169.3, 168.2, 151.8, 149.3, 145.1, 143.4, 139.2, 138.9, 131.2, 129.9, 129.5, 125.9, 120.1, 111.0, 111.0, 110.2, 108.3, 108.2, 61.0, 49.8, 42.8, 42.2, 21.3, 14.3.

#### 2-(1-benzyl-5-(benzylamino)-4-nitro-2-(p-tolyl)-1H-pyrrol-3-yl)-2-cyanoacetamide (4r)

Yellow powder; Yield: 95%, m.p: 236–238 °C; (TLC; n-hexane:EtOAc, 1:5, R_f_ = 0.32); IR (KBr): 3482, 3359, 3274 (NH), 3124, 3023, 2973, 2896, 1704 (CO), 1589, 1523, 1415, 1353, 1199, 1114, 1010, 937, 825, 752; ^1^H NMR (500 MHz, DMSO*-d*_*6*_) δ: 7.52 (1H, s, NH), 7.30–738 (5H, m, Ar), 7.24 (d, *J* = 7.5 Hz, 2H), 7.18–7.12 (m, 2H), 4.76 (s, 1H), 2.30 (d, *J* = 1.8 Hz, 3H); ^13^C NMR (125 MHz, DMSO*-d*_*6*_) δ: 165.9, 146.7, 139.3, 134.0, 132.8, 131.5, 130.6, 129.0, 129.0, 128.9, 127.4, 117.5, 115.2, 104.8, 36.8, 21.2.

#### 2-(2-(4-chlorophenyl)-1-(furan-2-ylmethyl)-5-((furan-2-ylmethyl)amino)-4-nitro-1H-pyrrol-3-yl)-2-cyanoacetamide (4 s)

Yellow powder; Yield: 97%, m.p: 238–240 °C; (TLC; n-hexane:EtOAc, 1:5, R_f_ = 0.32); IR (KBr): 3448, 3355, 3274 (NH), 3016, 2969, 1739 (CO), 1581, 1519, 1419, 1361, 1214, 1118, 1006, 933, 717, 659; ^1^H NMR (500 MHz, DMSO*-d*_*6*_) δ:7.75–7.69 (1H, s, NH), 7.64 (1H, d, *J* = 2.0 Hz, NH), 7.58–7.51 (3H, m, Ar), 7.44 (1H, s, NH), 7.32 (2H, d, *J* = 7.8 Hz, Ar), 7.23 (1H, s, NH), 6.44 (1H, q, *J* = 2.3 Hz), 6.36 (2H, t, *J* = 2.6 Hz), 6.08 (1H, t, *J* = 2.6 Hz), 5.08 (d, *J* = 17.3 Hz, 1H), 4.97 (d, *J* = 17.4 Hz, 1H), 4.75 (d, *J* = 2.1 Hz, 1H), 4.66 (dd, *J* = 6.3, 2.0 Hz, 2H); ^13^C NMR (125 MHz, DMSO*-d*_*6*_) δ: 165.3, 151.7, 148.9, 145.3, 143.6, 143.4, 139.2, 135.0, 133.2, 129.6, 129.3, 127.0, 119.0, 117.3, 111.1, 111.1, 108.9, 108.4, 106.5, 42.9, 42.2, 36.3; MS (m/z): 479 [M^+^], 416, 391, 337, 310, , 282, 236, 163, 139, 111, 81, 44.

#### Ethyl 3-amino-2-(1-benzyl-5-(benzylamino)-4-nitro-2-(p-tolyl)-1H-pyrrol-3-yl)-3-oxopropanoate (4t)

Yellow powder; Yield: 93%, m.p: 157–159 °C; (TLC; n-hexane:EtOAc, 1:5, R_f_ = 0.34); IR (KBr): 3459, 3301 (NH), 3170, 3027, 2933, 2877, 1674(CO), 1581, 1531, 1423, 1346, 1245, 1114, 979, 817, 725, 698; ^1^H NMR (500 MHz, DMSO*-d*_*6*_) δ: 8.17 (1H, t, *J* = 6.6 Hz), 7.46–7.08 (14H, m, Ar), 6.91–6.86 (2H, m, Ar), 6.41 (1H, d, *J* = 1.8 Hz, 1H), 5.00 (2H, s), 4.52 (2H, d, *J* = 6.4 Hz), 4.42 (1H, s), 3.92 (2H, q, *J* = 7.1 Hz), 2.28 (3H, d, *J* = 2.7 Hz), 1.08 (3H, t, *J* = 7.1, 3.6 Hz); ^13^C NMR (125 MHz, DMSO*-d*_*6*_) δ: 169.4, 168.3, 156.3, 146.0, 139.0, 138.8, 137.4, 131.1, 130.4, 129.5, 129.1, 128.0, 127.8, 127.4, 125.9, 125.7, 119.1, 110.0, 98.3, 61.0, 49.8, 48.7, 48.2, 21.3, 14.3.

#### 2-(5-amino-4-nitro-2-phenyl-1H-pyrrol-3-yl)-2-cyanoacetamide (4u)

Yellow powder; Yield: 90%, m.p: 236–238 °C; (TLC; n-hexane:EtOAc, 1:5, R_f_ = 0.20); IR (KBr): 3594, 3448, 3328 (NH), 3016, 2969, 1743 (CO), 1689, 1650, 1589, 1496, 1400, 1365, 1284, 1218, 1133, 1072, 917, 771; ^1^H NMR (300 MHz, DMSO*-d*_*6*_) δ:11.36 (1H, s, NH), 7.54–7.39 (6H, m, Ar), 7.36–7.29 (3H, m, Ar), 5.05 (1H, s, CH); ^13^C NMR (75 MHz, DMSO*-d*_*6*_) δ: 166.1, 146.7, 130.0, 129.3, 128.9, 128.6, 127.1, 117.9, 116.4, 102.4, 36.4.

#### 2-(5-amino-4-nitro-2-(p-tolyl)-1H-pyrrol-3-yl)-2-cyanoacetamide (4v)

Yellow powder; Yield: 93%, m.p: 236–238 °C; (TLC; n-hexane:EtOAc, 1:5, R_f_ = 0.18); IR (KBr): 3417, 3297, 3259, 3023, 2969, 2877, 1735 (CO), 1670, 1639, 1477, 1396, 1365, 1280, 1157, 1126, 1076, 887, 755; ^1^H NMR (500 MHz, DMSO*-d*_*6*_) δ: 11.27 (1H, s, NH), 7.41 (1H, s, NH), 7.32–7.28 (6H, m, Ar), 7.27–7.19 (1H, m, Ar), 5.04 (1H, s, CH), 2.37–2.33 (3H, m, Me); ^13^C NMR (125 MHz, DMSO*-d*_*6*_) δ: 166.17, 157.97, 146.70, 141.46, 138.51, 137.67, 129.87, 129.33, 128.48, 127.19, 127.17, 125.28, 117.92, 116.43, 108.38, 102.05, 88.32, 74.54, 36.44, 21.33, 21.10, 19.03; ; MS (m/z): 299 [M],239, 194, 169, 140, 118, 91, 44.

#### 2-(5-amino-2-(4-chlorophenyl)-4-nitro-1H-pyrrol-3-yl)-2-cyanoacetamide (4w)

Yellow powder; Yield: 95%, m.p: 236–238 °C; (TLC; n-hexane:EtOAc, 1:5, R_f_ = 0.20); IR (KBr): 3259 (NH), 2881, 1673 (CO), 1484, 1392, 1272, 1211, 1126, 1049, 925, 817, 613; ^1^H NMR (500 MHz, DMSO*-d*_*6*_) δ: 11.33 (1H, s, NH), 7.51–7.13 (8H, m, Ar), 5.05 (1H, s, CH); ^13^C NMR (125 MHz, DMSO*-d*_*6*_) δ: 166.1, 146.7, 130.0, 129.3, 128.9, 128.6, 127.0, 117.8, 116.4, 102.4, 36.4.

#### 2-(5-amino-2-(4-methoxyphenyl)-4-nitro-1H-pyrrol-3-yl)-2-cyanoacetamide (4x)

Yellow powder; Yield: 92%, m.p: 236–238 °C; (TLC; n-hexane:EtOAc, 1:5, R_f_ = 0.18); IR (KBr): 3305, 3259, 3208 (NH), 2877, 1677 (CO), 1481, 1392, 1280, 1130, 1049, 921, 817, 759; ^1^H NMR (300 MHz, DMSO*-d*_*6*_) δ: 11.29 (1H, s, NH), 7.44 (1H, s, NH), 7.37–7.32 (3H, m, Ar), 7.28–7.24 (1H, m, Ar), 7.11–7.04 (2H, m, Ar), 7.01–6.85 (1H, m, Ar), 5.01 (1H, s, CH), 3.81 (3H, s, OMe); ^13^C NMR (75 MHz, DMSO*-d*_*6*_) δ: 166.27, 157.92, 146.67, 136.36, 130.07, 129.15, 127.22, 126.65, 122.29, 117.97, 116.32, 114.78, 114.11, 113.00, 108.38, 101.54, 91.86, 88.26, 74.58, 55.75, 55.61, 36.47.

#### 2-(5-amino-2-(naphthalen-2-yl)-4-nitro-1H-pyrrol-3-yl)-2-cyanoacetamide (4y)

Yellow powder; Yield: 82%, m.p: 270–272 °C; (TLC; n-hexane:EtOAc, 1:5, R_f_ = 0.22); ^1^H NMR (300 MHz, DMSO*-d*_*6*_) δ: 11.49 (s, 1H), 8.06–7.95 (4H, m, Ar), 7.63–7.54 (3H, m, Ar), 7.49–7.35 (4H, m, Ar), 5.25 (1H, s, CH); ^13^C NMR (75 MHz, DMSO*-d*_*6*_) δ: 166.2, 146.8, 133.1, 132.9, 128.8, 128.5, 128.1, 127.6, 127.2, 127.2, 127.0, 126.3, 117.9, 116.6, 103.0, 36.4.

#### 2-cyano-2-(1-(3,4-dimethylphenyl)-5-(methylthio)-4-nitro-2-(p-tolyl)-1H-pyrrol-3-yl)acetamide (4z)

Yellow powder; Yield: 92%, m.p: 188–190 °C; (TLC; n-hexane:EtOAc, 1:2, R_f_ = 0.22); ^1^H NMR (300 MHz, DMSO*-d*_*6*_) δ: 7.64 (1H, s, NH_2_), 7.41 (1H, d, *J* = 4.3 Hz), 7.19–7.08 (m, 6H, Ar), 4.75 (1H, s, CH), 2.33 (3H, d, *J* = 2.7 Hz, SMe), 2.26–2.20 (9H, m, Me); ^13^C NMR (75 MHz, DMSO*-d*_*6*_) δ: 165.2, 139.2, 137.9, 137.4, 137.3, 136.5, 134.4, 131.0, 130.2, 129.9, 129.5, 126.5, 125.6, 117.1, 108.7, 108.6, 36.7, 21.3, 19.8, 19.7, 19.5, 19.1; MS (m/z): 434 [M^+^], 388, 298, 222, 222, 163, 142, 105, 79, 44.

#### 2-(2-(4-chlorophenyl)-1-(4-methoxyphenyl)-5-(methylthio)-4-nitro-1H-pyrrol-3-yl)-2-cyanoacetamide (4aa)

Yellow powder; Yield: 93%, m.p: 204–206 °C; (TLC; n-hexane:EtOAc, 1:2, R_f_ = 0.22); ^1^H NMR (500 MHz, DMSO*-d*_*6*_) δ: 7.61 (1H, s, NH_2_), 7.45–7.29 (5H, m, Ar), 7.28–7.20 (3H, m, Ar), 6.97–6.91 (2H, m, Ar), 4.93 (1H, s, CH), 3.76 (3H, s, OMe), 2.31 (3H, s, SMe): ^13^C NMR (125 MHz, DMSO*-d*_*6*_) δ: 165.3, 159.8, 136.5, 136.2, 134.6, 133.1, 130.4, 130.4, 129.2, 129.0, 127.5, 117.1, 114.4, 109.0, 55.8, 36.6, 19.0; MS (m/z): 456 [M^+^], 413, 355, 320, 244, 201, 165, 121, 77, 44.

#### Ethyl3-amino-2-(1-(3,4-dimethylphenyl)-5-(methylthio)-4-nitro-2-phenyl-1H-pyrrol-3-yl)-3-oxopropanoate (4ab)

Yellow powder; Yield: 85%, m.p: 208–210 °C; (TLC; n-hexane:EtOAc, 1:2, R_f_ = 0.23); ^1^H NMR (300 MHz, DMSO*-d*_*6*_) δ: 7.40 (1H, s, NH_2_), 7.33–7.26 (3H, m, Ar), 7.25–6.88 (6H, m, Ar), 4.54 (1H, s, CH), 4.05–3.88 (2H, m, OCH_2_), 2.30 (3H, s, SMe), 2.17 (6H, d, *J* = 15.5 Hz, Me), 1.13 (3H, t, *J* = 7.1 Hz, Me); ^13^C NMR (75 MHz, DMSO*-d*_*6*_) δ: 168.9, 168.0, 138.7, 137.0, 137.4, 136.3, 134.5, 131.3, 130.1, 129.9, 129.4, 129.1, 128.5, 127.0, 126.5, 112.3, 61.3, 49.7, 19.6, 19.4, 14.3.

#### Ethyl 3-amino-2-(2-(4-chlorophenyl)-1-(4-methoxyphenyl)-5-(methylthio)-4-nitro-1H-pyrrol-3-yl)-3-oxopropanoate (4ac)

Pale green powder; Yield: 89%, m.p: 180–182 °C; (TLC; n-hexane:EtOAc, 1:2, R_f_ = 0.21); ^1^H NMR (500 MHz, DMSO*-d*_*6*_) δ: 8.15 (1H, s, NH_2_), 7.59–738 (8H, m, Ar), 6.99 (1H, s), 4.80 (1H, s, CH), 4.21–4.11 (2H, m, OCH_2_), 3.76 (3H, s, OMe), 2.01 (3H, s, SMe),1.22 (3H, s, Me); ^13^C NMR (125 MHz, DMSO*-d*_*6*_) δ: 165.6, 164.1, 159.6, 142.6, 129.5, 129.3, 129.0, 125.7, 116.8, 115.0, 114.7, 97.9, 62.4, 55.9, 55.8, 53.4, 36.8, 16.5, 14.2.

#### 2-(2-(4-chlorophenyl)-1-(4-hydroxyphenyl)-5-(methylthio)-4-nitro-1H-pyrrol-3-yl)-2-cyanoacetamide (4ad)

Yellow powder; Yield: 92%, m.p: 157–159 °C; (TLC; n-hexane:EtOAc, 1:2, R_f_ = 0.20); ^1^H NMR (500 MHz, DMSO*-d*_*6*_) δ: 9.85 (1H, s, OH), 7.57 (1H, s, NH_2_), 7.40 (2H, d, *J* = 8.1 Hz, Ar), 7.37 (1H, s), 7.19 (2H, d, *J* = 8.2 Hz, Ar), 7.17–7.07 (2H, m, Ar), 6.73–670 (2H, m, Ar), 4.90 (1H, s, CH), 2.28 (3H, s, SMe); ^13^C NMR (125 MHz, DMSO*-d*_*6*_) δ: 165.3, 158.2, 136.3, 136.3, 134.5, 133.1, 130.4, 130.3, 128.9, 127.7, 127.6, 117.1, 115.8, 108.8, 36.6, 18.9; MS (m/z): 442 [M^+^], 396, 368, 340, 306, 230, 174, 151, 126, 93, 44.

#### 2-cyano-2-(1-(4-hydroxyphenyl)-5-(methylthio)-4-nitro-2-(p-tolyl)-1H-pyrrol-3-yl)acetamide (4ae)

Pale yellow powder; Yield: 92%, m.p: 160–162 °C; (TLC; n-hexane:EtOAc, 1:2, R_f_ = 0.22); ^1^H NMR (500 MHz, DMSO*-d*_*6*_) δ: 9.80 (1H, s, OH), 7.57 (1H, s, NH_2_), 7.36 (1H, s, NH_2_), 7.18–7.03 (6H, m, Ar), 6.70 (2H, d, *J* = 10 Hz, Ar), 4.72 (1H, s, CH), 2.28 (3H, s, SMe), 2.25 (3H, s, Me); ^13^C NMR (125 MHz, DMSO*-d*_*6*_) δ:165.30, 158.16, 139.14, 137.69, 136.32, 131.06, 130.38, 130.36, 130.16, 129.51, 127.97, 125.72, 117.20, 115.74, 108.42, 36.76, 21.30, 19.03, 19.00.

#### Methyl 3-amino-2-(5-(methylthio)-4-nitro-2-phenyl-1-(p-tolyl)-1H-pyrrol-3-yl)-3-oxopropanoate (4af)

Pale green powder; Yield: 85%, m.p: 178–180 °C; (TLC; n-hexane:EtOAc, 1:2, R_f_ = 0.23); ^1^H NMR (300 MHz, DMSO*-d*_*6*_) δ: 7.43(1H, m), 7.32–7.06 (m, 8H), 4.58 (1H, s, CH), 3.51 (3H, s, OMe), 2.29 (6H, s, SMe, Me); ^13^C NMR (75 MHz, DMSO*-d*_*6*_) δ: 169.4, 167.9, 138.9, 138.8, 136.3, 134.3, 131.4, 129.8, 129.3, 129.2, 129.0, 128.6, 127.1, 112.2, 52.6, 49.5, 21.1, 19.6.

#### 2-cyano-2-(5-(methylthio)-4-nitro-1,2-di-p-tolyl-1H-pyrrol-3-yl)acetamide (4ag)

Pale yellow powder; Yield: 89%, m.p: 216–218 °C; (TLC; n-hexane:EtOAc, 1:2, R_f_ = 0.22); ^1^H NMR (500 MHz, DMSO*-d*_*6*_) δ: 7.61 (1H, s), 7.39 (1H, s), 7.29 (1H, d, *J* = 8.2 Hz), 7.22–7.18 (3H, m, Ar), 7.15–7.06 (4H, m, Ar), 4.75 (1H, s, CH), 2.31–2.24 (9H, m, SMe, Me); ^13^C NMR (125 MHz, DMSO*-d*_*6*_) δ: 165.2, 139.2, 139.1, 137.4, 136.6, 134.3, 131.0, 129.8, 129.5, 129.0, 125.5, 117.1, 108.6, 36.7, 21.3, 2.1, 19.1.

#### 2-(2-(4-chlorophenyl)-5-(methylthio)-4-nitro-1-(p-tolyl)-1H-pyrrol-3-yl)-2-cyanoacetamide (4ah)

Pale yellow powder; Yield: 90%, m.p: 228–230 °C; (TLC; n-hexane:EtOAc, 1:2, R_f_ = 0.22); ^1^H NMR (500 MHz, DMSO*-d*_*6*_) δ: 7.61 (1H, s, NH_2_), 7.42–7.39 (3H, m, Ar), 7.29 (1H, d, *J* = 8.2 Hz, Ar), 7.26–7.18 (5H, m, Ar), 4.93 (1H, s, CH), 2.30 (6H, d, *J* = 5 Hz, SMe, Me); ^13^C NMR (125 MHz, DMSO*-d*_*6*_) δ: 165.2, 139.3, 136.6, 136.0, 134.6, 134.0, 133.1, 130.1, 129.9, 129.9, 129.0, 127.5, 117.1, 109.1, 36.6, 21.1, 19.1.

#### Ethyl3-amino-2-(5-(methylthio)-4-nitro-2-phenyl-1-(p-tolyl)-1H-pyrrol-3-yl)-3-oxopropanoate (4ai)

Yellow powder; Yield: 89%, m.p: 200–202 °C; (TLC; n-hexane:EtOAc, 1:2, R_f_ = 0.29); ^1^H NMR (300 MHz, DMSO*-d*_*6*_) δ: 7.40 (1H, s, NH_2_), 7.35–7.05 (10H, m, Ar), 4.55 (1H, s, CH), 4.04–3.87 (2H, m, OCH_2_), 1.13 (3H, t, *J* = 7.1 Hz, Me); ^13^C NMR (75 MHz, DMSO*-d*_*6*_) δ: 168.9, 168.0, 138.9, 138.8, 136.3, 134.4, 131.4, 129.8, 129.3, 129.2, 129.0, 128.6, 127.1, 112.3, 61.3, 49.7, 21.1, 19.6, 14.3.

#### Methyl3-amino-2-(1-(3,4-dimethylphenyl)-5-(methylthio)-4-nitro-2-phenyl-1H-pyrrol-3-yl)-3-oxopropanoate (4aj)

Yellow powder; Yield: 89%, m.p: 201–203 °C; (TLC; n-hexane:EtOAc, 1:2, R_f_ = 0.28); ^1^H NMR (300 MHz, DMSO*-d*_*6*_) δ: 7.44 (1H, s, NH), 7.34–7.00 (9H, m, Ar), 4.57 (1H, s, CH), 3.51 (3H, s, OMe), 2.29 (3H, s, SMe), 2.16 (6H, d, *J* = 16.6 Hz, Me); ^13^C NMR (75 MHz, DMSO*-d*_*6*_) δ: 169.5, 167.9, 138.7, 137.7, 137.4, 136.3, 134.5, 131.3, 130.1, 129.9, 129.3, 129.2, 128.6, 127.1, 126.5, 112.2, 52.6, 49.5, 19.7, 19.6, 19.4; MS (m/z): 453 [M^+^], 407, 379, 349, 331, 304, 273, 244, 208, 189, 163, 128, 105, 77, 44.

#### Ethyl3-amino-2-(5-(methylthio)-4-nitro-1-phenyl-2-(p-tolyl)-1H-pyrrol-3-yl)-3-oxopropanoate (4ak)

Yellow powder; Yield: 92%, m.p: 235–237 °C; (TLC; n-hexane:EtOAc, 1:2, R_f_ = 0.28); ^1^H NMR (500 MHz, DMSO*-d*_*6*_) δ: 7.61 (1H, s, NH_2_), 7.43–7.37 (5H, m, Ar), 7.32 (1H, d, *J* = 7.6 Hz), 7.12 (2H, d, *J* = 7.7 Hz, Ar), 7.08 (2H, d, *J* = 8.0 Hz, Ar), 4.78 (1H, s, CH), 2.31 (3H , s, SMe), 2.25 (3H, s, Me); ^13^C NMR (125 MHz, DMSO*-d*_*6*_) δ: 165.2, 139.2, 137.4, 136.8, 136.7, 131.0, 129.7, 129.6, 129.5, 129.3, 125.5, 117.1, 108.7, 36.7, 21.2, 19.1.

#### Methyl3-amino-2-(1-(3,4-dimethylphenyl)-2-(4-methoxyphenyl)-5-(methylthio)-4-nitro-1H-pyrrol-3-yl)-3-oxopropanoate (4al)

Yellow powder; Yield: 91%, m.p: 218–220 °C; (TLC; n-hexane:EtOAc, 1:2, R_f_ = 0.23); ^1^H NMR (300 MHz, DMSO*-d*_*6*_) δ: 7.43 (1H, s, NH_2_), 7.27–7.03 (6H, m, Ar), 6.88–6.80 (2H, m, Ar), 4.55 (1H, s, CH), 3.70 (3H, s, OMe), 3.54 (3H, s, OMe), 2.29 (3H, s, SMe), 2.18 (6H, d, *J* = 12.8 Hz, Me); ^13^C NMR (75 MHz, DMSO*-d*_*6*_) δ: 169.6, 168.0, 159.7, 138.6, 137.6, 137.4, 136.3, 134.6, 132.7, 130.1, 129.9, 126.8, 126.5, 121.4, 114.0, 112.1, 55.5, 52.6, 49.6, 19.6, 19.4; MS (m/z): 483 [M^+^], 409, 379, 349, 317, 288, 238, 178, 158, 135, 105, 79, 44.

#### 2-cyano-2-(1-(4-methoxyphenyl)-5-(methylthio)-4-nitro-2-(p-tolyl)-1H-pyrrol-3-yl)acetamide (4am)

Pale green powder; Yield: 92%, m.p: 163–165 °C; (TLC; n-hexane:EtOAc, 1:2, R_f_ = 0.22); ^1^H NMR (500 MHz, DMSO*-d*_*6*_) δ: 7.60 (1H, s, NH_2_), 7.39–7.31 (2H, m, Ar), 7.24 (1H, d, *J* = 8.8 Hz), 7.16–7.07 (4H, m, Ar), 6.96–6.90 (2H, m, Ar), 4.75 (1H, s, CH), 3.75 (3H, s, OMe), 2.31 (3H, s, SMe), 2.25 (3H, s, Me); ^13^C NMR (125 MHz, DMSO*-d*_*6*_) δ: 165.2, 159.7, 139.2, 137.6, 136.4, 131.0, 130.4, 130.1, 129.5, 129.4, 125.6, 117.1, 114.4, 108.5, 55.8, 36.7, 21.3, 19.0.

### AChE and BChE inhibition

A previously described modified protocol of Ellman's spectrophotometric assay in clear flat-bottomed, 96-well plates, was used. Briefly, 20 µL AChE 0.18 units/mL, or 20 µL BChE iodide 0.162 units/mL and 20 µL DTNB (final concentration of 301 μM) was added to 200 μl sodium phosphate buffer (0.1 mol/L, pH 7.4) in separate wells of a 96-well microplate and gently mixed. Then, 10 μl of different concentrations of test compounds were incubated for 15 min at 37 °C followed by the addition of acetylthiocholine (ATCh) or butyrylthiocholine (BTCh) (20 μl, final concentration of 452 μM) to produce the yellow anion of 5-thio-2-nitrobenzoic acid^66,67^. The absorbance of each well was measured at 415 nm using a microplate reader. IC_50_ values and inhibition values were calculated with the software GraphPad Prism as the mean of three independent experiments and are expressed as mean ± SEM^68,69^.

### Kinetic study of AChE inhibition

The kinetic study of AChE was carried out at five different concentrations (0–8 µM) of compound **4ad** by Ellman's method. Lineweaver–Burk reciprocal plots (1/v vs. 1/[s]) were constructed at varying concentrations of the substrate acetylthiocholine (0.1–1 mM) to obtain the type of inhibition. The inhibition constant *K*_*i*_ was calculated by the plot of slopes versus the corresponding concentrations of the **4ad**.

### Molecular docking

To understand structural aspects of ligand binding using induced-fit molecular docking (IFD), we used. The SMILE format of **4ad** was converted to a three-dimensional structure within the Maestro software package. The X-ray structures of AChE (PDB code: 4EY7). and BChE (PDB code: 4BDS) were selected as targets for dockings and were prepared with the Protein Preparation Wizard interface of Maestro via removing the ligand and water molecules, adding hydrogen atoms, optimizing their position, and assigning the ionization states of acid and basic residues according to PROPKA prediction at pH 7.0. The molecular docking was performed using IFD mode with the ligands as flexible, the force field was set as OPLS-2005, and all other parameters were set to default. The binding site was used to generate the grid for IFD calculation. The maximum 20 poses with receptor and ligand van der Waals radii of 0.7 and 0.5, respectively considered. Residues within 8 Å of the crystallographic ligands at the active site were refined followed by side-chain optimization. Structures in which primeenergy is more than 30 kcal/mol are eliminated. The re-docking experiment for validation of the used docking protocol was done and recorded the RMSD value of 0.79, indicating the docking experiment is reliable ^70,71^.

### Molecular dynamic simulations

Molecular simulations of this study were performed using the Desmond v5.3 using the Maestro interface (from Schrödinger 2018‐4 suite). The appropriate pose for the MD simulation procedure of the compound was achieved by the IFD method. To build the system for MD simulation, the protein–ligand complex was solvated with SPC explicit water molecules and placed in the center of an orthorhombic box of appropriate size in the periodic boundary condition. Sufficient counter‐ions and a 0.15 M solution of NaCl were also utilized to neutralize the system and to simulate the real cellular ionic concentrations, respectively. The MD protocol involved minimization, pre-production, and finally production MD simulation steps. In the minimization procedure, the entire system was allowed to relax for 2500 steps by the steepest descent approach. Then the temperature of the system was raised from 0 to 300 K with a small force constant on the enzyme to restrict any drastic changes. MD simulations were performed via NPT (constant number of atoms, constant pressure i.e. 1.01325 bar, and constant temperature i.e. 300 K) ensemble. The optimum system was finally subjected to produce MD simulations for 30 ns for the protein–ligand complex. During the simulation, every 1000 ps of the actual frame was stored. The dynamic behavior and structural changes of the systems were analyzed by the calculation of the RMSD and RMSF. Subsequently, the representative frames of the simulation were extracted based on the clustering method from the equilibrated trajectory system for investigating of ligand–protein complex interaction.

The geometric criteria for H-bond interaction are the distance of 2.5 Å between the donor and acceptor atoms, a donor angle of ≥ 120° between the donor-hydrogen-acceptor atoms, and an acceptor angle of ≥ 90°. The π-cation happened between aromatic and charged groups within a 4.5 Å distance. The π–π and other non-specific hydrophobic side chains were seen within 3.6 Å of a ligand's aromatic or aliphatic carbons. Ionic interactions or polar interactions happened between two oppositely charged atoms within 3.7 Å of each other which did not involve in the H-bond. Water Bridge is hydrogen-bonded protein–ligand interaction mediated by water with a distance of 2.8 Å between the donor and acceptor atoms, a donor angle of ≥ 110° between the donor-hydrogen with acceptor atoms, and an acceptor angle of ≥ 90° between the hydrogen-acceptor- atoms.

## Supplementary Information

Supplementary Information.

## Data Availability

The datasets generated and/or analyzed during the current study are available in the Worldwide Protein Data Bank (wwPDB) repository. (http://www.rcsb.org).
