# Supplementary material for: Design, synthesis, in silico and biological evaluations of novel polysubstituted pyrroles as selective acetylcholinesterase inhibitors against Alzheimer’s disease
Source: Sci Rep. 2022 Sep 8;12:15236. doi: 10.1038/s41598-022-18224-6 (PMC9454393; doi:10.1038/s41598-022-18224-6)
Supplement: Supplementary file 1 — Supplementary Information. [file 41598_2022_18224_MOESM1_ESM.pdf]

Supplementary data for the following manuscript:

Highly efficient, one-pot, multi-component synthesis of novel polysubstituted pyrroles as selective acetylcholinesterase inhibitors; An *in vitro*, molecular docking, and molecular dynamics simulation studies

Hormoz Pourtaher,[a] Alireza Hasaninejad[a]* Armin Iraji[b], Aida Iraji[c,d]*

[a]*Department of Chemistry, Faculty of Sciences, Persian Gulf University, Bushehr 75169, Iran.*

[b] *Tri-Institutional Center for Translational Research in Neuroimaging and Data Science (TReNDS), Georgia State University, Georgia Institute of Technology, and Emory University, Atlanta, GA 30303, USA*

[c] *Stem Cells Technology Research Center, Shiraz University of Medical Sciences, Shiraz, Iran*

[d] *Central Research Laboratory, Shiraz University of Medical Sciences, Shiraz, Iran*

*Corresponding Authors:

Alireza Hasaninejada

✉e-mail: alirezahasaninejad@gmail.com; a_hasaninejad@yahoo.com

Tel: (+)+98(771)4541494

Fax: +98(771)4541494;

Aida Iraji

✉e-mail: iraji@sums.ac.ir; aida.iraji@gmail.com

Address: Stem Cells Technology Research Center, Shiraz University of Medical Sciences, Shiraz, Iran
Central Research Laboratory, Shiraz University of Medical Sciences, Shiraz, Iran

4a-H

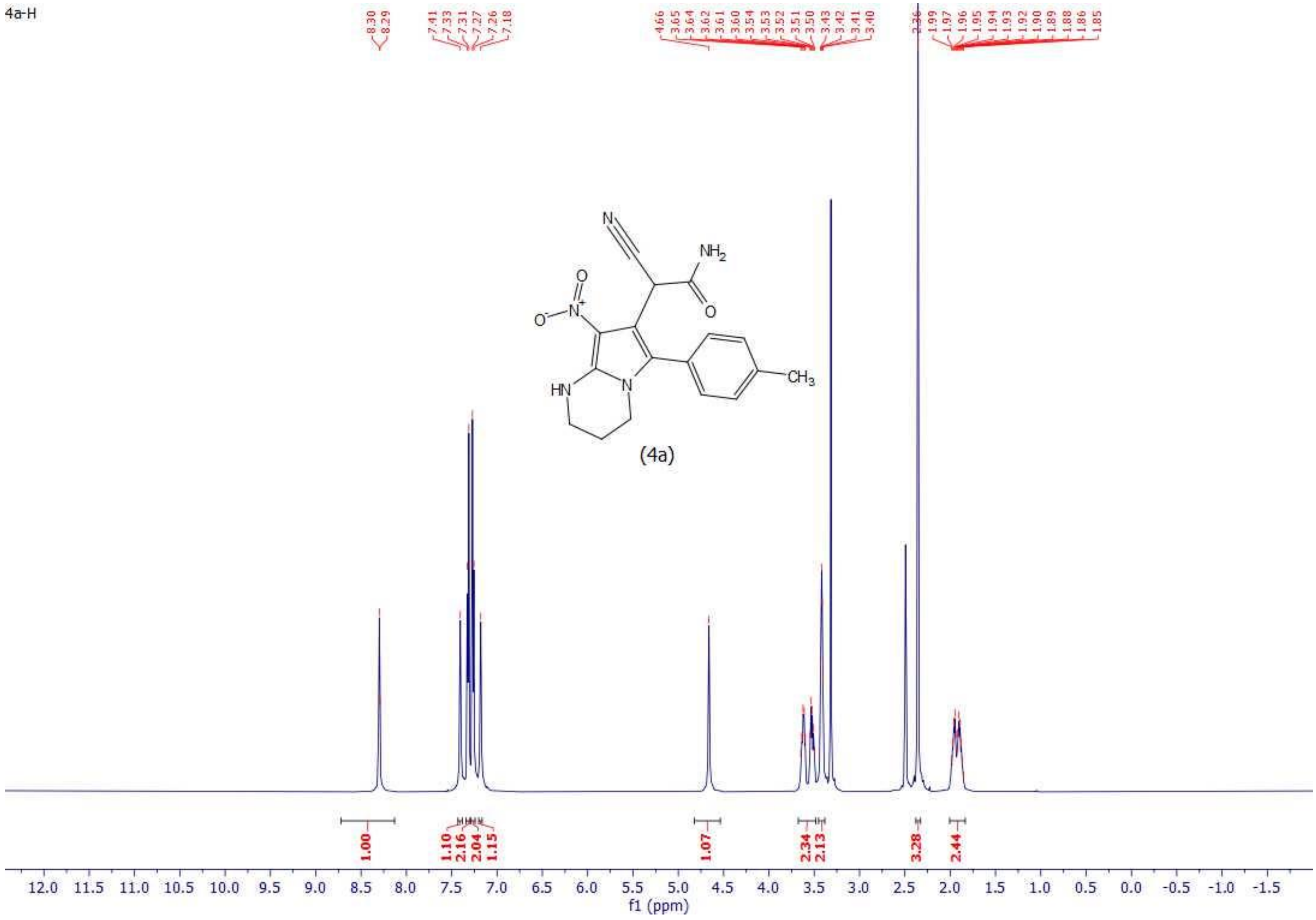
8.30
8.29
7.41
7.33
7.31
7.27
7.26
7.18
4.66
3.65
3.64
3.62
3.61
3.60
3.54
3.53
3.52
3.51
3.50
3.43
3.42
3.41
3.40
2.36
1.99
1.97
1.96
1.95
1.94
1.93
1.92
1.90
1.89
1.88
1.86
1.85
N
NH2
O
O-
N+
O
HN
N
CH3
(4a)
1.00
1.10
2.16
2.04
1.15
1.07
2.34
2.13
3.28
2.44
12.0 11.5 11.0 10.5 10.0 9.5 9.0 8.5 8.0 7.5 7.0 6.5 6.0 5.5 5.0 4.5 4.0 3.5 3.0 2.5 2.0 1.5 1.0 0.5 0.0 -0.5 -1.0 -1.5
f1 (ppm)

4a-H

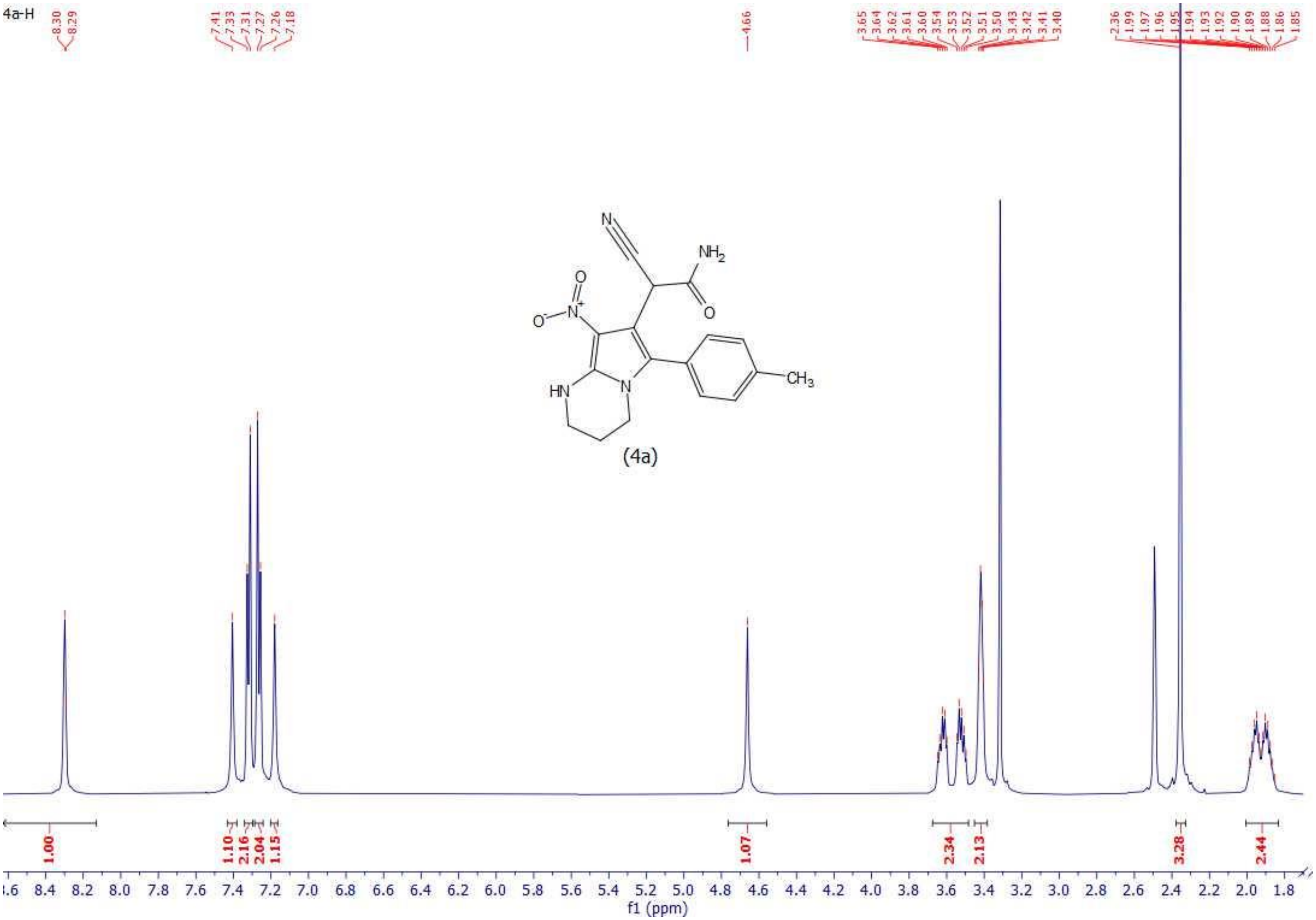
8.30
8.29
7.41
7.33
7.31
7.27
7.26
7.18
4.66
3.65
3.64
3.62
3.61
3.60
3.54
3.53
3.52
3.51
3.50
3.43
3.42
3.41
3.40
2.36
1.99
1.97
1.96
1.95
1.94
1.93
1.92
1.90
1.89
1.88
1.86
1.85
(4a)
1.00
1.10
2.16
2.04
1.15
1.07
2.34
2.13
3.28
2.44
f1 (ppm)

4a-C

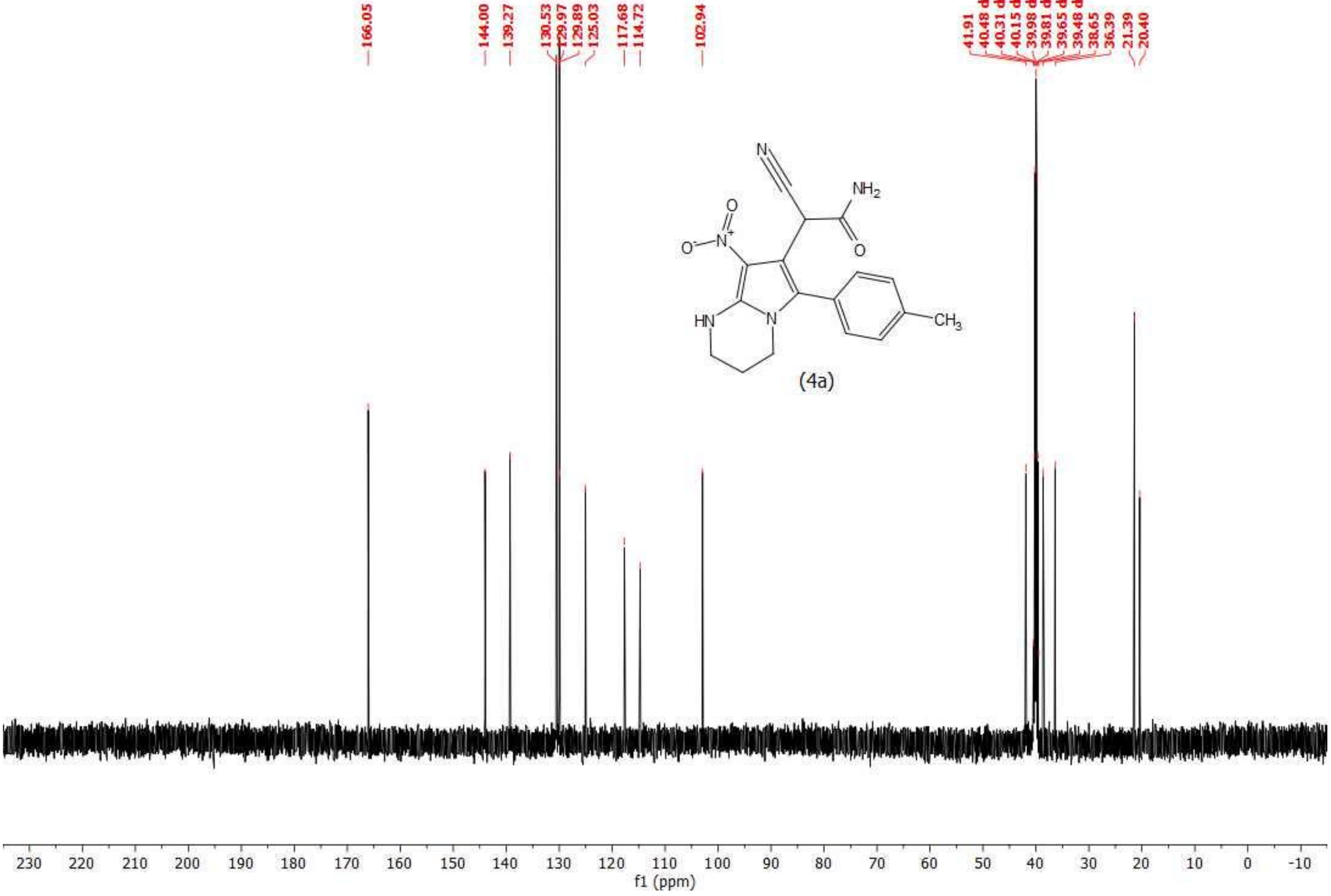
166.05
144.00
139.27
130.53
129.97
129.89
125.03
117.68
114.72
102.94
41.91
40.48 dmso
40.31 dmso
40.15 dmso
39.98 dmso
39.81 dmso
39.65 dmso
39.48 dmso
38.65
36.39
21.39
20.40
N
NH2
O
O-
N+
O
HN
N
CH3
(4a)
230
220
210
200
190
180
170
160
150
140
130
120
110
100
90
80
70
60
50
40
30
20
10
0
-10
f1 (ppm)

4a-C

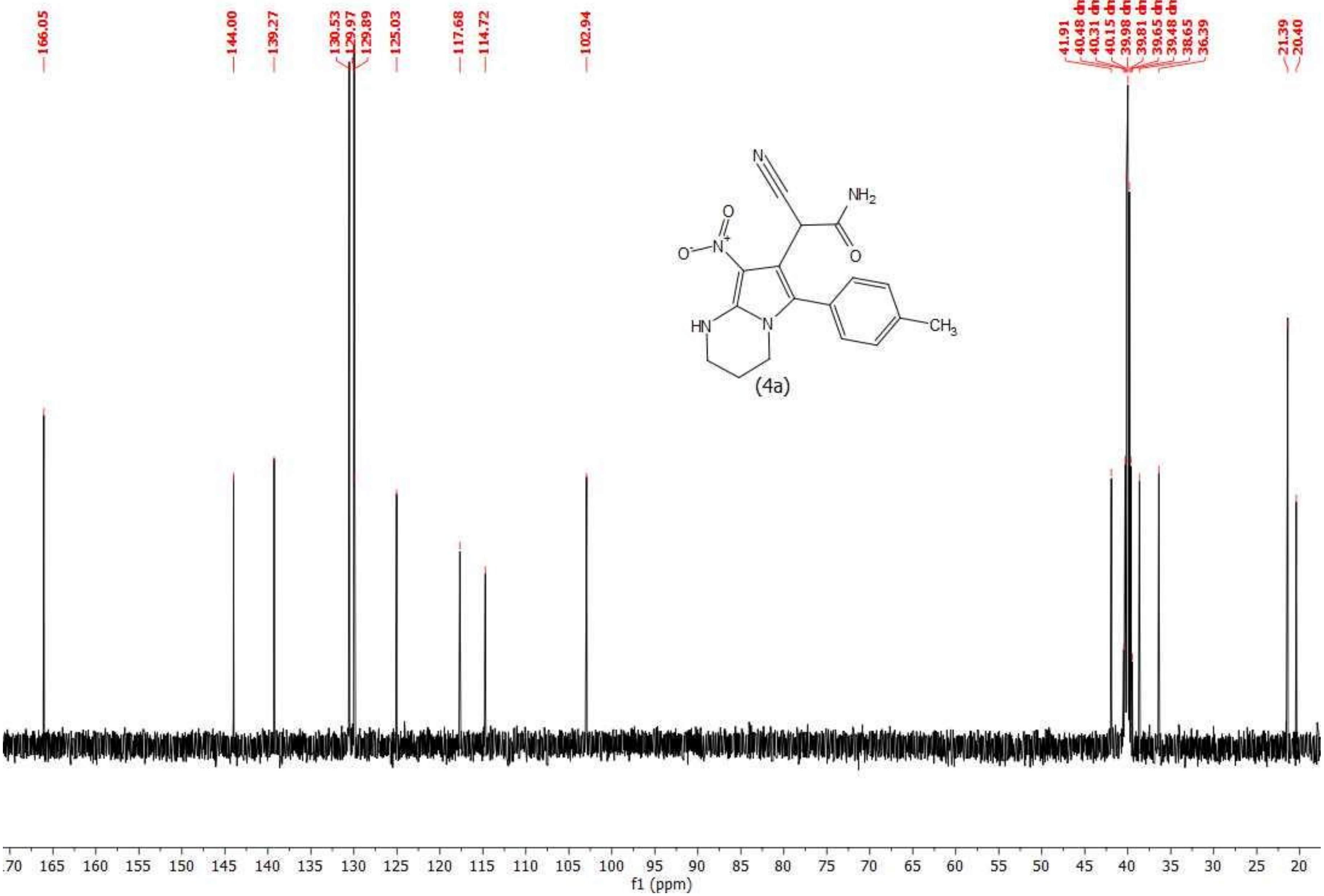

166.05
144.00
139.27
130.53
129.97
129.89
125.03
117.68
114.72
102.94
41.91
40.48 dmso
40.31 dmso
40.15 dmso
39.98 dmso
39.81 dmso
39.65 dmso
39.48 dmso
38.65
36.39
21.39
20.40
N
NH2
O
O
N+
O-
HN
N
CH3
(4a)
70 165 160 155 150 145 140 135 130 125 120 115 110 105 100 95 90 85 80 75 70 65 60 55 50 45 40 35 30 25 20
f1 (ppm)

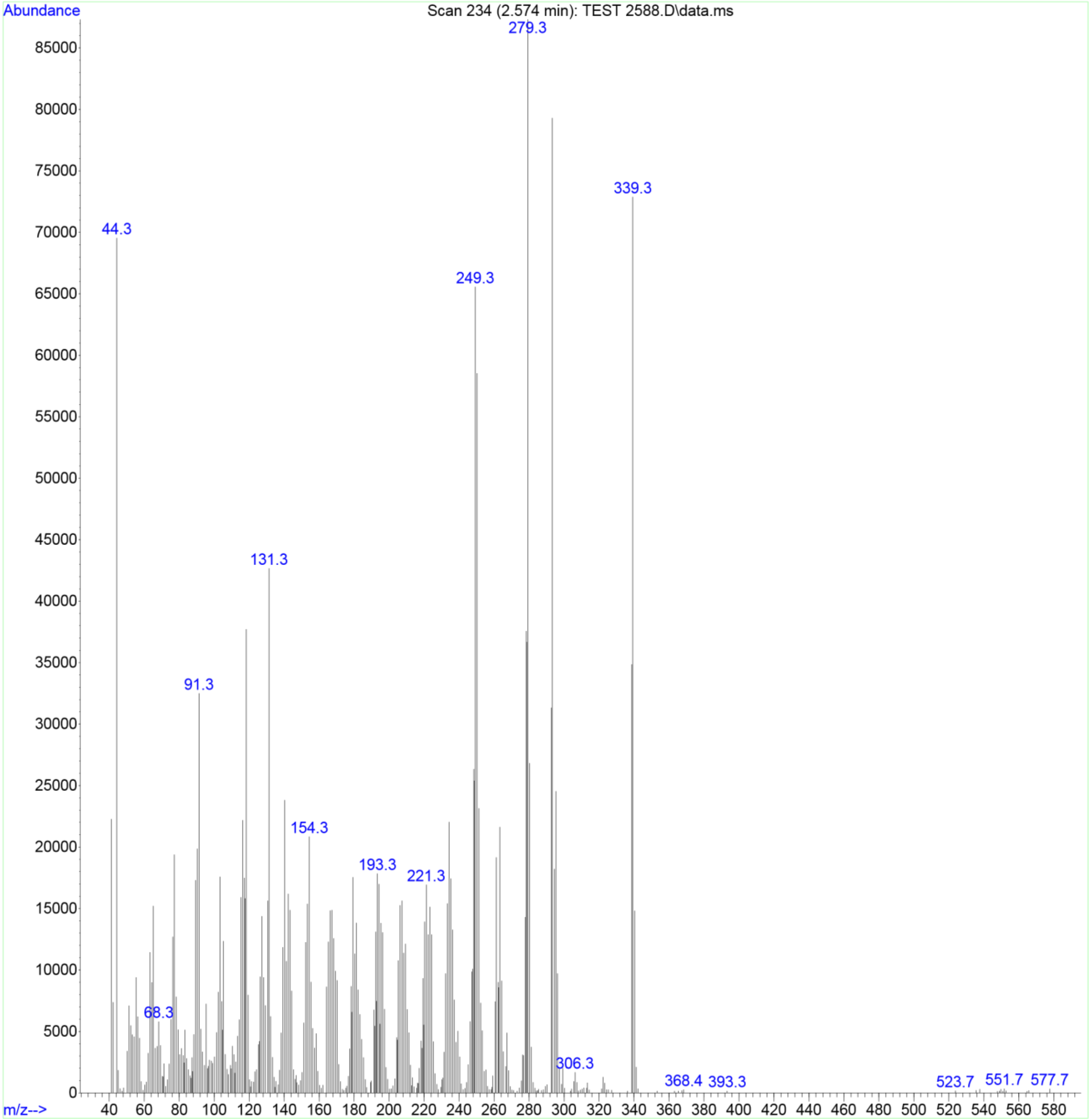

$C_{17}H_{17}N5O_3$

(339/35)

(4a)

4b-H

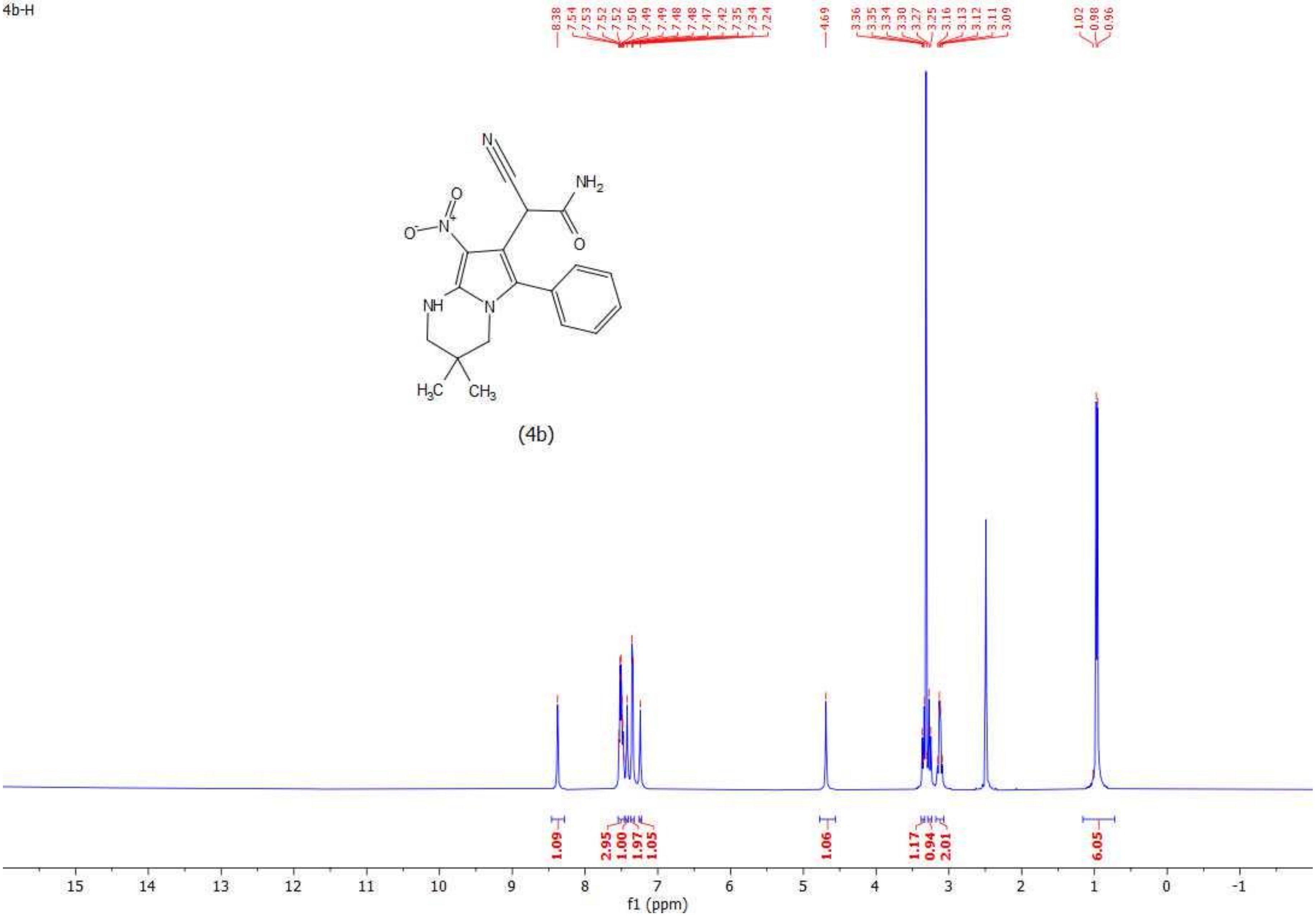
8.38 7.54 7.53 7.52 7.52 7.50 7.49 7.49 7.48 7.48 7.47 7.42 7.35 7.34 7.24
4.69
3.36 3.35 3.34 3.30 3.27 3.25 3.16 3.13 3.12 3.11 3.09
1.02 0.98 0.96
N
NH2
O
O-
N+
NH
N
H3C
CH3
1.09
2.95 1.00 1.97 1.05
1.06
1.17 0.94 2.01
6.05
15 14 13 12 11 10 9 8 7 6 5 4 3 2 1 0 -1
f1 (ppm)

(4b)

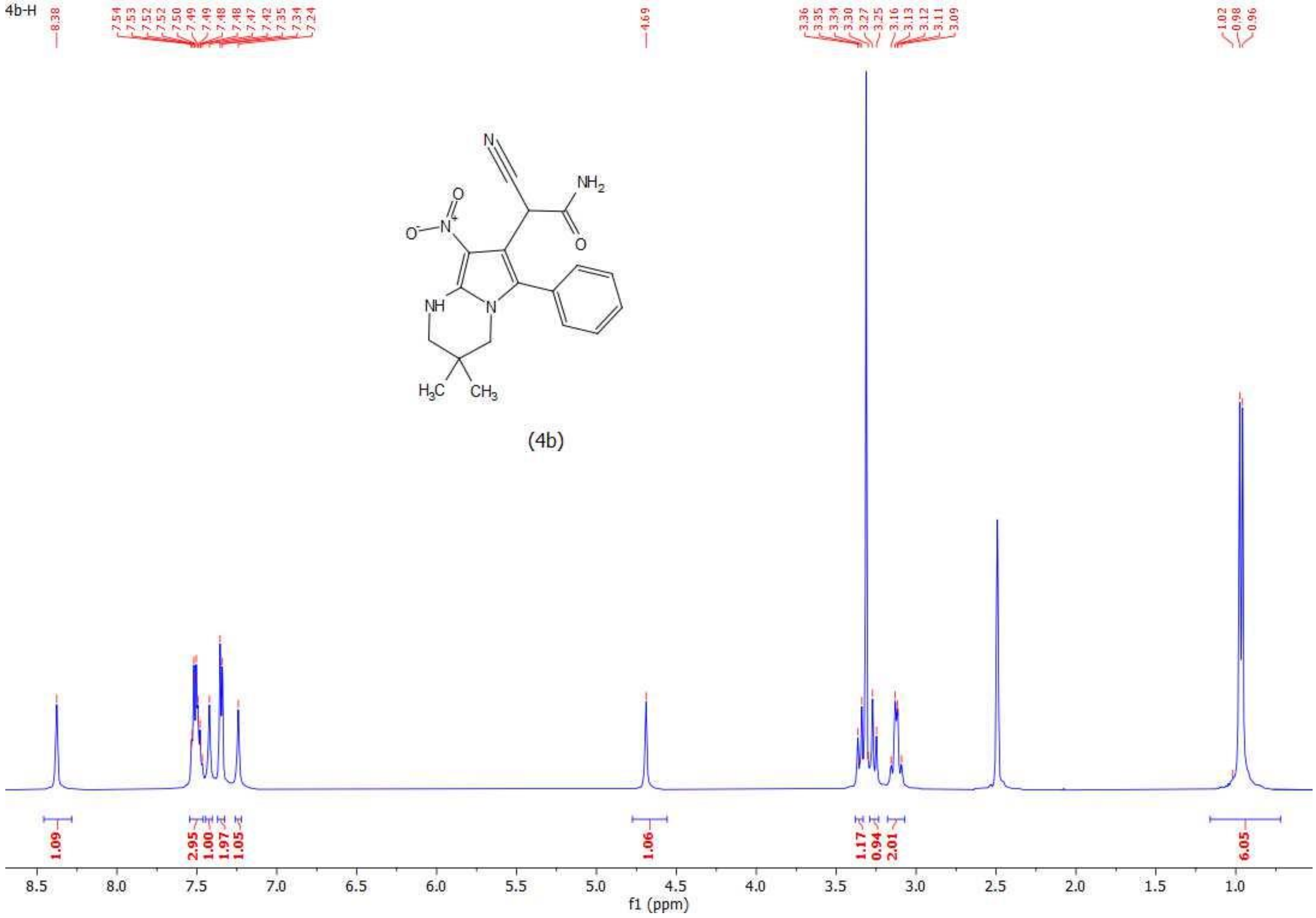
4b-H
8.38
7.54
7.53
7.52
7.52
7.50
7.49
7.49
7.48
7.48
7.47
7.42
7.35
7.34
7.24
4.69
3.36
3.35
3.34
3.30
3.27
3.25
3.16
3.13
3.12
3.11
3.09
1.02
0.98
0.96
NH2
O
O-
N+
NH
N
H3C
CH3
(4b)
1.09
2.95
1.00
1.97
1.05
1.06
1.17
0.94
2.01
6.05
8.5
8.0
7.5
7.0
6.5
6.0
5.5
5.0
4.5
4.0
3.5
3.0
2.5
2.0
1.5
1.0
f1 (ppm)

4b-C

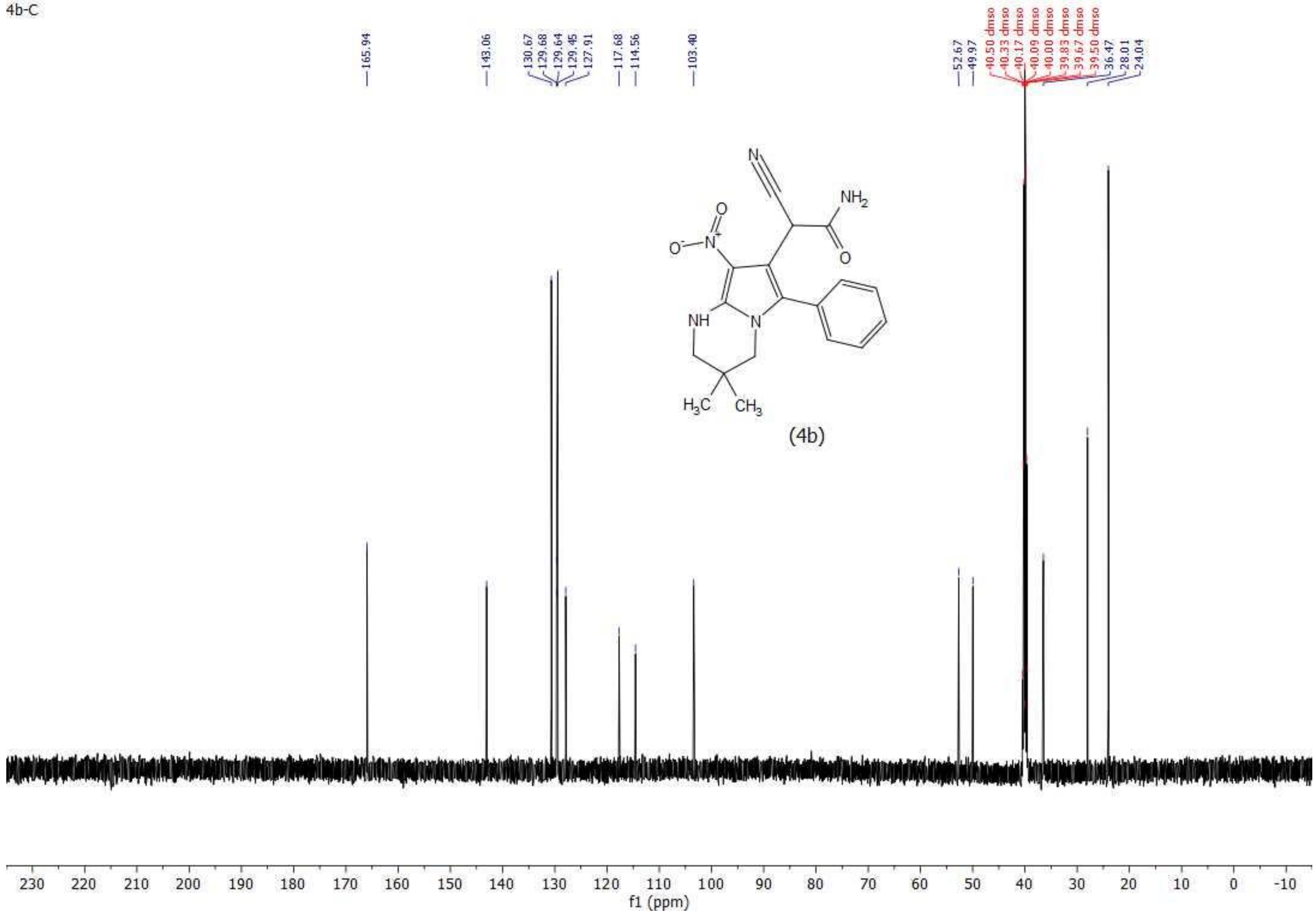
165.94
143.06
130.67
129.68
129.64
129.45
127.91
117.68
114.56
103.40
52.67
49.97
40.50 dmso
40.33 dmso
40.17 dmso
40.09 dmso
40.00 dmso
39.83 dmso
39.67 dmso
39.50 dmso
36.47
28.01
24.04
NH
N
NH_2
O
N^+
O^-
N
H_3C
CH_3
(4b)
230 220 210 200 190 180 170 160 150 140 130 120 110 100 90 80 70 60 50 40 30 20 10 0 -10
f1 (ppm)

4b-C

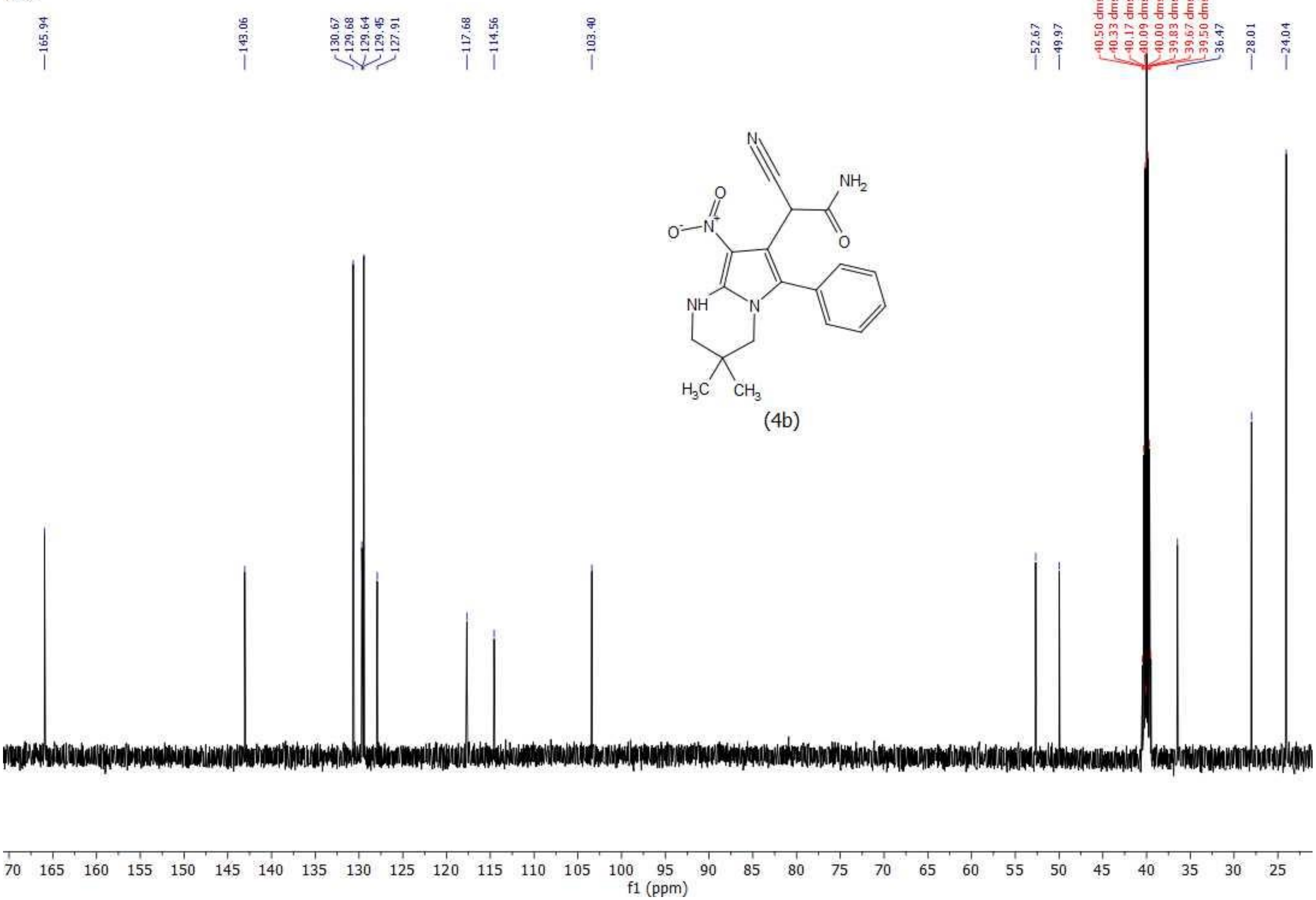
165.94
143.06
130.67
129.68
129.64
129.45
127.91
117.68
114.56
103.40
52.67
49.97
40.50 dmso
40.33 dmso
40.17 dmso
40.09 dmso
40.00 dmso
39.83 dmso
39.67 dmso
39.50 dmso
36.47
28.01
24.04
N
NH2
O
O-
N+
NH
N
H3C
CH3
(4b)
70 165 160 155 150 145 140 135 130 125 120 115 110 105 100 95 90 85 80 75 70 65 60 55 50 45 40 35 30 25
f1 (ppm)

4c-H

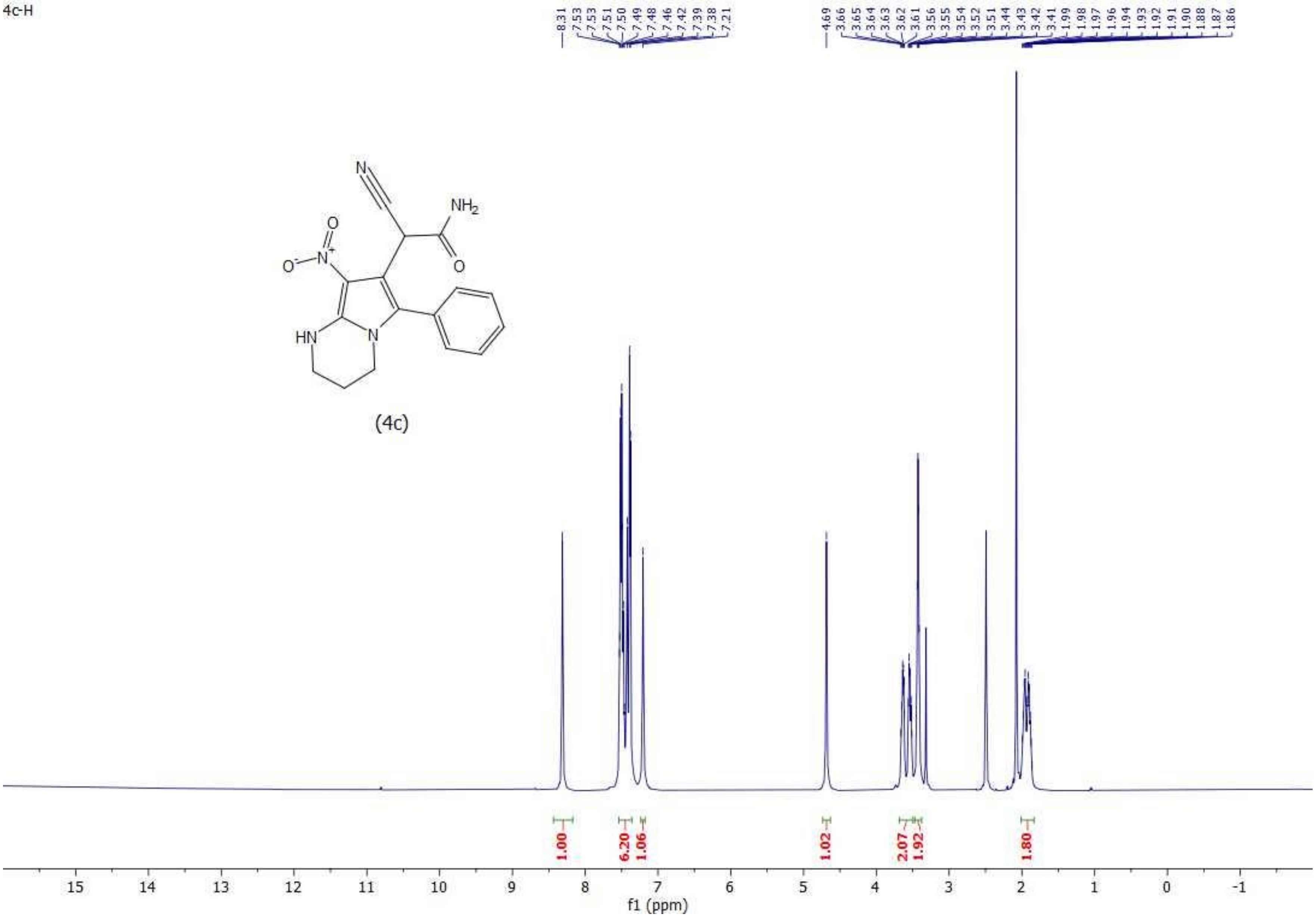
N
NH2
O
O-
N+
O
HN
N
(4c)
8.31
7.53
7.53
7.51
7.50
7.49
7.48
7.46
7.42
7.39
7.38
7.21
4.69
3.66
3.65
3.64
3.63
3.62
3.61
3.56
3.55
3.54
3.52
3.51
3.44
3.43
3.42
3.41
1.99
1.98
1.97
1.96
1.94
1.93
1.92
1.91
1.90
1.88
1.87
1.86
1.00
6.20
1.06
1.02
2.07
1.92
1.80
15 14 13 12 11 10 9 8 7 6 5 4 3 2 1 0 -1
f1 (ppm)

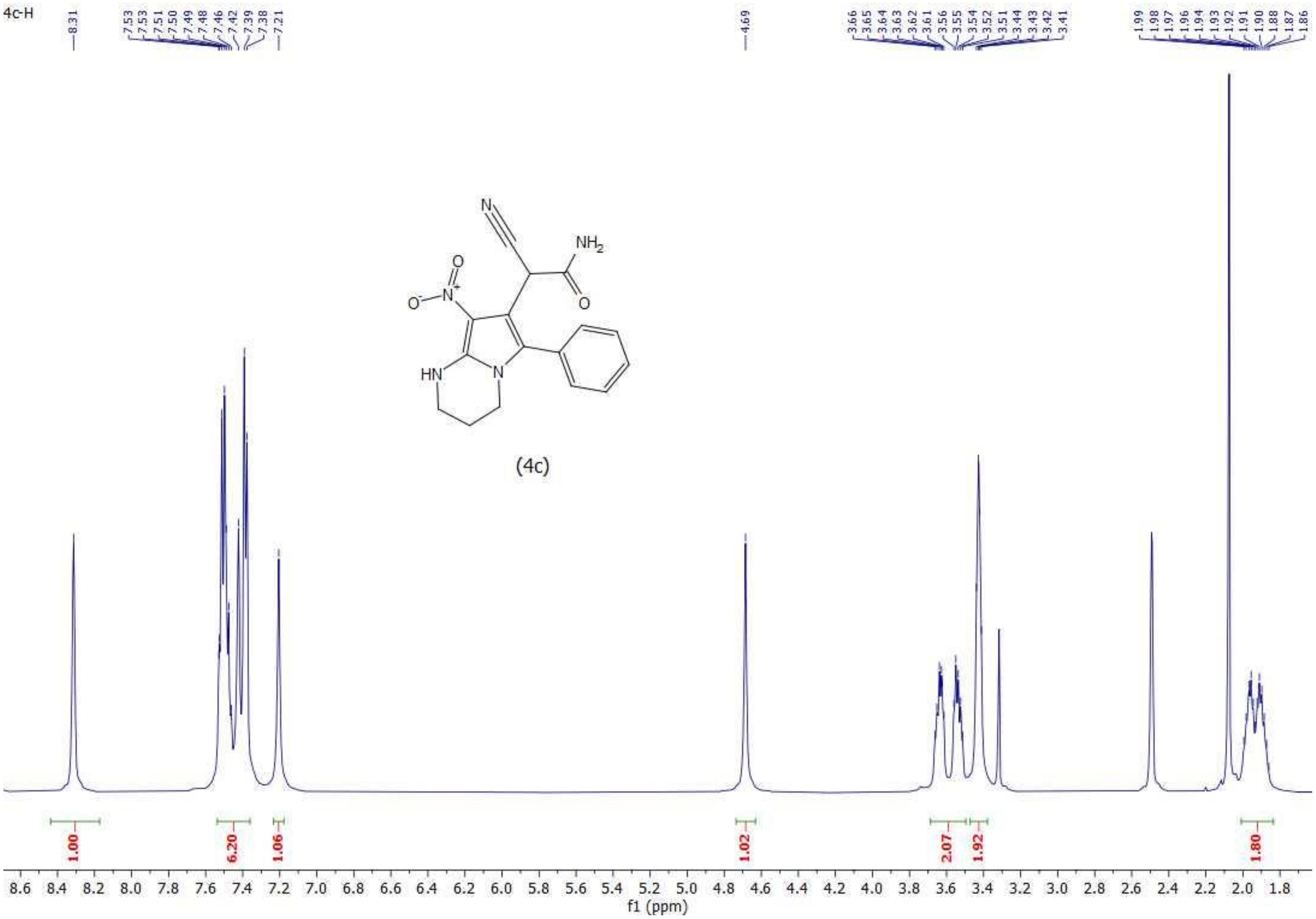
4c-H
8.31
7.53
7.53
7.51
7.50
7.49
7.48
7.46
7.42
7.39
7.38
7.21
4.69
3.66
3.65
3.64
3.63
3.62
3.61
3.56
3.55
3.54
3.52
3.51
3.44
3.43
3.42
3.41
1.99
1.98
1.97
1.96
1.94
1.93
1.92
1.91
1.90
1.88
1.87
1.86
N
NH2
O
O-
N+
HN
N
(4c)
1.00
6.20
1.06
1.02
2.07
1.92
1.80
8.6 8.4 8.2 8.0 7.8 7.6 7.4 7.2 7.0 6.8 6.6 6.4 6.2 6.0 5.8 5.6 5.4 5.2 5.0 4.8 4.6 4.4 4.2 4.0 3.8 3.6 3.4 3.2 3.0 2.8 2.6 2.4 2.2 2.0 1.8
f1 (ppm)

4c-C

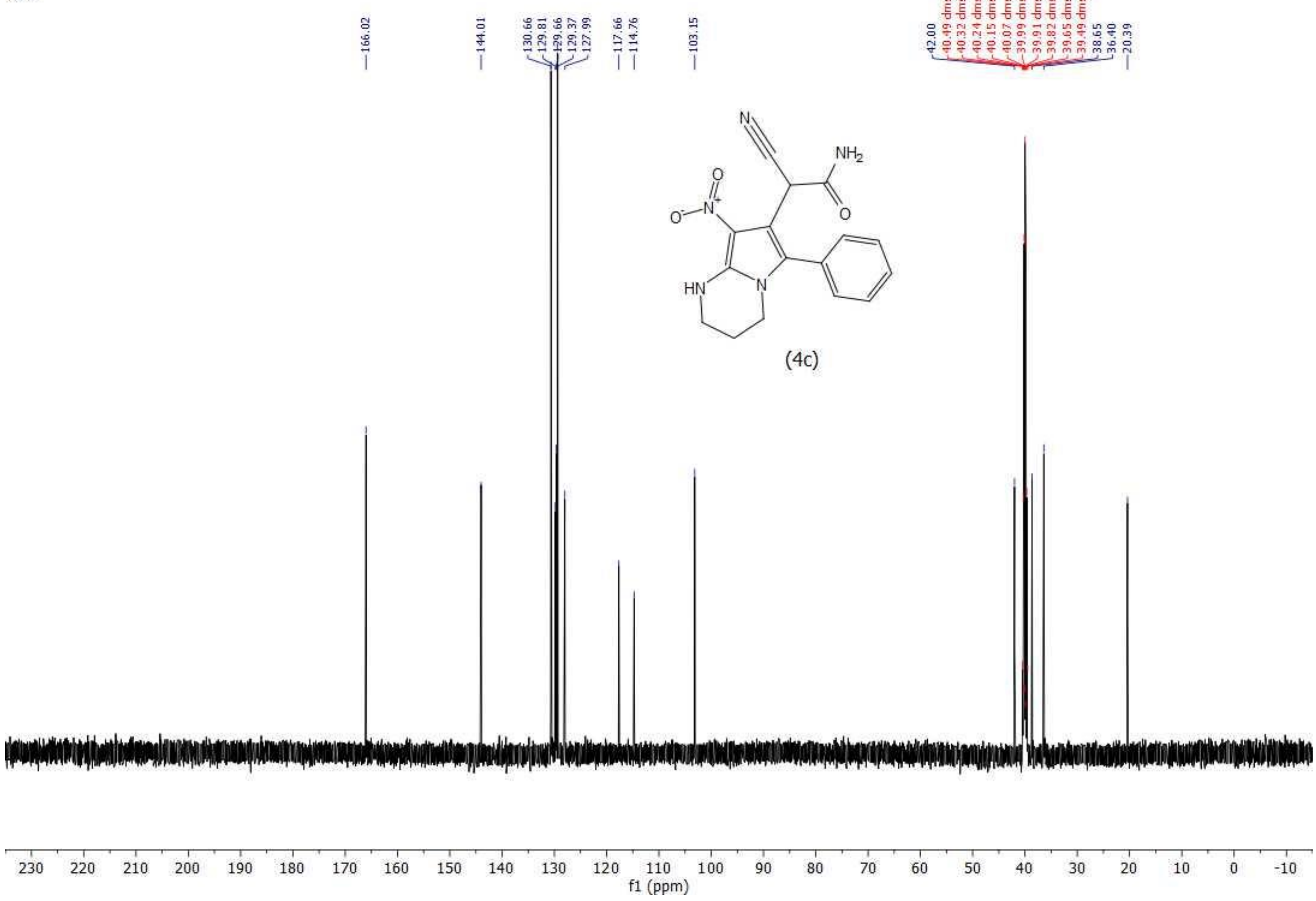
166.02
144.01
130.66
129.81
129.66
129.37
127.99
117.66
114.76
103.15
42.00
40.49 dmso
40.32 dmso
40.24 dmso
40.15 dmso
40.07 dmso
39.99 dmso
39.91 dmso
39.82 dmso
39.65 dmso
39.49 dmso
38.65
36.40
20.39
(4c)
f1 (ppm)

4c-C

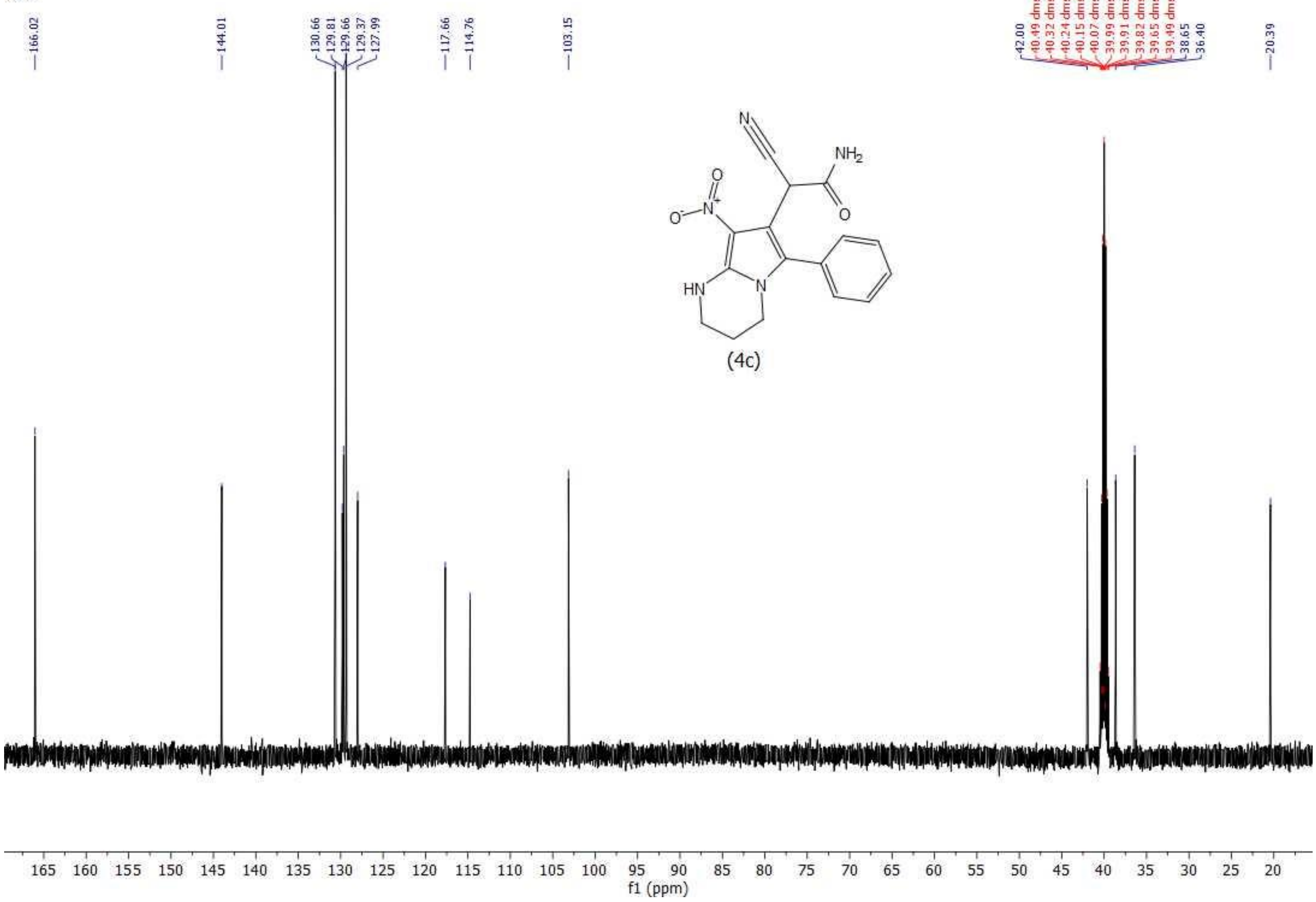
166.02
144.01
130.66
129.81
129.66
129.37
127.99
117.66
114.76
103.15
42.00
40.49 dmso
40.32 dmso
40.24 dmso
40.15 dmso
40.07 dmso
39.99 dmso
39.91 dmso
39.82 dmso
39.65 dmso
39.49 dmso
38.65
36.40
20.39
N
NH2
O
O
N+
O-
HN
N
(4c)
165 160 155 150 145 140 135 130 125 120 115 110 105 100 95 90 85 80 75 70 65 60 55 50 45 40 35 30 25 20
f1 (ppm)

4d-H

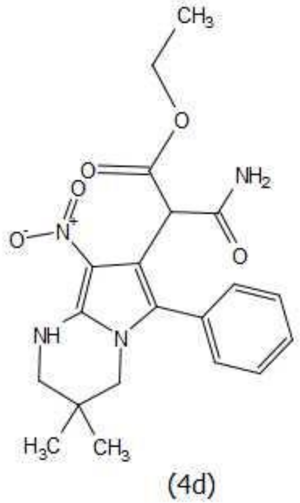

(4d)

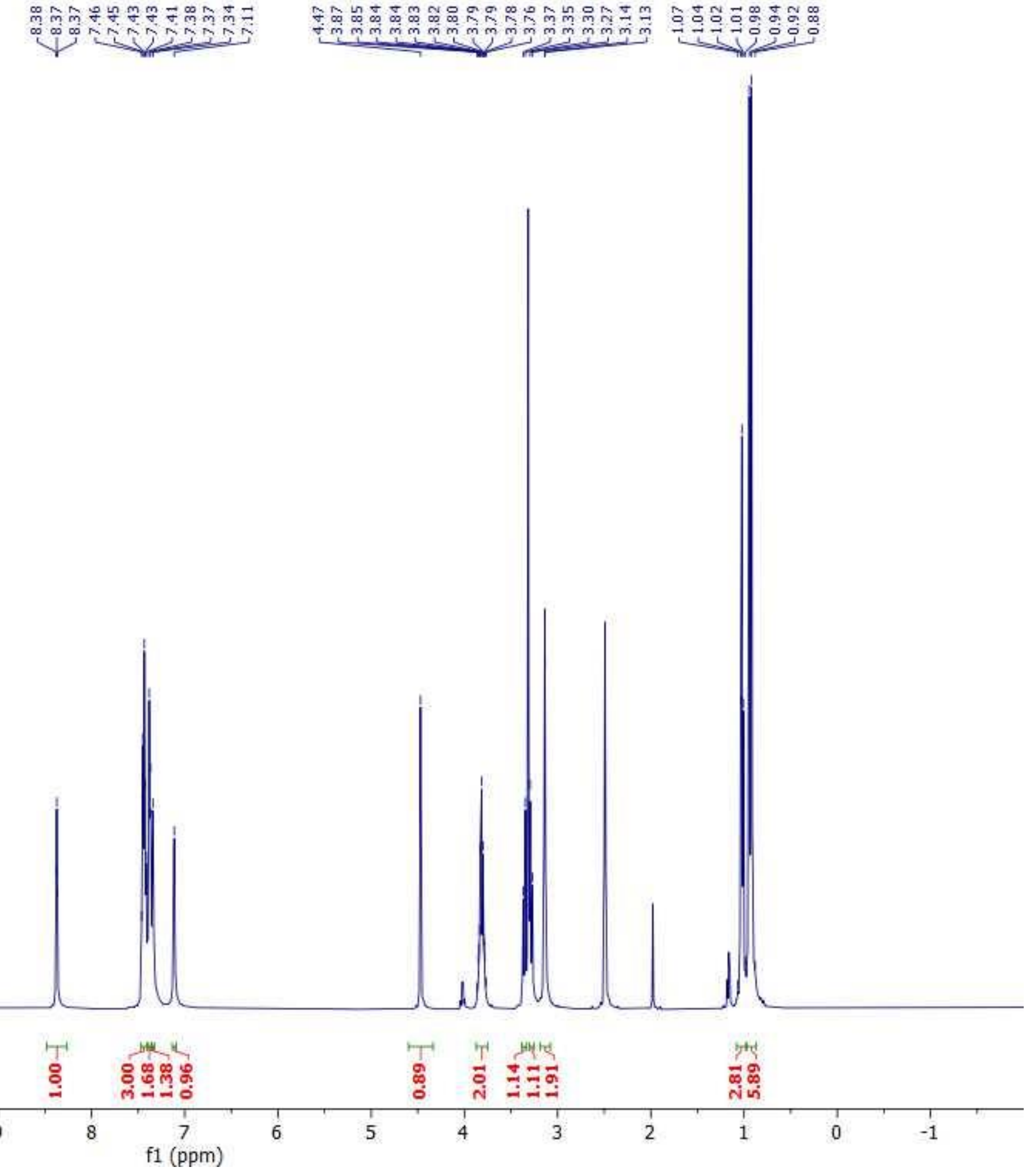

8.38
8.37
8.37
7.46
7.45
7.43
7.43
7.41
7.38
7.37
7.34
7.11
4.47
3.87
3.85
3.84
3.84
3.83
3.82
3.80
3.79
3.79
3.78
3.76
3.37
3.35
3.30
3.27
3.14
3.13
1.07
1.04
1.02
1.01
0.98
0.94
0.92
0.88
1.00
3.00
1.68
1.38
0.96
0.89
2.01
1.14
1.11
1.91
2.81
5.89
f1 (ppm)

4d-H

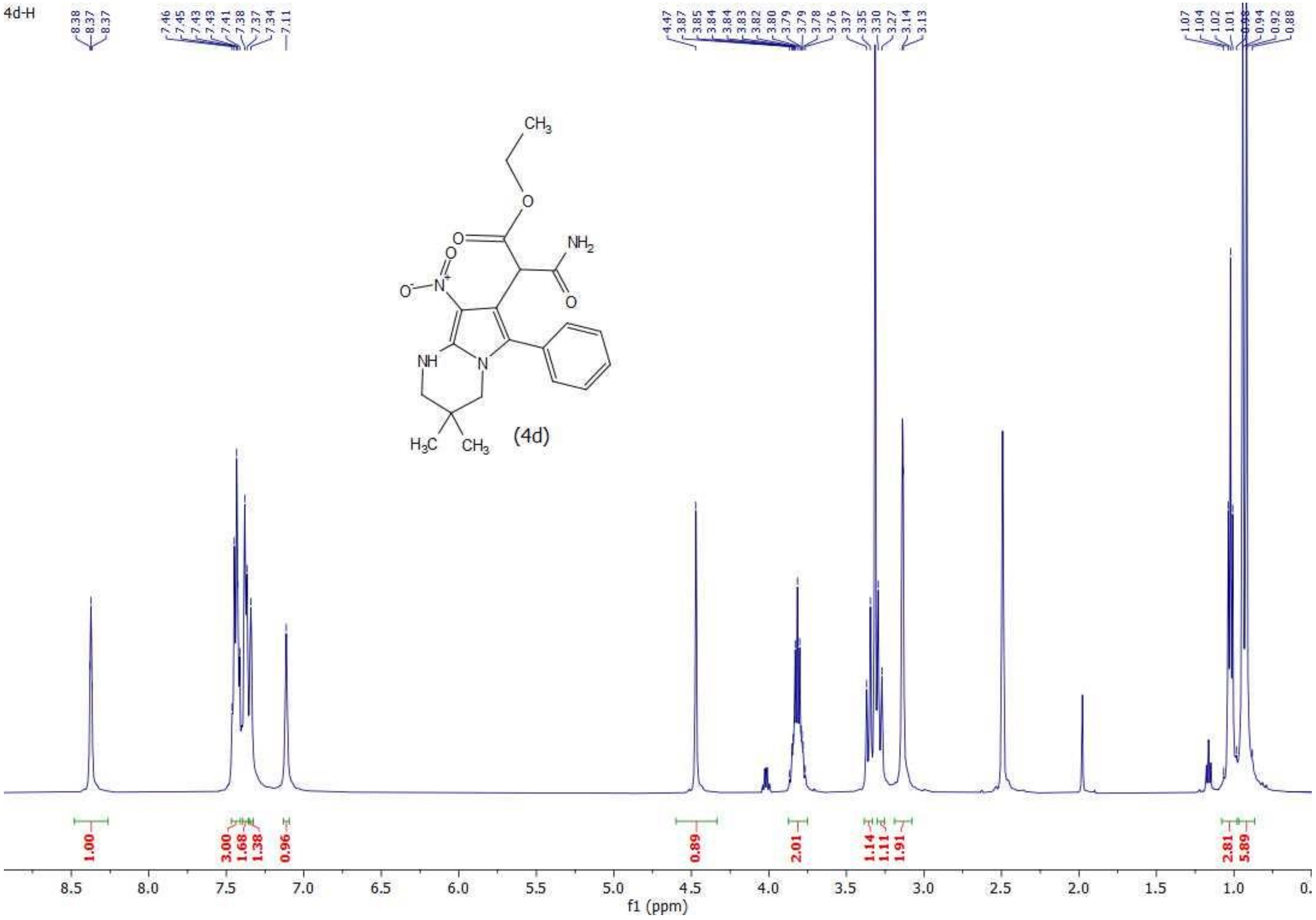
8.38
8.37
8.37
7.46
7.45
7.43
7.43
7.41
7.38
7.37
7.34
7.11
4.47
3.87
3.85
3.84
3.84
3.83
3.82
3.80
3.79
3.79
3.78
3.76
3.37
3.35
3.30
3.27
3.14
3.13
1.07
1.04
1.02
1.01
0.98
0.94
0.92
0.88
CH3
O
O
NH2
O
O-
N+
O
NH
N
H3C
CH3
(4d)
1.00
3.00
1.68
1.38
0.96
0.89
2.01
1.14
1.11
1.91
2.81
5.89
f1 (ppm)

4d-C

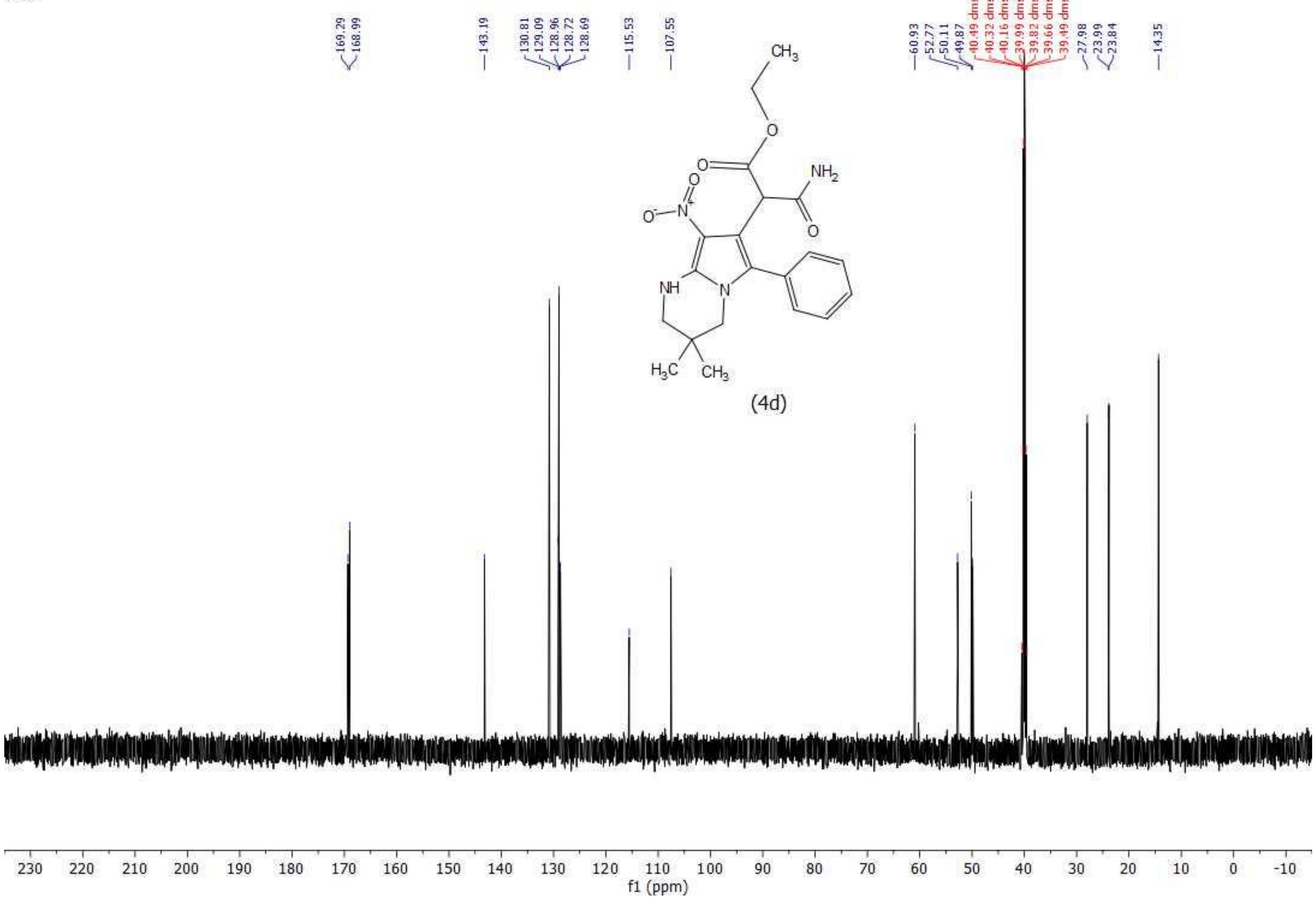
169.29
168.99
143.19
130.81
129.09
128.96
128.72
128.69
115.53
107.55
60.93
52.77
50.11
49.87
40.49 dmso
40.32 dmso
40.16 dmso
39.99 dmso
39.82 dmso
39.66 dmso
39.49 dmso
27.98
23.99
23.84
14.35
(4d)
230 220 210 200 190 180 170 160 150 140 130 120 110 100 90 80 70 60 50 40 30 20 10 0 -10
f1 (ppm)

4d-C

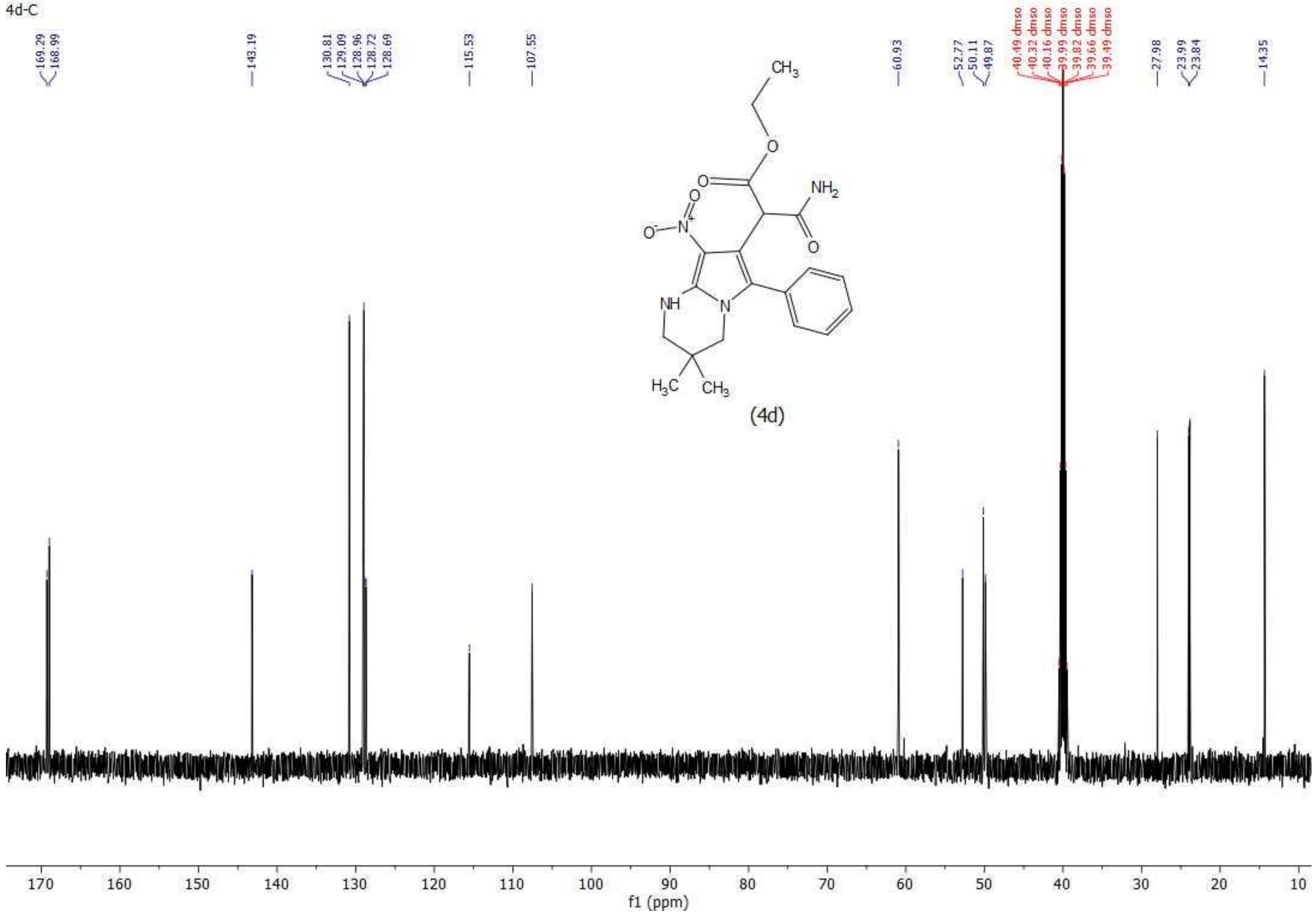
169.29
168.99
143.19
130.81
129.09
128.96
128.72
128.69
115.53
107.55
60.93
52.77
50.11
49.87
40.49 dmso
40.32 dmso
40.16 dmso
39.99 dmso
39.82 dmso
39.66 dmso
39.49 dmso
27.98
23.99
23.84
14.35
CH_3
O
O
NH_2
O
O^-
N^+
O
NH
N
H_3C
CH_3
(4d)
170 160 150 140 130 120 110 100 90 80 70 60 50 40 30 20 10
f1 (ppm)

4e-H

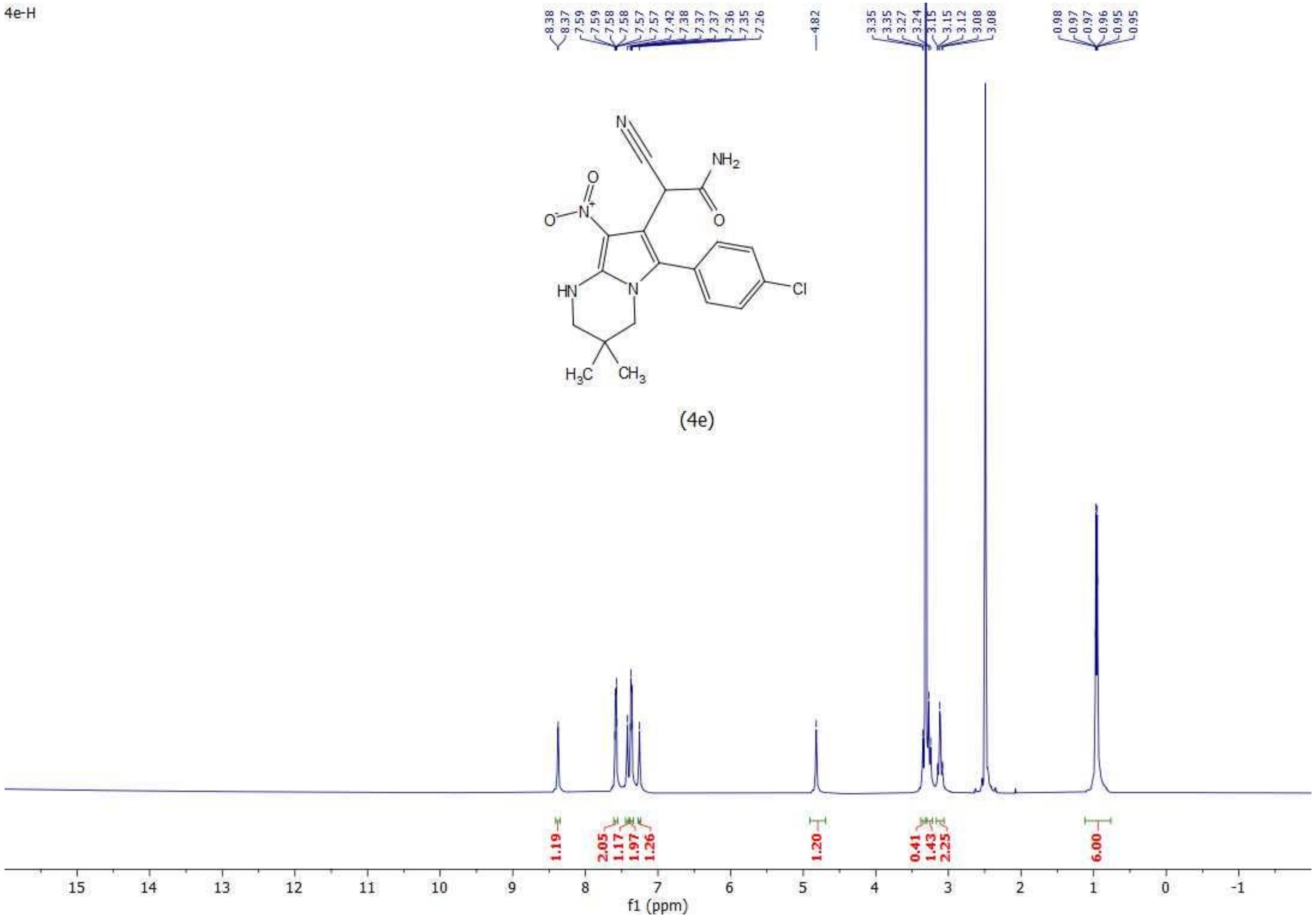
8.38
8.37
7.59
7.59
7.58
7.58
7.57
7.57
7.42
7.38
7.37
7.37
7.36
7.35
7.26
4.82
3.35
3.35
3.27
3.24
3.15
3.15
3.12
3.08
3.08
0.98
0.97
0.97
0.96
0.95
0.95
N
NH_2
O
N^+
O^-
O
HN
N
Cl
H_3C
CH_3
1.19
2.05
1.17
1.97
1.26
1.20
0.41
1.43
2.25
6.00
15
14
13
12
11
10
9
8
7
6
5
4
3
2
1
0
-1
f1 (ppm)

(4e)

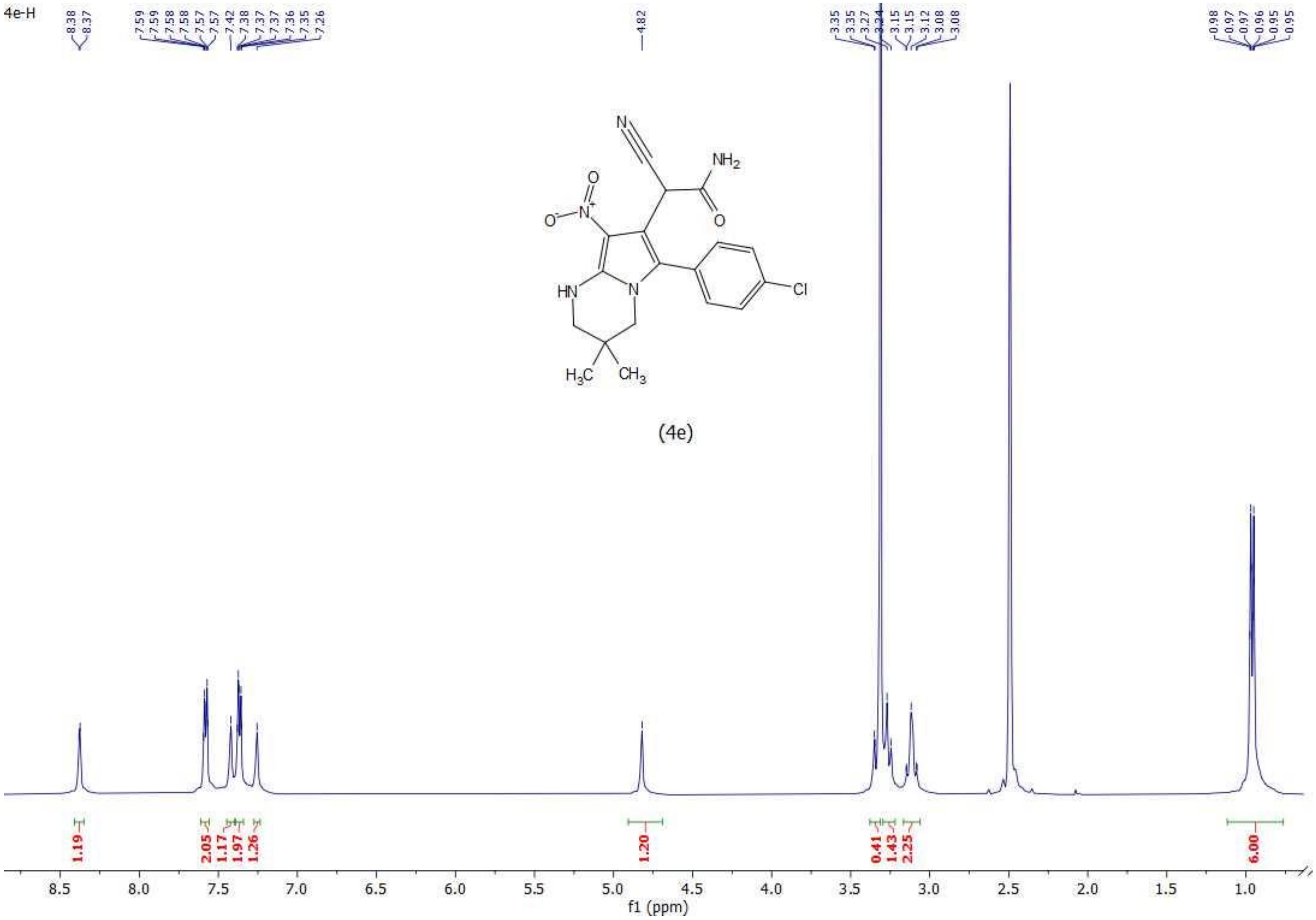
4e-H
8.38
8.37
7.59
7.59
7.58
7.58
7.57
7.57
7.42
7.38
7.37
7.37
7.36
7.35
7.26
4.82
3.35
3.35
3.27
3.24
3.15
3.15
3.12
3.08
3.08
0.98
0.97
0.97
0.96
0.95
0.95
(4e)
1.19
2.05
1.17
1.97
1.26
1.20
0.41
1.43
2.25
6.00
f1 (ppm)

4e-C

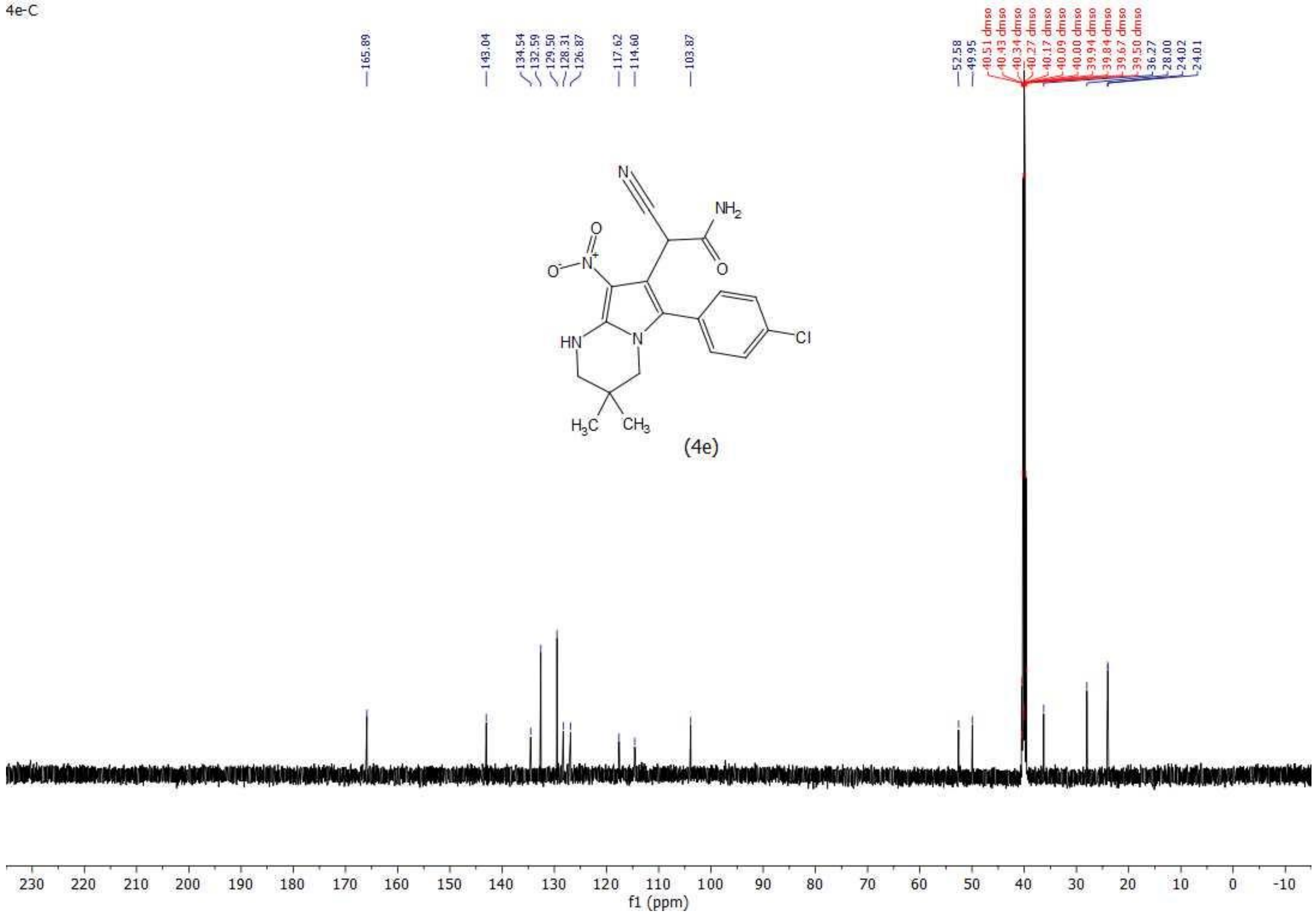
165.89
143.04
134.54
132.59
129.50
128.31
126.87
117.62
114.60
103.87
52.58
49.95
40.51 dmso
40.43 dmso
40.34 dmso
40.27 dmso
40.17 dmso
40.09 dmso
40.00 dmso
39.94 dmso
39.84 dmso
39.67 dmso
39.50 dmso
36.27
28.00
24.02
24.01
N
NH2
O
O-
N+
HN
N
Cl
H3C
CH3
230 220 210 200 190 180 170 160 150 140 130 120 110 100 90 80 70 60 50 40 30 20 10 0 -10
f1 (ppm)

(4e)

4e-C

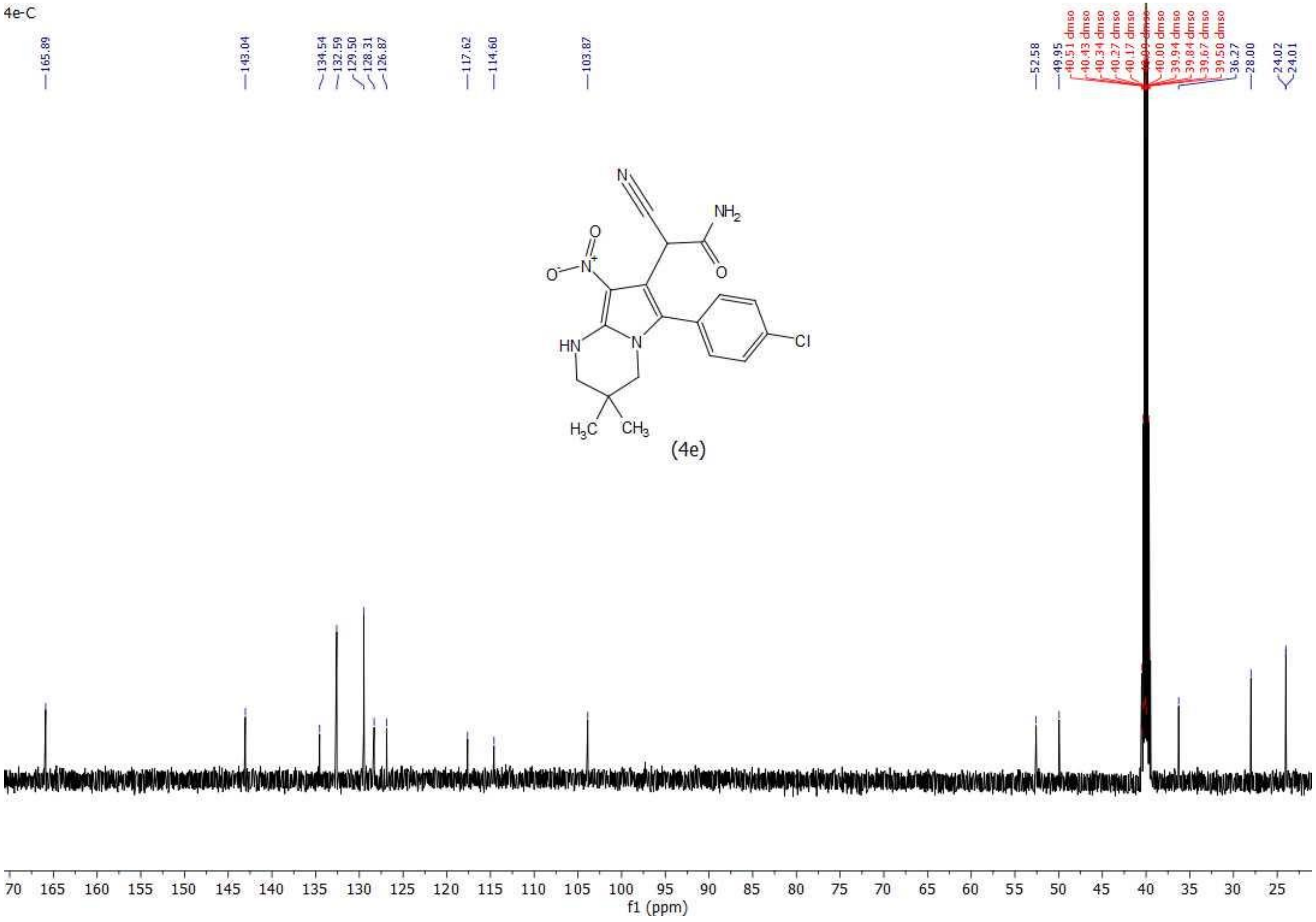

165.89
143.04
134.54
132.59
129.50
128.31
126.87
117.62
114.60
103.87
52.58
49.95
40.51 dmso
40.43 dmso
40.34 dmso
40.27 dmso
40.17 dmso
40.00 dmso
39.94 dmso
39.84 dmso
39.67 dmso
39.50 dmso
36.27
28.00
24.02
24.01
N
NH2
O
O-
N+
HN
N
Cl
H3C
CH3
(4e)
70 165 160 155 150 145 140 135 130 125 120 115 110 105 100 95 90 85 80 75 70 65 60 55 50 45 40 35 30 25
f1 (ppm)

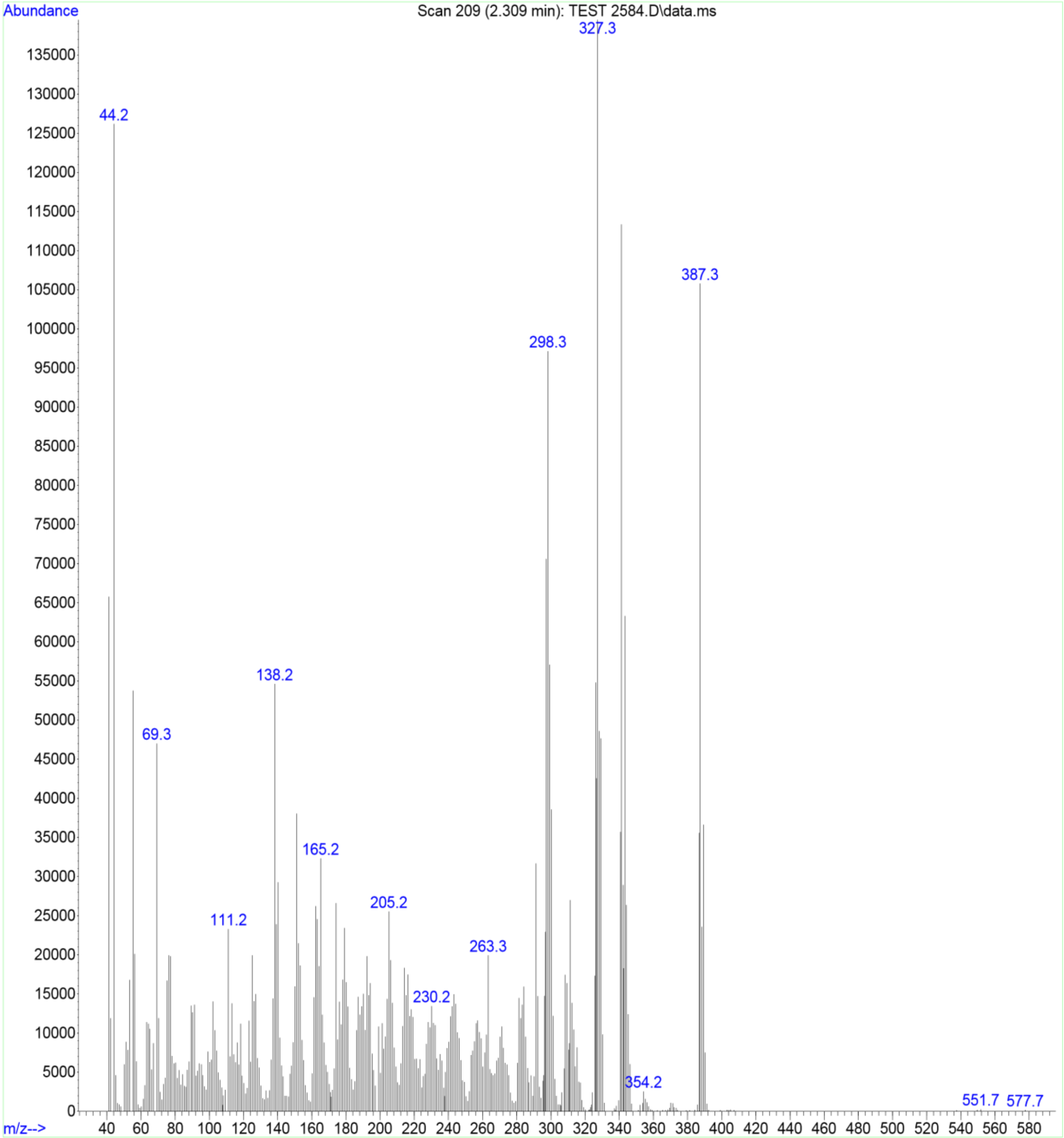

$C_{18}H_{18}ClN_5O_3$

(387/82)

(4e)

4f-H

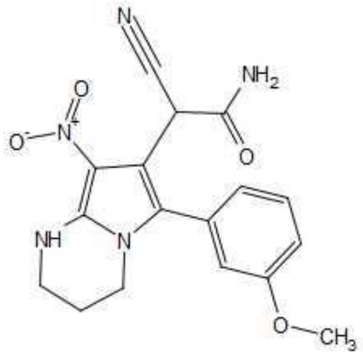
N
NH2
O
O-
N+
NH
N
O
CH3

(4f)

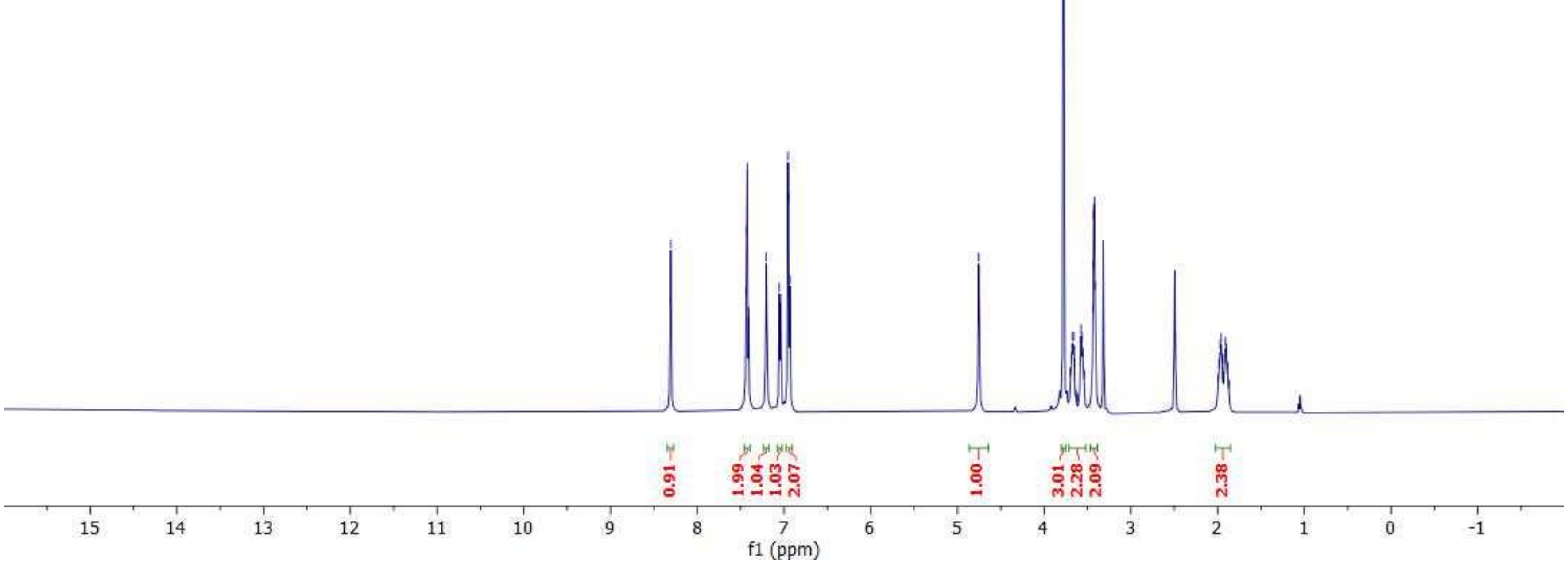
8.31
8.31
7.44
7.43
7.42
7.41
7.40
7.20
7.06
7.05
7.04
7.04
6.96
6.95
6.95
6.93
4.75
3.78
3.77
3.70
3.69
3.67
3.66
3.65
3.63
3.62
3.59
3.57
3.56
3.55
3.54
3.44
3.43
3.43
3.42
3.41
2.00
1.98
1.97
1.96
1.95
1.93
1.92
1.91
1.90
1.88
1.87
0.91
1.99
1.04
1.03
2.07
1.00
3.01
2.28
2.09
2.38
15
14
13
12
11
10
9
8
7
6
5
4
3
2
1
0
-1
f1 (ppm)

4f-H

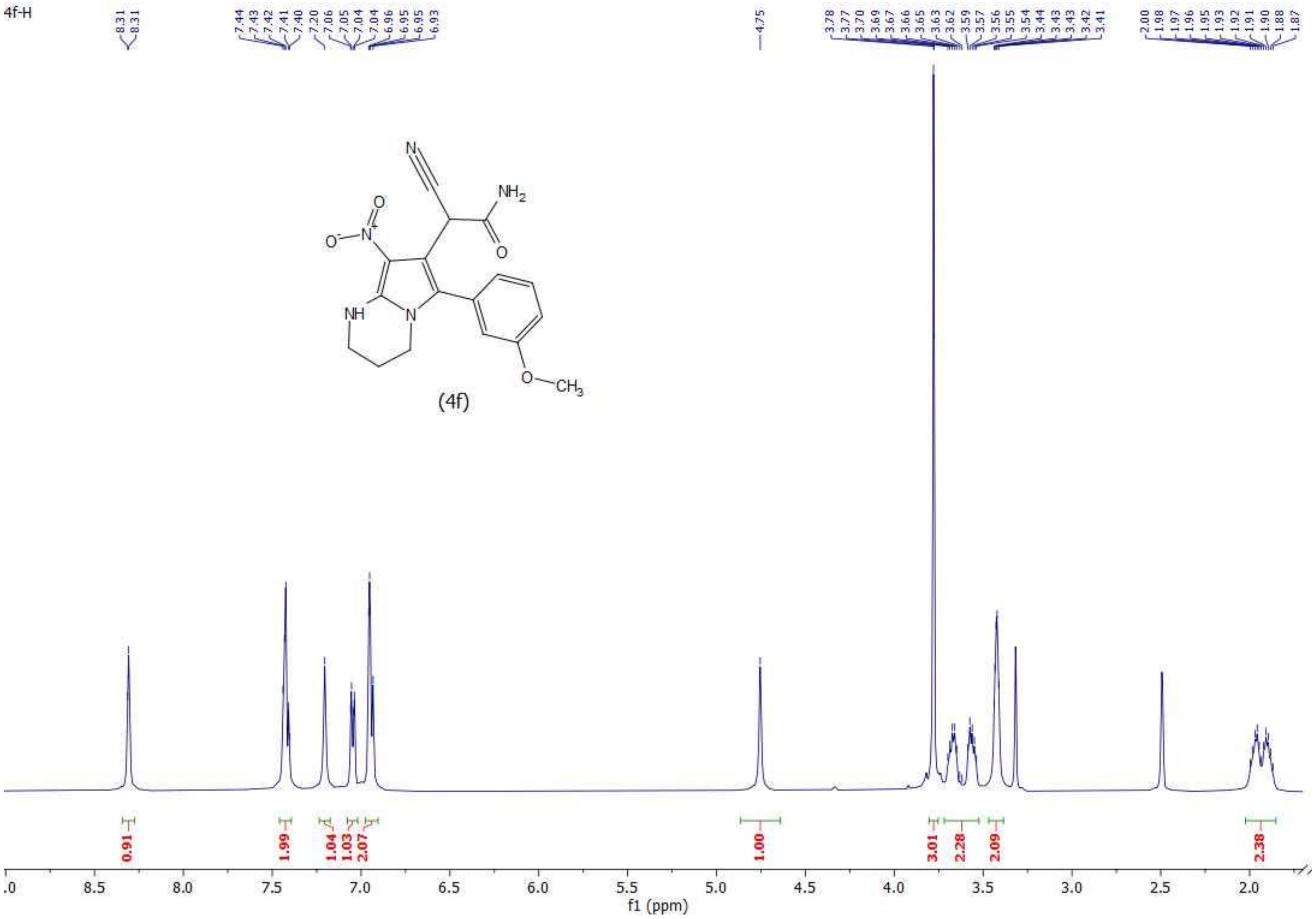
8.31 8.31
7.44 7.43 7.42 7.41 7.40 7.20 7.06 7.05 7.04 7.04 6.96 6.95 6.95 6.93
4.75
3.78 3.77 3.70 3.69 3.67 3.66 3.65 3.63 3.62 3.59 3.57 3.56 3.55 3.54 3.44 3.43 3.43 3.42 3.41
2.00 1.98 1.97 1.96 1.95 1.93 1.92 1.91 1.90 1.88 1.87
NH2
N
O
O-
N+
NH
O
CH3
(4f)
0.91
1.99
1.04
1.03
2.07
1.00
3.01
2.28
2.09
2.38
f1 (ppm)

4f-C

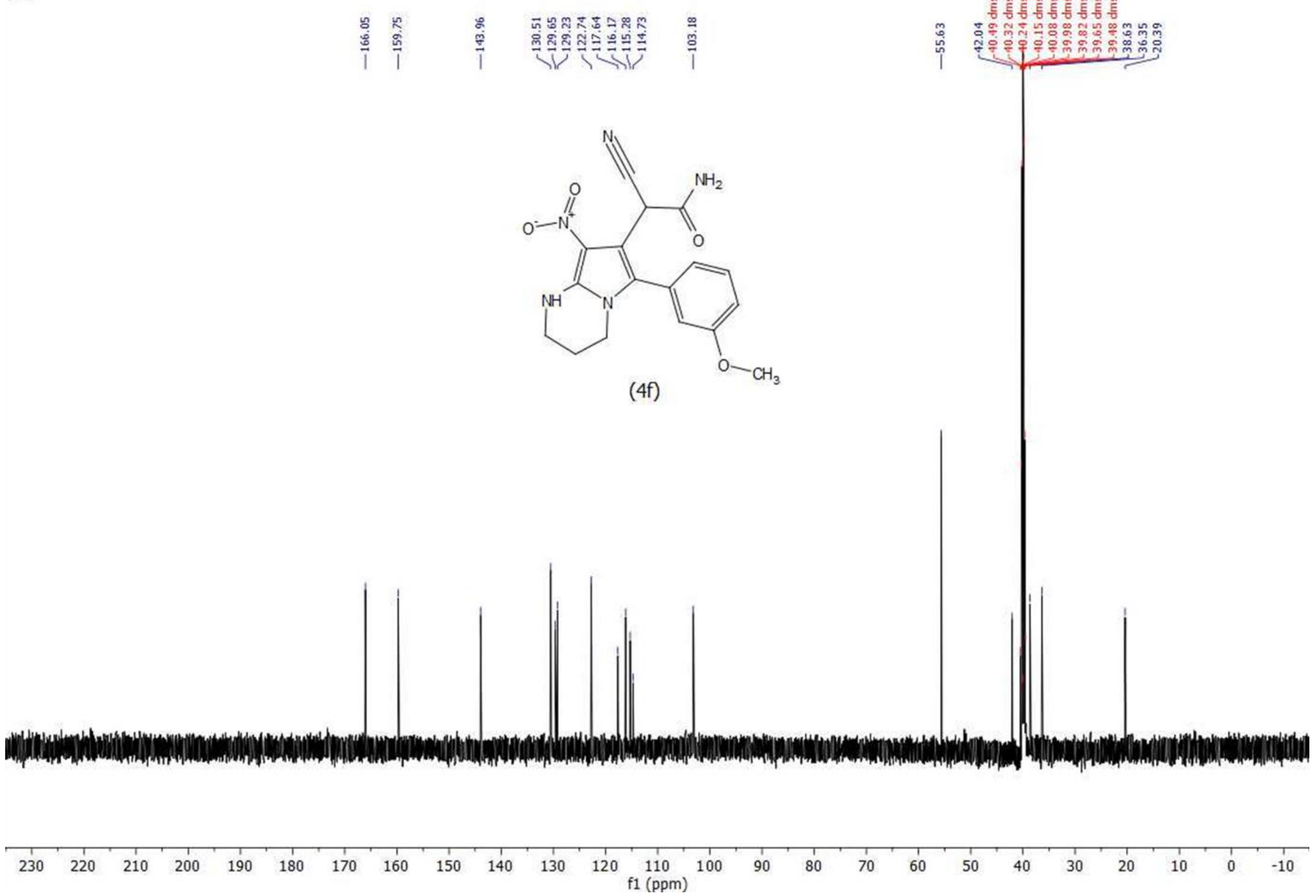

166.05
159.75
143.96
130.51
129.65
129.23
122.74
117.64
116.17
115.28
114.73
103.18
55.63
42.04
40.49 dmso
40.32 dmso
40.24 dmso
40.15 dmso
40.08 dmso
39.98 dmso
39.82 dmso
39.65 dmso
39.48 dmso
38.63
36.35
20.39
N
NH2
O
O-
N+
NH
N
O
CH3
(4f)
230 220 210 200 190 180 170 160 150 140 130 120 110 100 90 80 70 60 50 40 30 20 10 0 -10
f1 (ppm)

4g-H

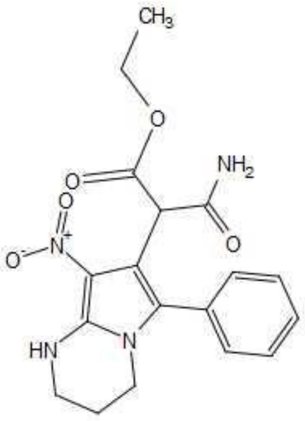

(4g)

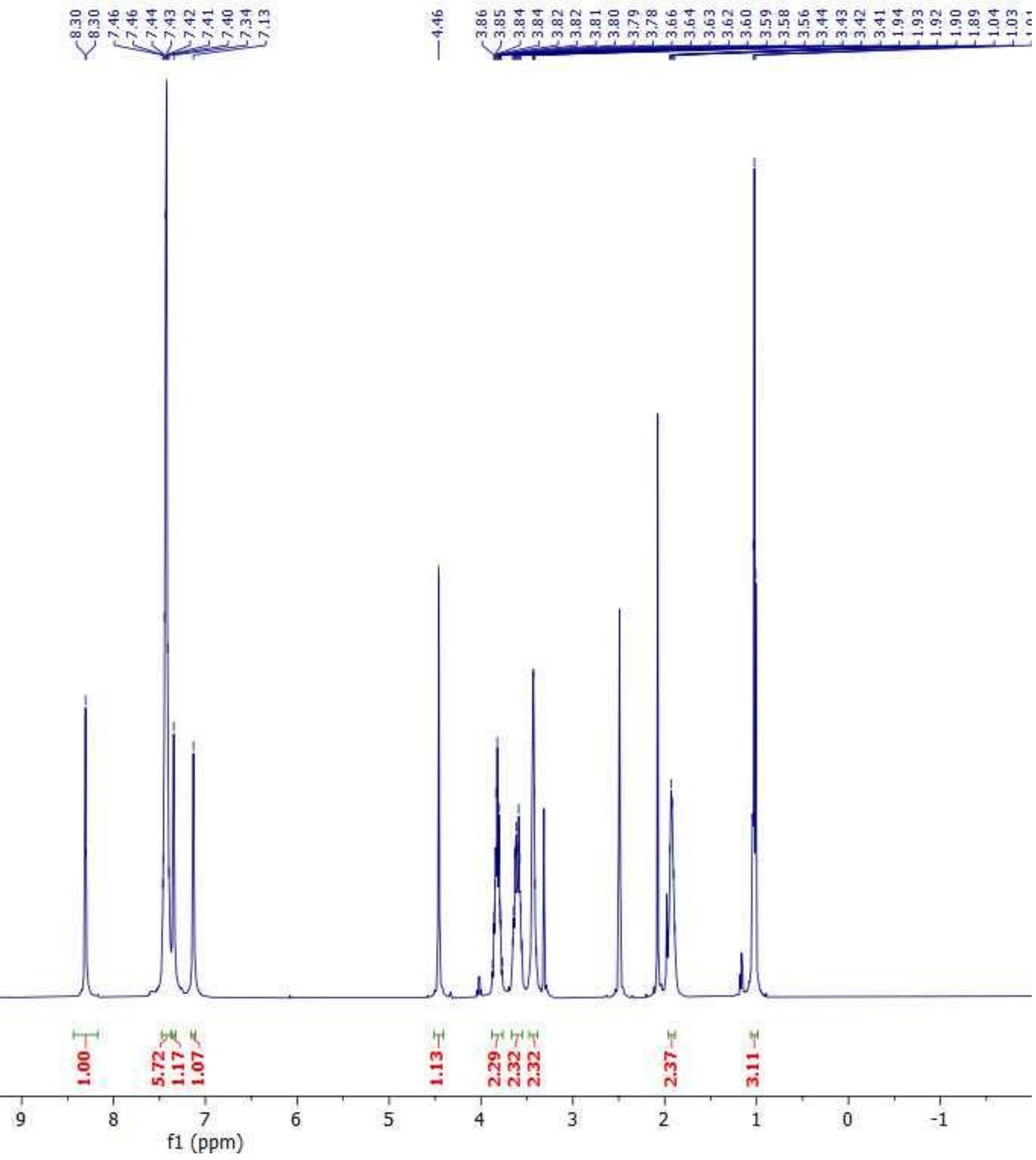
8.30
8.30
7.46
7.46
7.44
7.43
7.42
7.41
7.40
7.34
7.13
4.46
3.86
3.85
3.84
3.84
3.82
3.82
3.81
3.80
3.79
3.78
3.66
3.64
3.63
3.62
3.60
3.59
3.58
3.56
3.44
3.43
3.42
3.41
1.94
1.93
1.92
1.90
1.89
1.04
1.03
1.00
5.72
1.17
1.07
1.13
2.29
2.32
2.32
2.37
3.11
15
14
13
12
11
10
9
8
7
6
5
4
3
2
1
0
-1
f1 (ppm)

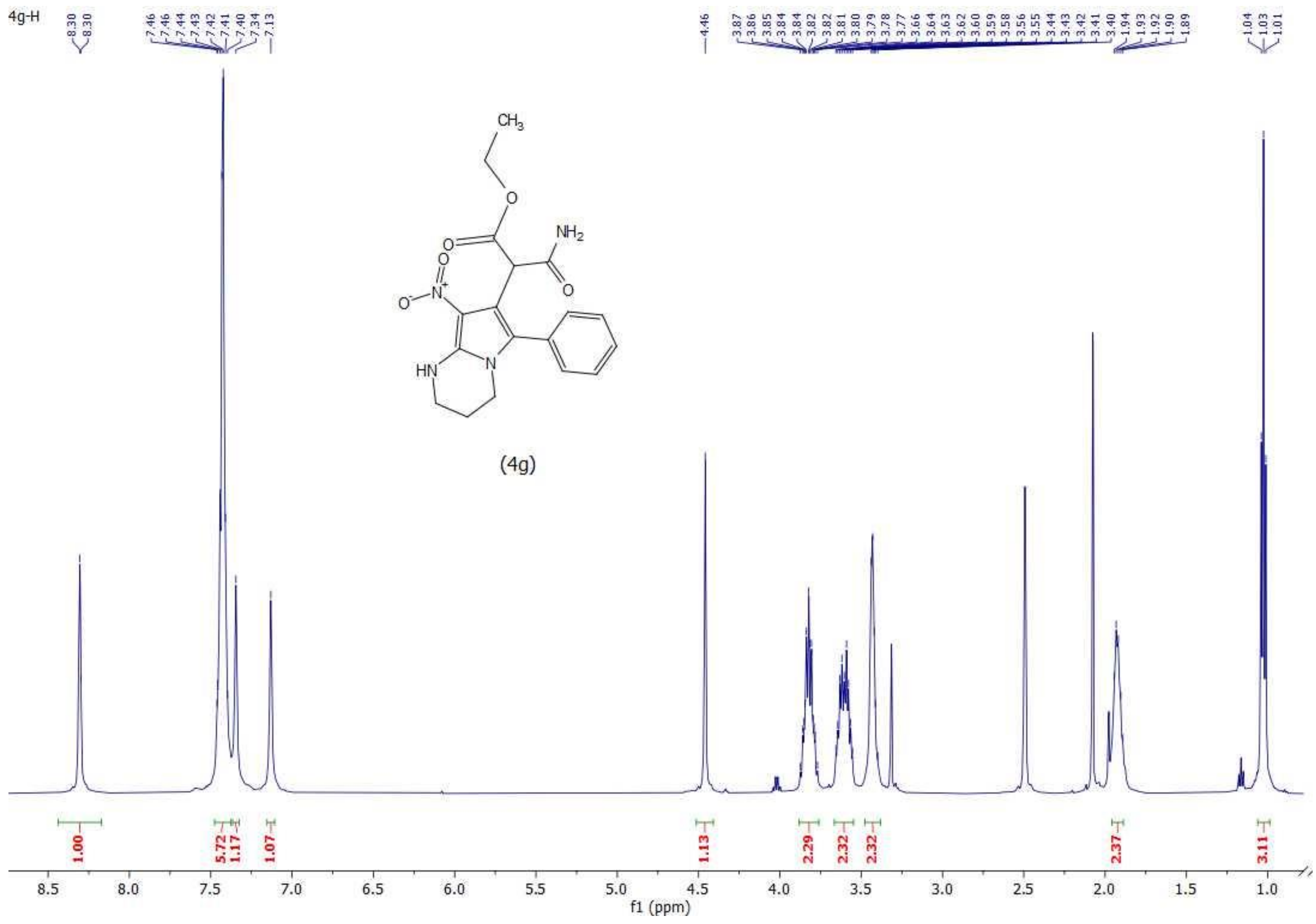
4g-H
8.30
8.30
7.46
7.46
7.44
7.43
7.42
7.41
7.40
7.34
7.13
4.46
3.87
3.86
3.85
3.84
3.84
3.82
3.82
3.81
3.80
3.79
3.78
3.77
3.66
3.64
3.63
3.62
3.60
3.59
3.58
3.56
3.55
3.44
3.43
3.42
3.41
3.40
1.94
1.93
1.92
1.90
1.89
1.04
1.03
1.01
(4g)
1.00
5.72
1.17
1.07
1.13
2.29
2.32
2.32
2.37
3.11
f1 (ppm)

4g-C

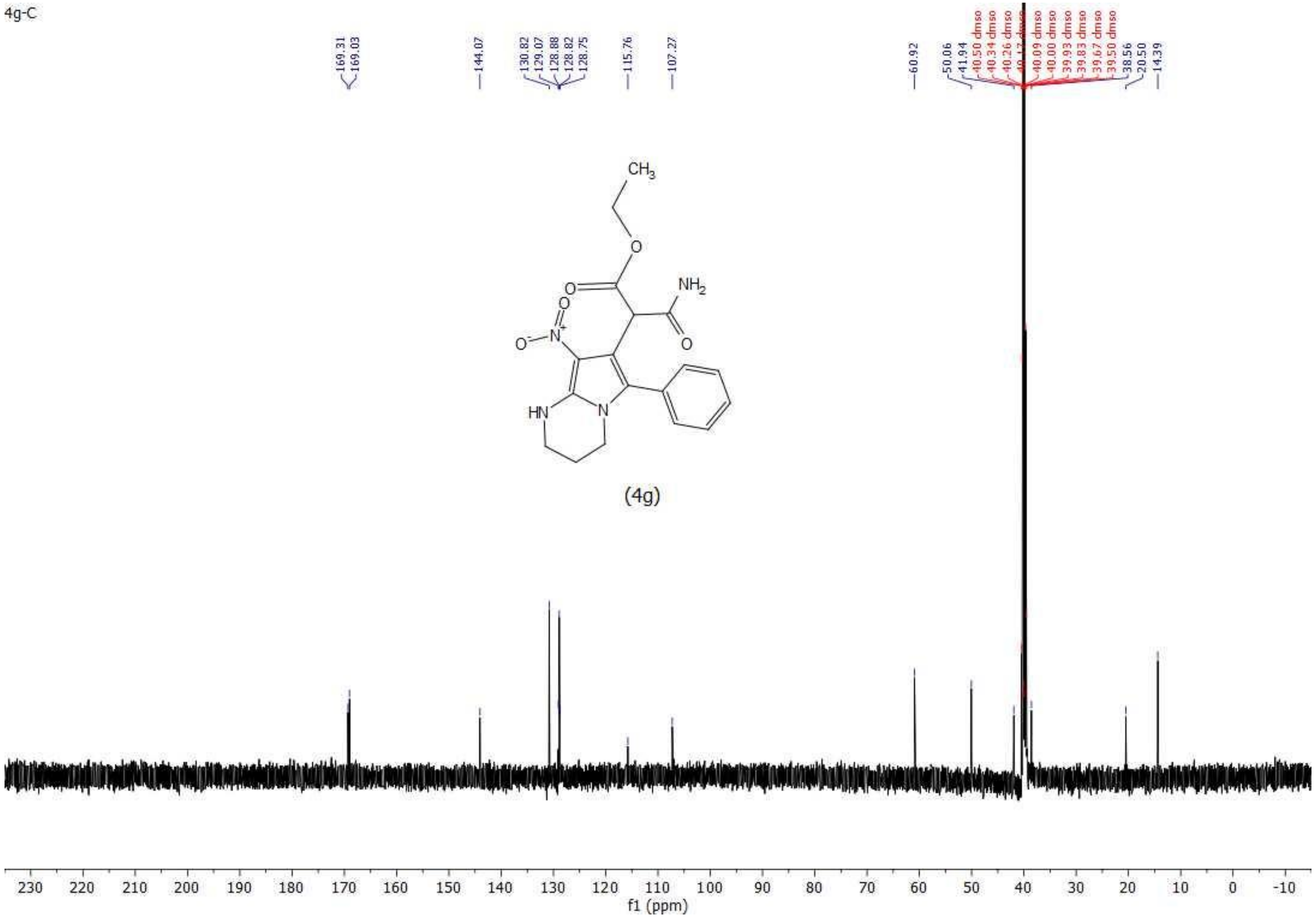
169.31
169.03
144.07
130.82
129.07
128.88
128.82
128.75
115.76
107.27
60.92
50.06
41.94
40.50 dmso
40.34 dmso
40.26 dmso
40.17 dmso
40.09 dmso
40.00 dmso
39.93 dmso
39.83 dmso
39.67 dmso
39.50 dmso
38.56
20.50
14.39
CH3
O
NH2
O
O
N+
O-
HN
N
230 220 210 200 190 180 170 160 150 140 130 120 110 100 90 80 70 60 50 40 30 20 10 0 -10
f1 (ppm)

(4g)

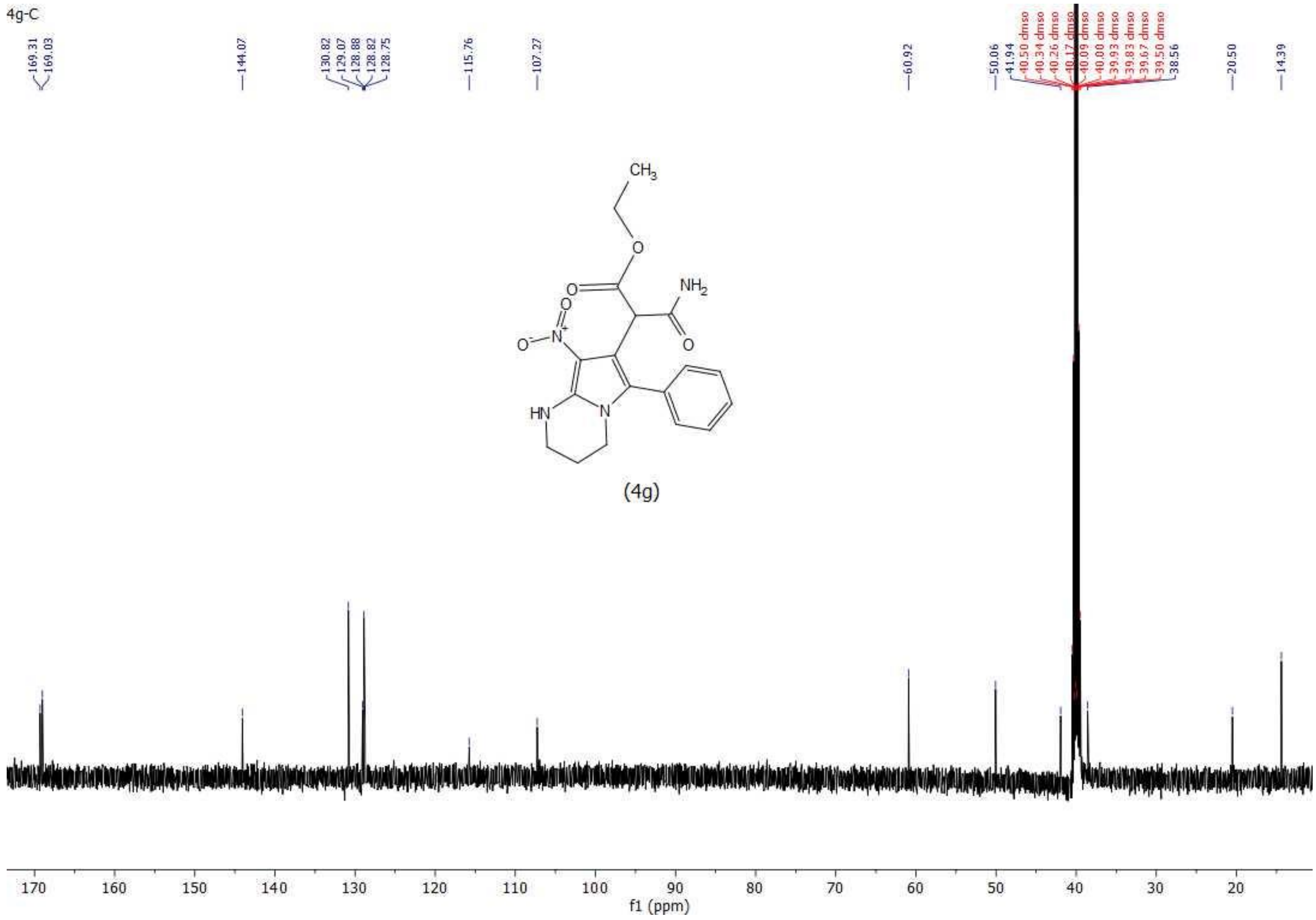
4g-C
169.31
169.03
144.07
130.82
129.07
128.88
128.82
128.75
115.76
107.27
60.92
50.06
41.94
40.50 dmso
40.34 dmso
40.26 dmso
40.17 dmso
40.09 dmso
40.00 dmso
39.93 dmso
39.83 dmso
39.67 dmso
39.50 dmso
38.56
20.50
14.39
CH3
O
O
NH2
O
N
O
HN
N
(4g)
170
160
150
140
130
120
110
100
90
80
70
60
50
40
30
20
f1 (ppm)

4h-H

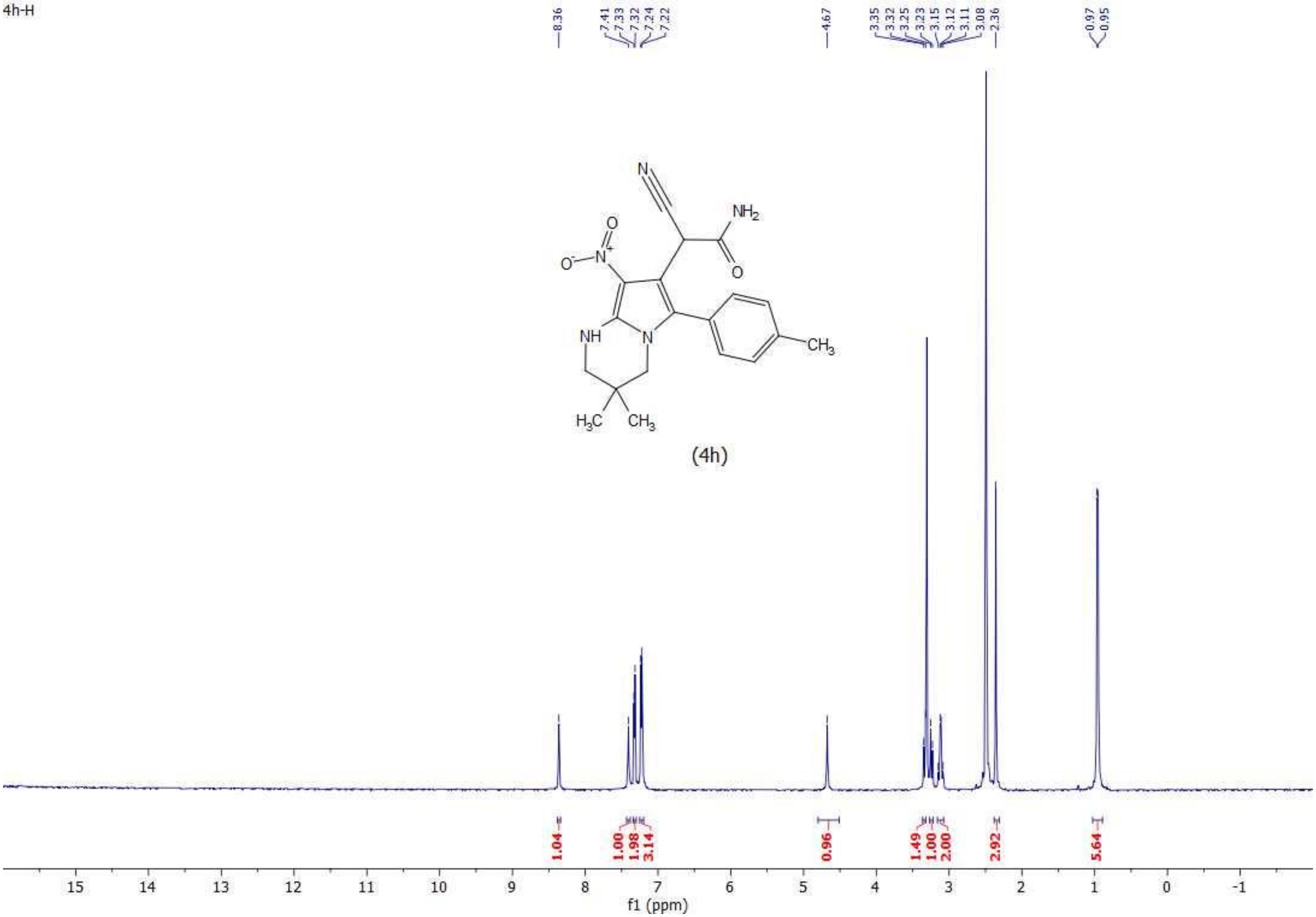

8.36
7.41
7.33
7.32
7.24
7.22
4.67
3.35
3.32
3.25
3.23
3.15
3.12
3.11
3.08
2.36
0.97
0.95
N
NH2
O
O-
N+
NH
N
CH3
H3C
CH3
(4h)
1.04
1.00
1.98
3.14
0.96
1.49
1.00
2.00
2.92
5.64
15
14
13
12
11
10
9
8
7
6
5
4
3
2
1
0
-1
f1 (ppm)

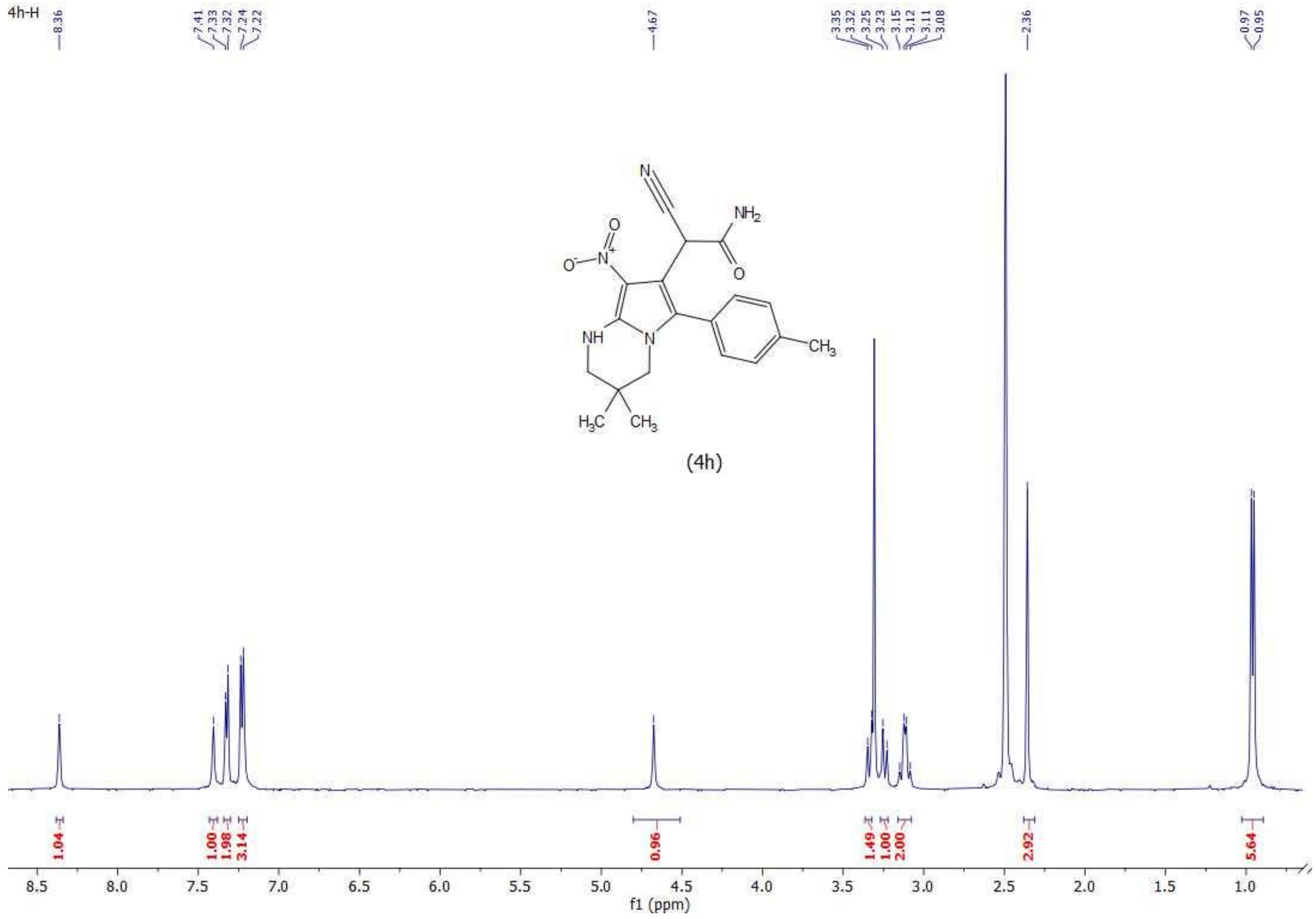
4h-H
8.36
7.41
7.33
7.32
7.24
7.22
4.67
3.35
3.32
3.25
3.23
3.15
3.12
3.11
3.08
2.36
0.97
0.95
NH₂
O
N
O⁻
N⁺
NH
N
CH₃
H₃C
CH₃
(4h)
1.04
1.00
1.98
3.14
0.96
1.49
1.00
2.00
2.92
5.64
8.5
8.0
7.5
7.0
6.5
6.0
5.5
5.0
4.5
4.0
3.5
3.0
2.5
2.0
1.5
1.0
f1 (ppm)

4h-C

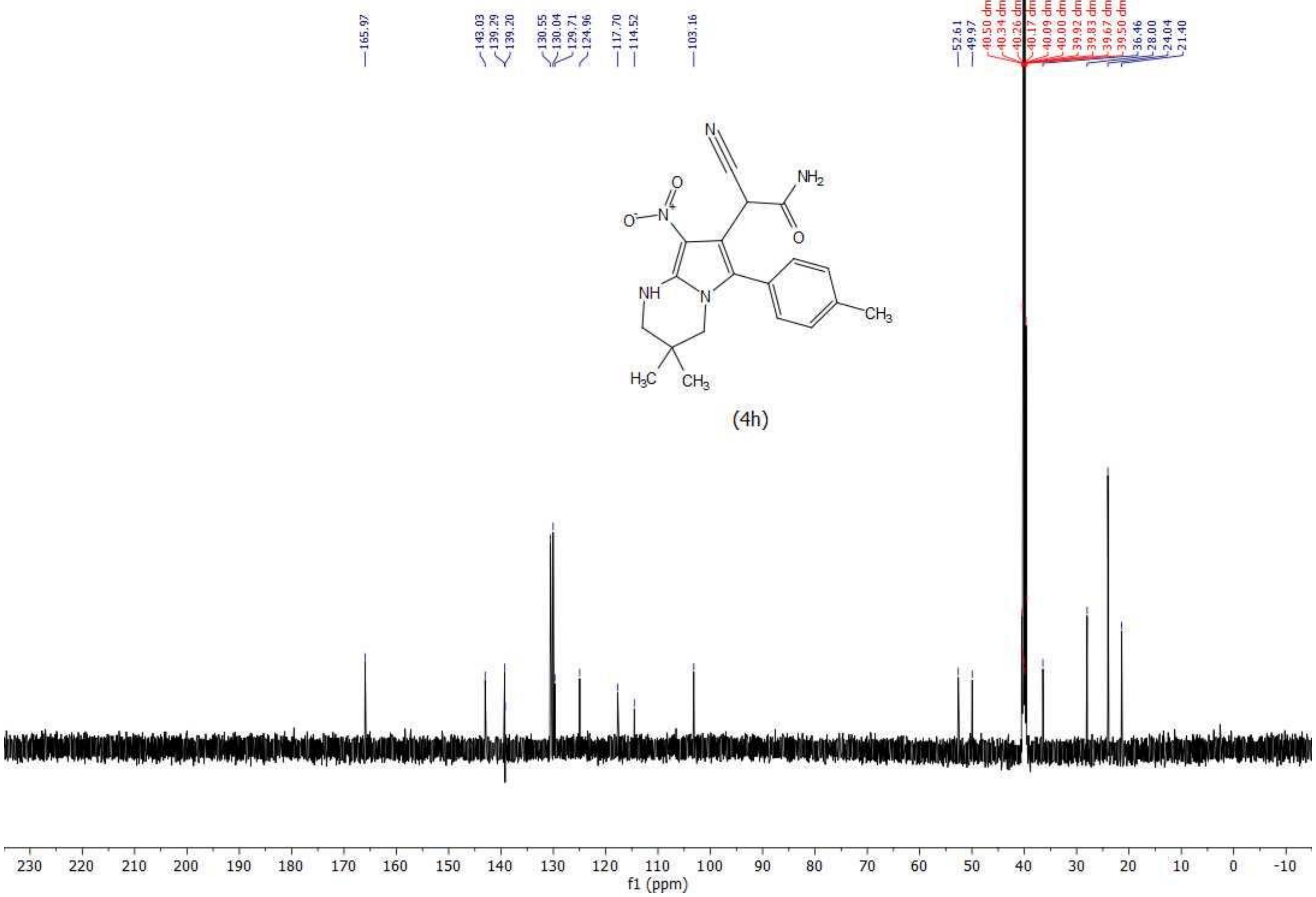

165.97
143.03
139.29
139.20
130.55
130.04
129.71
124.96
117.70
114.52
103.16
52.61
49.97
40.50 dmso
40.34 dmso
40.26 dmso
40.17 dmso
40.09 dmso
40.00 dmso
39.92 dmso
39.83 dmso
39.67 dmso
39.50 dmso
36.46
28.00
24.04
21.40
N
NH2
O
O-
N+
NH
N
CH3
H3C
CH3
230 220 210 200 190 180 170 160 150 140 130 120 110 100 90 80 70 60 50 40 30 20 10 0 -10
f1 (ppm)

(4h)

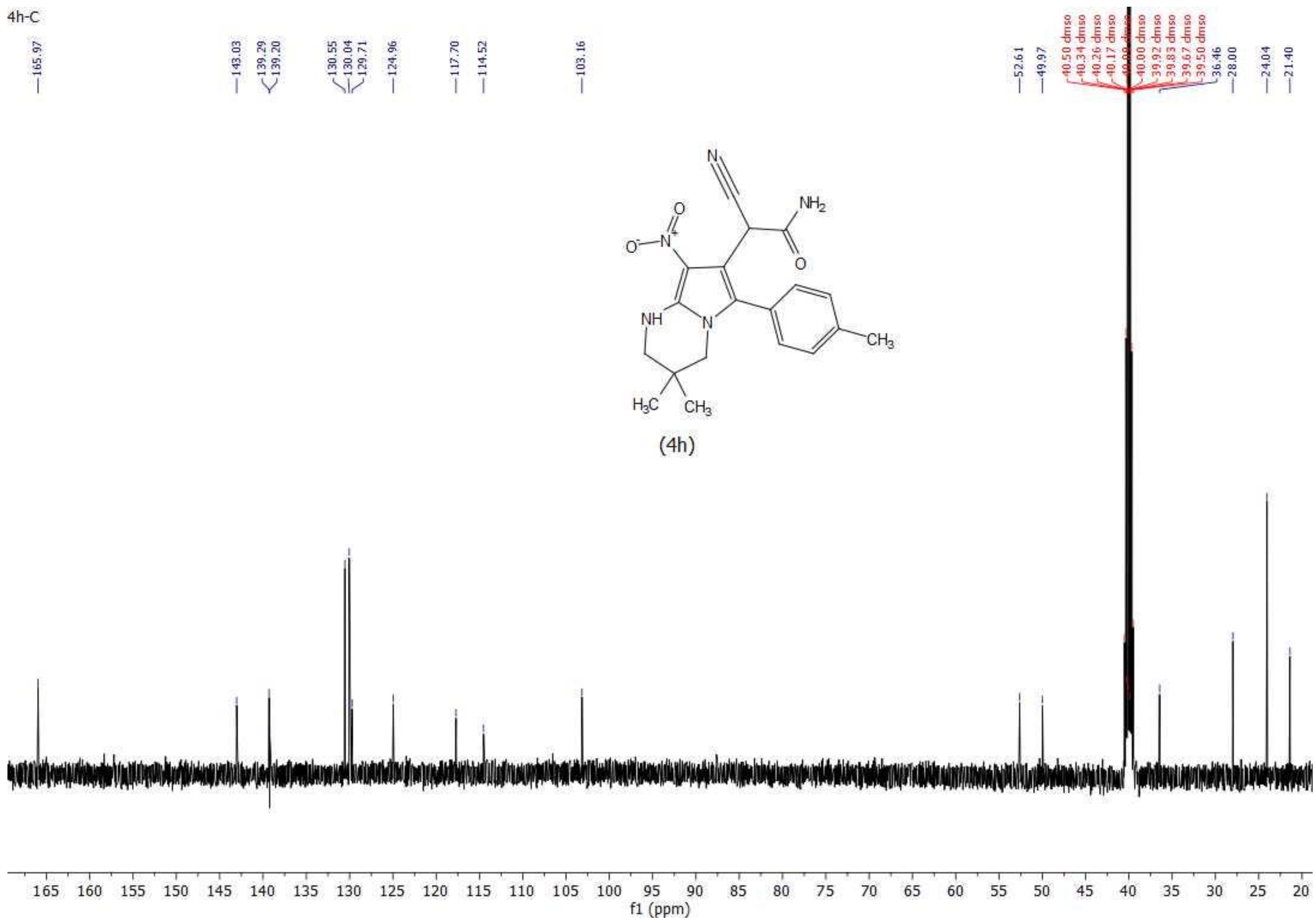
4h-C
165.97
143.03
139.29
139.20
130.55
130.04
129.71
124.96
117.70
114.52
103.16
52.61
49.97
40.50 dmso
40.34 dmso
40.26 dmso
40.17 dmso
40.00 dmso
39.92 dmso
39.83 dmso
39.67 dmso
39.50 dmso
36.46
28.00
24.04
21.40
N
NH2
O
O-
N+
NH
N
CH3
H3C
CH3
(4h)
f1 (ppm)

4i-H

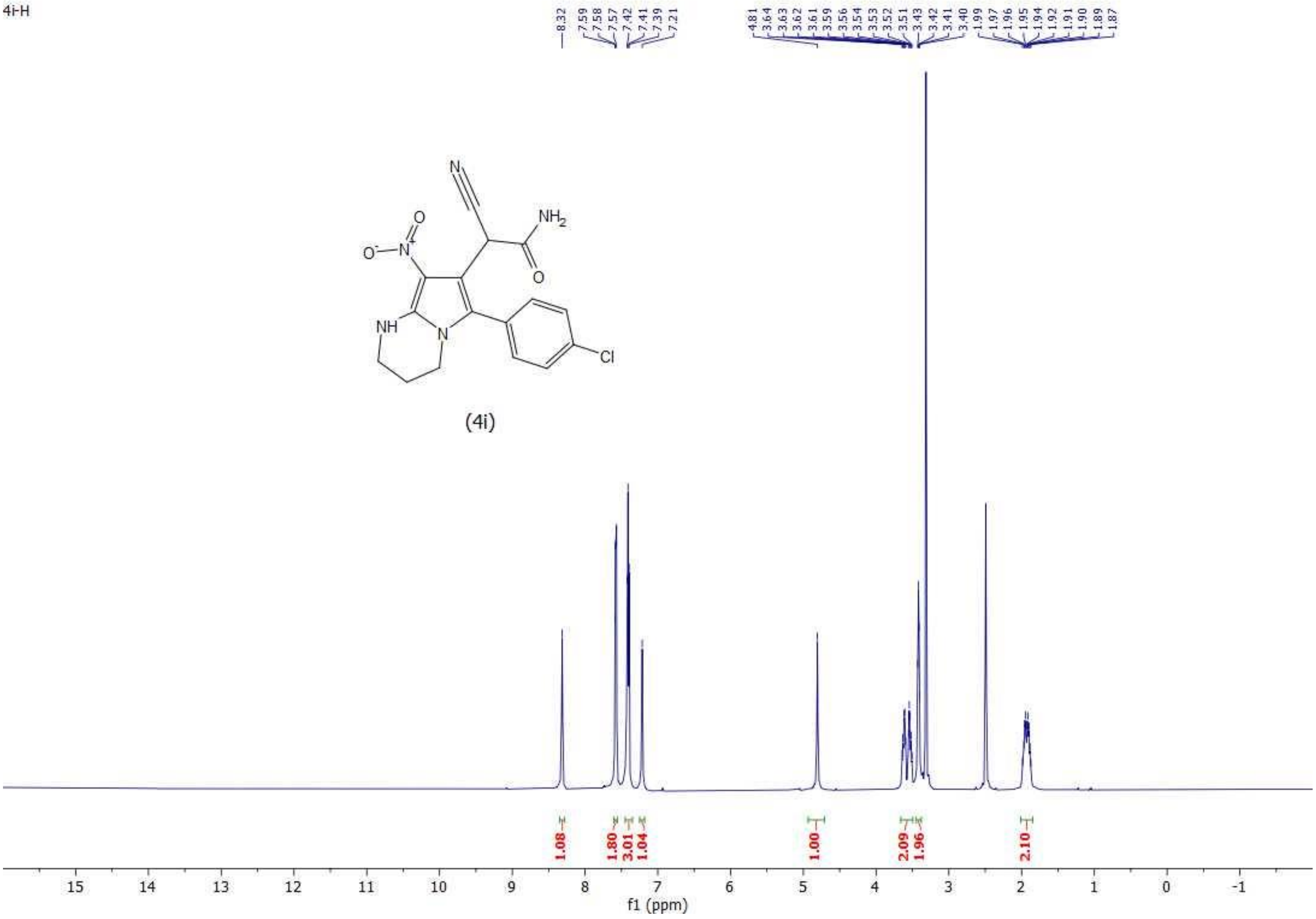
8.32
7.59
7.58
7.57
7.42
7.41
7.39
7.21
4.81
3.64
3.63
3.62
3.61
3.59
3.56
3.54
3.53
3.52
3.51
3.43
3.42
3.41
3.40
1.99
1.97
1.96
1.95
1.94
1.92
1.91
1.90
1.89
1.87
N
NH2
O
O
N+
O-
NH
N
Cl
(4i)
1.08
1.80
3.01
1.04
1.00
2.09
1.96
2.10
15
14
13
12
11
10
9
8
7
6
5
4
3
2
1
0
-1
f1 (ppm)

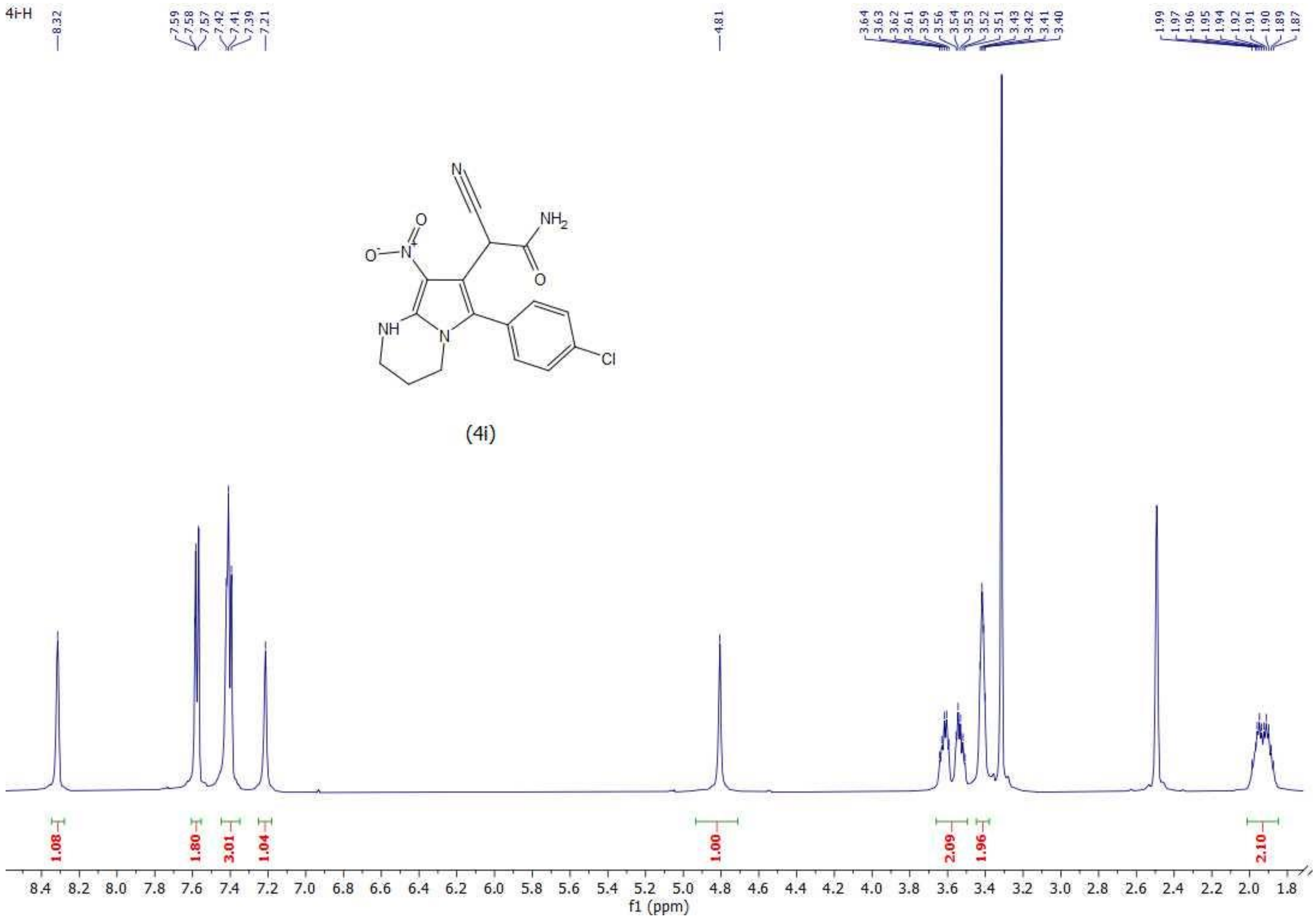
4i-H
8.32
7.59
7.58
7.57
7.42
7.41
7.39
7.21
4.81
3.64
3.63
3.62
3.61
3.59
3.56
3.54
3.53
3.52
3.51
3.43
3.42
3.41
3.40
1.99
1.97
1.96
1.95
1.94
1.92
1.91
1.90
1.89
1.87
N
NH2
O
O-
N+
NH
N
Cl
(4i)
1.08
1.80
3.01
1.04
1.00
2.09
1.96
2.10
8.4 8.2 8.0 7.8 7.6 7.4 7.2 7.0 6.8 6.6 6.4 6.2 6.0 5.8 5.6 5.4 5.2 5.0 4.8 4.6 4.4 4.2 4.0 3.8 3.6 3.4 3.2 3.0 2.8 2.6 2.4 2.2 2.0 1.8
f1 (ppm)

4i-C

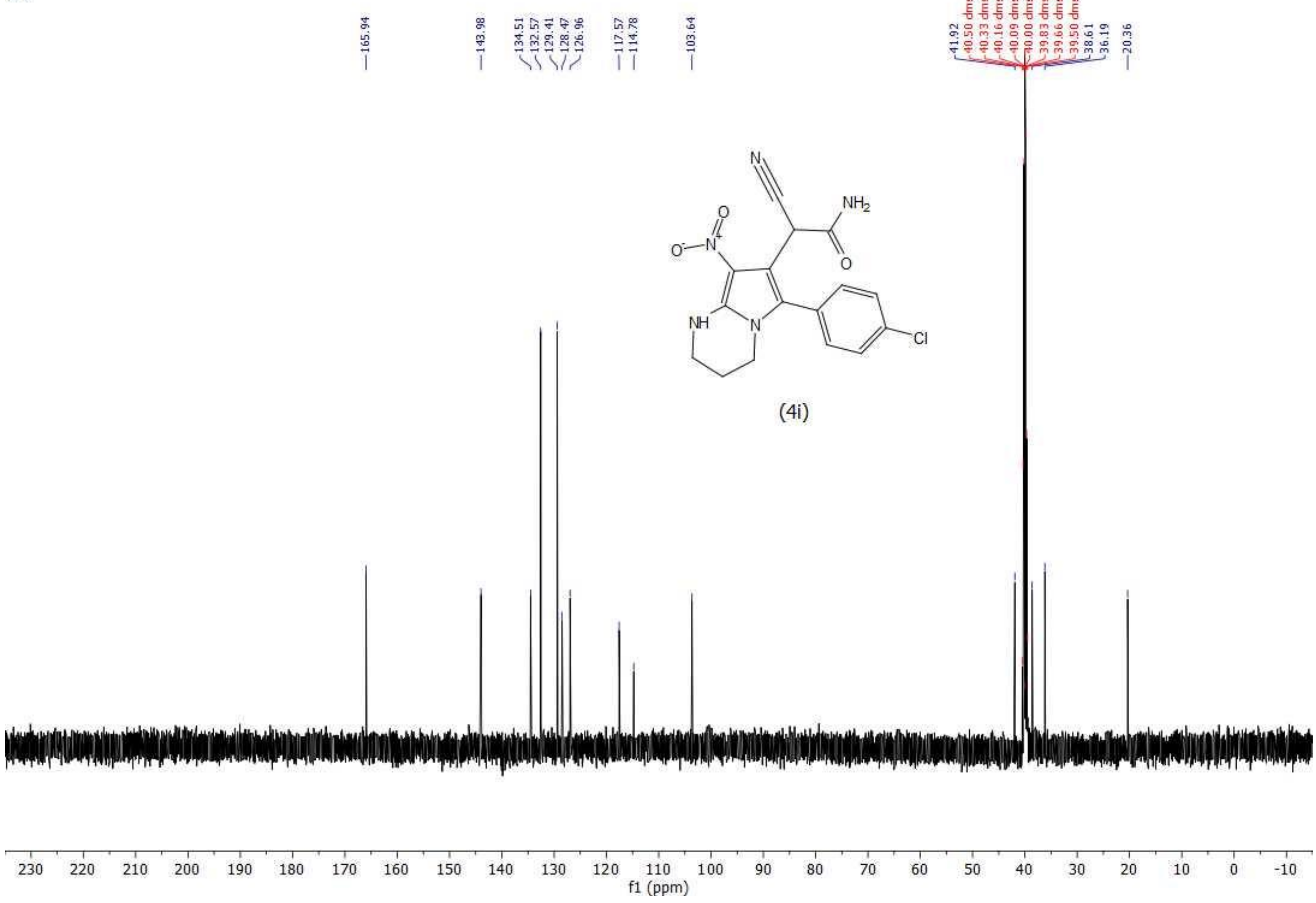
165.94
143.98
134.51
132.57
129.41
128.47
126.96
117.57
114.78
103.64
41.92
40.50 dmso
40.33 dmso
40.16 dmso
40.09 dmso
40.00 dmso
39.83 dmso
39.66 dmso
39.50 dmso
38.61
36.19
20.36
N
NH2
O
O
N+
O-
NH
N
Cl
(4i)
230 220 210 200 190 180 170 160 150 140 130 120 110 100 90 80 70 60 50 40 30 20 10 0 -10
f1 (ppm)

4i-C

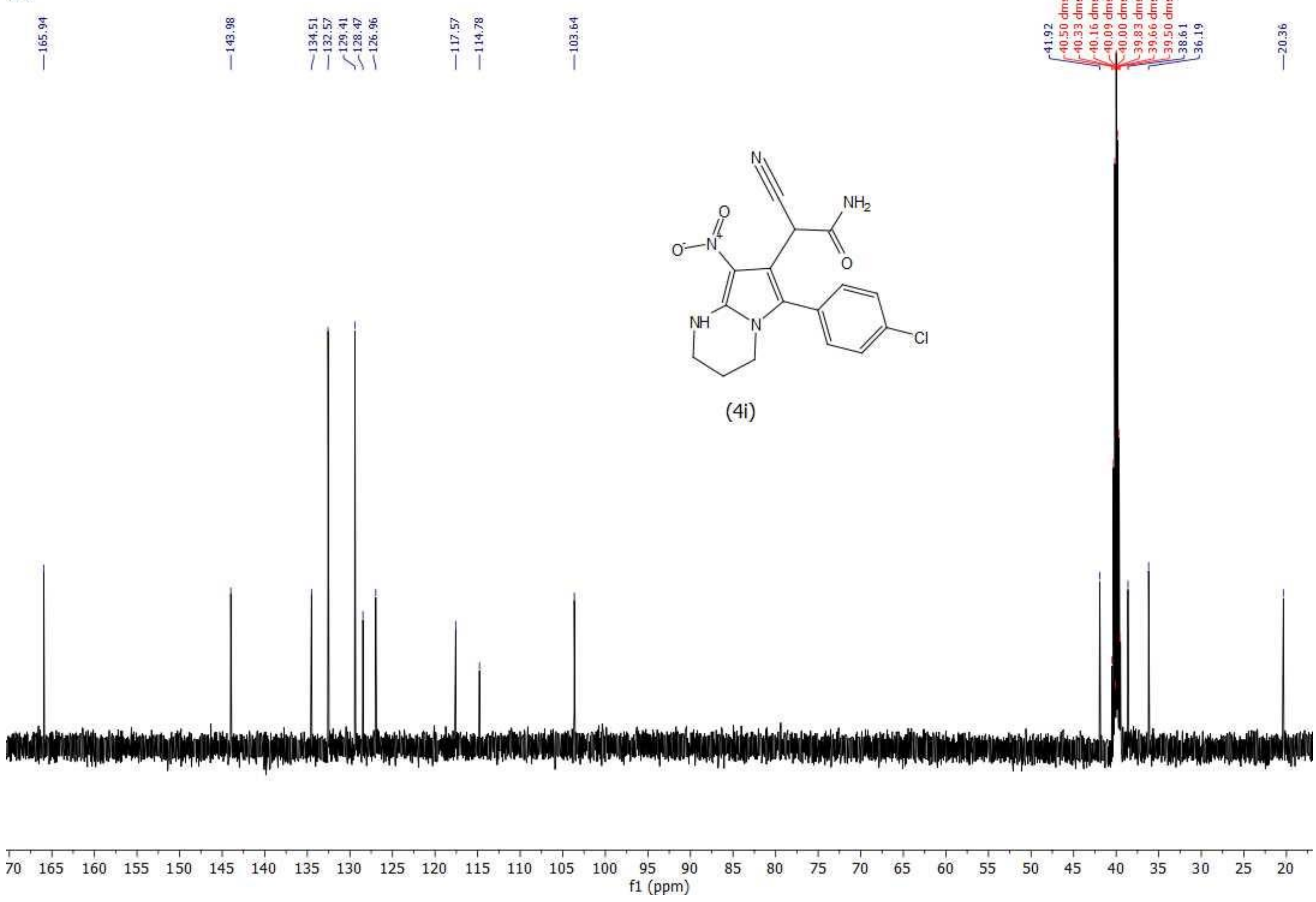
165.94
143.98
134.51
132.57
129.41
128.47
126.96
117.57
114.78
103.64
41.92
40.50 dmso
40.33 dmso
40.16 dmso
40.09 dmso
40.00 dmso
39.83 dmso
39.66 dmso
39.50 dmso
38.61
36.19
20.36
(4i)
70 165 160 155 150 145 140 135 130 125 120 115 110 105 100 95 90 85 80 75 70 65 60 55 50 45 40 35 30 25 20
f1 (ppm)

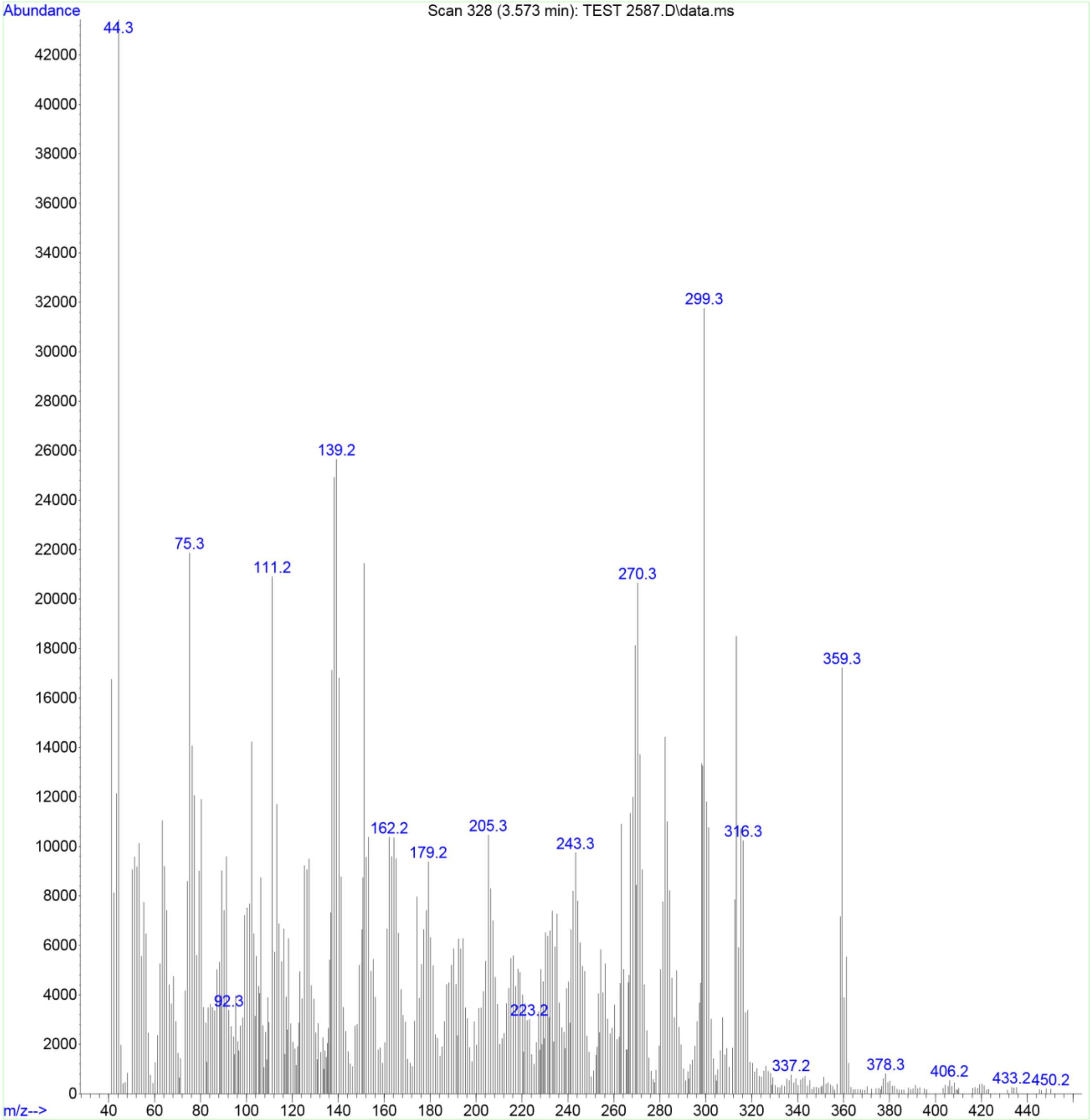

$C_{16}H_{14}ClN_5O_3$

(359.7)

(4i)

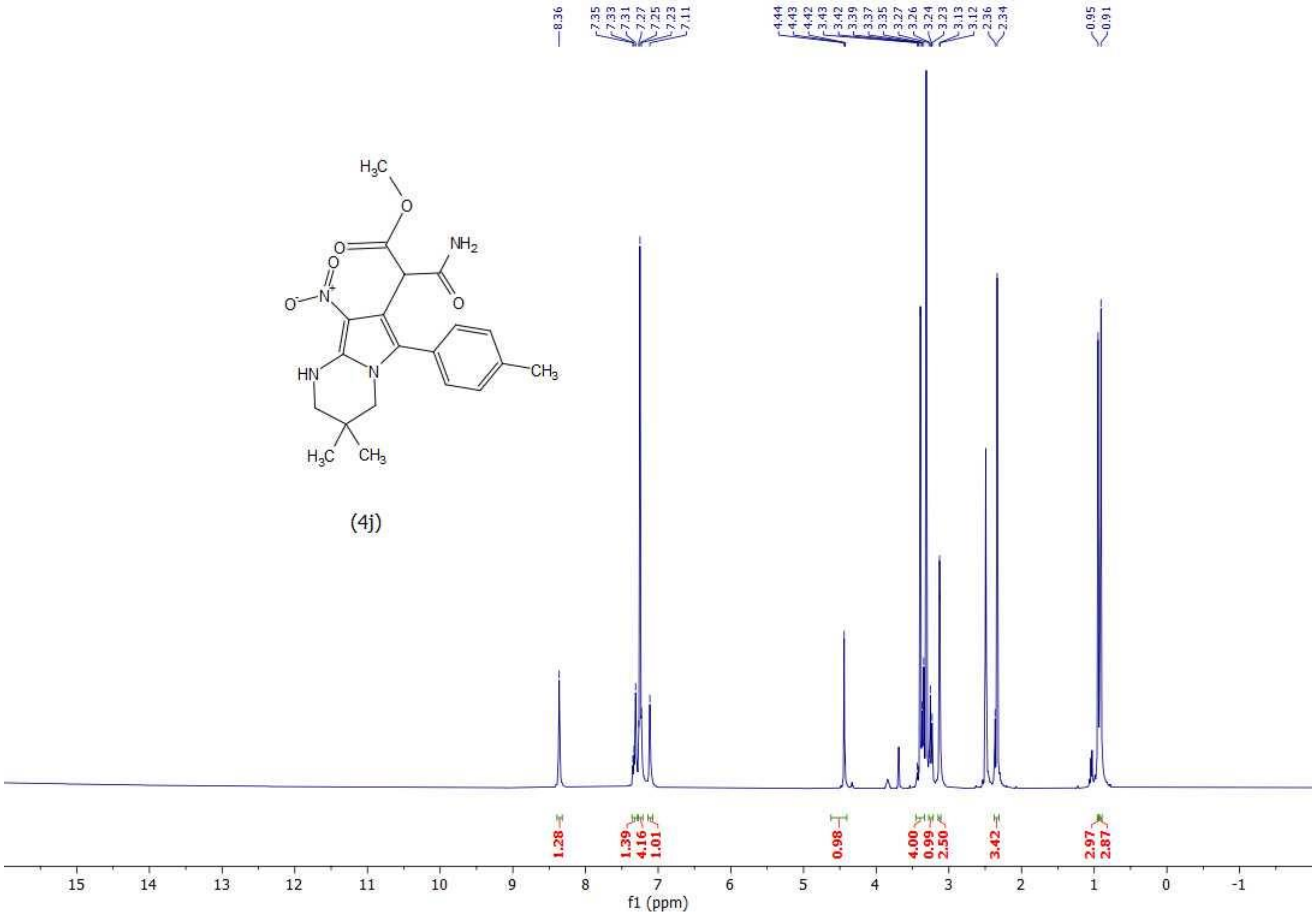
H3C
O
O
NH2
O
O
N+
O-
HN
N
CH3
H3C
CH3
(4j)
8.36
7.35
7.33
7.31
7.27
7.25
7.23
7.11
4.44
4.43
4.42
3.43
3.42
3.39
3.37
3.35
3.27
3.26
3.24
3.23
3.13
3.12
2.36
2.34
0.95
0.91
1.28
1.39
4.16
1.01
0.98
4.00
0.99
2.50
3.42
2.97
2.87
15
14
13
12
11
10
9
8
7
6
5
4
3
2
1
0
-1
f1 (ppm)

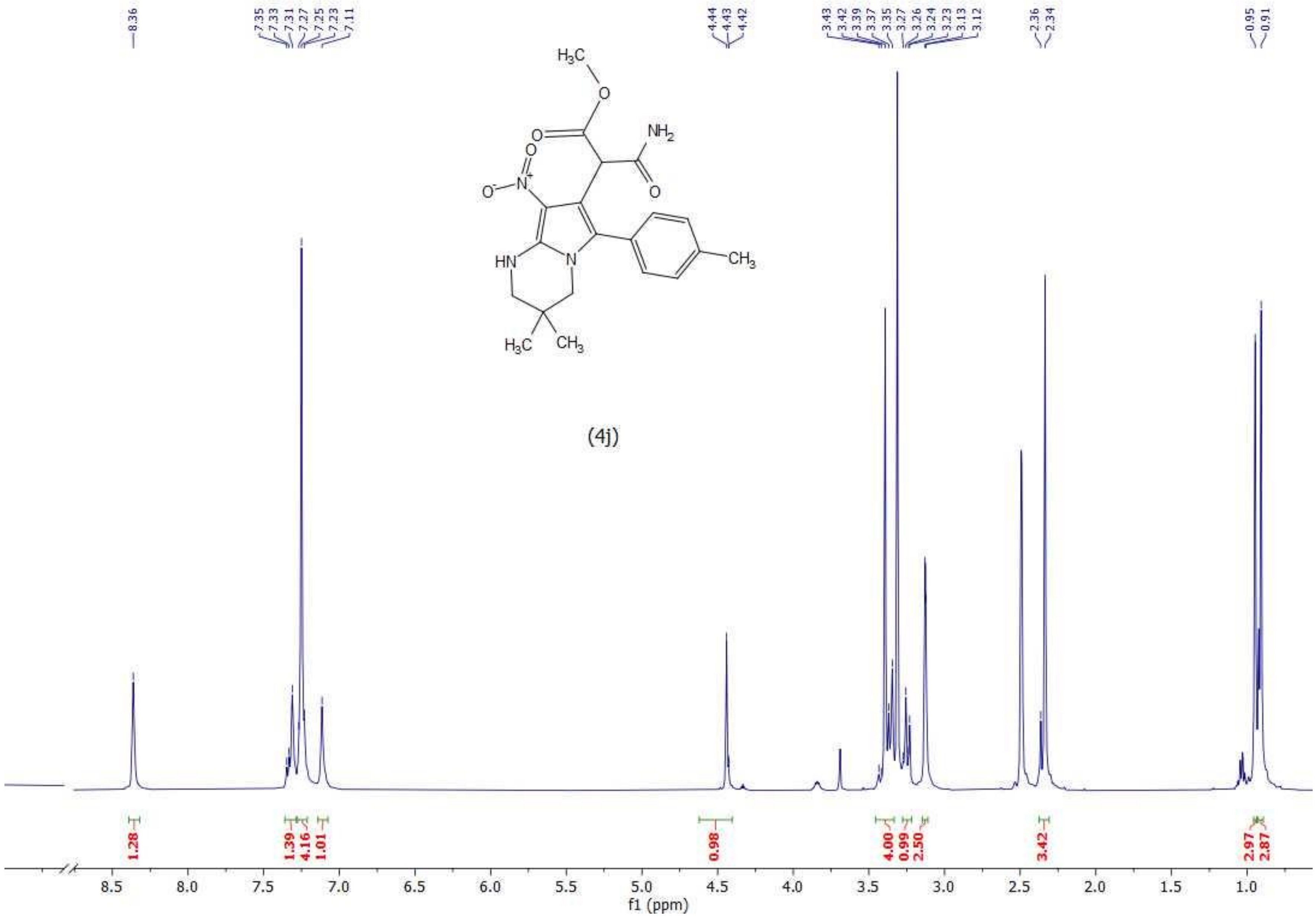
8.36
7.35
7.33
7.31
7.27
7.25
7.23
7.11
4.44
4.43
4.42
3.43
3.42
3.39
3.37
3.35
3.27
3.26
3.24
3.23
3.13
3.12
2.36
2.34
0.95
0.91
(4j)
1.28
1.39
4.16
1.01
0.98
4.00
0.99
2.50
3.42
2.97
2.87
8.5
8.0
7.5
7.0
6.5
6.0
5.5
5.0
4.5
4.0
3.5
3.0
2.5
2.0
1.5
1.0
f1 (ppm)

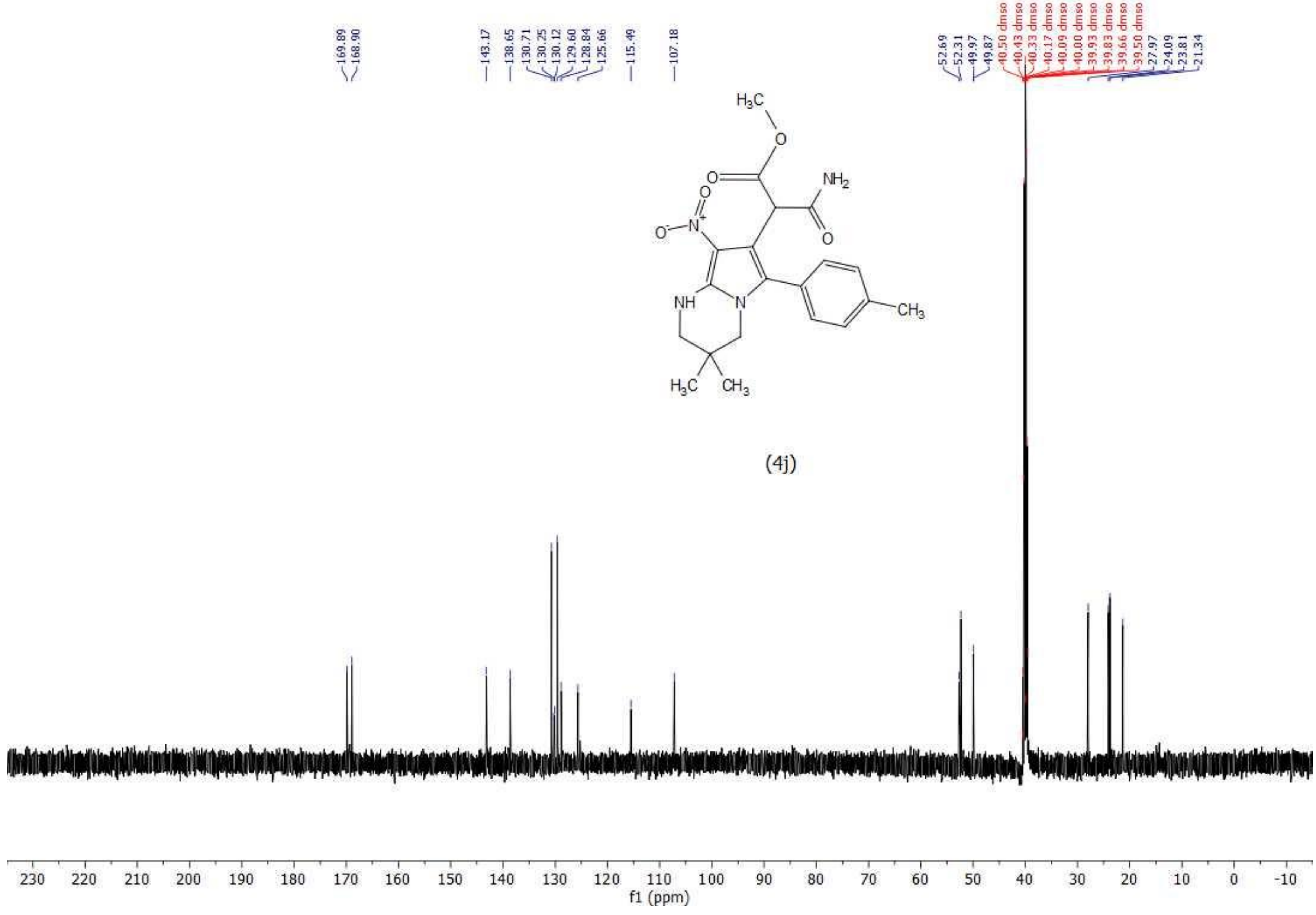
169.89
168.90
143.17
138.65
130.71
130.25
130.12
129.60
128.84
125.66
115.49
107.18
52.69
52.31
49.97
49.87
40.50 dmso
40.43 dmso
40.33 dmso
40.17 dmso
40.09 dmso
40.00 dmso
39.93 dmso
39.83 dmso
39.66 dmso
39.50 dmso
27.97
24.09
23.81
21.34
H3C
O
NH2
O
O
N+
O-
NH
N
CH3
H3C
CH3
(4j)
230 220 210 200 190 180 170 160 150 140 130 120 110 100 90 80 70 60 50 40 30 20 10 0 -10
f1 (ppm)

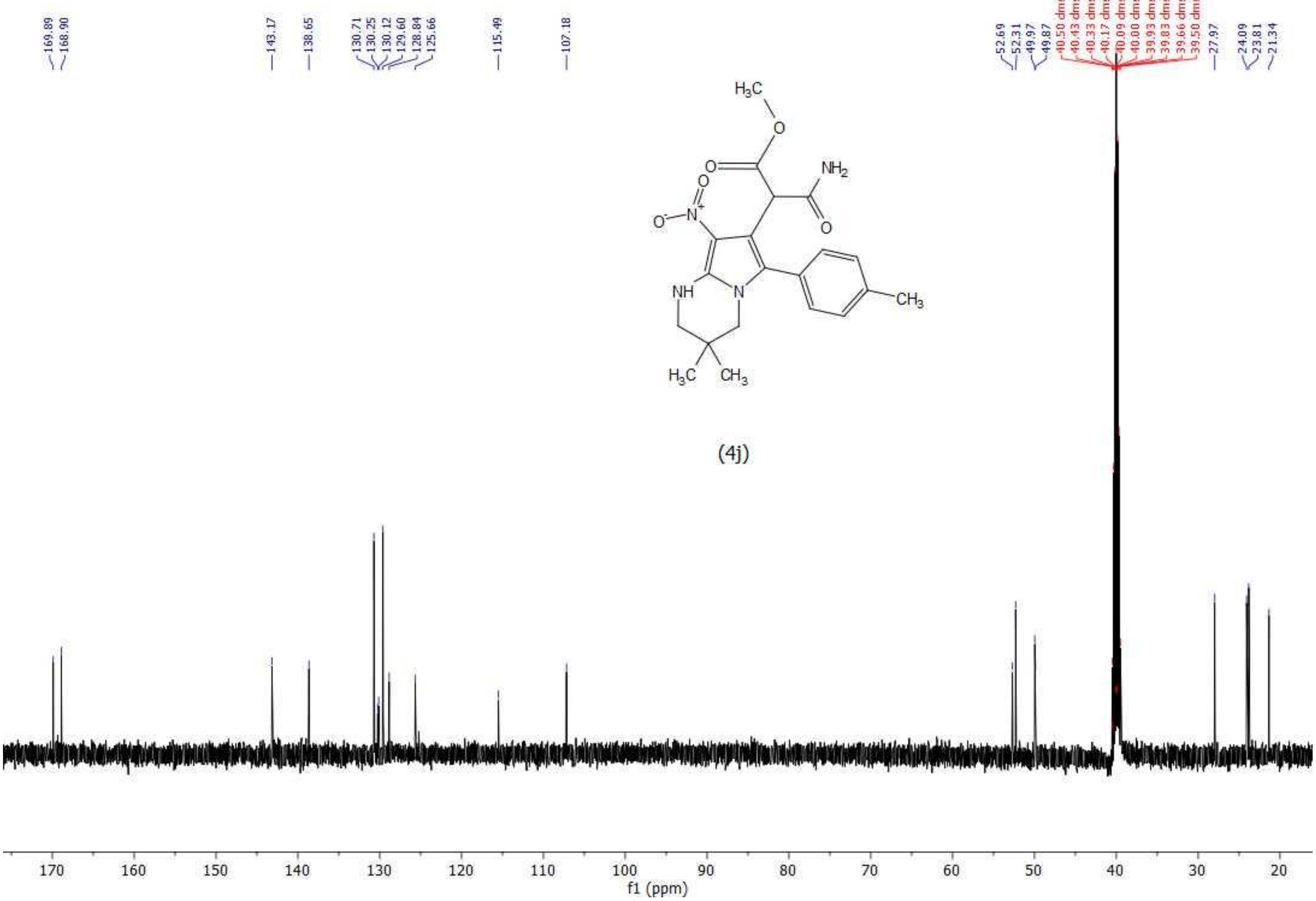
169.89
168.90
143.17
138.65
130.71
130.25
130.12
129.60
128.84
125.66
115.49
107.18
52.69
52.31
49.97
49.87
40.50 dmso
40.43 dmso
40.33 dmso
40.17 dmso
40.09 dmso
40.00 dmso
39.93 dmso
39.83 dmso
39.66 dmso
39.50 dmso
27.97
24.09
23.81
21.34
H_3C
O
O
NH_2
O
O^-
N^+
O
NH
N
CH_3
H_3C
CH_3
170
160
150
140
130
120
110
100
90
80
70
60
50
40
30
20
f1 (ppm)

(4j)

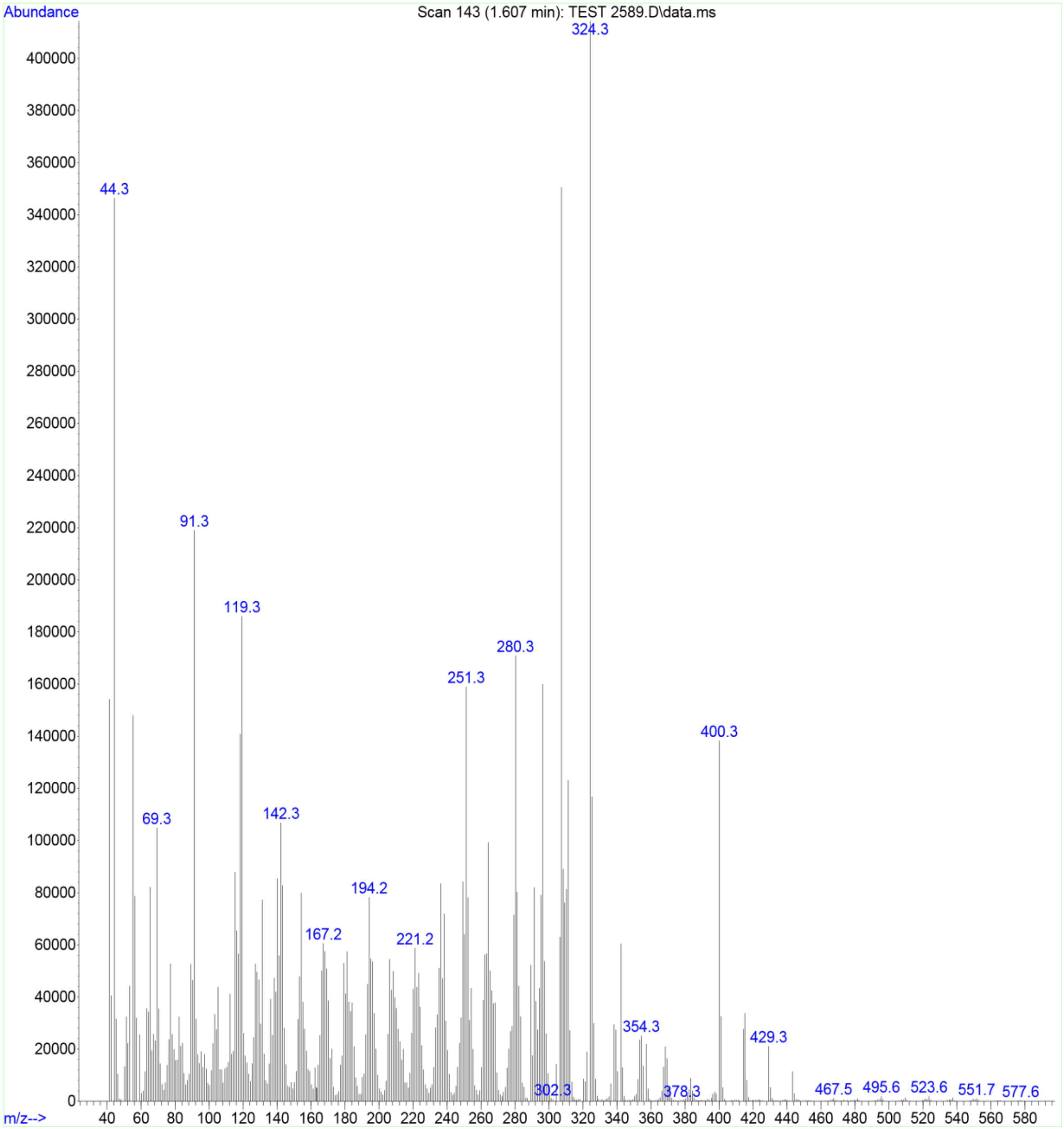

$C_{20}H_{24}N_4O_5$

(400.4)

(4j)

4k-H

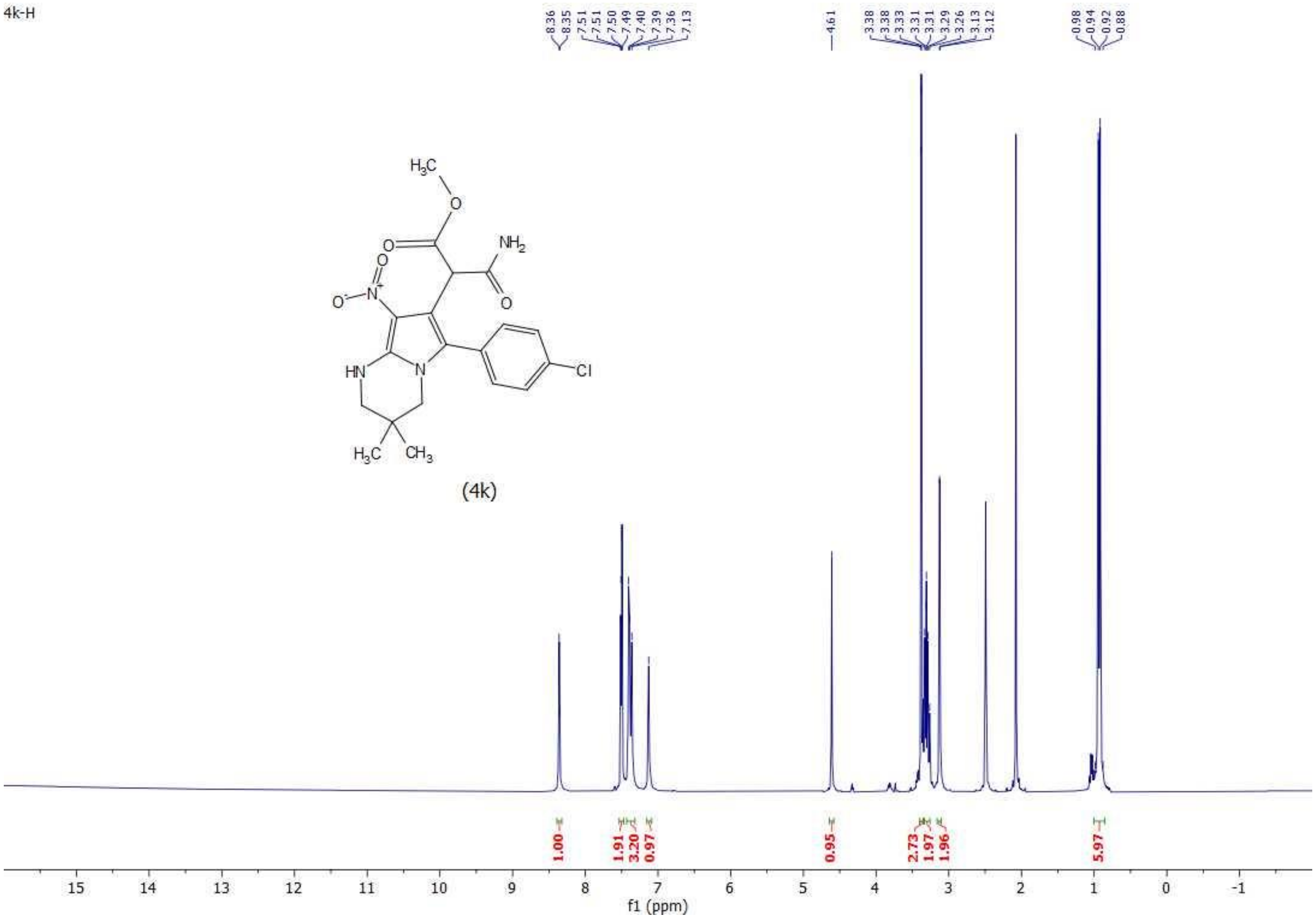
8.36
8.35
7.51
7.51
7.50
7.49
7.40
7.39
7.36
7.13
4.61
3.38
3.38
3.33
3.31
3.31
3.29
3.26
3.13
3.12
0.98
0.94
0.92
0.88
H3C
O
O
NH2
O
N+
O-
O
HN
N
Cl
H3C
CH3
(4k)
1.00
1.91
3.20
0.97
0.95
2.73
1.97
1.96
5.97
15
14
13
12
11
10
9
8
7
6
5
4
3
2
1
0
-1
f1 (ppm)

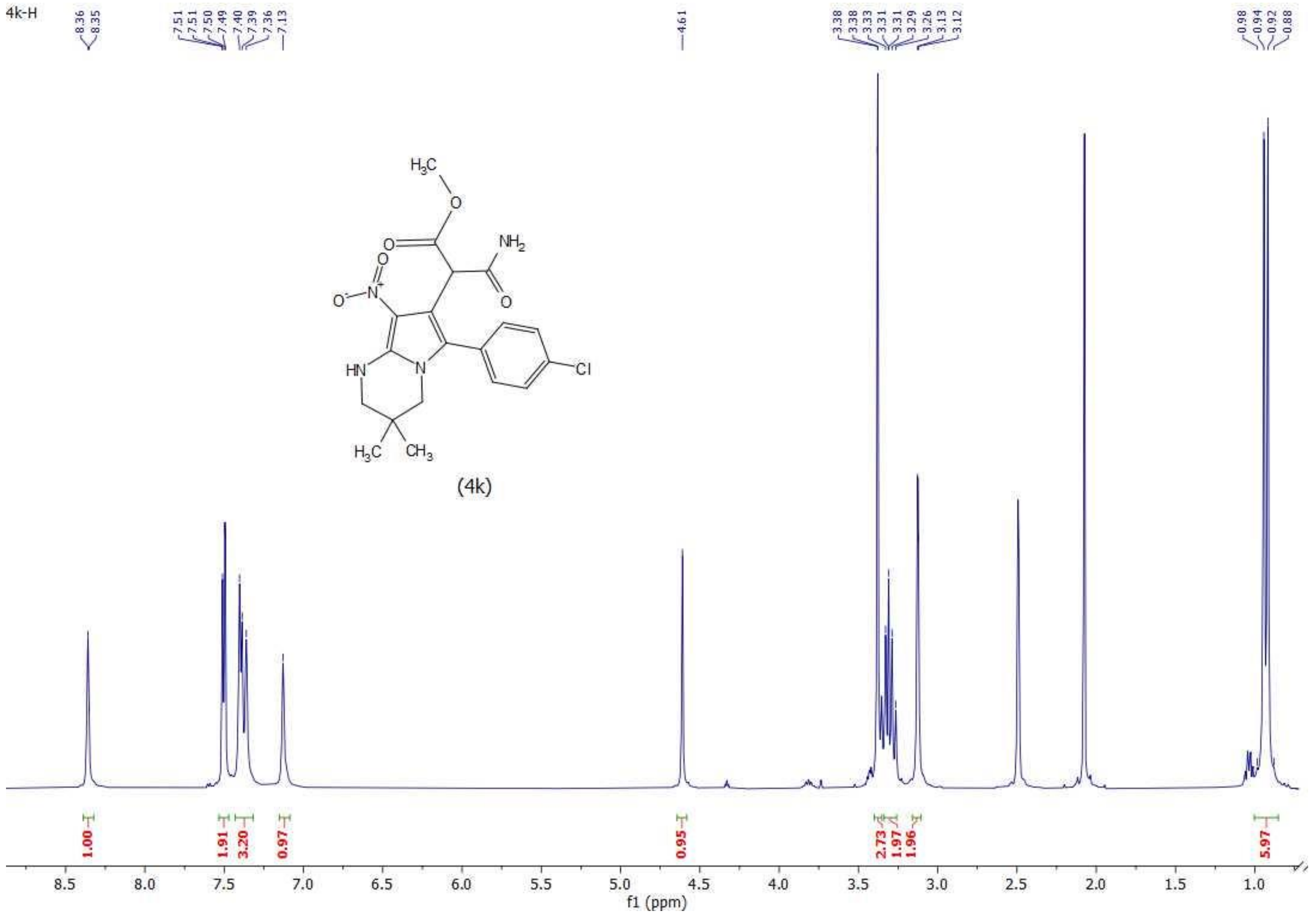
4k-H
8.36
8.35
7.51
7.51
7.50
7.49
7.40
7.39
7.36
7.13
4.61
3.38
3.38
3.33
3.31
3.31
3.29
3.26
3.13
3.12
0.98
0.94
0.92
0.88
H_3C
O
O
NH_2
O
O
N^+
O^-
HN
N
Cl
H_3C
CH_3
(4k)
1.00
1.91
3.20
0.97
0.95
2.73
1.97
1.96
5.97
8.5
8.0
7.5
7.0
6.5
6.0
5.5
5.0
4.5
4.0
3.5
3.0
2.5
2.0
1.5
1.0
f1 (ppm)

C4j

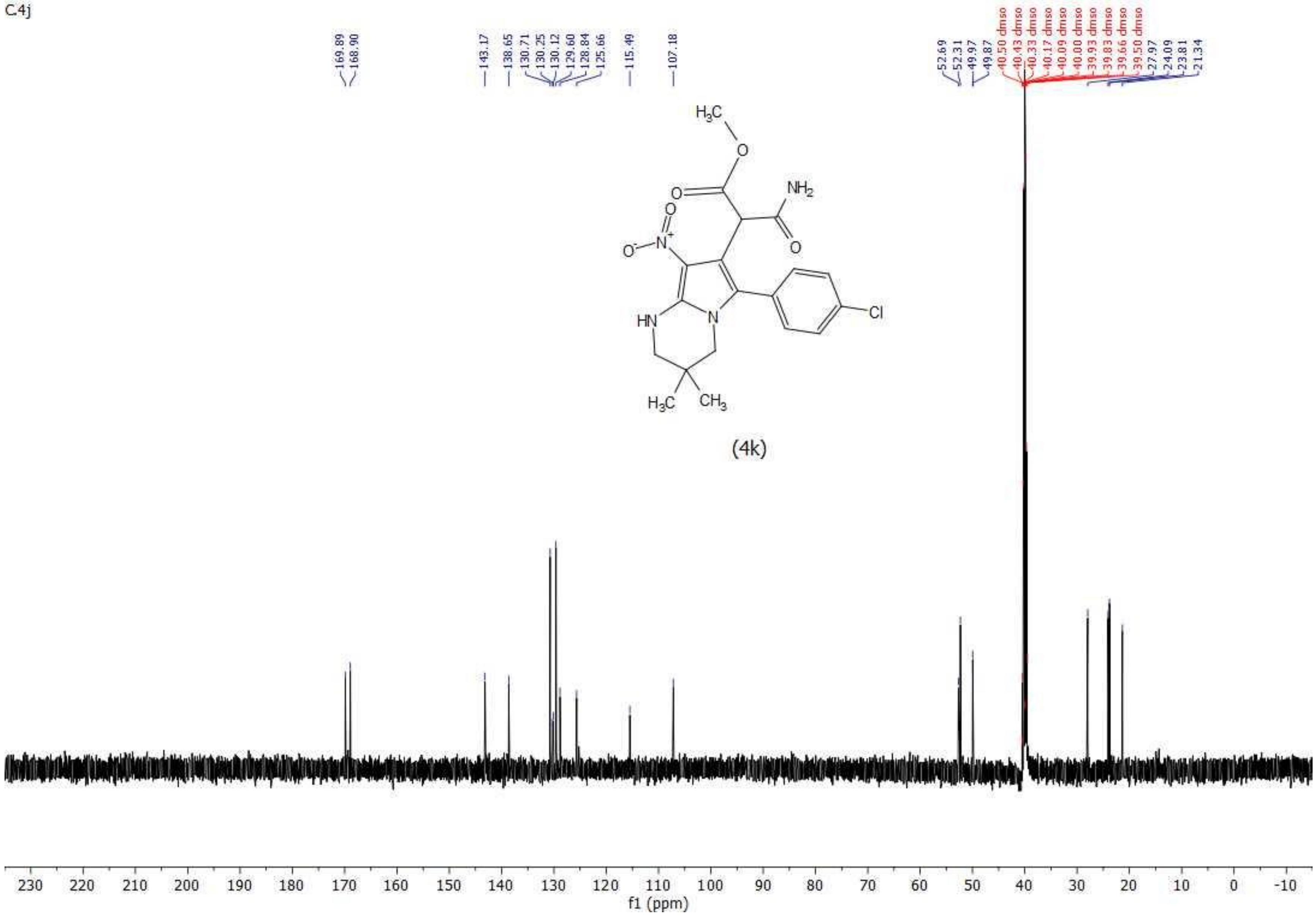
169.89
168.90
143.17
138.65
130.71
130.25
130.12
129.60
128.84
125.66
115.49
107.18
52.69
52.31
49.97
49.87
40.50 dmso
40.43 dmso
40.33 dmso
40.17 dmso
40.09 dmso
40.00 dmso
39.93 dmso
39.83 dmso
39.66 dmso
39.50 dmso
27.97
24.09
23.81
21.34
H_3C
O
O
NH_2
O
N^+
O^-
O
HN
N
Cl
H_3C
CH_3
230 220 210 200 190 180 170 160 150 140 130 120 110 100 90 80 70 60 50 40 30 20 10 0 -10
f1 (ppm)

(4k)

C4j

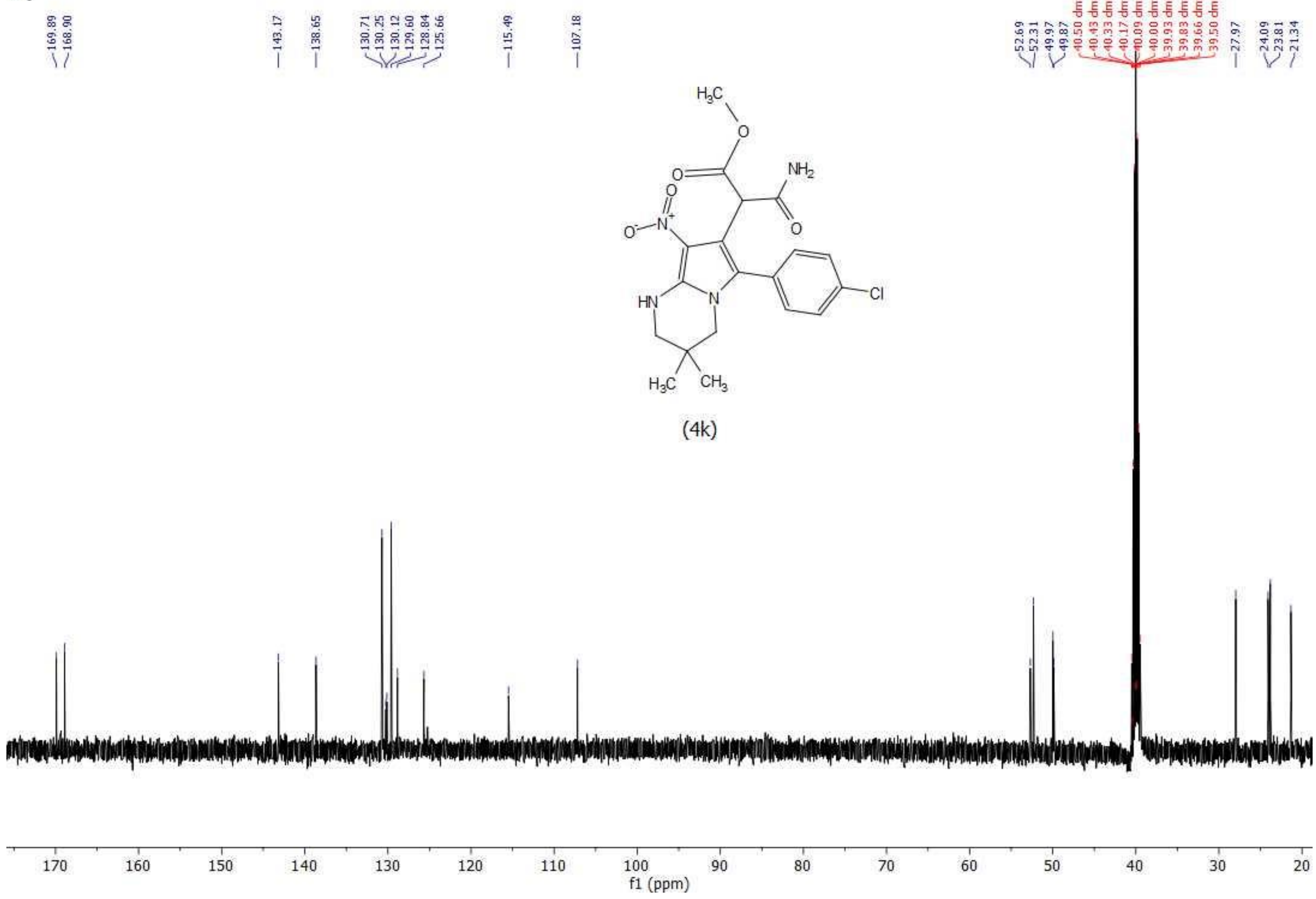
169.89
168.90
143.17
138.65
130.71
130.25
130.12
129.60
128.84
125.66
115.49
107.18
52.69
52.31
49.97
49.87
40.50 dmso
40.43 dmso
40.33 dmso
40.17 dmso
40.09 dmso
40.00 dmso
39.93 dmso
39.83 dmso
39.66 dmso
39.50 dmso
27.97
24.09
23.81
21.34
H_3C
O
NH_2
O
O
O^-
N^+
O
HN
N
Cl
H_3C
CH_3
(4k)
170
160
150
140
130
120
110
100
90
80
70
60
50
40
30
20
f1 (ppm)

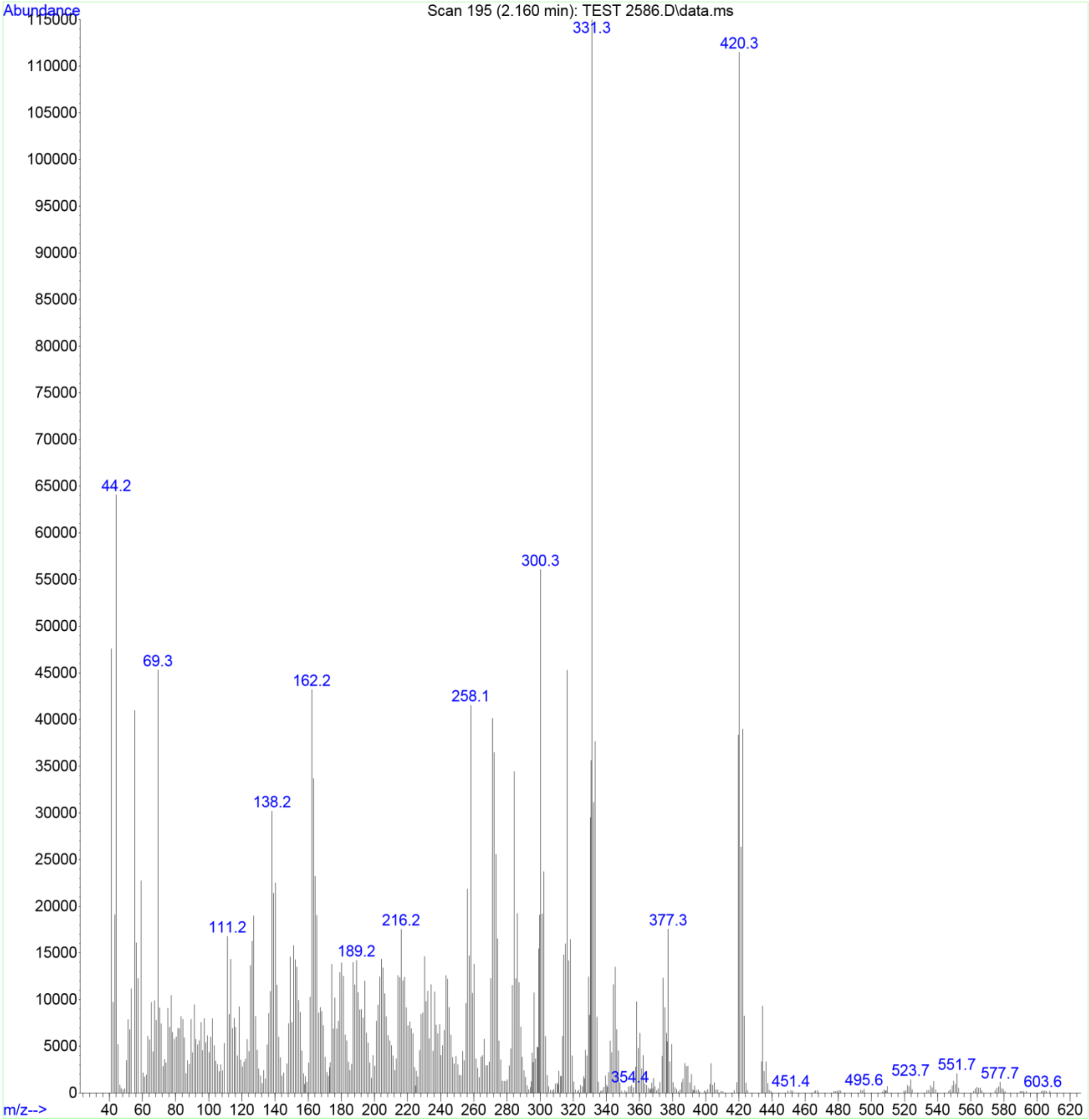

$C_{19}H_{21}ClN_4O_5$

(420/85)

(4k)

H.4l

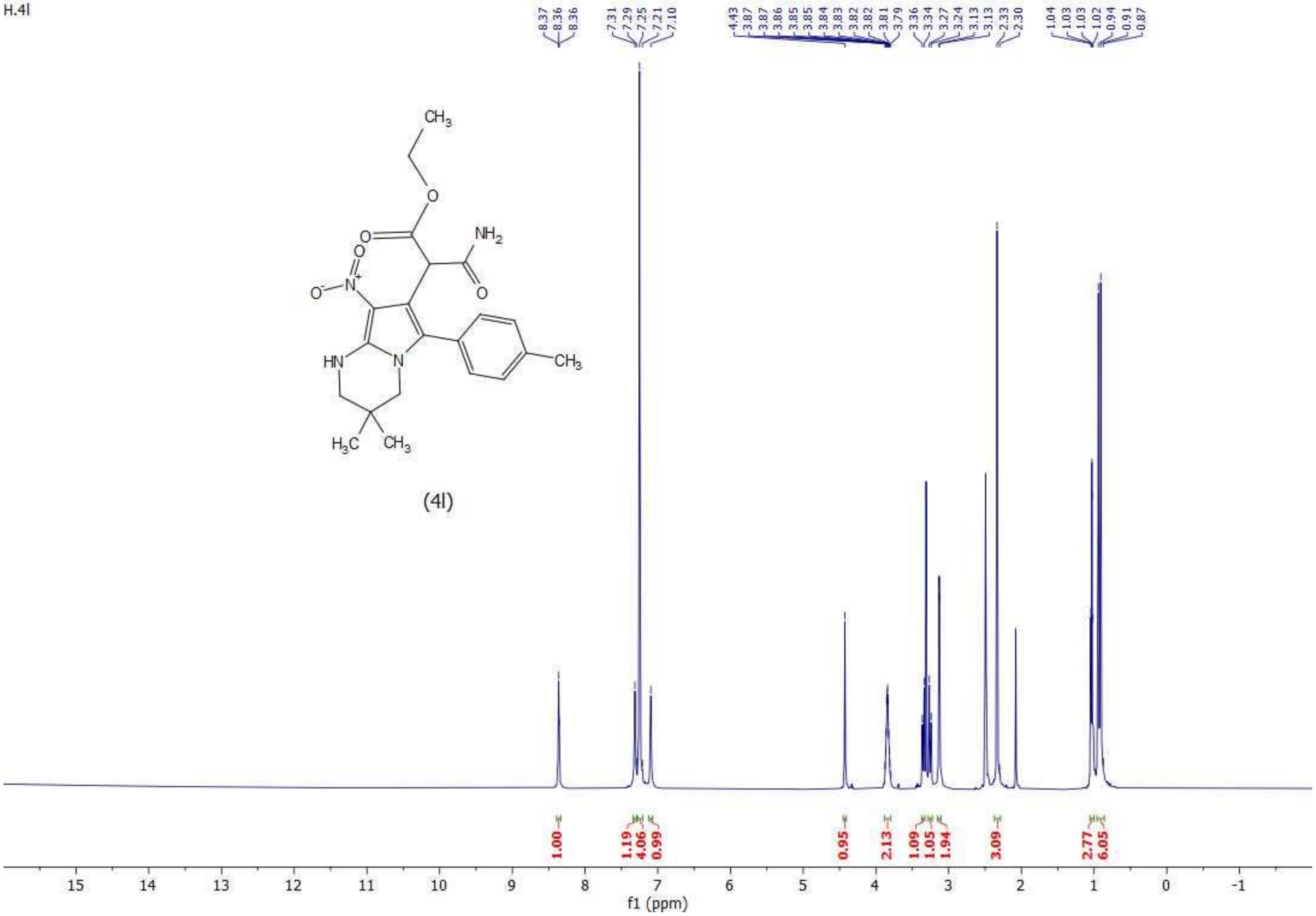
8.37 8.36 8.36
7.31 7.29 7.25 7.21 7.10
4.43 3.87 3.87 3.86 3.85 3.85 3.84 3.83 3.82 3.82 3.81 3.79
3.36 3.34 3.27 3.24 3.13 3.13 2.33 2.30
1.04 1.03 1.03 1.02 0.94 0.91 0.87
CH3
O
NH2
O
N+
O-
HN
N
CH3
H3C
CH3
(4l)
1.00
1.19 4.06 0.99
0.95
2.13
1.09 1.05 1.94
3.09
2.77 6.05
15 14 13 12 11 10 9 8 7 6 5 4 3 2 1 0 -1
f1 (ppm)

H.4l

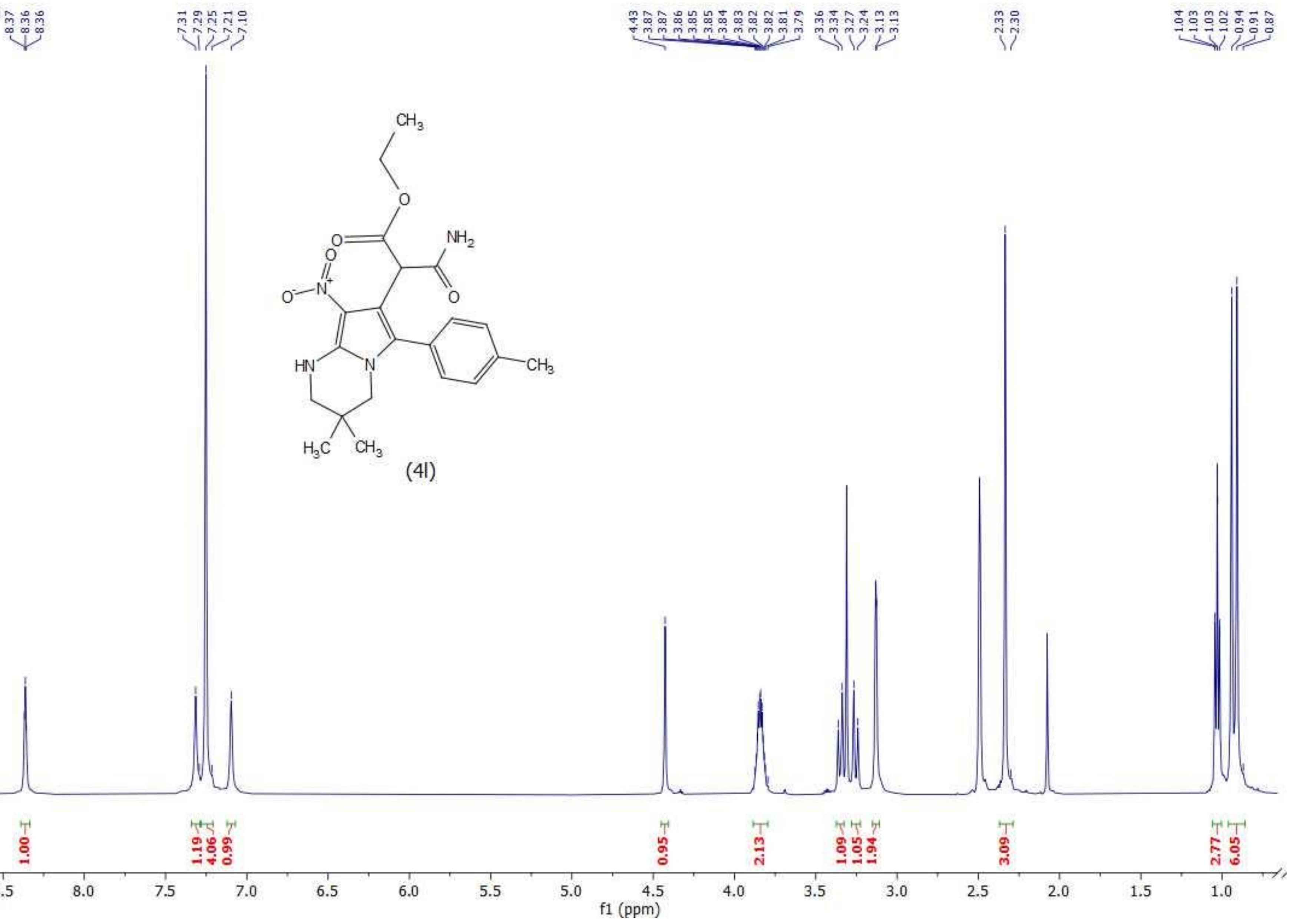
8.37
8.36
8.36
7.31
7.29
7.25
7.21
7.10
4.43
3.87
3.87
3.86
3.85
3.85
3.84
3.83
3.82
3.82
3.81
3.79
3.36
3.34
3.27
3.24
3.13
3.13
2.33
2.30
1.04
1.03
1.03
1.02
0.94
0.91
0.87
(4l)
1.00
1.19
4.06
0.99
0.95
2.13
1.09
1.05
1.94
3.09
2.77
6.05
8.5
8.0
7.5
7.0
6.5
6.0
5.5
5.0
4.5
4.0
3.5
3.0
2.5
2.0
1.5
1.0
f1 (ppm)

C4l

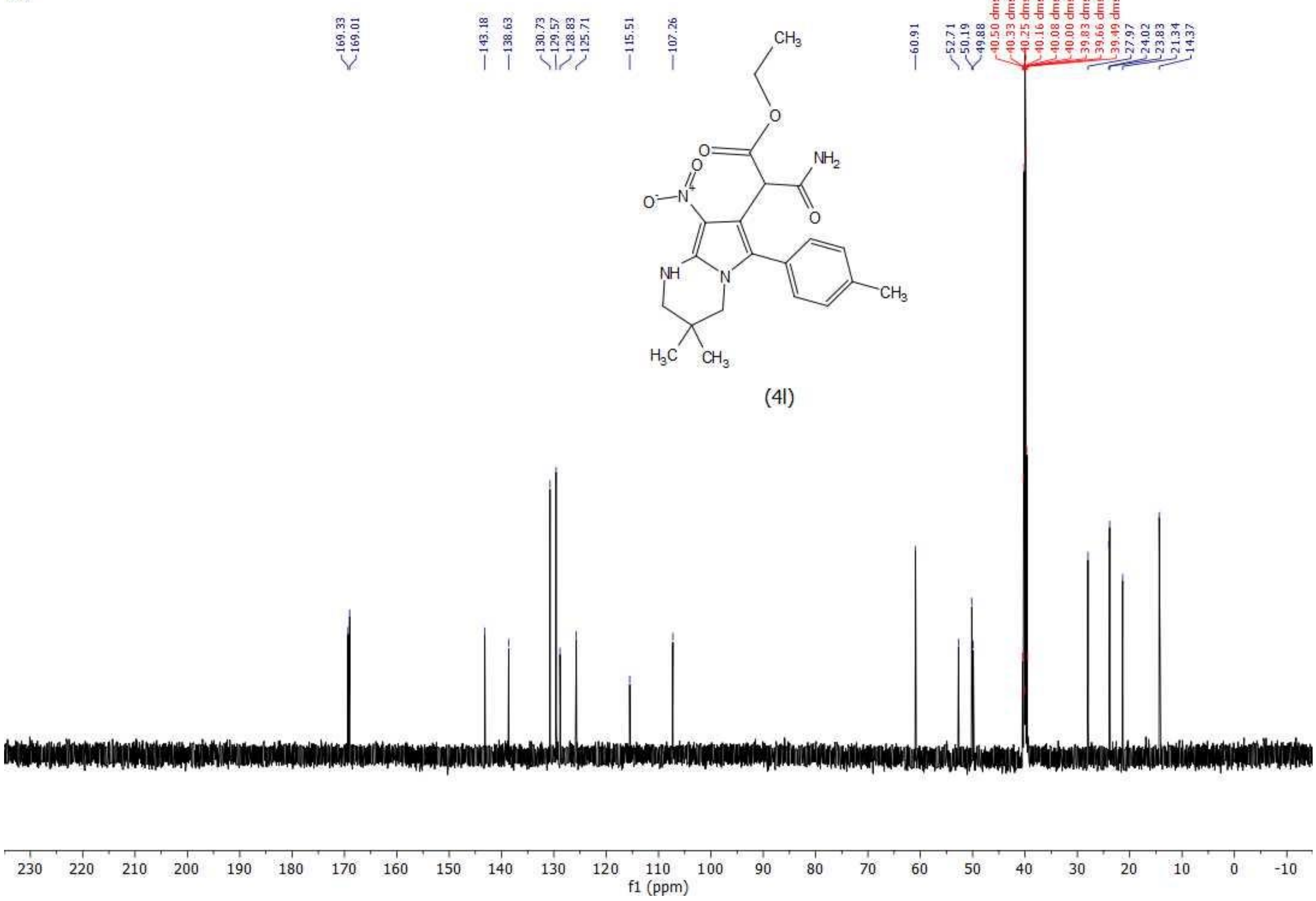
169.33
169.01
143.18
138.63
130.73
129.57
128.83
125.71
115.51
107.26
60.91
52.71
50.19
49.88
40.50 dmso
40.33 dmso
40.25 dmso
40.16 dmso
40.08 dmso
40.00 dmso
39.83 dmso
39.66 dmso
39.49 dmso
27.97
24.02
23.83
21.34
14.37
(4l)
f1 (ppm)

H.4m

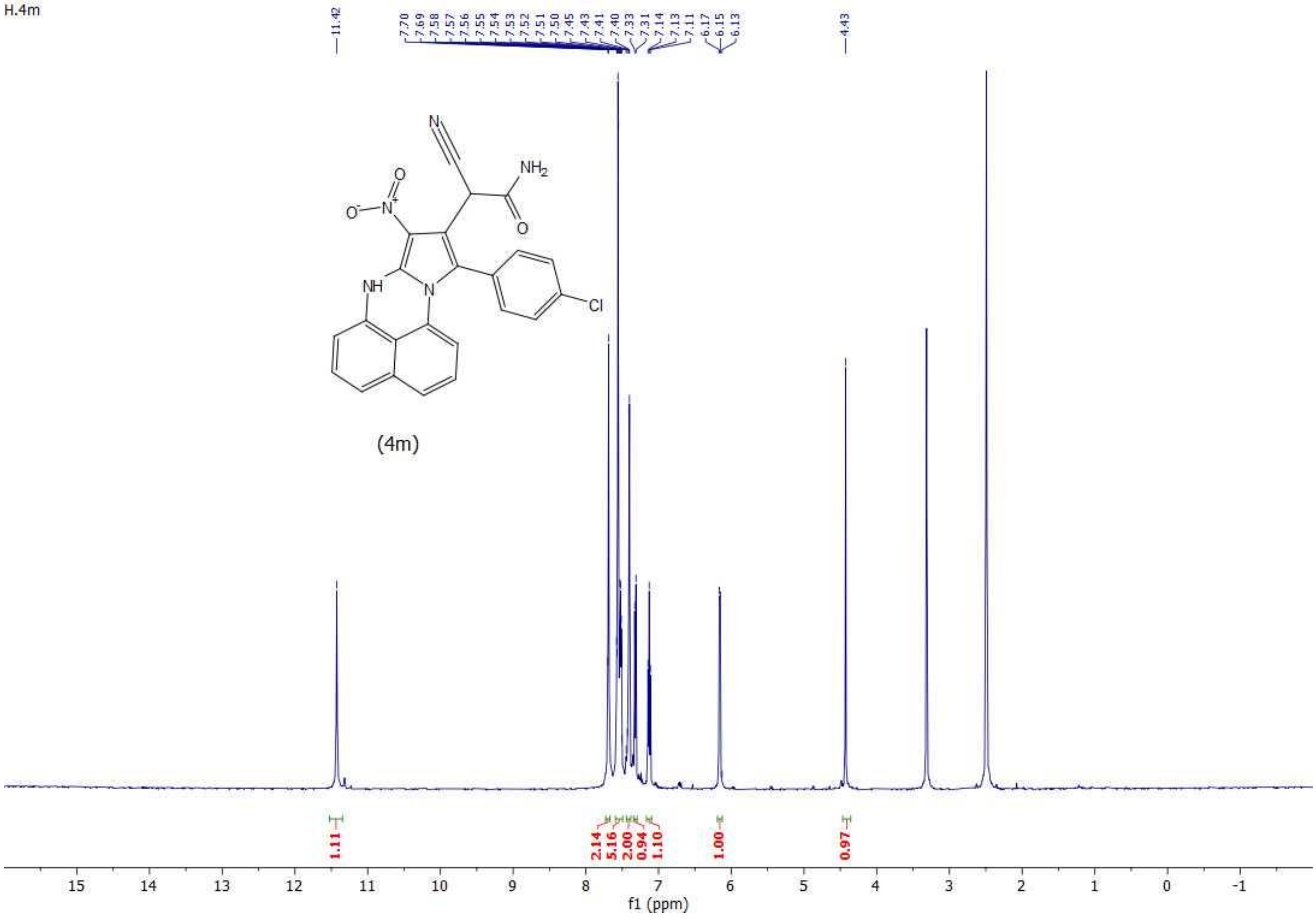
11.42
7.70 7.69 7.58 7.57 7.56 7.55 7.54 7.53 7.52 7.51 7.50 7.45 7.43 7.41 7.40 7.33 7.31 7.14 7.13 7.11 6.17 6.15 6.13
4.43
NH$_2$
O
N
O$^-$
N$^+$
NH
N
Cl
(4m)
1.11
2.14
5.16
2.00
0.94
1.10
1.00
0.97
15 14 13 12 11 10 9 8 7 6 5 4 3 2 1 0 -1
f1 (ppm)

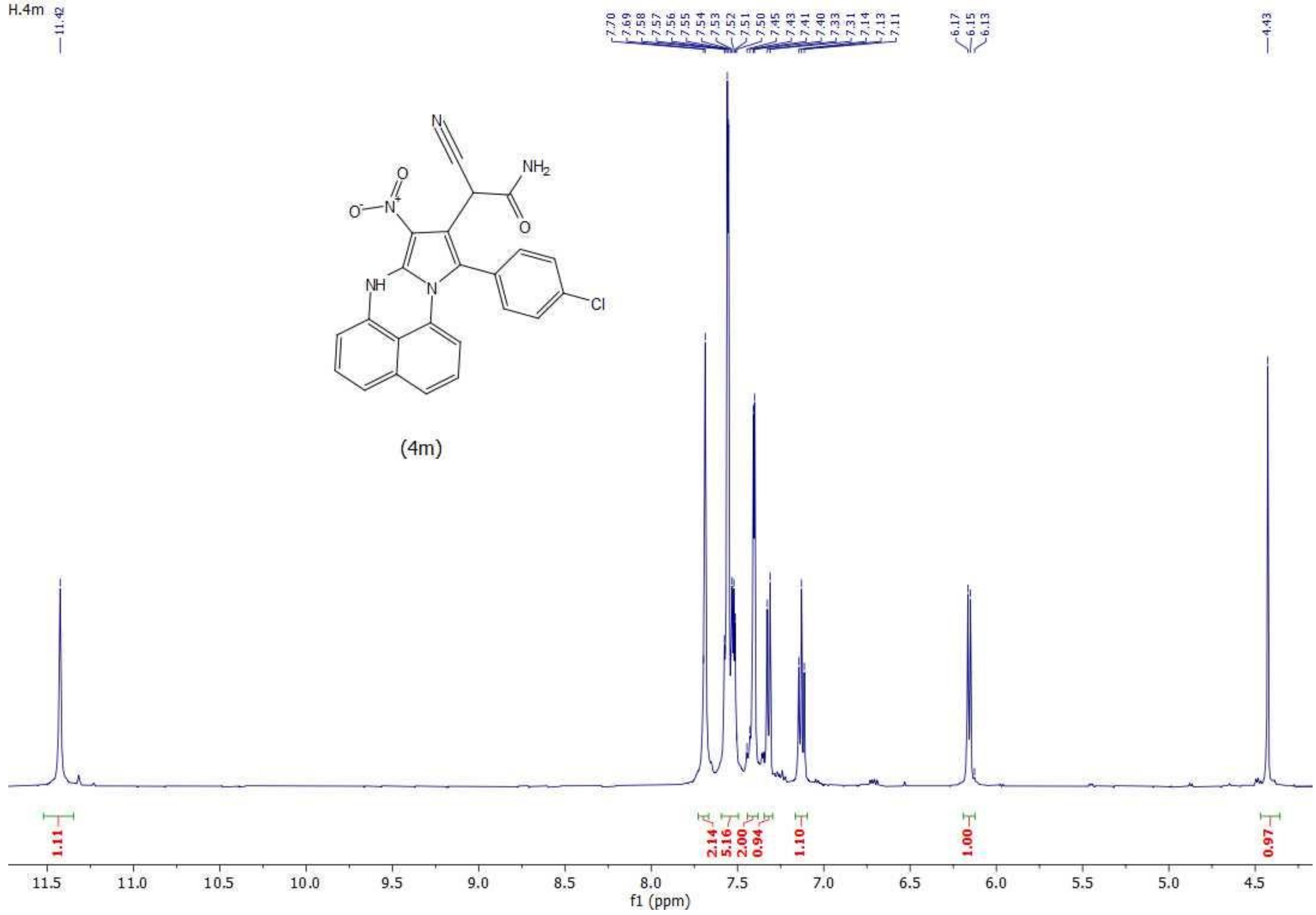
H.4m
11.42
7.70
7.69
7.58
7.57
7.56
7.55
7.54
7.53
7.52
7.51
7.50
7.45
7.43
7.41
7.40
7.33
7.31
7.14
7.13
7.11
6.17
6.15
6.13
4.43
NH$_2$
Cl
(4m)
1.11
2.14
5.16
2.00
0.94
1.10
1.00
0.97
11.5
11.0
10.5
10.0
9.5
9.0
8.5
8.0
7.5
7.0
6.5
6.0
5.5
5.0
4.5
f1 (ppm)

C4m

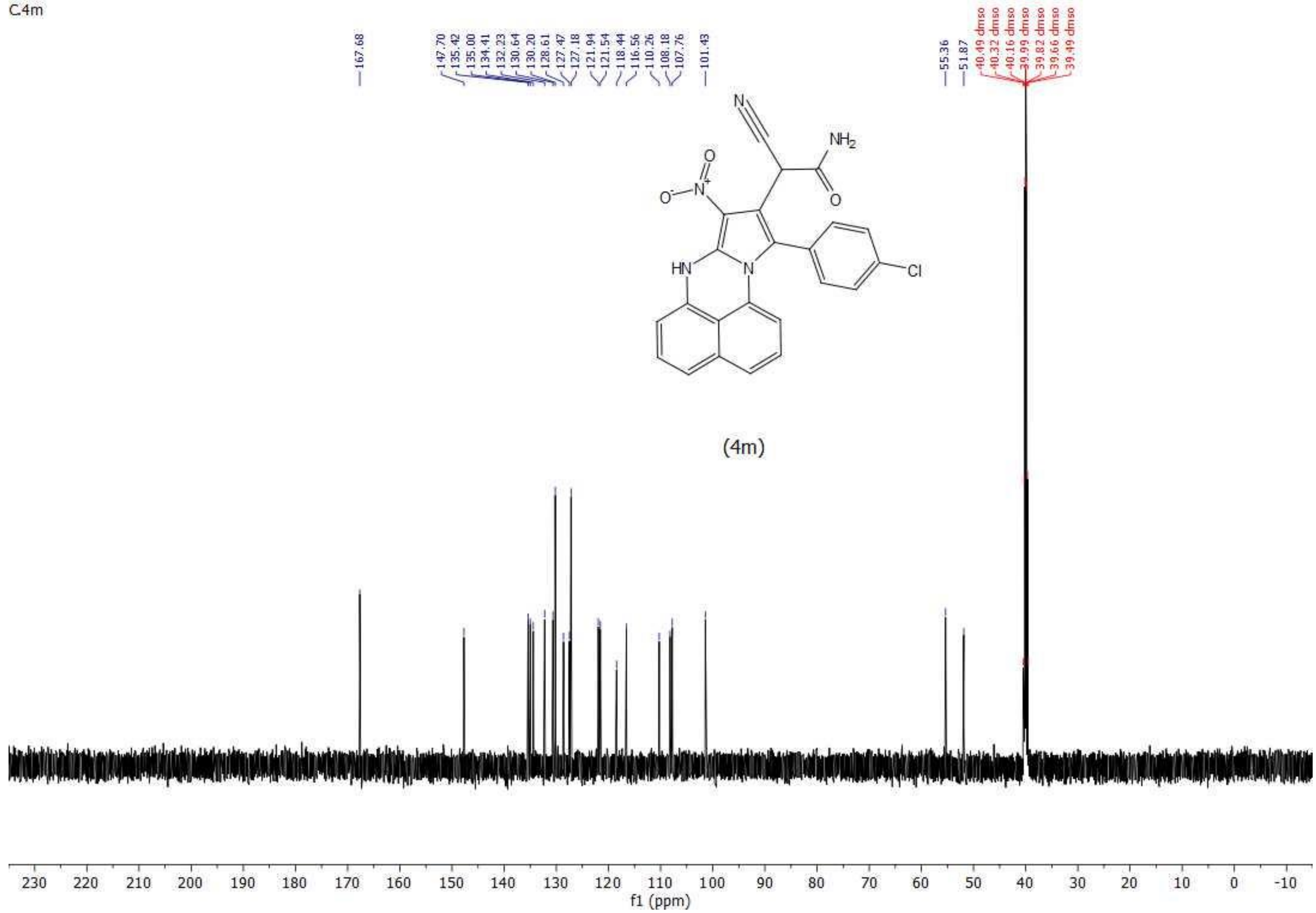

167.68
147.70
135.42
135.00
134.41
132.23
130.64
130.20
128.61
127.47
127.18
121.94
121.54
118.44
116.56
110.26
108.18
107.76
101.43
55.36
51.87
40.49 dmso
40.32 dmso
40.16 dmso
39.99 dmso
39.82 dmso
39.66 dmso
39.49 dmso
NH2
O
N
Cl
HN
(4m)
230 220 210 200 190 180 170 160 150 140 130 120 110 100 90 80 70 60 50 40 30 20 10 0 -10
f1 (ppm)

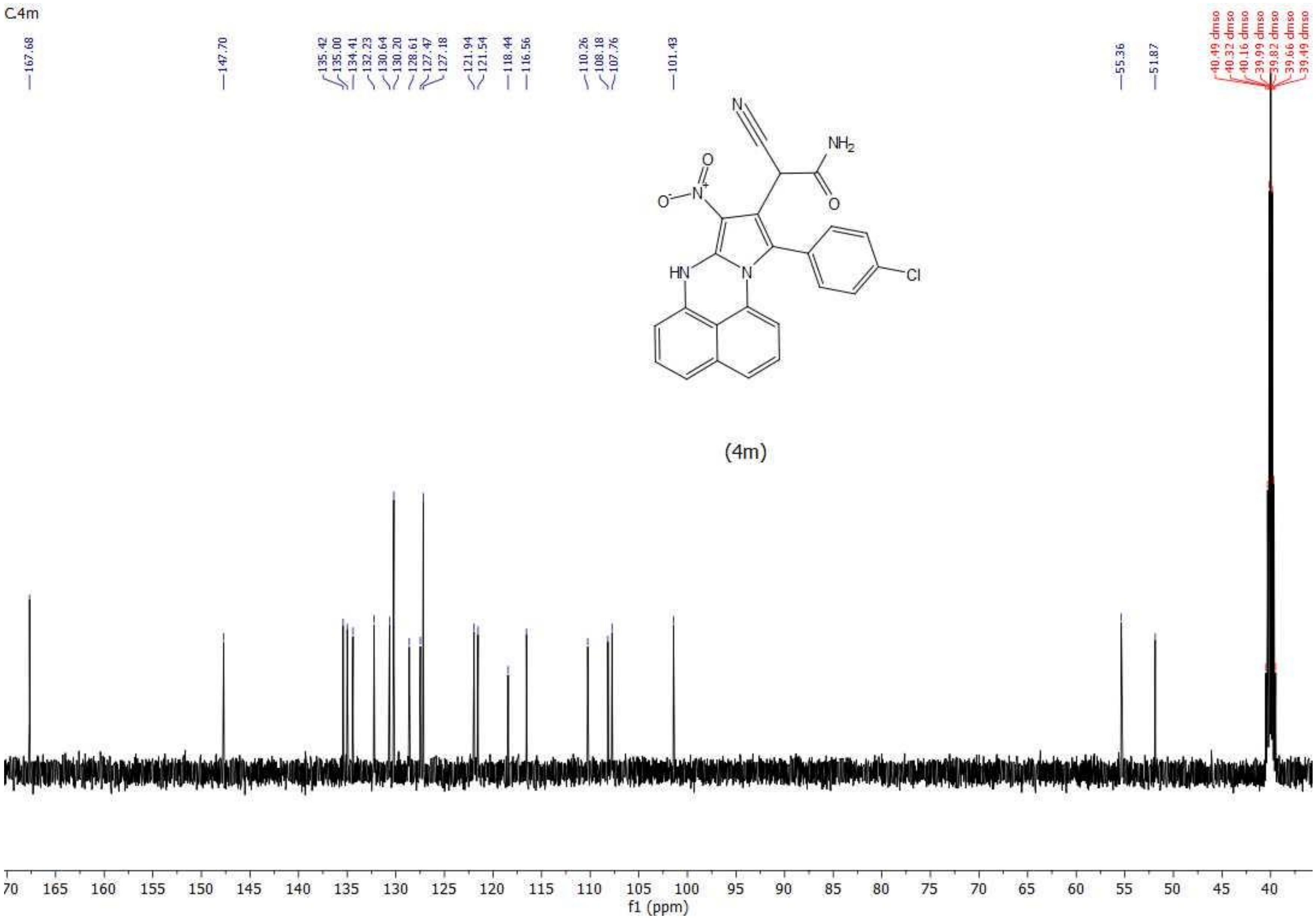
C4m
167.68
147.70
135.42
135.00
134.41
132.23
130.64
130.20
128.61
127.47
127.18
121.94
121.54
118.44
116.56
110.26
108.18
107.76
101.43
55.36
51.87
40.49 dmso
40.32 dmso
40.16 dmso
39.99 dmso
39.82 dmso
39.66 dmso
39.49 dmso
(4m)
f1 (ppm)

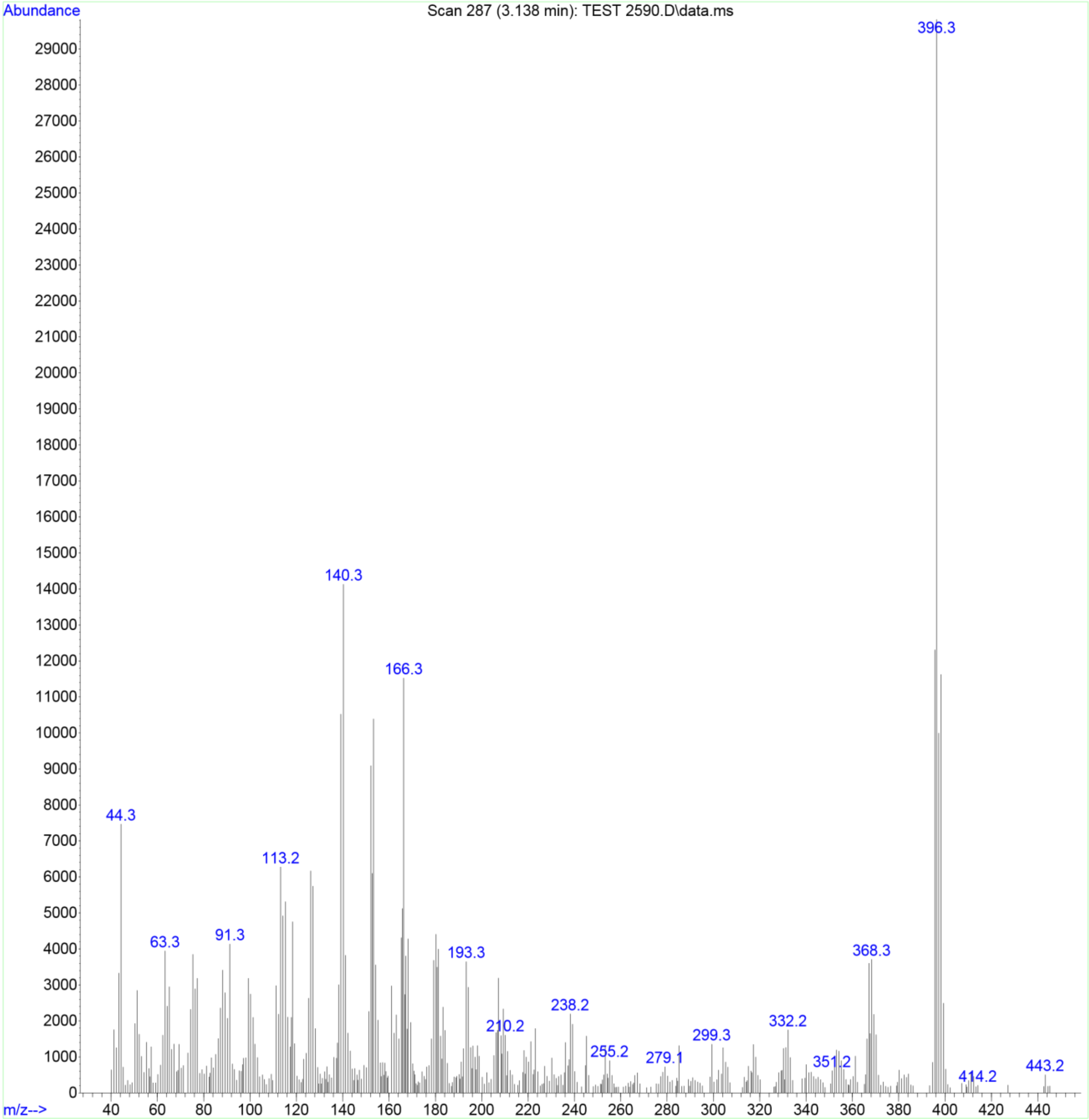

$C_{23}H_{14}ClN_5O_3$

(443/8)

(4m)

H.4n

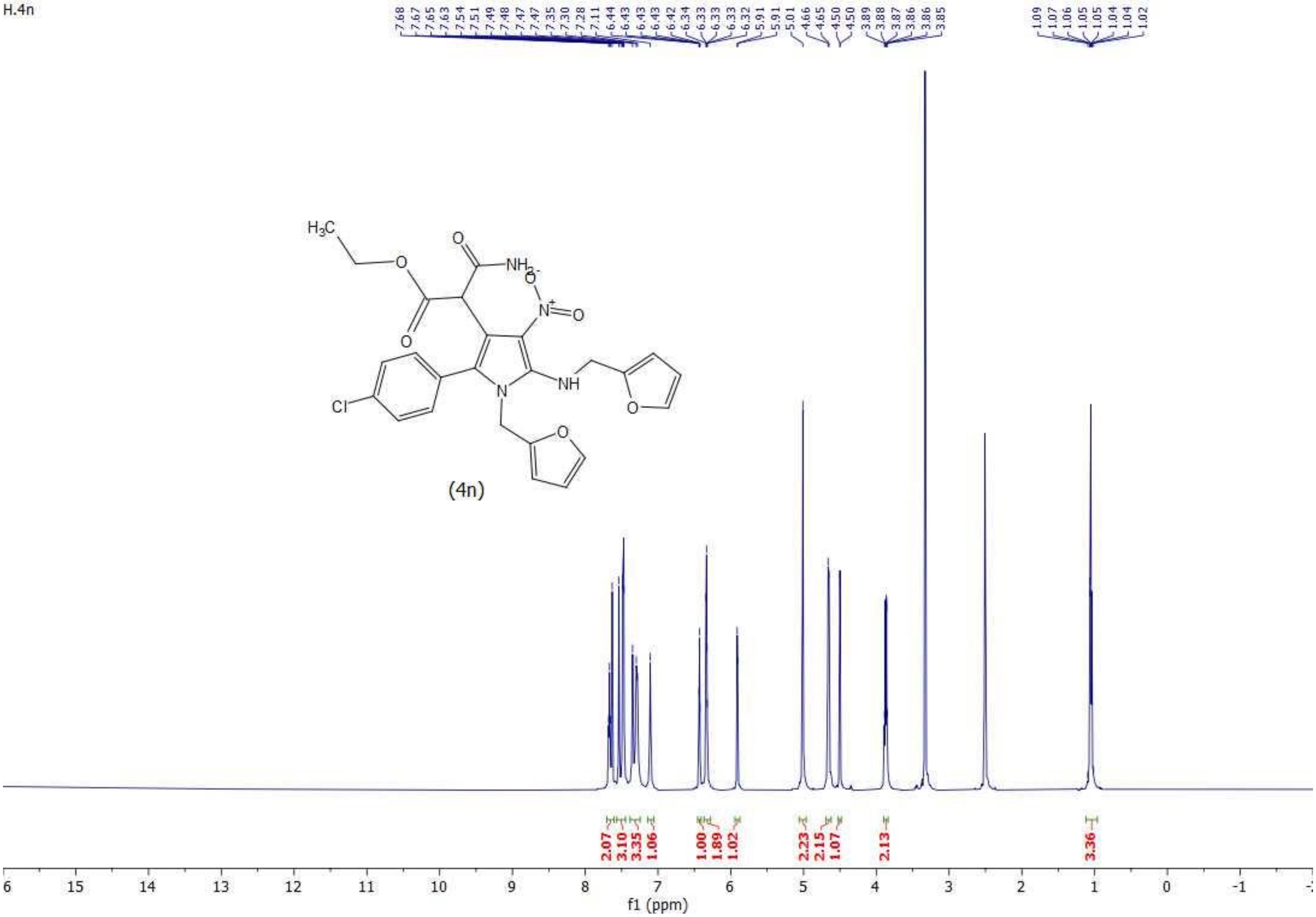
7.68
7.67
7.65
7.63
7.54
7.51
7.49
7.48
7.47
7.47
7.35
7.30
7.28
7.11
6.44
6.43
6.43
6.43
6.42
6.34
6.33
6.33
6.33
6.32
5.91
5.91
5.01
4.66
4.65
4.50
4.50
3.89
3.88
3.87
3.86
3.86
3.85
1.09
1.07
1.06
1.05
1.05
1.04
1.04
1.02
(4n)
2.07
3.10
3.35
1.06
1.00
1.89
1.02
2.23
2.15
1.07
2.13
3.36
f1 (ppm)

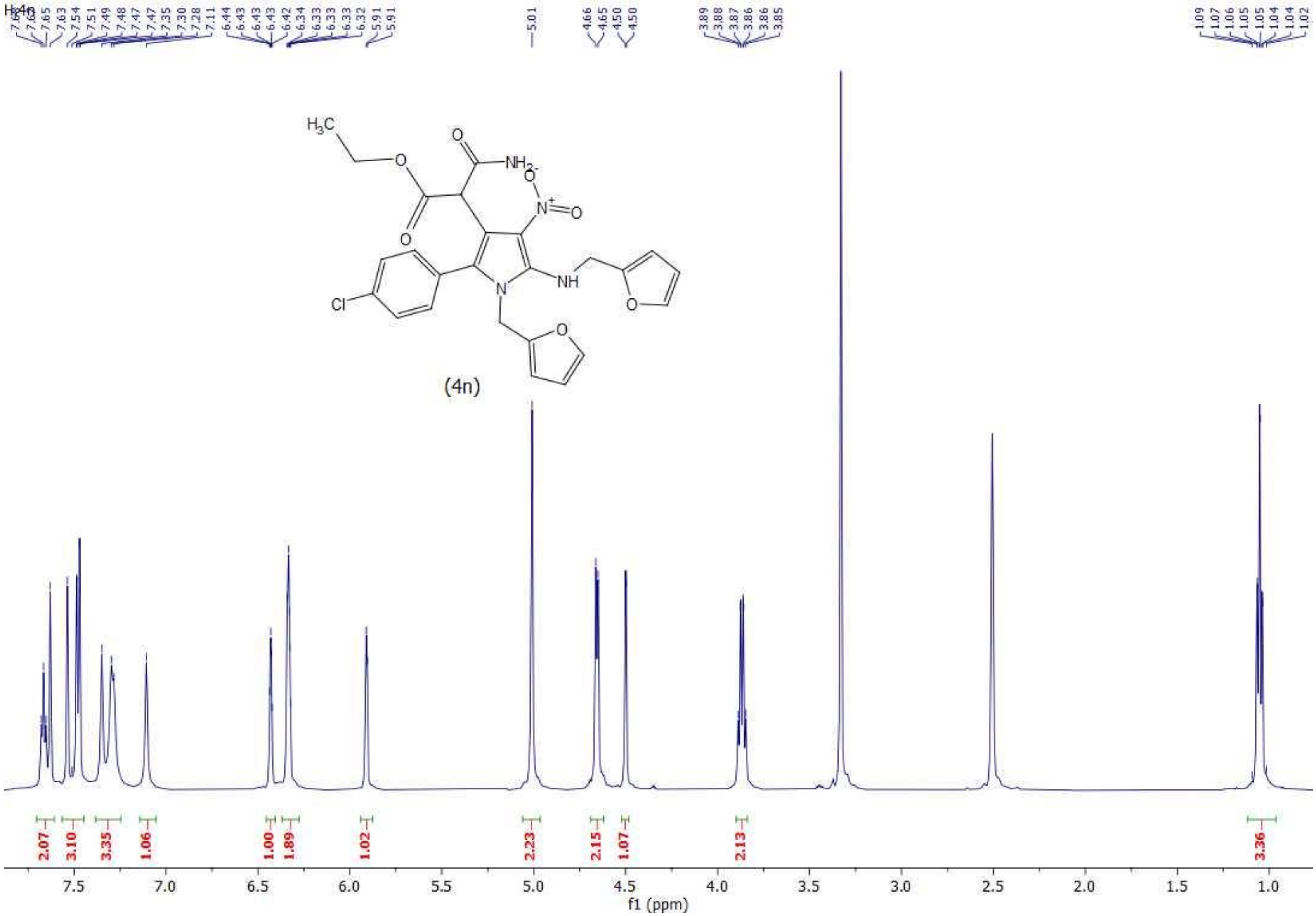
H-4n
7.65
7.63
7.54
7.51
7.49
7.48
7.47
7.47
7.35
7.30
7.28
7.11
6.44
6.43
6.43
6.43
6.42
6.34
6.33
6.33
6.33
6.32
5.91
5.91
5.01
4.66
4.65
4.50
4.50
3.89
3.88
3.87
3.86
3.86
3.85
1.09
1.07
1.06
1.05
1.05
1.04
1.04
1.02
(4n)
2.07
3.10
3.35
1.06
1.00
1.89
1.02
2.23
2.15
1.07
2.13
3.36
7.5
7.0
6.5
6.0
5.5
5.0
4.5
4.0
3.5
3.0
2.5
2.0
1.5
1.0
f1 (ppm)

C4n

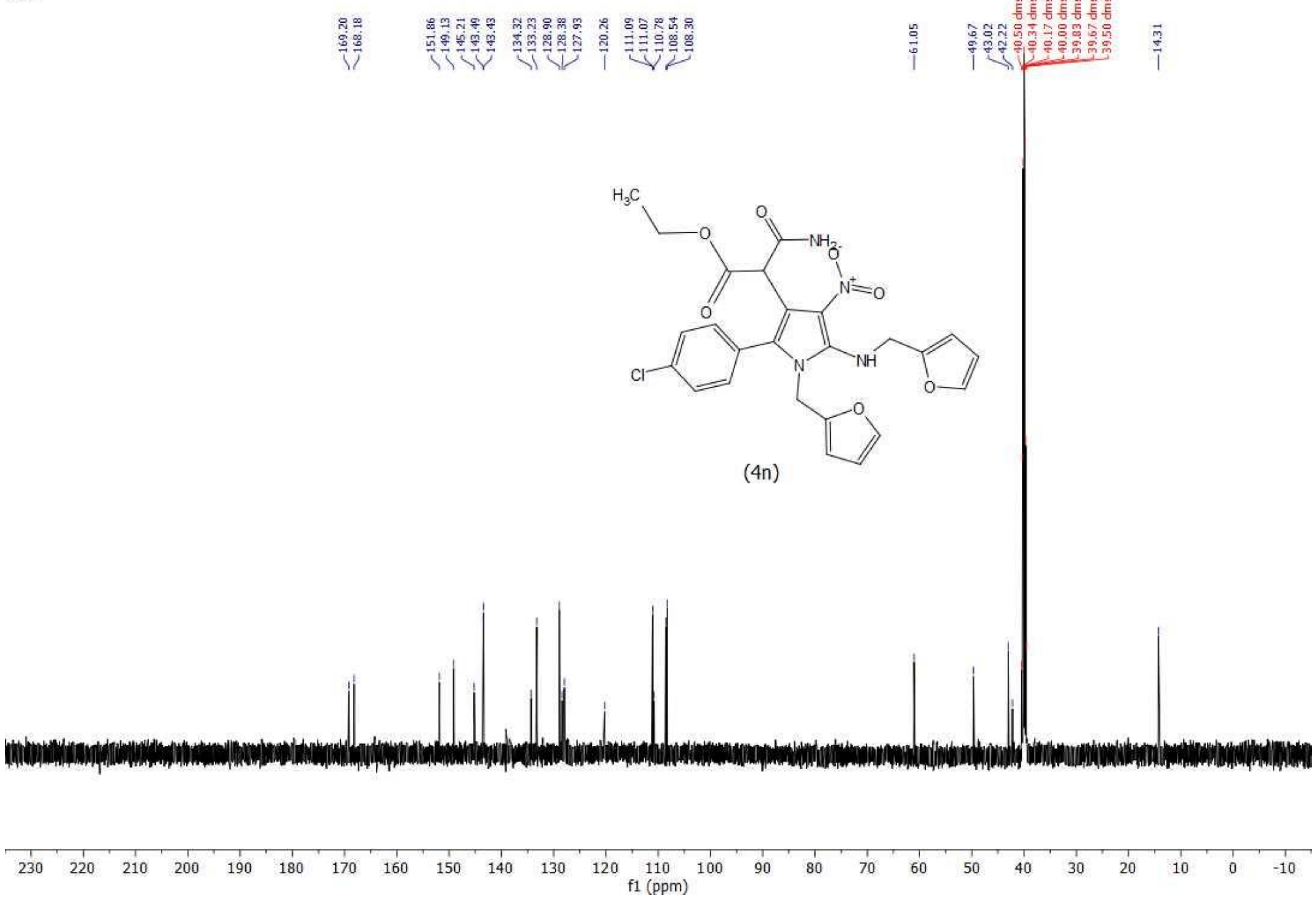
169.20
168.18
151.86
149.13
145.21
143.49
143.43
134.32
133.23
128.90
128.38
127.93
120.26
111.09
111.07
110.78
108.54
108.30
61.05
49.67
43.02
42.22
40.50 dmso
40.34 dmso
40.17 dmso
40.00 dmso
39.83 dmso
39.67 dmso
39.50 dmso
14.31
(4n)
230 220 210 200 190 180 170 160 150 140 130 120 110 100 90 80 70 60 50 40 30 20 10 0 -10
f1 (ppm)

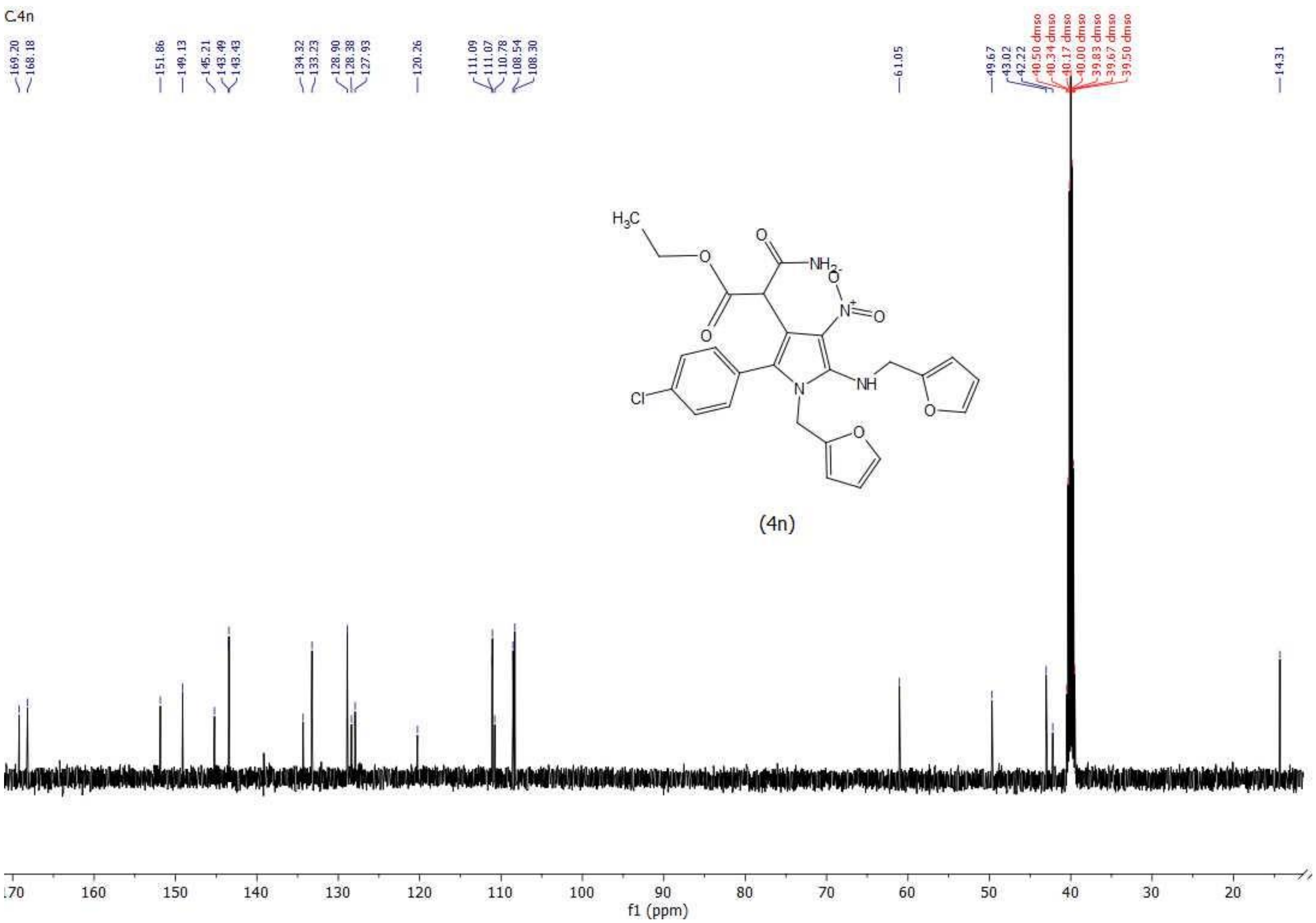
C4n
169.20
168.18
151.86
149.13
145.21
143.49
143.43
134.32
133.23
128.90
128.38
127.93
120.26
111.09
111.07
110.78
108.54
108.30
61.05
49.67
43.02
42.22
40.50 dmso
40.34 dmso
40.17 dmso
40.00 dmso
39.83 dmso
39.67 dmso
39.50 dmso
14.31
(4n)
170
160
150
140
130
120
110
100
90
80
70
60
50
40
30
20
f1 (ppm)

H.4o

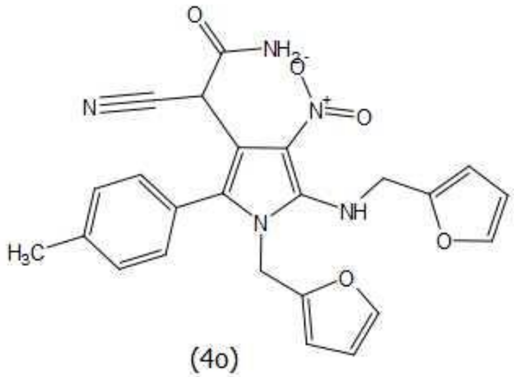

(4o)

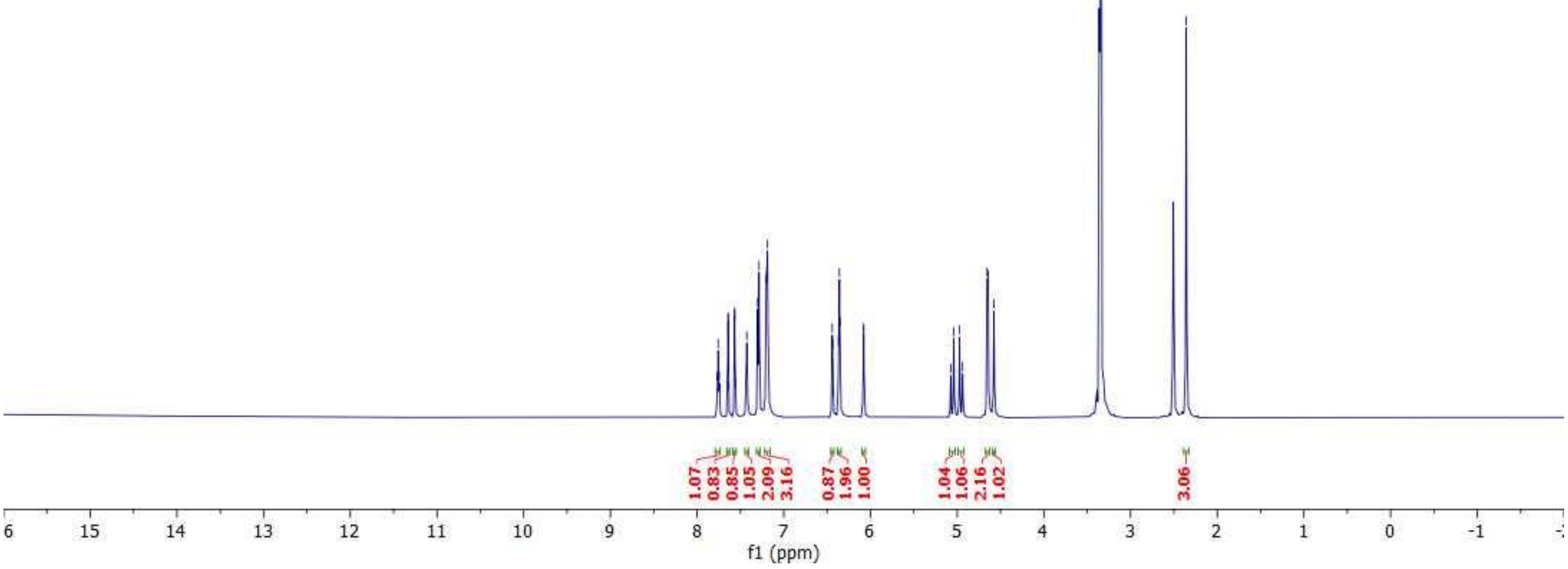
7.77
7.76
7.74
7.64
7.64
7.63
7.57
7.56
7.56
7.43
7.30
7.29
7.20
7.19
6.45
6.44
6.44
6.43
6.37
6.36
6.36
6.35
6.08
6.08
5.07
5.04
4.97
4.94
4.65
4.64
4.58
2.36
1.07
0.83
0.85
1.05
2.09
3.16
0.87
1.96
1.00
1.04
1.06
2.16
1.02
3.06
f1 (ppm)

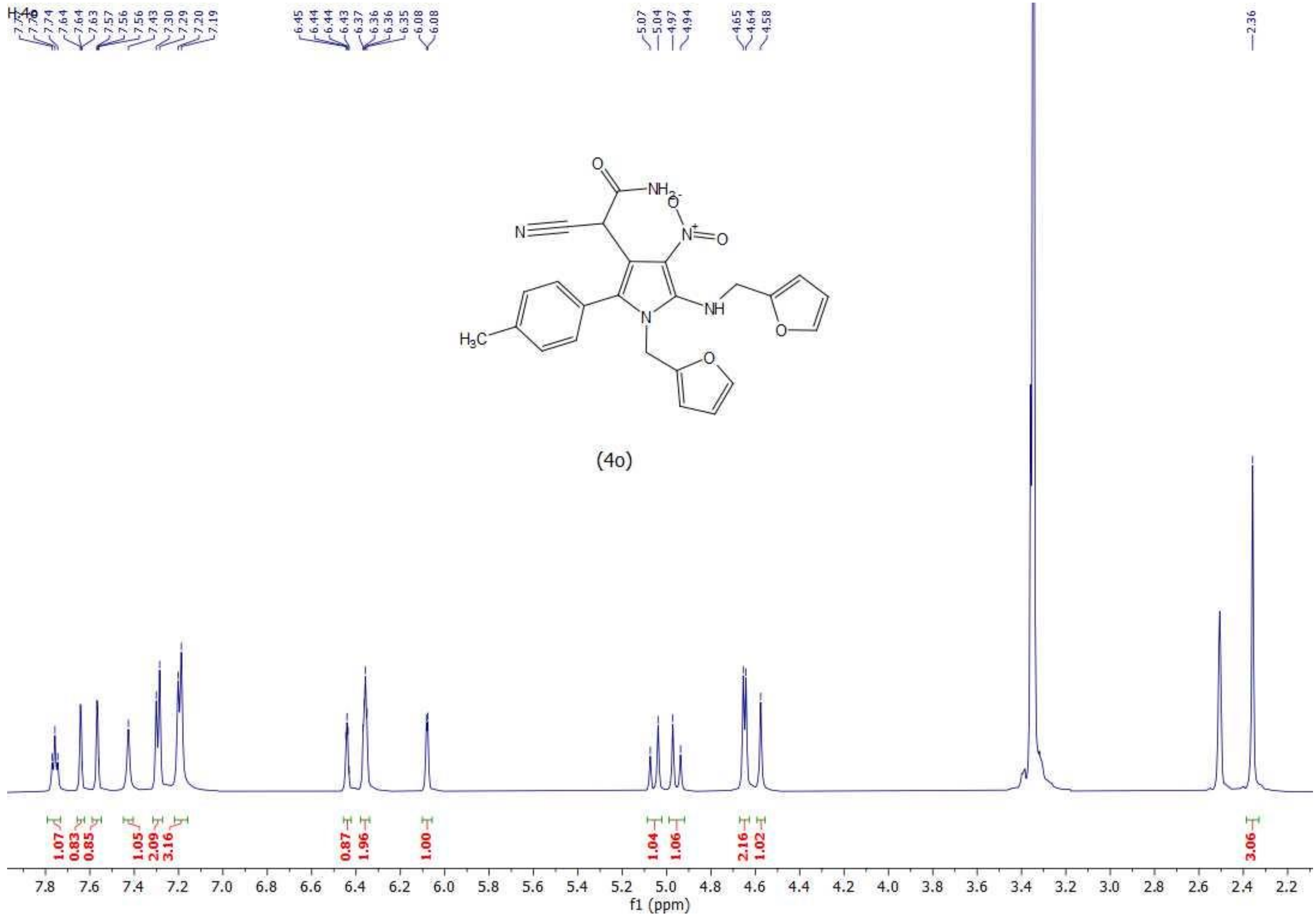
H-4o
7.74
7.64
7.63
7.57
7.56
7.43
7.30
7.29
7.20
7.19
6.45
6.44
6.44
6.43
6.37
6.36
6.36
6.35
6.08
6.08
5.07
5.04
4.97
4.94
4.65
4.64
4.58
2.36
(4o)
1.07
0.83
0.85
1.05
2.09
3.16
0.87
1.96
1.00
1.04
1.06
2.16
1.02
3.06
f1 (ppm)

C4o

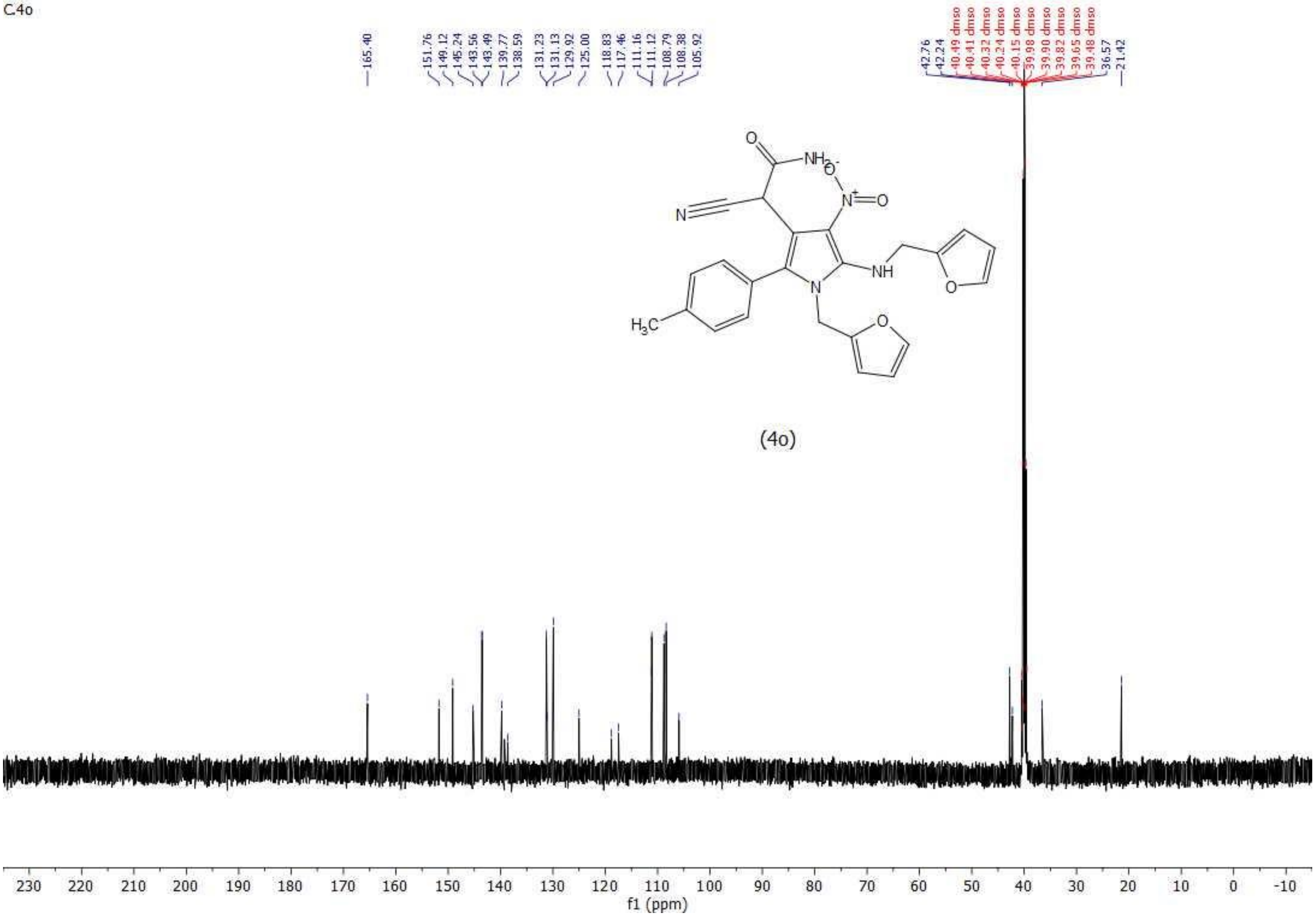
165.40
151.76
149.12
145.24
143.56
143.49
139.77
138.59
131.23
131.13
129.92
125.00
118.83
117.46
111.16
111.12
108.79
108.38
105.92
42.76
42.24
40.49 dmso
40.41 dmso
40.32 dmso
40.24 dmso
40.15 dmso
39.98 dmso
39.90 dmso
39.82 dmso
39.65 dmso
39.48 dmso
36.57
21.42
O
NH2
O-
N+
O
N
NH
N
O
O
H3C
(4o)
230 220 210 200 190 180 170 160 150 140 130 120 110 100 90 80 70 60 50 40 30 20 10 0 -10
f1 (ppm)

C4o

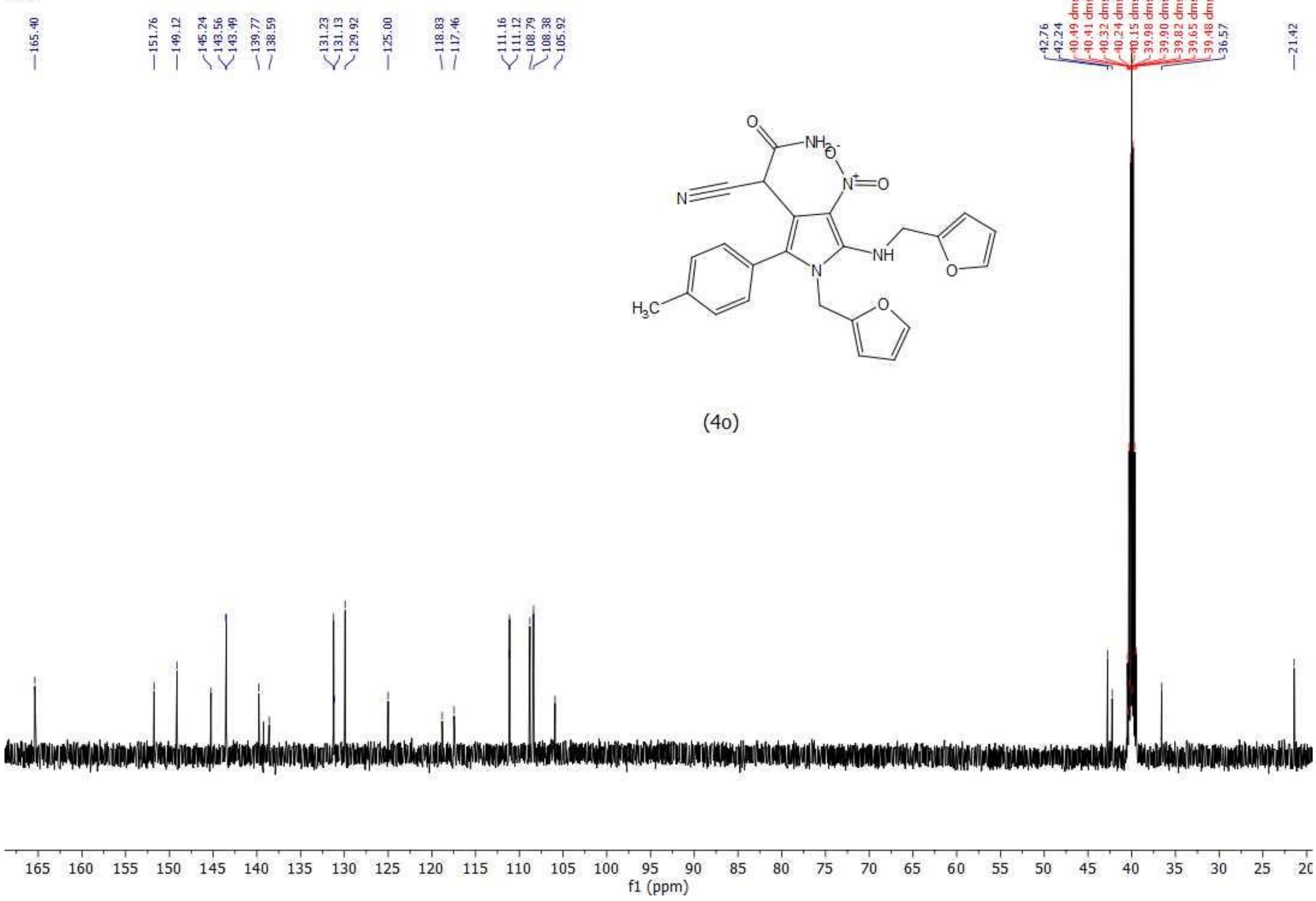
165.40
151.76
149.12
145.24
143.56
143.49
139.77
138.59
131.23
131.13
129.92
125.00
118.83
117.46
111.16
111.12
108.79
108.38
105.92
42.76
42.24
40.49 dmso
40.41 dmso
40.32 dmso
40.24 dmso
40.15 dmso
39.98 dmso
39.90 dmso
39.82 dmso
39.65 dmso
39.48 dmso
36.57
21.42
(4o)
f1 (ppm)

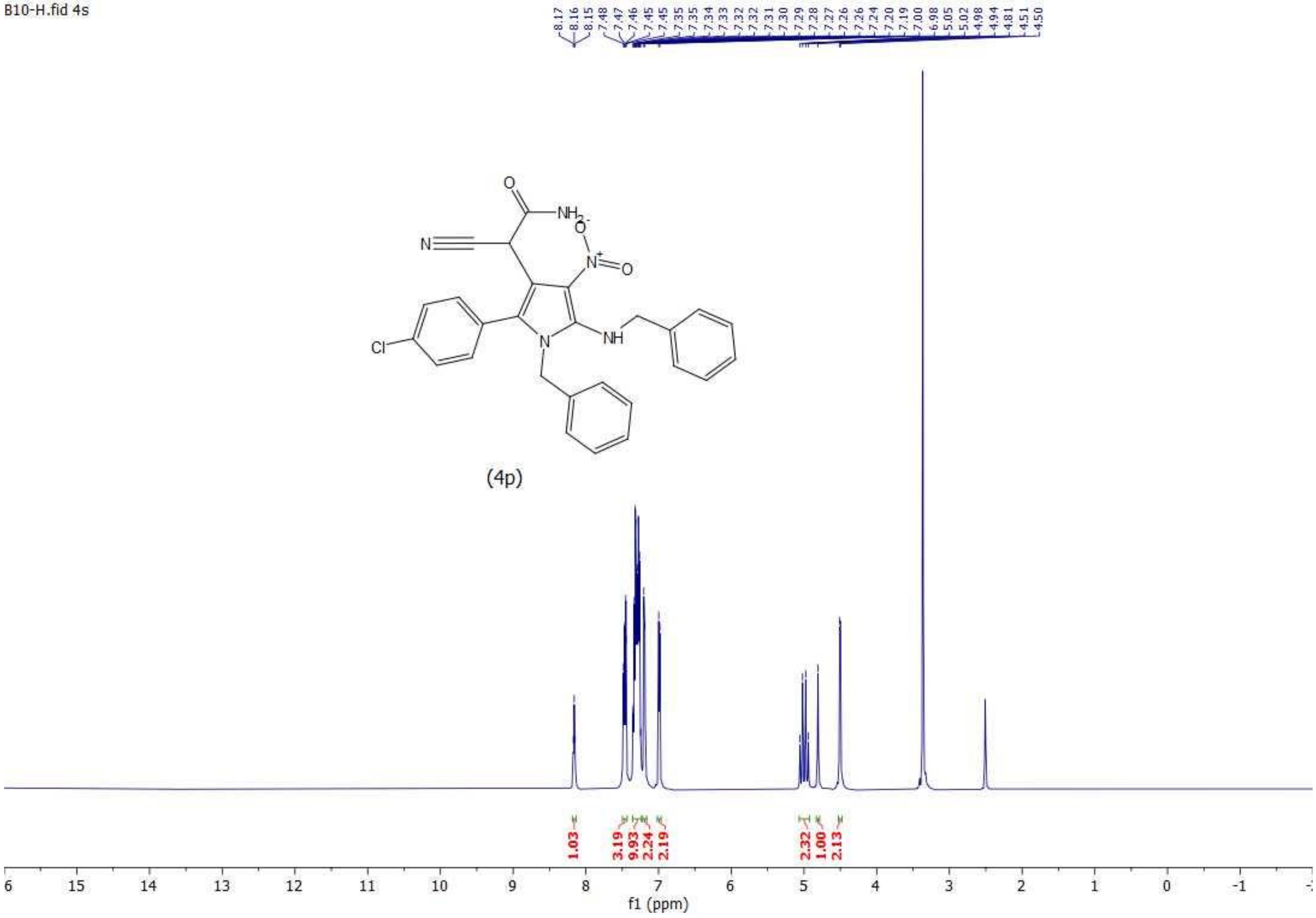
B10-H.fid 4s
8.17
8.16
8.15
7.48
7.47
7.46
7.45
7.45
7.35
7.35
7.34
7.33
7.32
7.32
7.31
7.30
7.29
7.28
7.27
7.26
7.26
7.24
7.20
7.19
7.00
6.98
5.05
5.02
4.98
4.94
4.81
4.51
4.50
N
O
NH2
O-
N+
O
Cl
N
NH
1.03
3.19
9.93
2.24
2.19
2.32
1.00
2.13
f1 (ppm)

(4p)

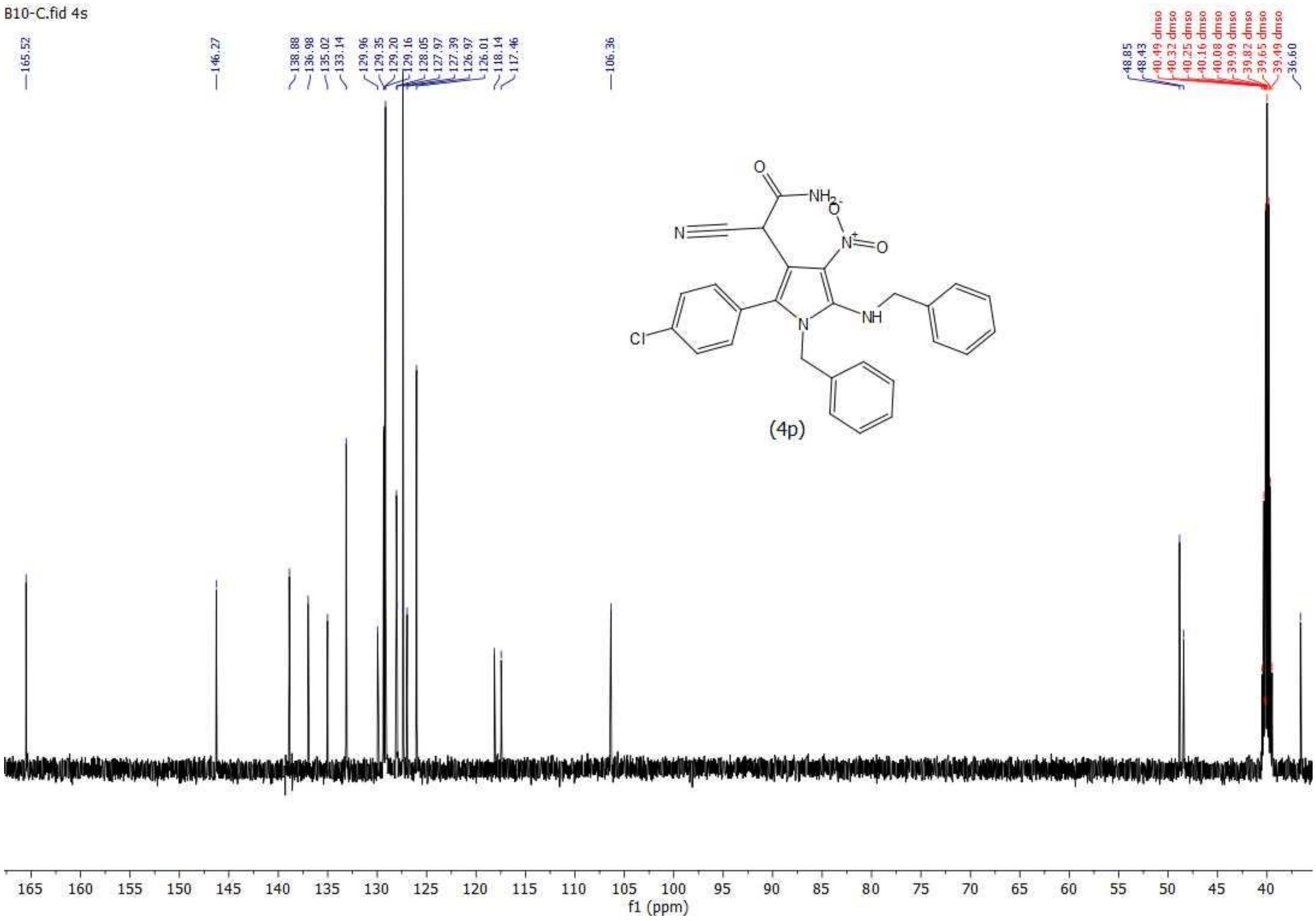
B10-C.fid 4s
165.52
146.27
138.88
136.98
135.02
133.14
129.96
129.35
129.20
129.16
128.05
127.97
127.39
126.97
126.01
118.14
117.46
106.36
48.85
48.43
40.49 dmso
40.32 dmso
40.25 dmso
40.16 dmso
40.08 dmso
39.99 dmso
39.82 dmso
39.65 dmso
39.49 dmso
36.60
O
NH2
O-
N+
O
N
NH
N
Cl
(4p)
f1 (ppm)

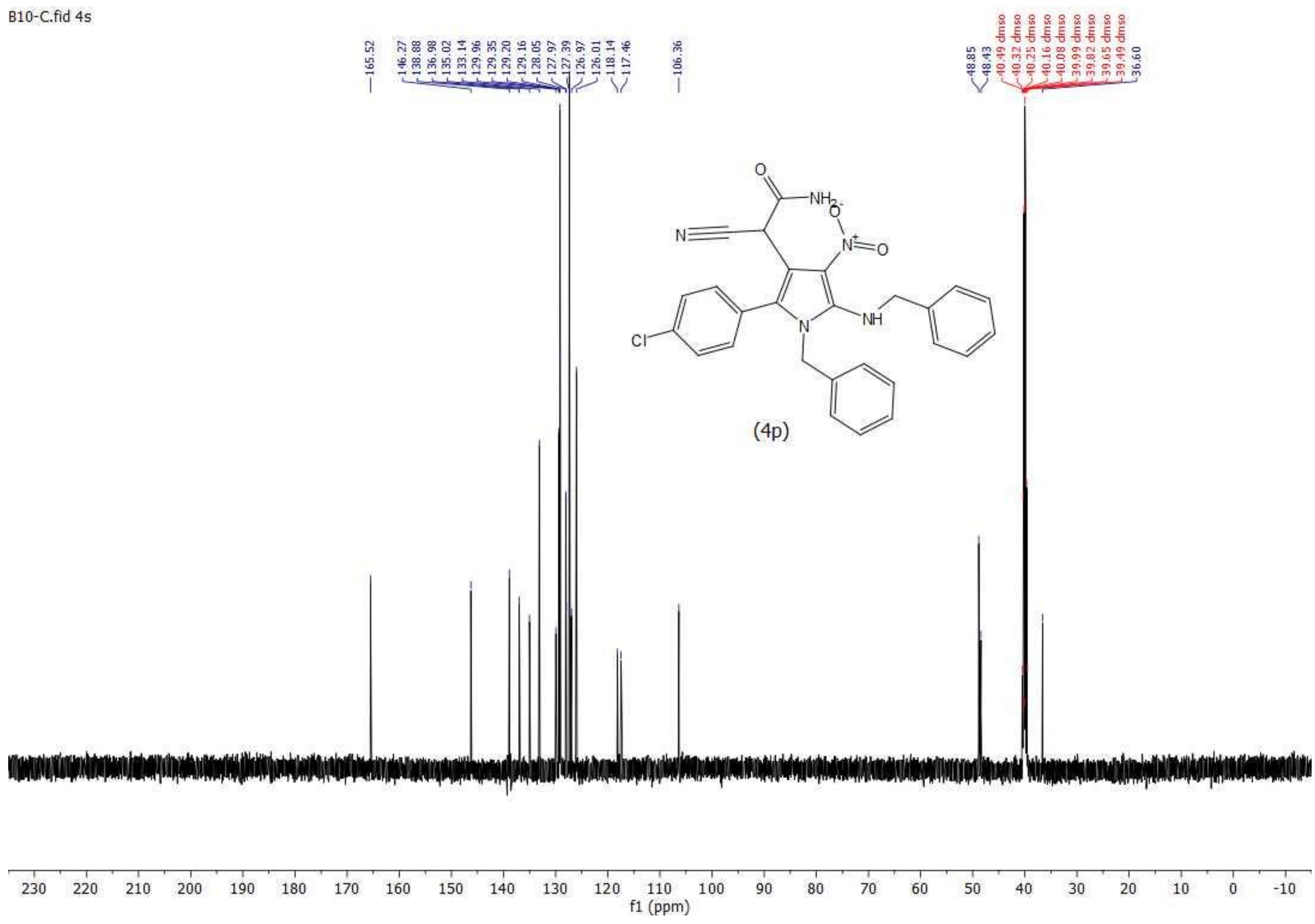

B10-C.fid 4s
165.52
146.27
138.88
136.98
135.02
133.14
129.96
129.35
129.20
129.16
128.05
127.97
127.39
126.97
126.01
118.14
117.46
106.36
48.85
48.43
40.49 dmso
40.32 dmso
40.25 dmso
40.16 dmso
40.08 dmso
39.99 dmso
39.82 dmso
39.65 dmso
39.49 dmso
36.60
O
NH_2
N
N^+
O^-
O
NH
Cl
N
(4p)
230 220 210 200 190 180 170 160 150 140 130 120 110 100 90 80 70 60 50 40 30 20 10 0 -10
f1 (ppm)

B10-C.fid 4s

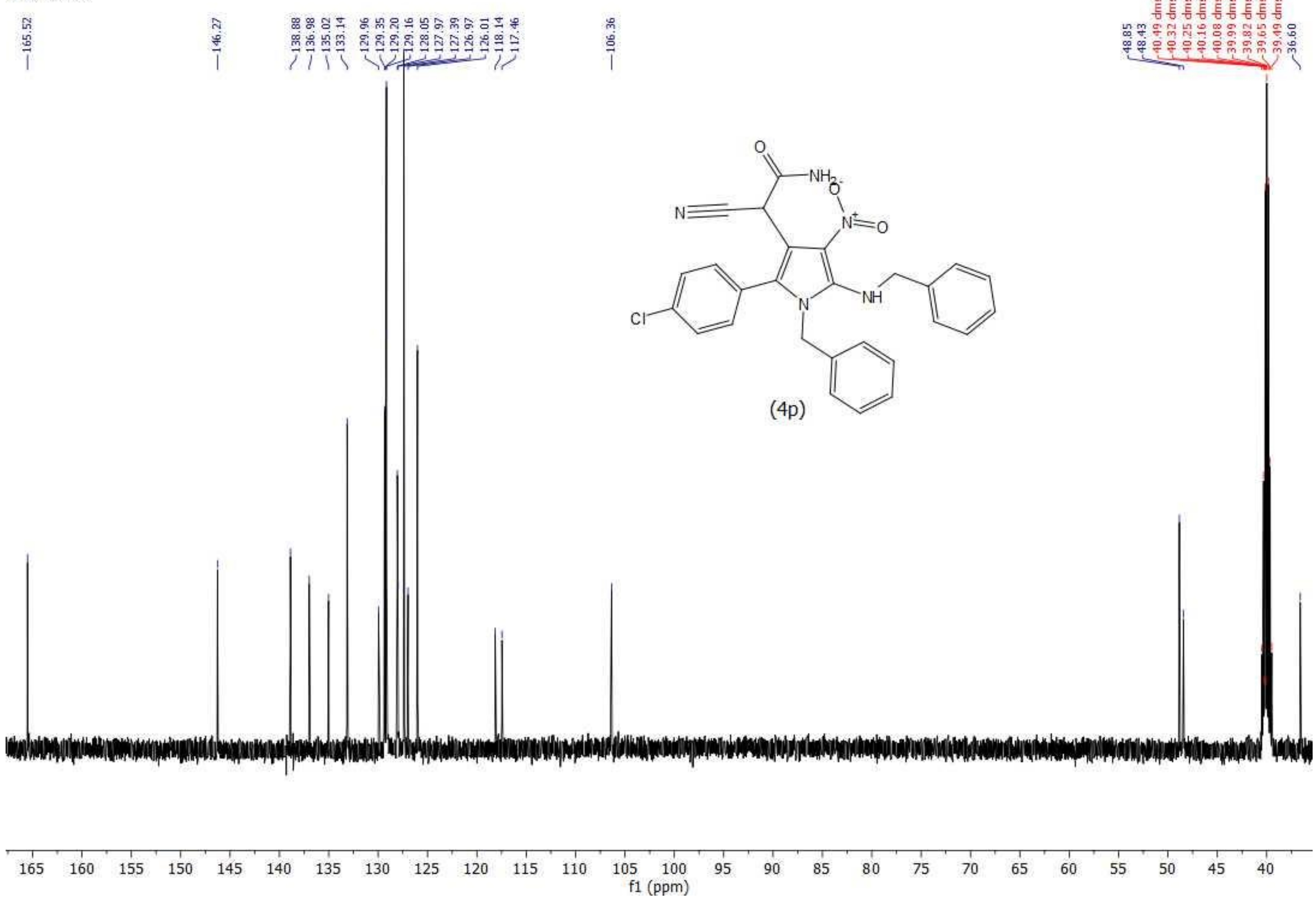

165.52
146.27
138.88
136.98
135.02
133.14
129.96
129.35
129.20
129.16
128.05
127.97
127.39
126.97
126.01
118.14
117.46
106.36
48.85
48.43
40.49 dmso
40.32 dmso
40.25 dmso
40.16 dmso
40.08 dmso
39.99 dmso
39.82 dmso
39.65 dmso
39.49 dmso
36.60
(4p)
f1 (ppm)

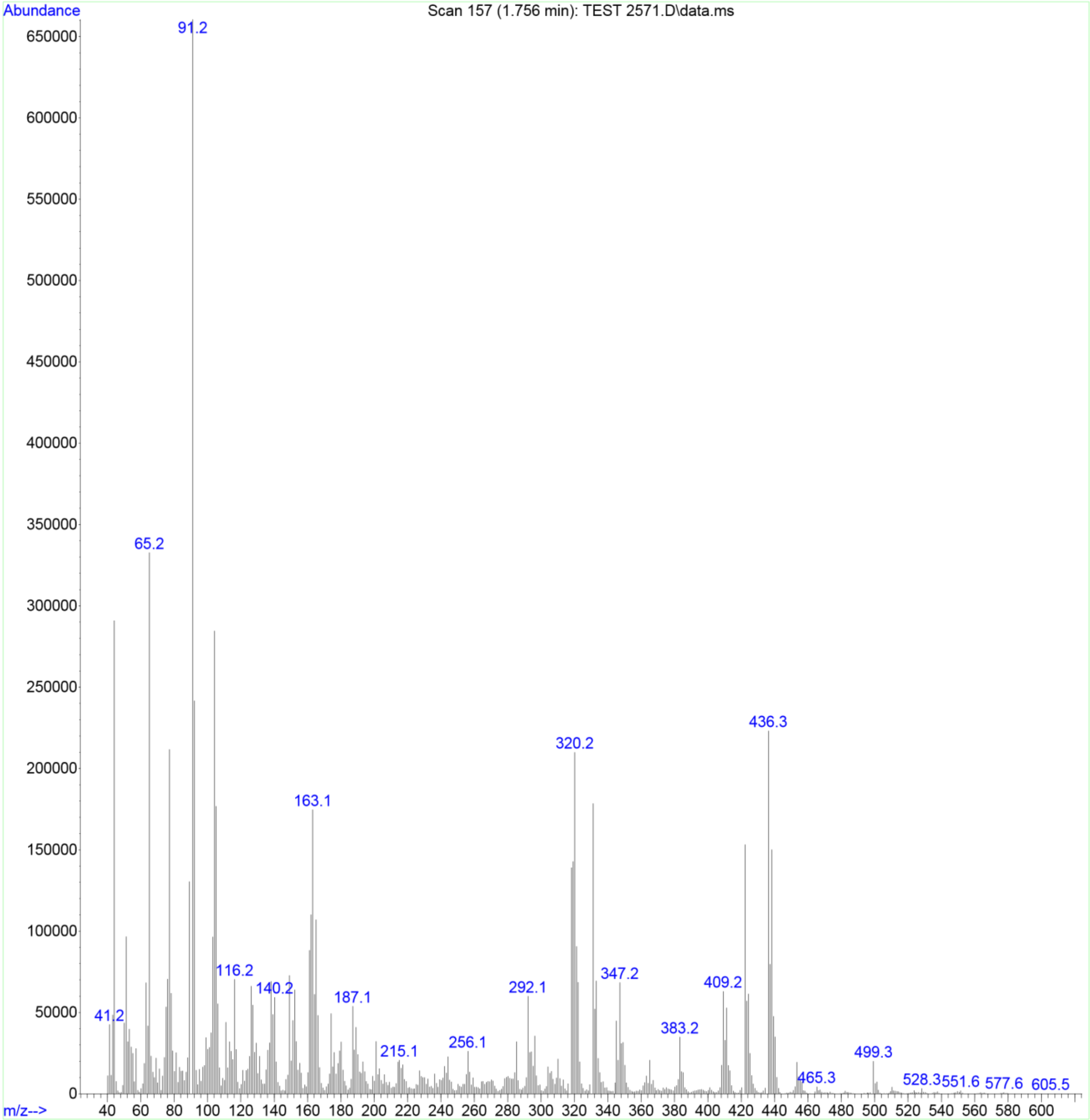

$C_{27}H_{22}ClN_5O_3$

(499/9)

(4p)

B11-H.fid 4 q

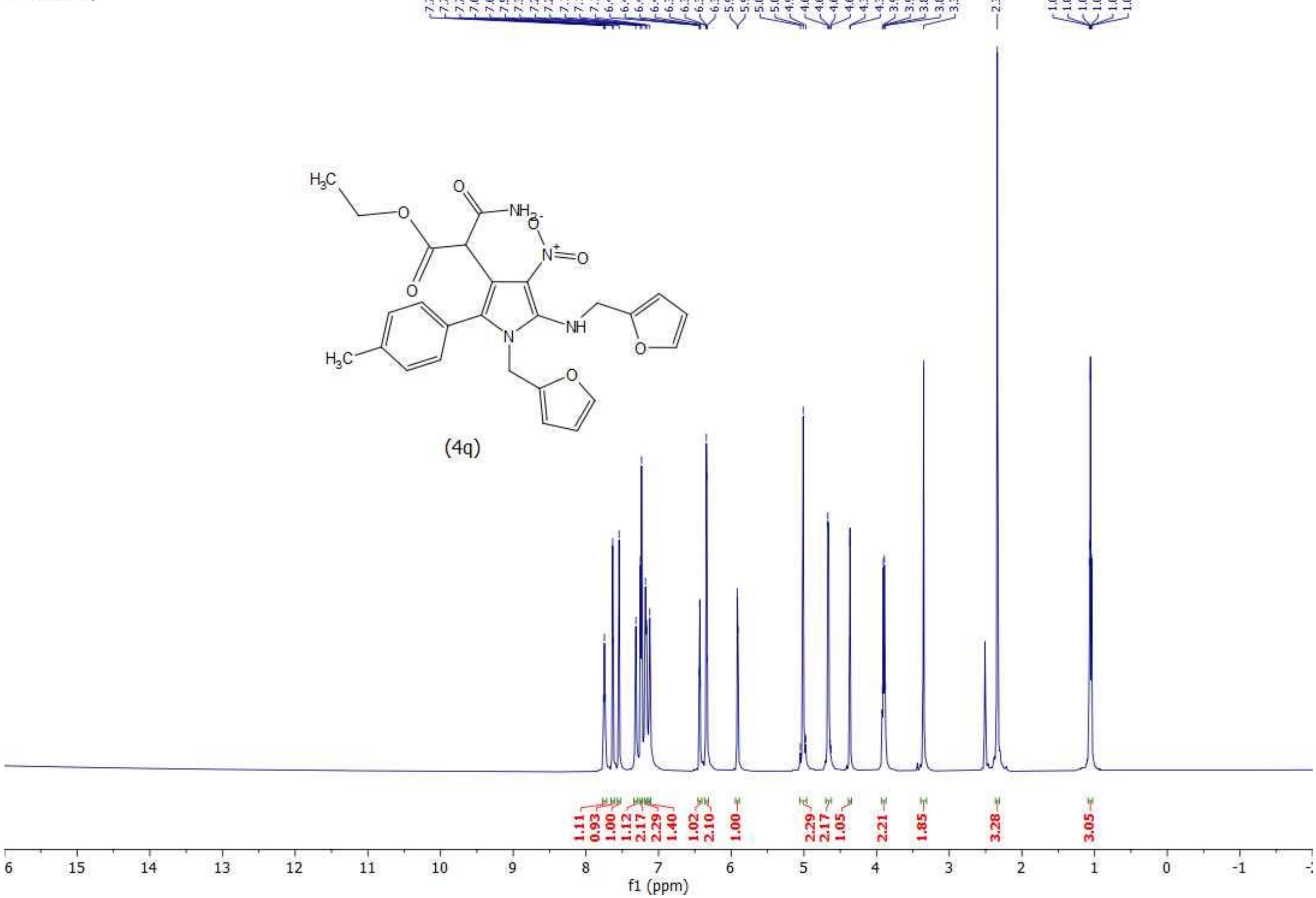
7.76
7.74
7.73
7.63
7.63
7.54
7.31
7.25
7.23
7.18
7.16
7.12
6.44
6.43
6.43
6.42
6.35
6.34
6.34
6.33
5.92
5.91
5.05
5.01
4.97
4.67
4.66
4.64
4.63
4.37
4.36
3.92
3.91
3.89
3.88
3.35
2.34
1.07
1.07
1.06
1.06
1.04
1.04
H3C
O
NH2
O
N+
O
O
NH
O
H3C
N
O
(4q)
1.11
0.93
1.00
1.12
2.17
2.29
1.40
1.02
2.10
1.00
2.29
2.17
1.05
2.21
1.85
3.28
3.05
f1 (ppm)

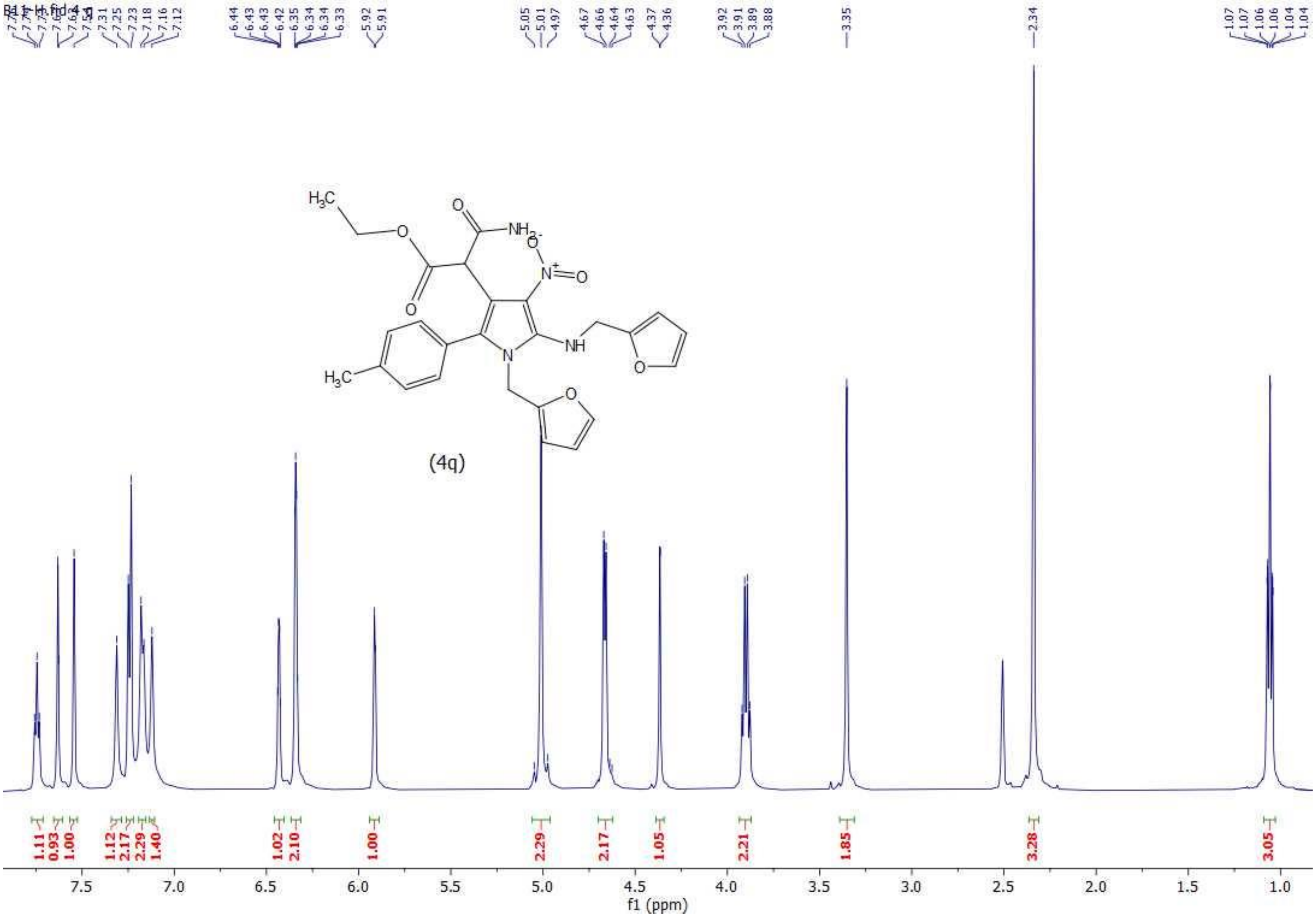
B11-H.fid 4 q
7.31
7.25
7.23
7.18
7.16
7.12
6.44
6.43
6.43
6.42
6.35
6.34
6.34
6.33
5.92
5.91
5.05
5.01
4.97
4.67
4.66
4.64
4.63
4.37
4.36
3.92
3.91
3.89
3.88
3.35
2.34
1.07
1.07
1.06
1.06
1.04
1.04
(4q)
1.11
0.93
1.00
1.12
2.17
2.29
1.40
1.02
2.10
1.00
2.29
2.17
1.05
2.21
1.85
3.28
3.05
f1 (ppm)

C4q

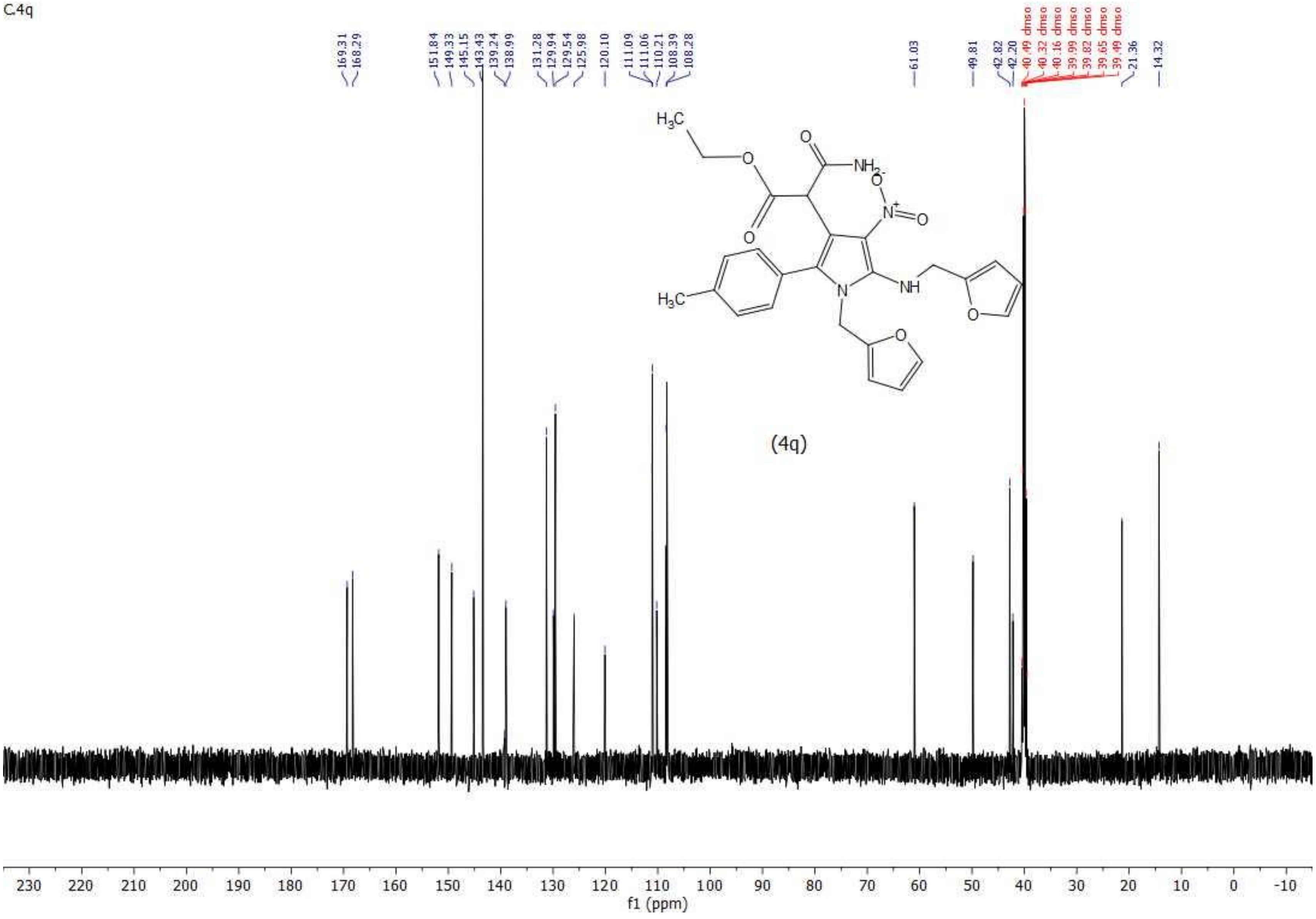
169.31
168.29
151.84
149.33
145.15
143.43
139.24
138.99
131.28
129.94
129.54
125.98
120.10
111.09
111.06
110.21
108.39
108.28
61.03
49.81
42.82
42.20
40.49 dmso
40.32 dmso
40.16 dmso
39.99 dmso
39.82 dmso
39.65 dmso
39.49 dmso
21.36
14.32
f1 (ppm)

(4q)

C4q

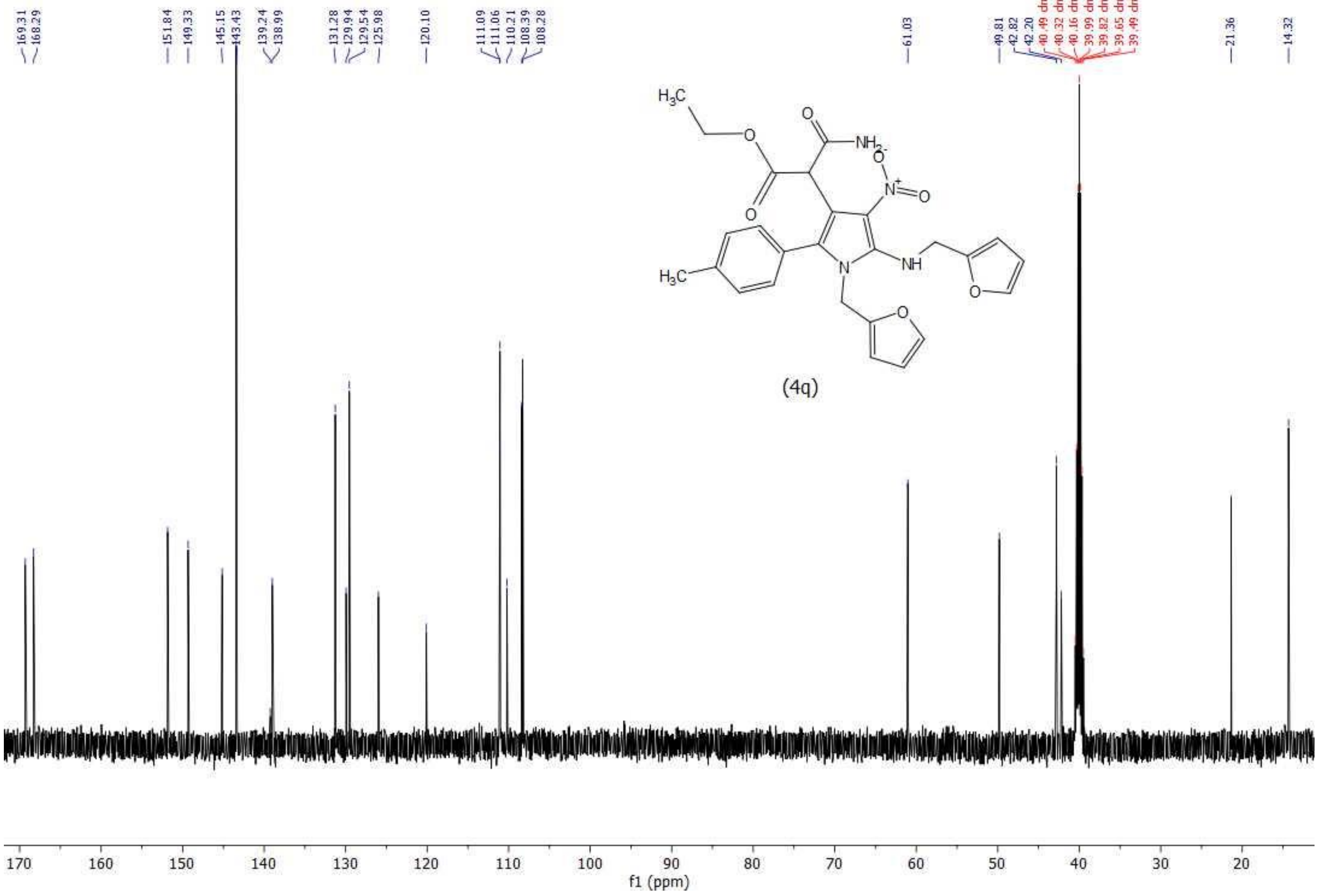
169.31
168.29
151.84
149.33
145.15
143.43
139.24
138.99
131.28
129.94
129.54
125.98
120.10
111.09
111.06
110.21
108.39
108.28
61.03
49.81
42.82
42.20
40.49 dmso
40.32 dmso
40.16 dmso
39.99 dmso
39.82 dmso
39.65 dmso
39.49 dmso
21.36
14.32
(4q)
f1 (ppm)

B12-H

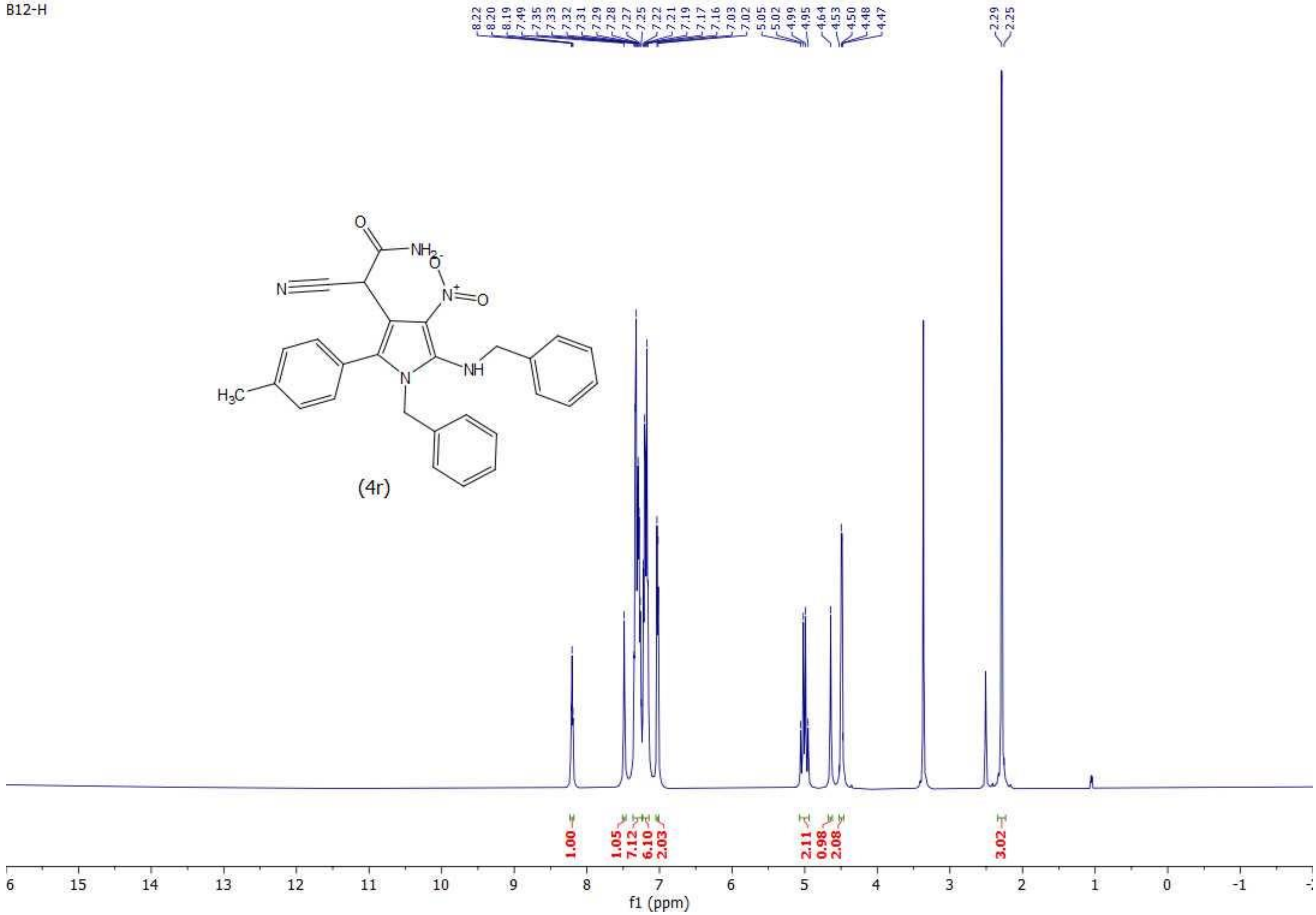
8.22
8.20
8.19
7.49
7.35
7.33
7.32
7.31
7.29
7.28
7.27
7.25
7.22
7.21
7.19
7.17
7.16
7.03
7.02
5.05
5.02
4.99
4.95
4.64
4.53
4.50
4.48
4.47
2.29
2.25
H_3C
N
O
NH_2
O^-
N^+
O
NH
N
(4r)
1.00
1.05
7.12
6.10
2.03
2.11
0.98
2.08
3.02
15
14
13
12
11
10
9
8
7
6
5
4
3
2
1
0
-1
f1 (ppm)

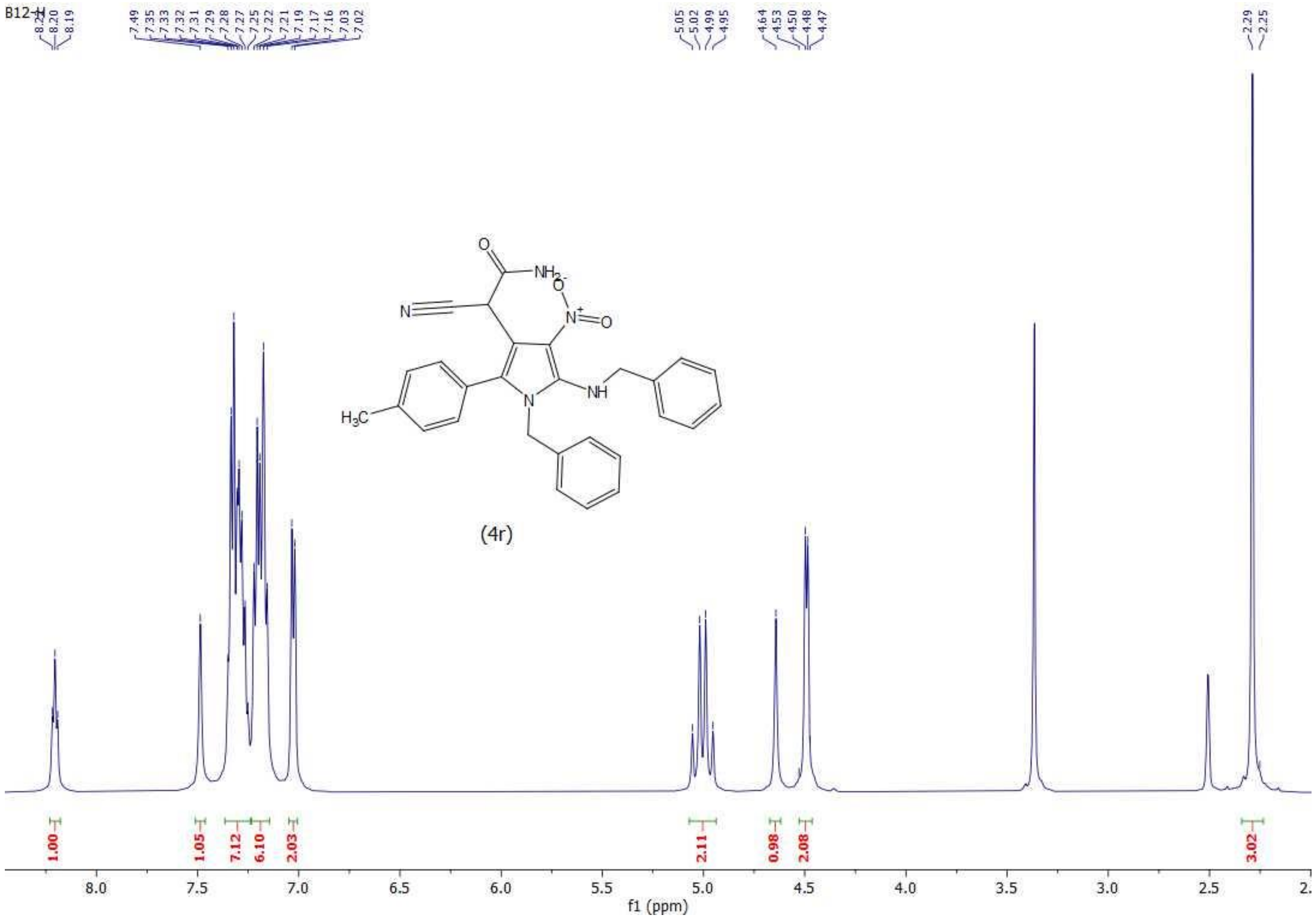
B12-H
8.21
8.20
8.19
7.49
7.35
7.33
7.32
7.31
7.29
7.28
7.27
7.25
7.22
7.21
7.19
7.17
7.16
7.03
7.02
5.05
5.02
4.99
4.95
4.64
4.53
4.50
4.48
4.47
2.29
2.25
O
NH2-
O-
N+
O
N
NH
N
H3C
(4r)
1.00
1.05
7.12
6.10
2.03
2.11
0.98
2.08
3.02
8.0
7.5
7.0
6.5
6.0
5.5
5.0
4.5
4.0
3.5
3.0
2.5
2.
f1 (ppm)

C4r

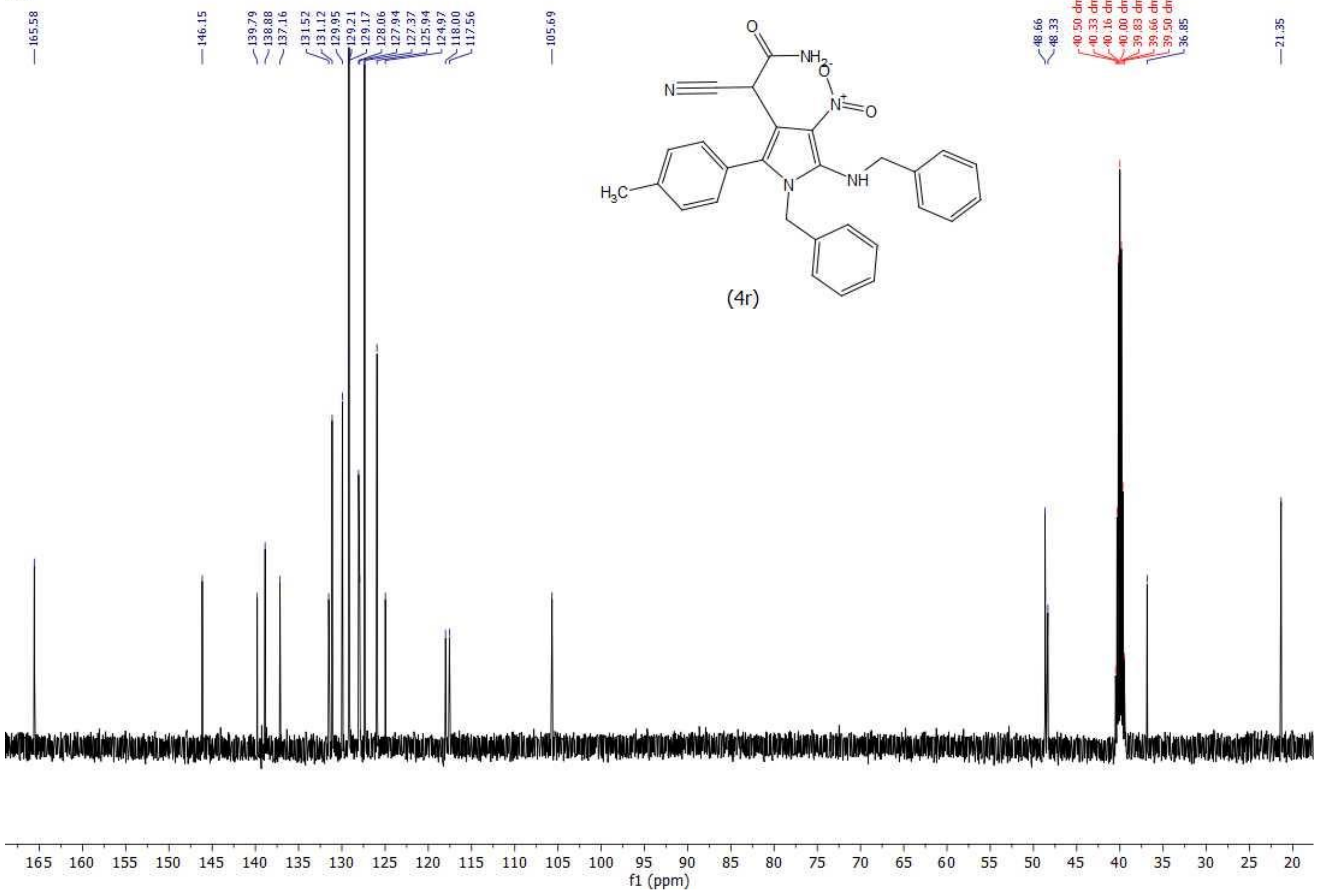

165.58
146.15
139.79
138.88
137.16
131.52
131.12
129.95
129.21
129.17
128.06
127.94
127.37
125.94
124.97
118.00
117.56
105.69
48.66
48.33
40.50 dmso
40.33 dmso
40.16 dmso
40.00 dmso
39.83 dmso
39.66 dmso
39.50 dmso
36.85
21.35
(4r)
f1 (ppm)

C4r

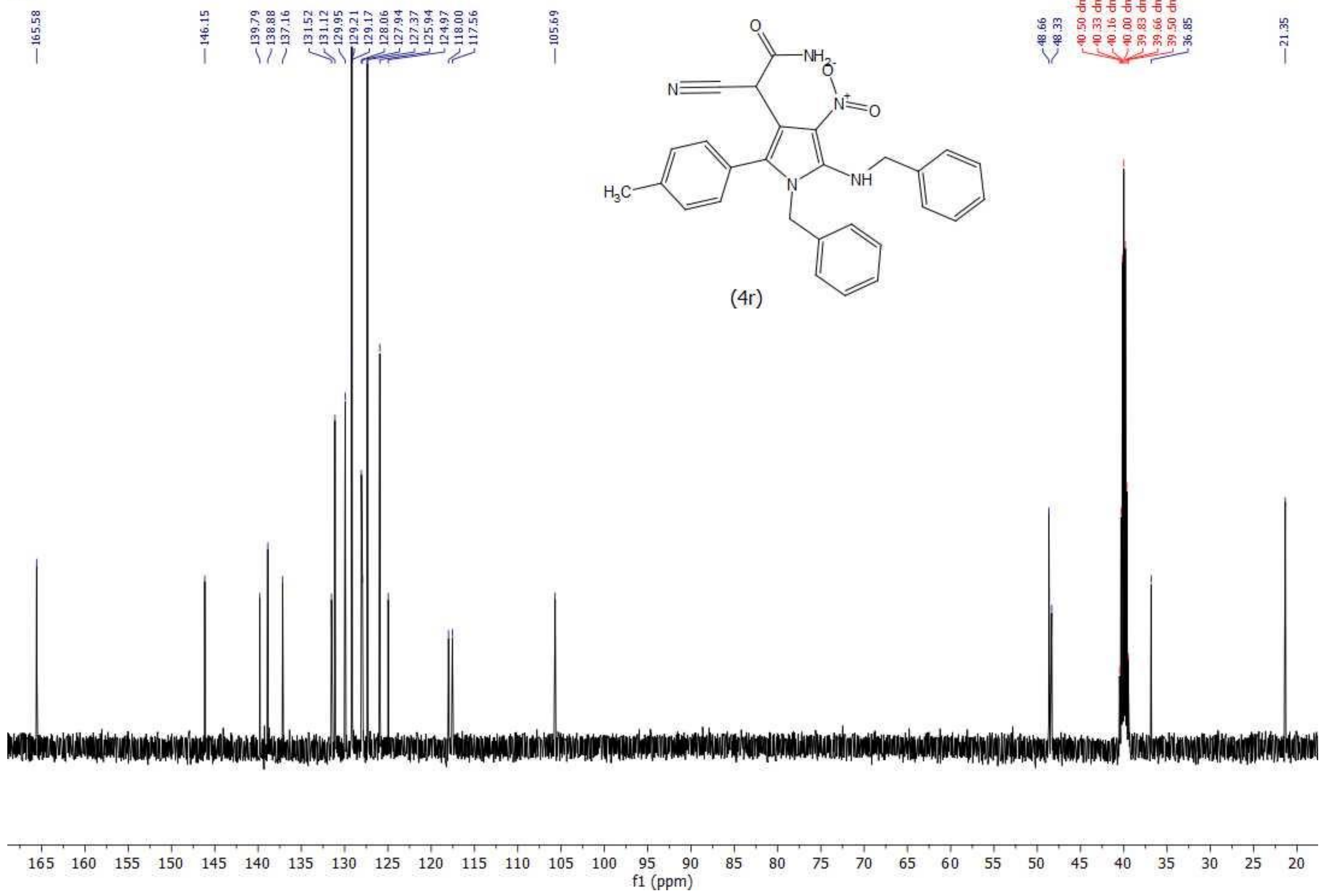
165.58
146.15
139.79
138.88
137.16
131.52
131.12
129.95
129.21
129.17
128.06
127.94
127.37
125.94
124.97
118.00
117.56
105.69
48.66
48.33
40.50 dmso
40.33 dmso
40.16 dmso
40.00 dmso
39.83 dmso
39.66 dmso
39.50 dmso
36.85
21.35
O
NH2
N
O
N
H3C
N
NH
(4r)
165 160 155 150 145 140 135 130 125 120 115 110 105 100 95 90 85 80 75 70 65 60 55 50 45 40 35 30 25 20
f1 (ppm)

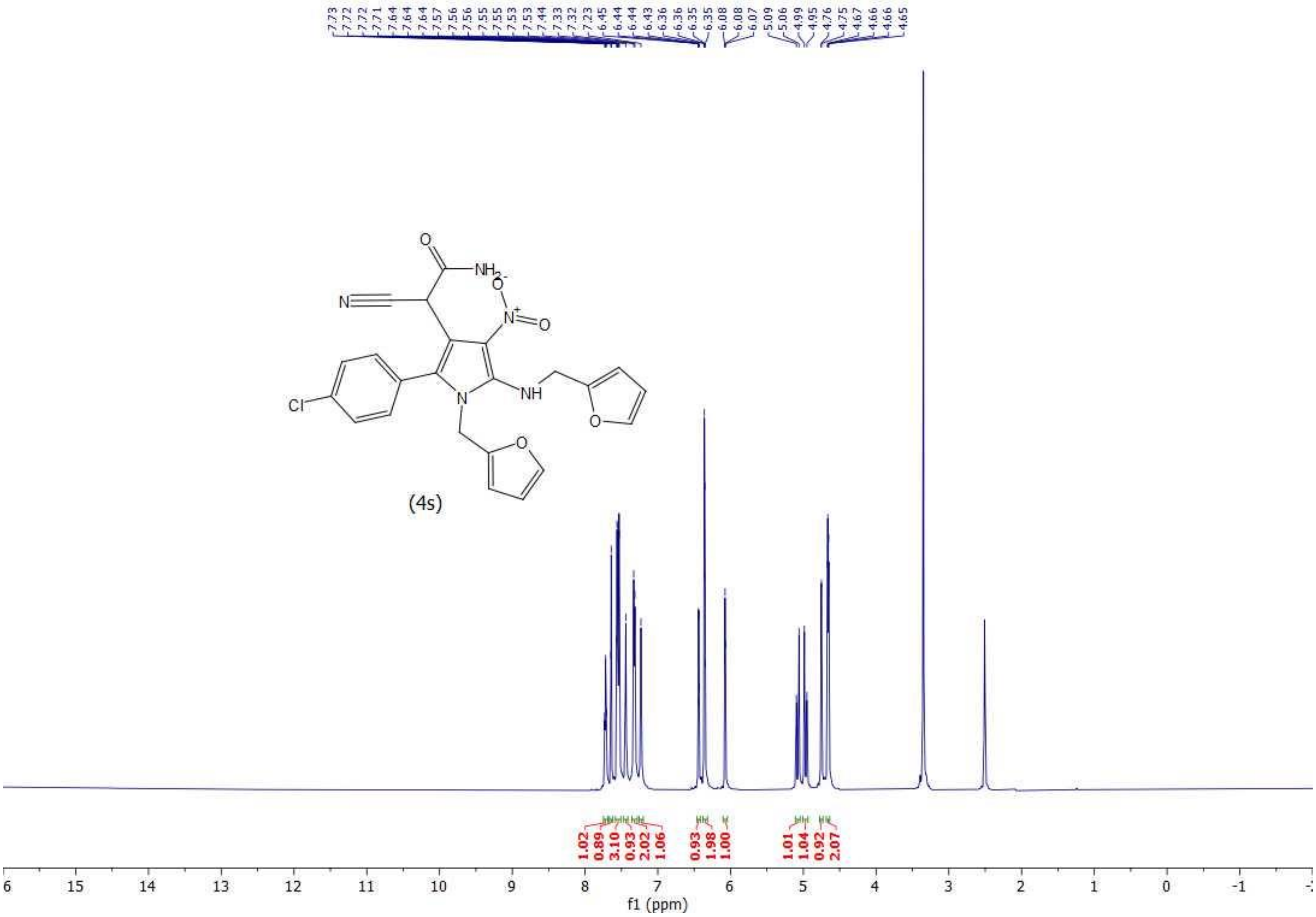

7.73
7.72
7.72
7.71
7.64
7.64
7.64
7.57
7.56
7.56
7.55
7.55
7.53
7.53
7.44
7.33
7.32
7.23
6.45
6.44
6.44
6.43
6.36
6.36
6.35
6.35
6.08
6.08
6.07
5.09
5.06
4.99
4.95
4.76
4.75
4.67
4.66
4.66
4.65
O
NH2
N
O-
N+
O
Cl
N
NH
O
O
(4s)
1.02
0.89
3.10
0.93
2.02
1.06
0.93
1.98
1.00
1.01
1.04
0.92
2.07
15
14
13
12
11
10
9
8
7
6
5
4
3
2
1
0
-1
f1 (ppm)

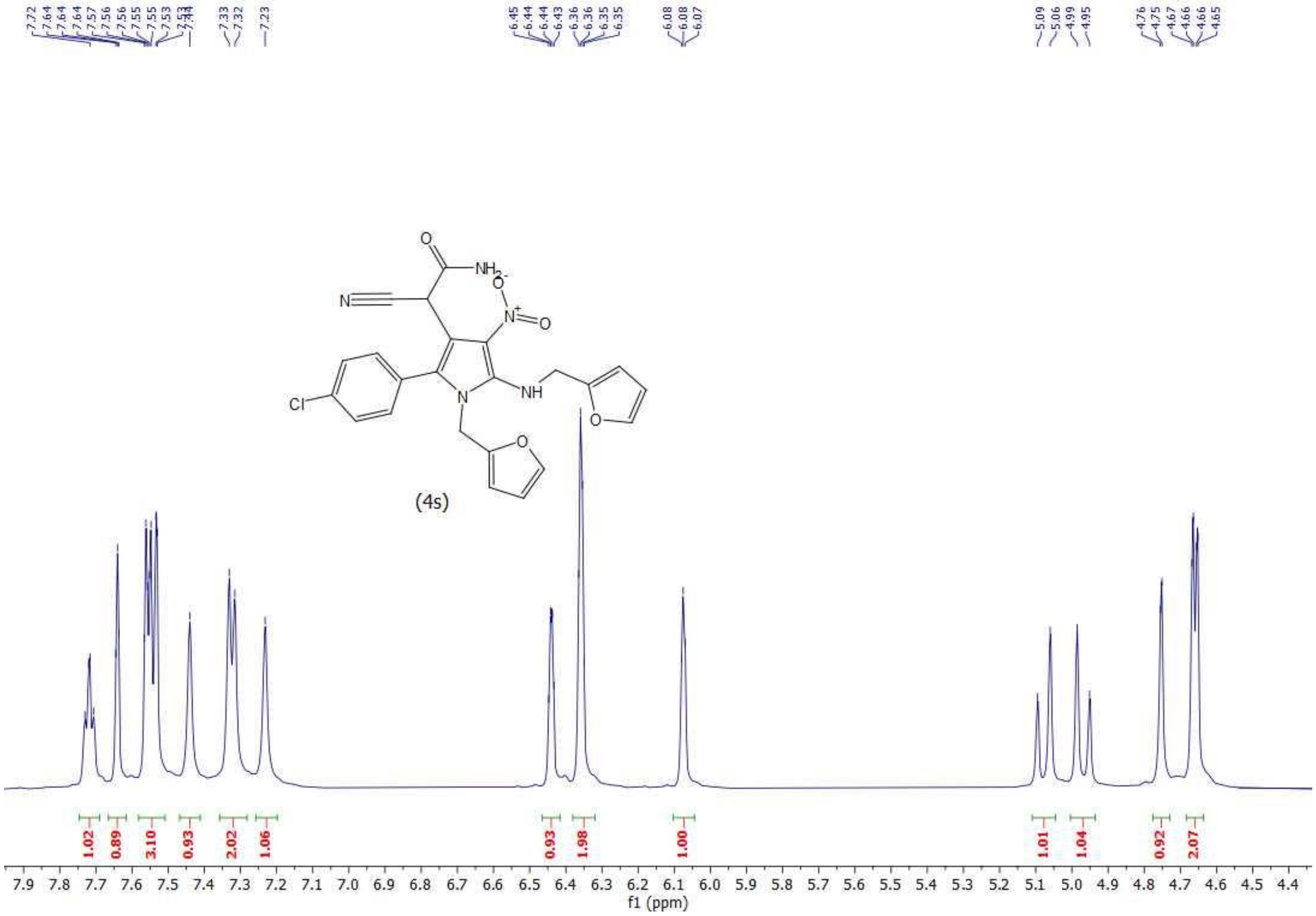
7.72 7.64 7.64 7.64 7.57 7.56 7.56 7.55 7.55 7.53 7.53 7.44
7.33 7.32
7.23
6.45 6.44 6.44 6.43 6.36 6.36 6.35 6.35
6.08 6.08 6.07
5.09 5.06 4.99 4.95
4.76 4.75 4.67 4.66 4.66 4.65
O
NH2
N
O
N+
O
Cl
N
NH
O
O
(4s)
1.02 0.89 3.10 0.93 2.02 1.06 0.93 1.98 1.00 1.01 1.04 0.92 2.07
7.9 7.8 7.7 7.6 7.5 7.4 7.3 7.2 7.1 7.0 6.9 6.8 6.7 6.6 6.5 6.4 6.3 6.2 6.1 6.0 5.9 5.8 5.7 5.6 5.5 5.4 5.3 5.2 5.1 5.0 4.9 4.8 4.7 4.6 4.5 4.4
f1 (ppm)

C4s

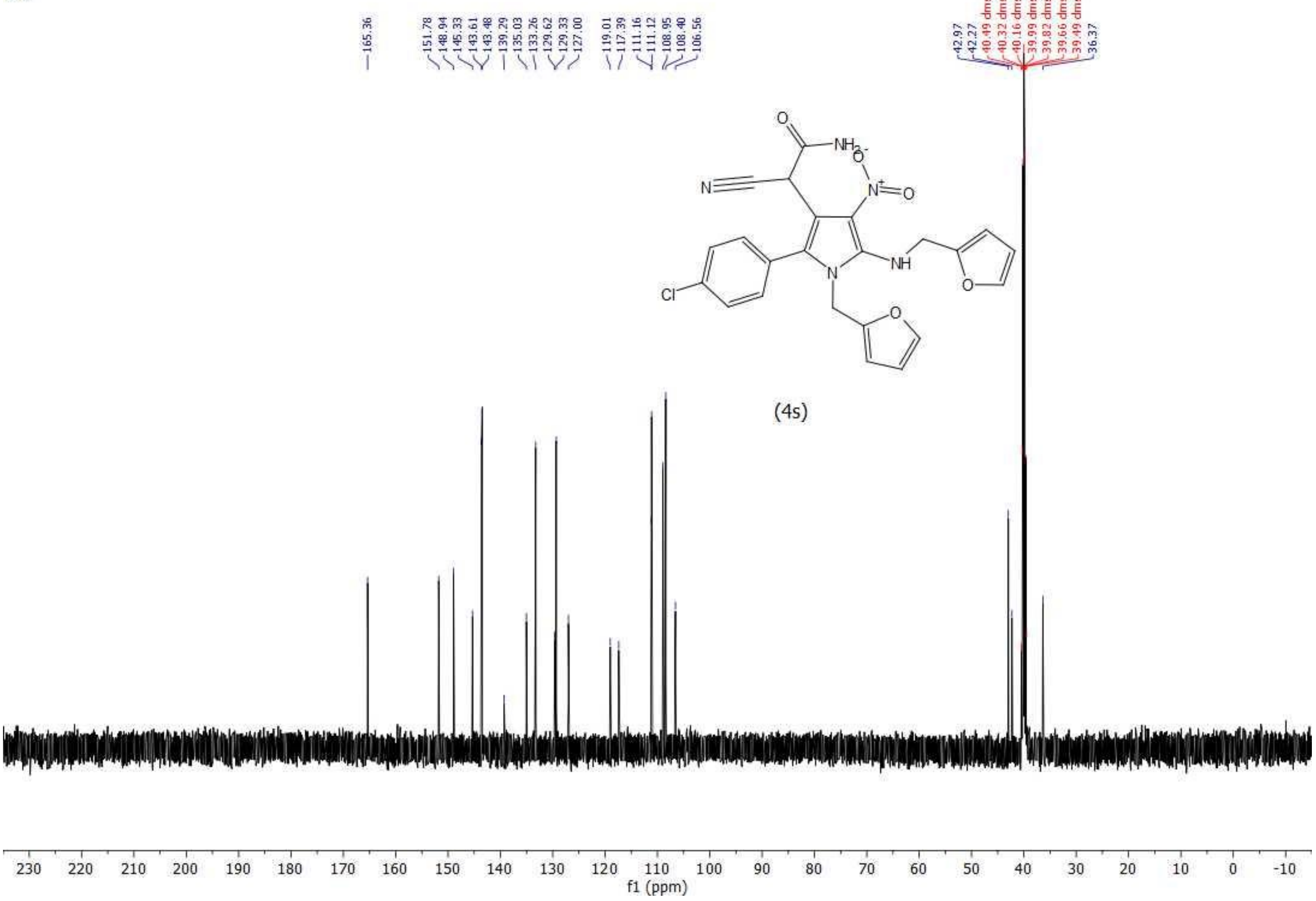

165.36
151.78
148.94
145.33
143.61
143.48
139.29
135.03
133.26
129.62
129.33
127.00
119.01
117.39
111.16
111.12
108.95
108.40
106.56
42.97
42.27
40.49 dmso
40.32 dmso
40.16 dmso
39.99 dmso
39.82 dmso
39.66 dmso
39.49 dmso
36.37
O
NH_2
N
Cl
NH
O
(4s)
230 220 210 200 190 180 170 160 150 140 130 120 110 100 90 80 70 60 50 40 30 20 10 0 -10
f1 (ppm)

C4s

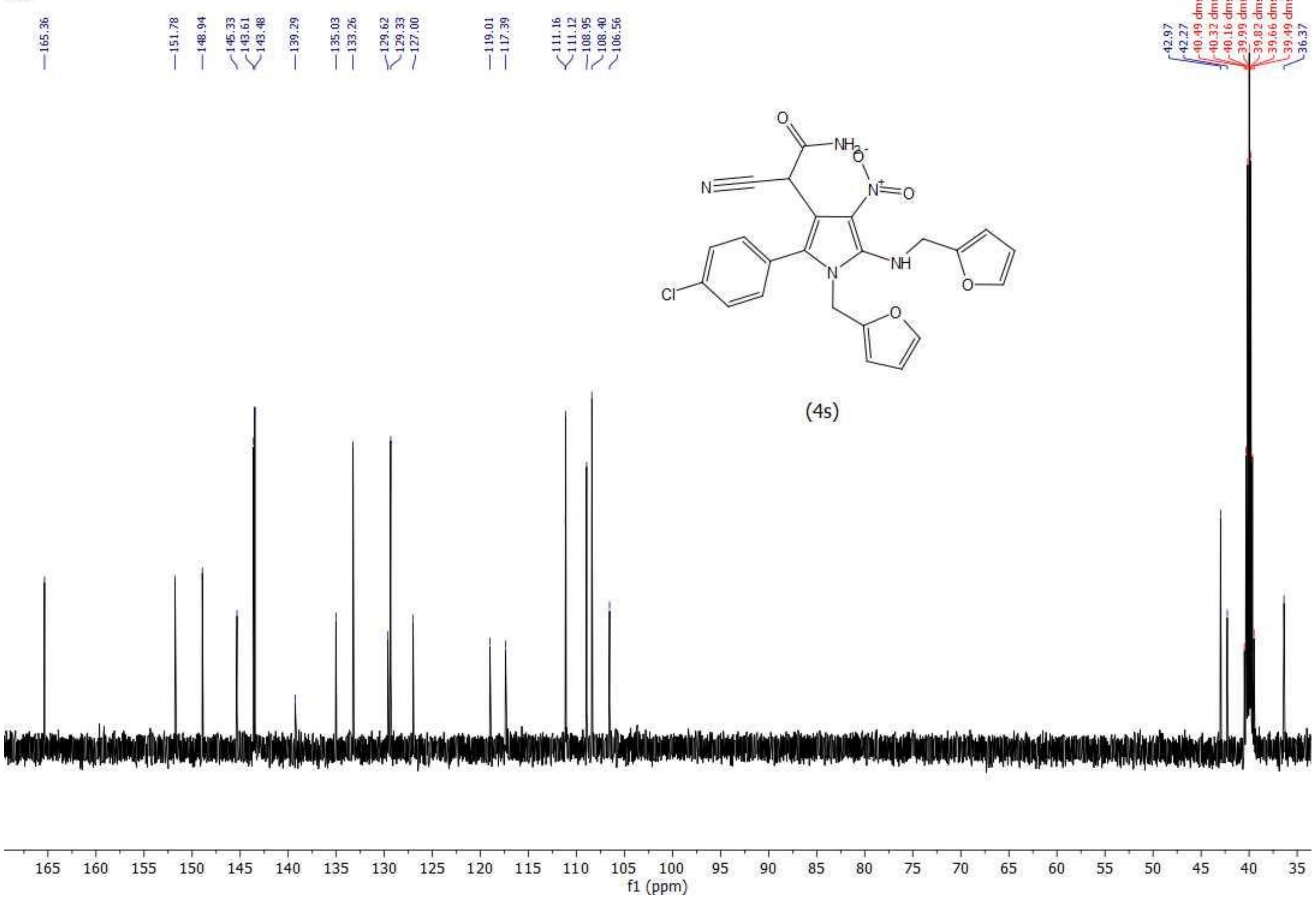
165.36
151.78
148.94
145.33
143.61
143.48
139.29
135.03
133.26
129.62
129.33
127.00
119.01
117.39
111.16
111.12
108.95
108.40
106.56
42.97
42.27
40.49 dmso
40.32 dmso
40.16 dmso
39.99 dmso
39.82 dmso
39.66 dmso
39.49 dmso
36.37
(4s)
f1 (ppm)

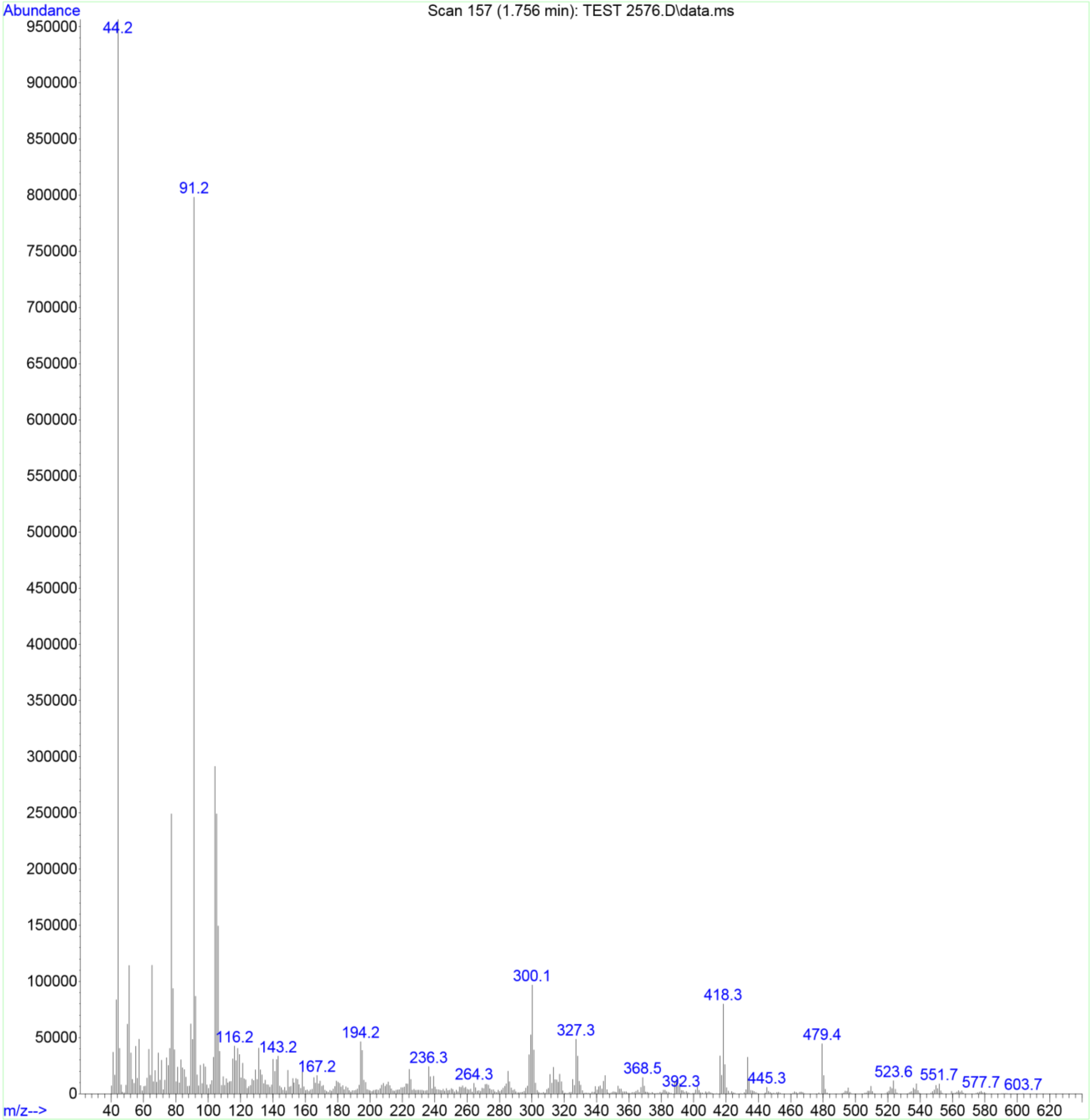

$C_{23}H_{18}ClN_5O_5$

(479/8)

(4s)

H.4t

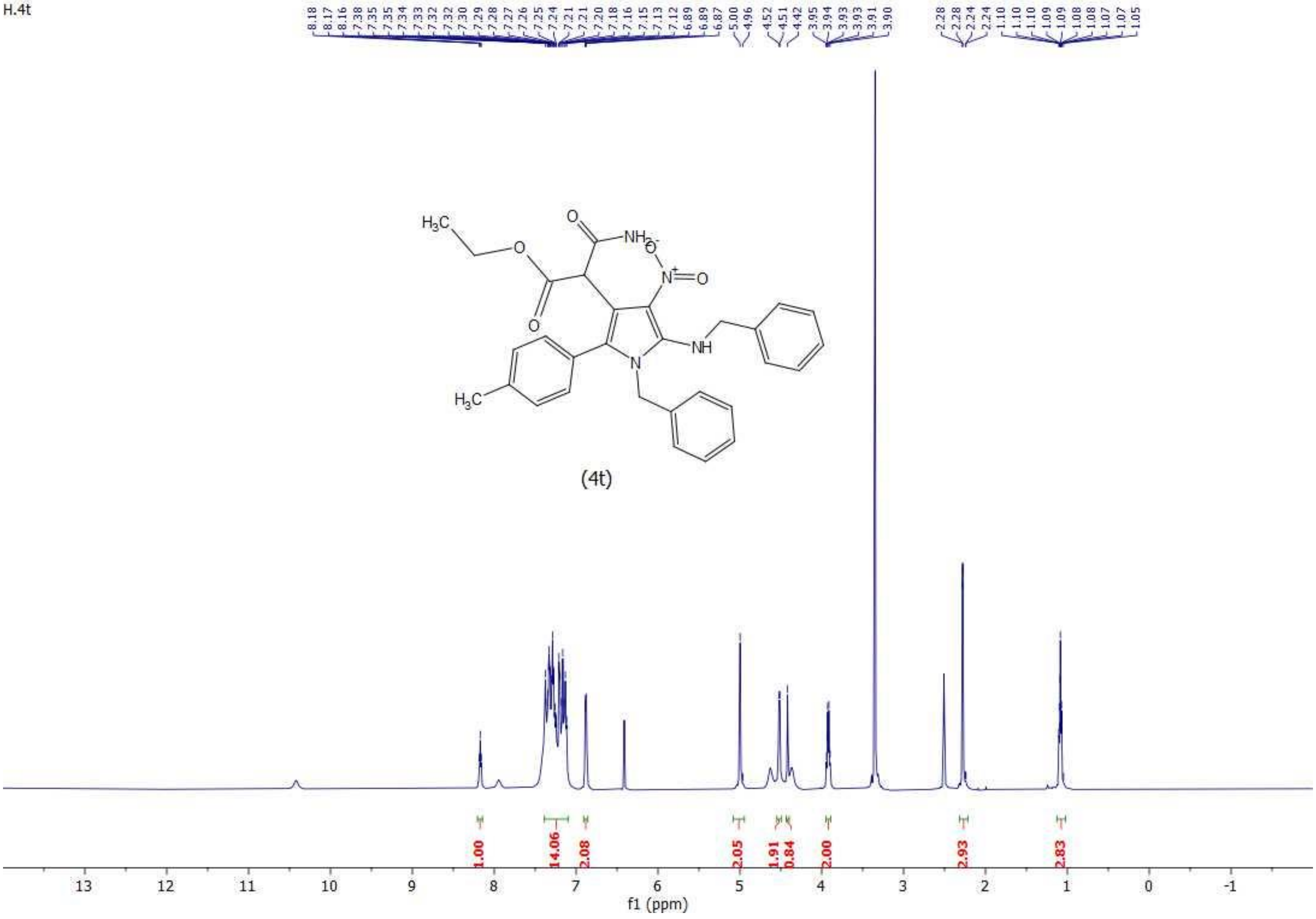
8.18 8.17 8.16 7.38 7.35 7.34 7.33 7.32 7.32 7.30 7.29 7.28 7.27 7.26 7.25 7.24 7.21 7.21 7.20 7.18 7.16 7.15 7.13 7.12 6.89 6.89 6.87 5.00 4.96 4.52 4.51 4.42 3.95 3.94 3.93 3.93 3.91 3.90 2.28 2.28 2.24 2.24 1.10 1.10 1.10 1.09 1.09 1.08 1.08 1.07 1.07 1.05
H3C
O
NH2
O
N
O
NH
N
H3C
(4t)
1.00
14.06
2.08
2.05
1.91
0.84
2.00
2.93
2.83
13 12 11 10 9 8 7 6 5 4 3 2 1 0 -1
f1 (ppm)

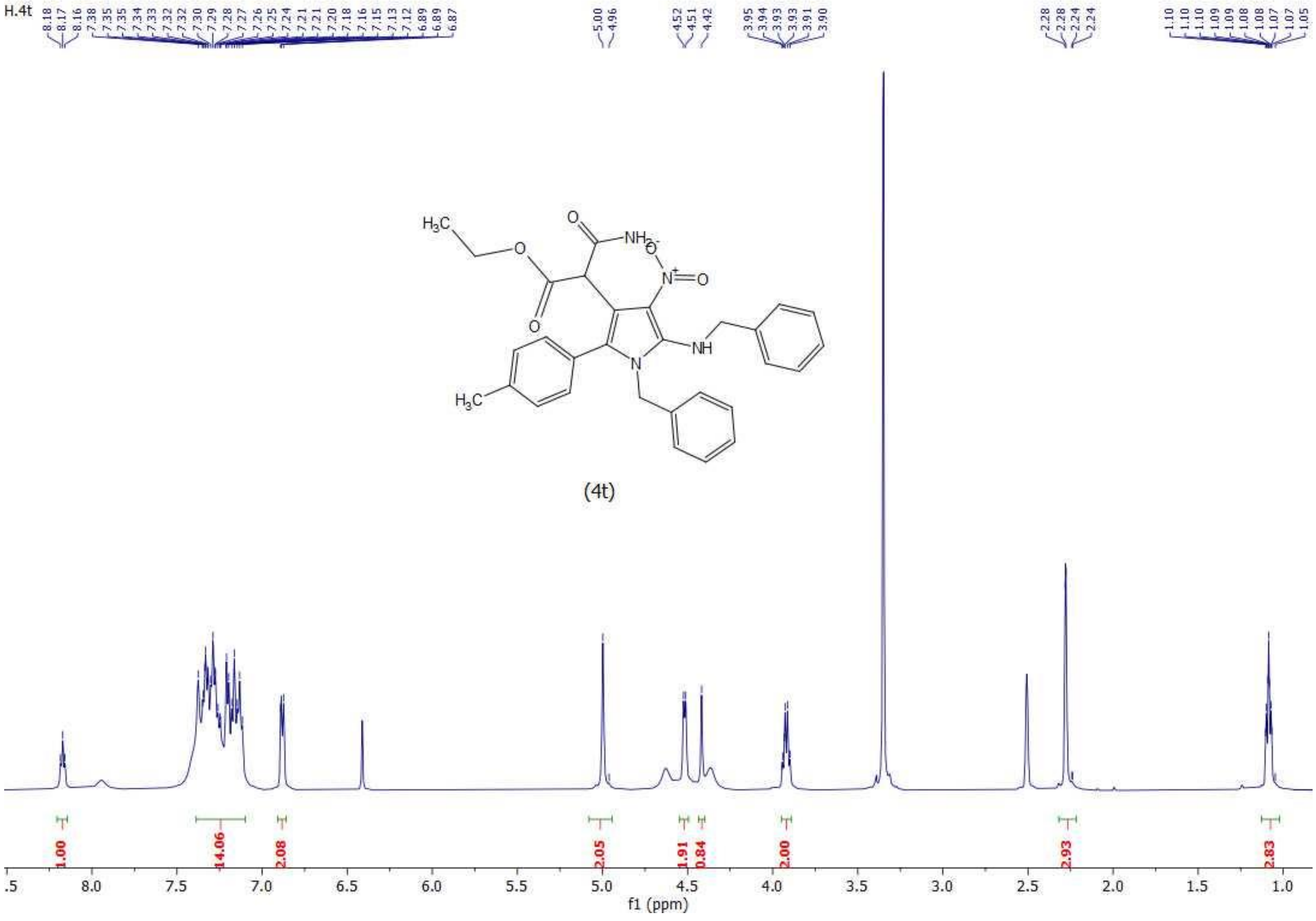
H.4t
8.18 8.17 8.16 7.38 7.35 7.35 7.34 7.33 7.32 7.32 7.30 7.29 7.28 7.27 7.26 7.25 7.24 7.21 7.21 7.20 7.18 7.16 7.15 7.13 7.12 6.89 6.89 6.87
5.00 4.96
4.52 4.51 4.42
3.95 3.94 3.93 3.93 3.91 3.90
2.28 2.28 2.24 2.24
1.10 1.10 1.10 1.09 1.09 1.08 1.08 1.08 1.07 1.07 1.05
(4t)
1.00
14.06
2.08
2.05
1.91
0.84
2.00
2.93
2.83
f1 (ppm)

C4t

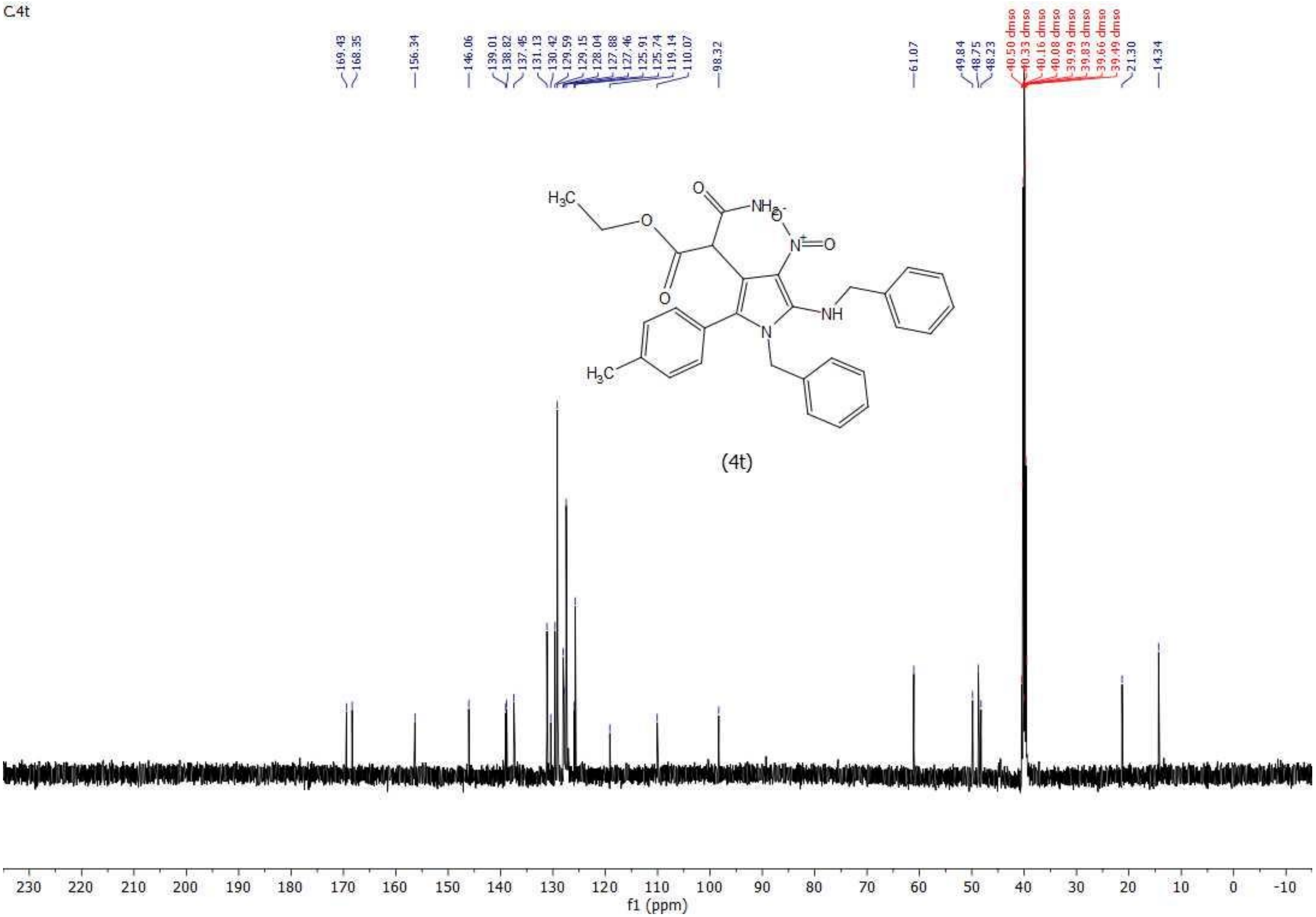
169.43
168.35
156.34
146.06
139.01
138.82
137.45
131.13
130.42
129.59
129.15
128.04
127.88
127.46
125.91
125.74
119.14
110.07
98.32
61.07
49.84
48.75
48.23
40.50 dmso
40.33 dmso
40.16 dmso
40.08 dmso
39.99 dmso
39.83 dmso
39.66 dmso
39.49 dmso
21.30
14.34
(4t)
f1 (ppm)

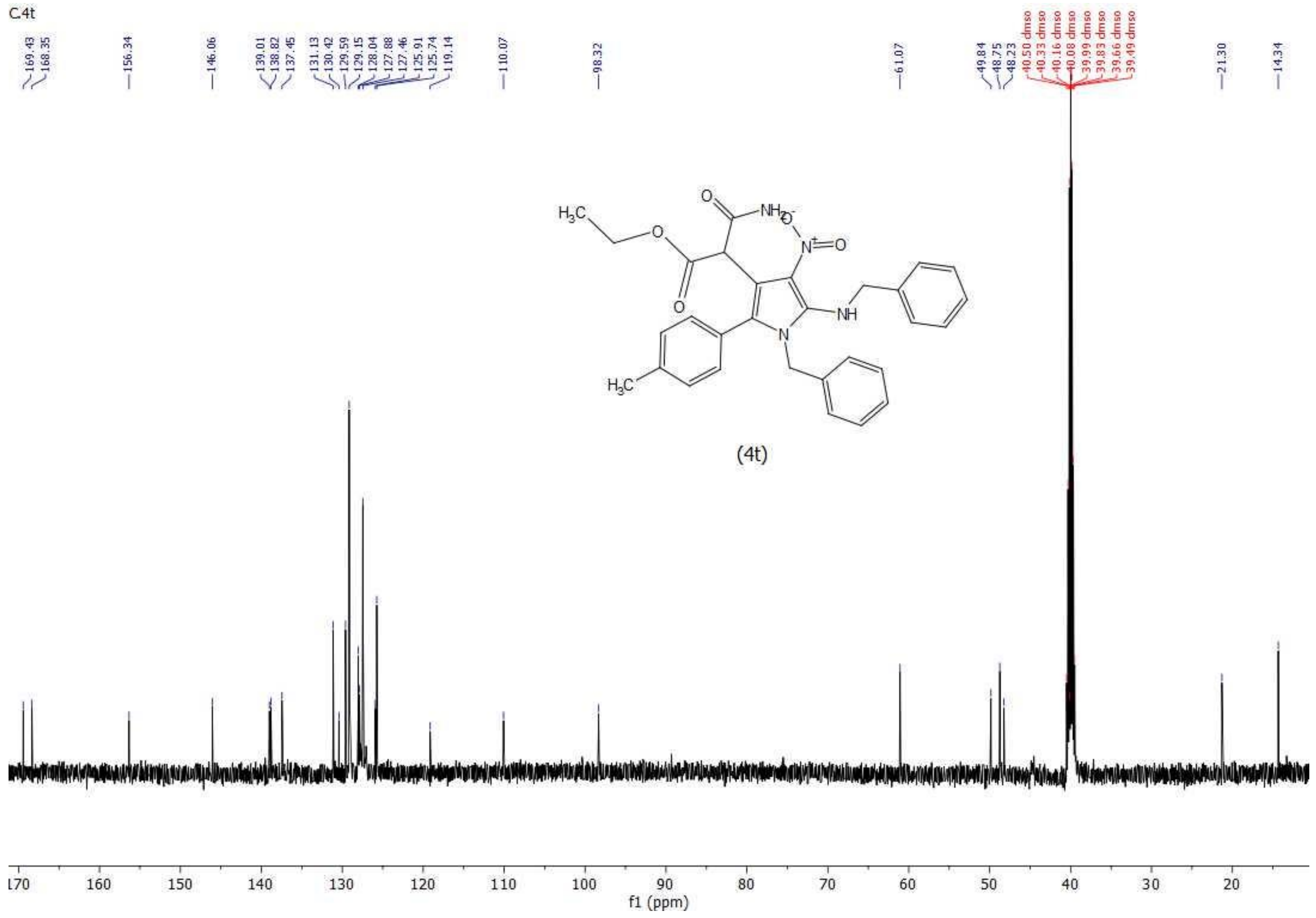
C.4t
169.43
168.35
156.34
146.06
139.01
138.82
137.45
131.13
130.42
129.59
129.15
128.04
127.88
127.46
125.91
125.74
119.14
110.07
98.32
61.07
49.84
48.75
48.23
40.50 dmso
40.33 dmso
40.16 dmso
40.08 dmso
39.99 dmso
39.83 dmso
39.66 dmso
39.49 dmso
21.30
14.34
(4t)
f1 (ppm)

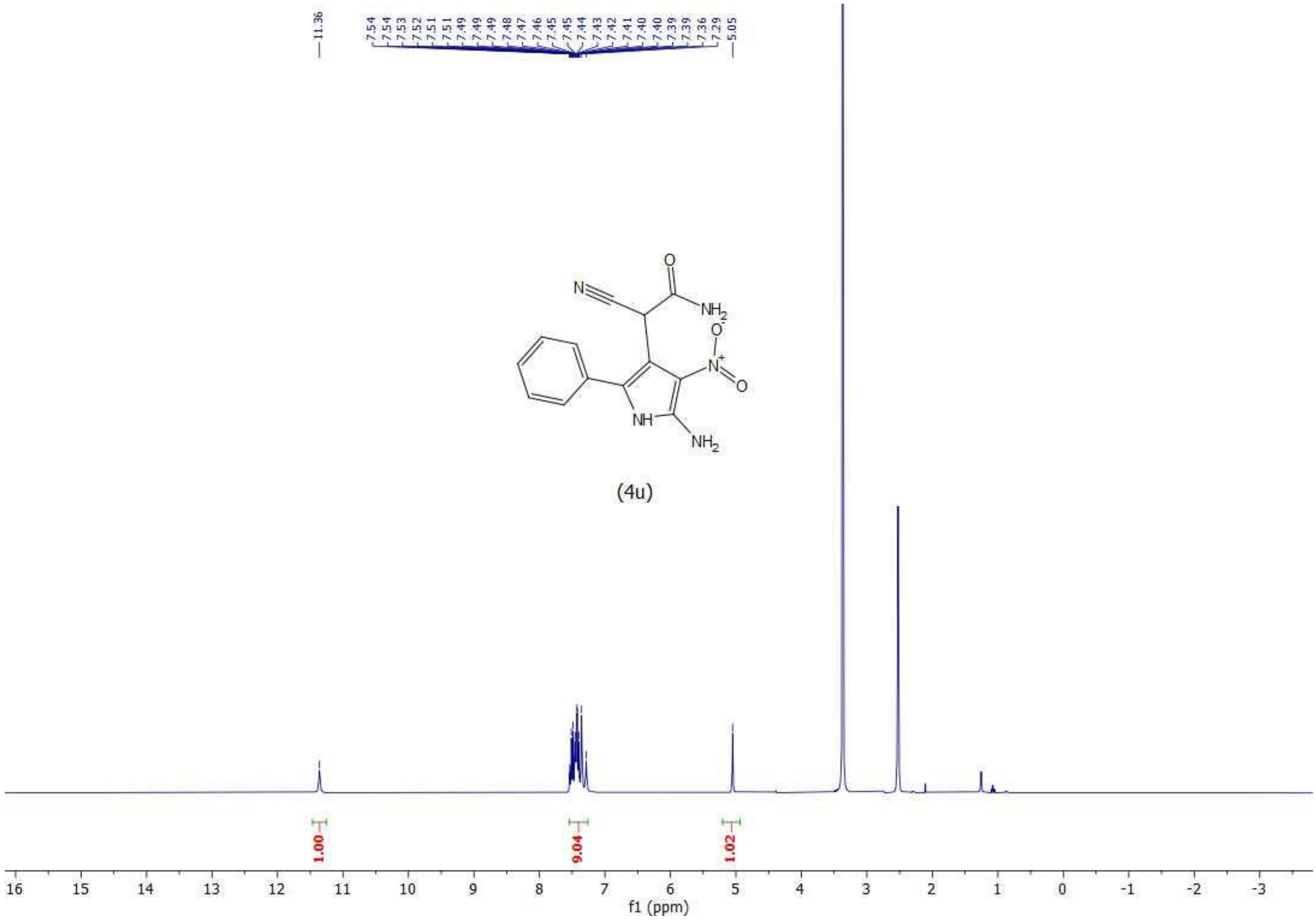

11.36
7.54
7.54
7.53
7.52
7.51
7.51
7.49
7.49
7.49
7.48
7.47
7.46
7.45
7.45
7.44
7.43
7.42
7.41
7.40
7.40
7.39
7.39
7.36
7.29
5.05
N
O
NH2
O-
N+
O
NH
NH2
(4u)
1.00
9.04
1.02
16
15
14
13
12
11
10
9
8
7
6
5
4
3
2
1
0
-1
-2
-3
f1 (ppm)

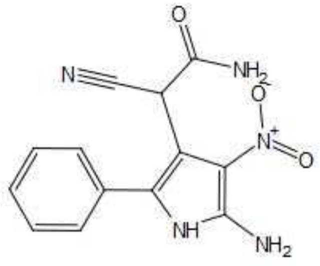

N
NH2
O
O
N+
O
NH
NH2

(4u

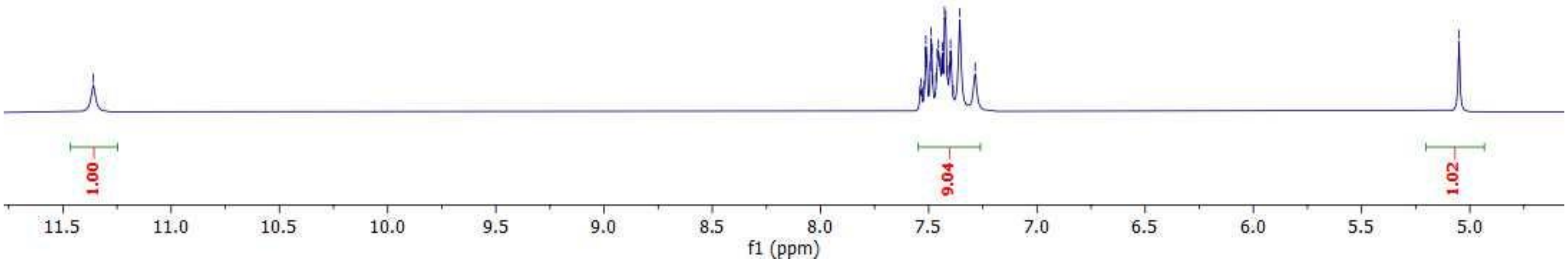

11.36
7.54
7.54
7.53
7.52
7.51
7.51
7.49
7.49
7.49
7.48
7.47
7.46
7.45
7.45
7.44
7.43
7.42
7.41
7.40
7.40
7.39
7.39
7.36
7.29
5.05
1.00
9.04
1.02
11.5
11.0
10.5
10.0
9.5
9.0
8.5
8.0
7.5
7.0
6.5
6.0
5.5
5.0
f1 (ppm)

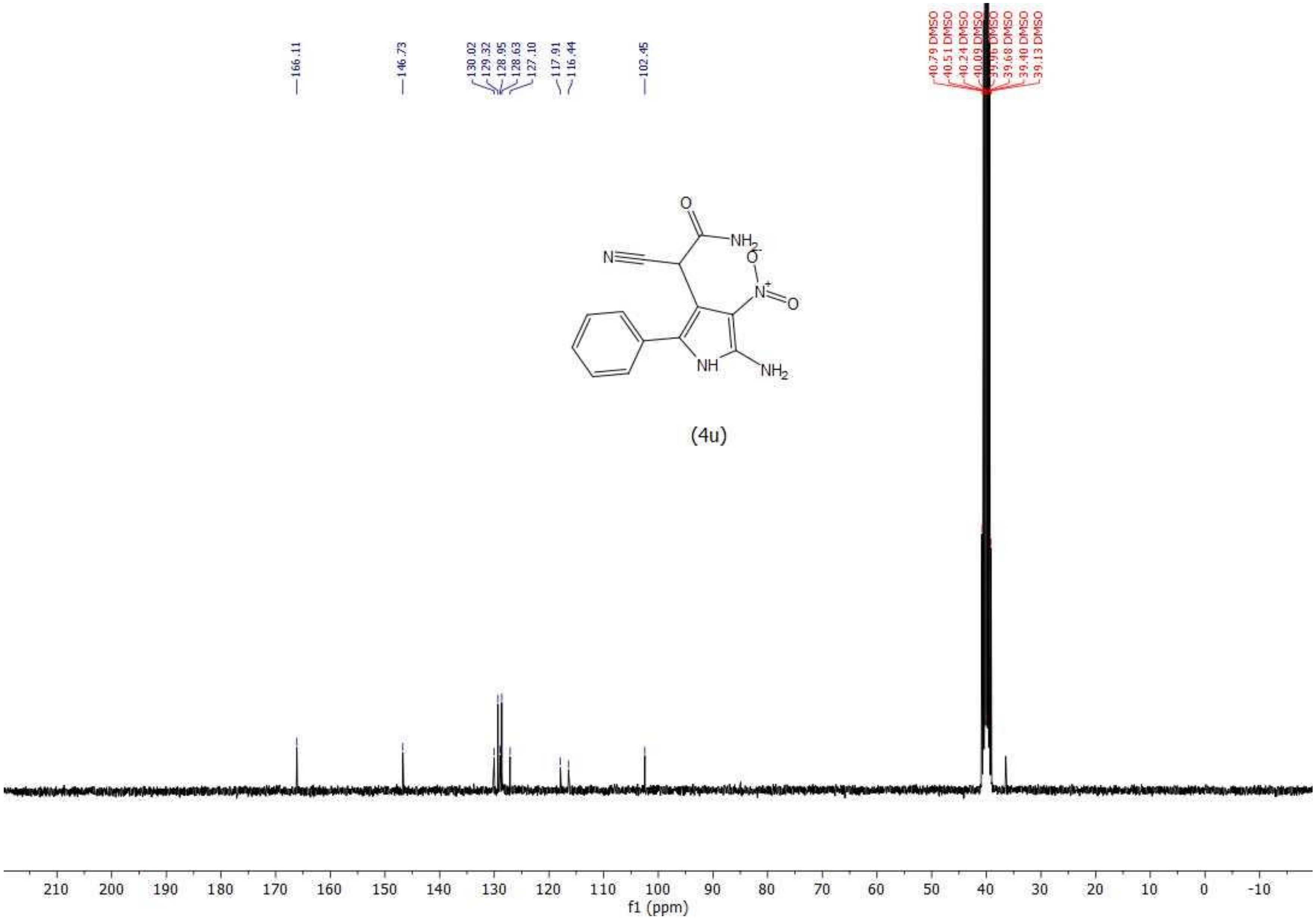
166.11
146.73
130.02
129.32
128.95
128.63
127.10
117.91
116.44
102.45
40.79 DMSO
40.51 DMSO
40.24 DMSO
40.09 DMSO
39.96 DMSO
39.68 DMSO
39.40 DMSO
39.13 DMSO
O
NH2
N
N+
O
O-
NH
NH2
(4u)
210
200
190
180
170
160
150
140
130
120
110
100
90
80
70
60
50
40
30
20
10
0
-10
f1 (ppm)

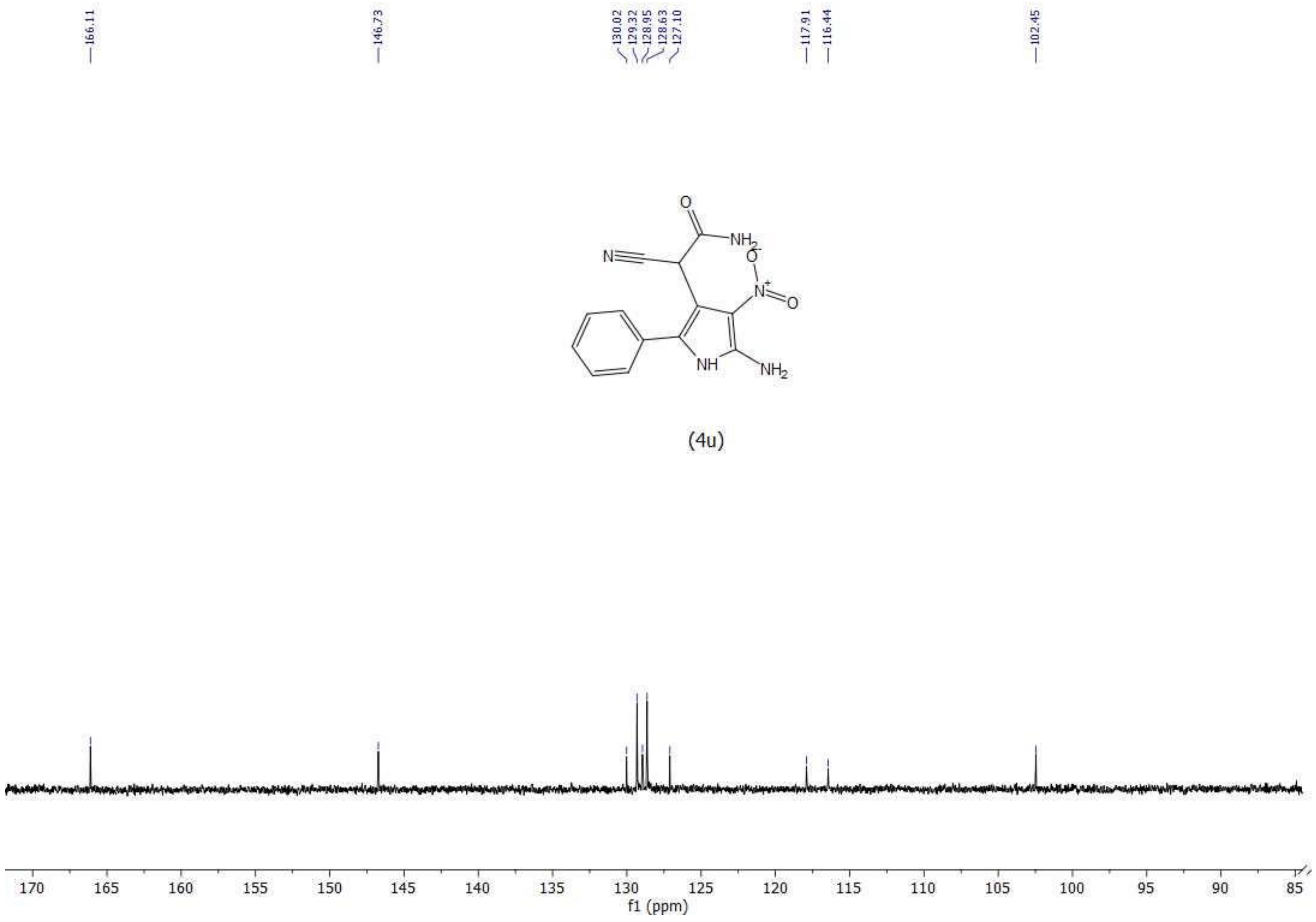
166.11
146.73
130.02
129.32
128.95
128.63
127.10
117.91
116.44
102.45
O
NH2
N
O-
N+
O
NH
NH2
(4u)
170
165
160
155
150
145
140
135
130
125
120
115
110
105
100
95
90
85
f1 (ppm)

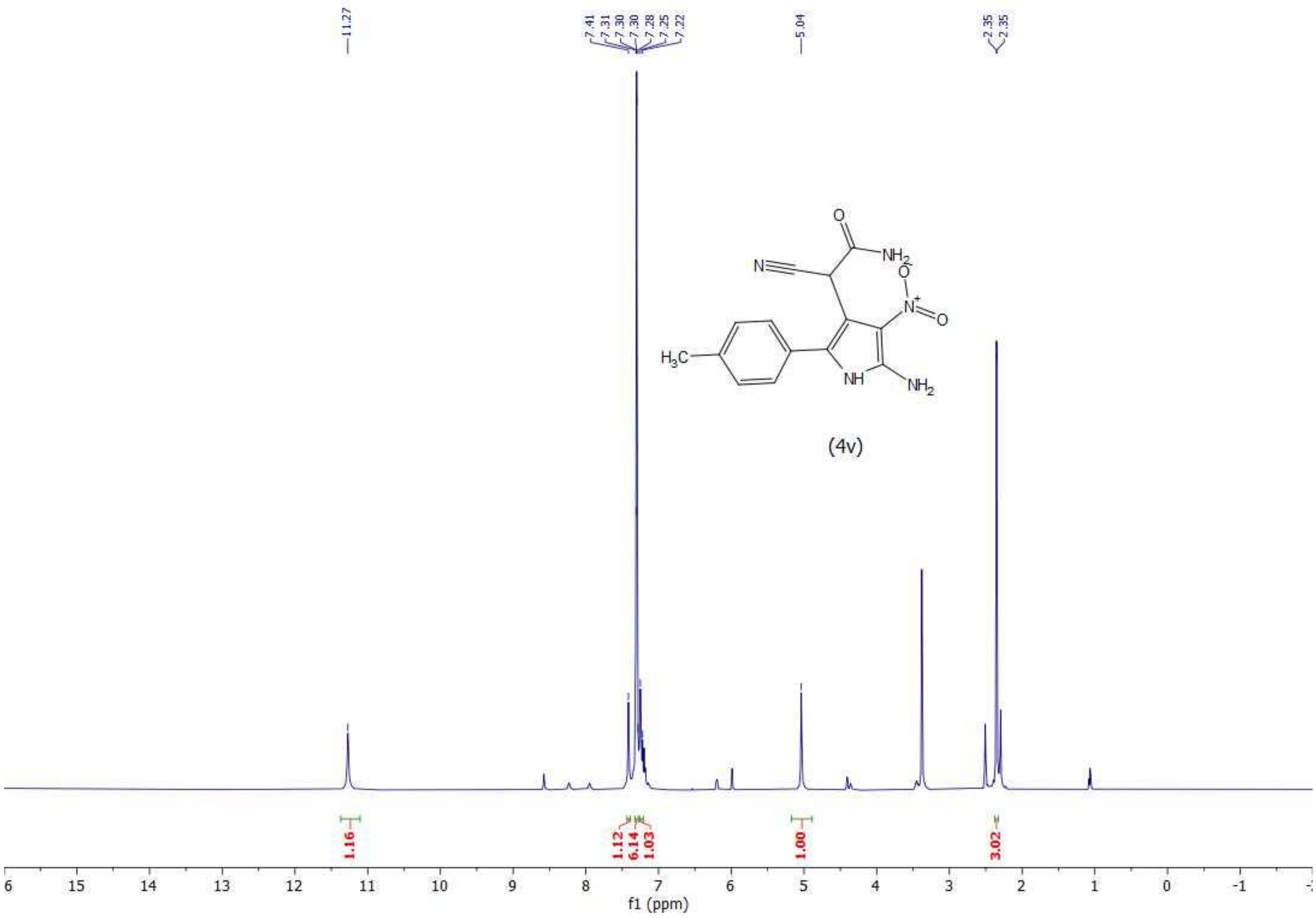
11.27
7.41
7.31
7.30
7.30
7.28
7.25
7.22
5.04
2.35
2.35
N
NH2
O
N+
O-
H3C
NH
NH2
1.16
1.12
6.14
1.03
1.00
3.02
f1 (ppm)

(4v)

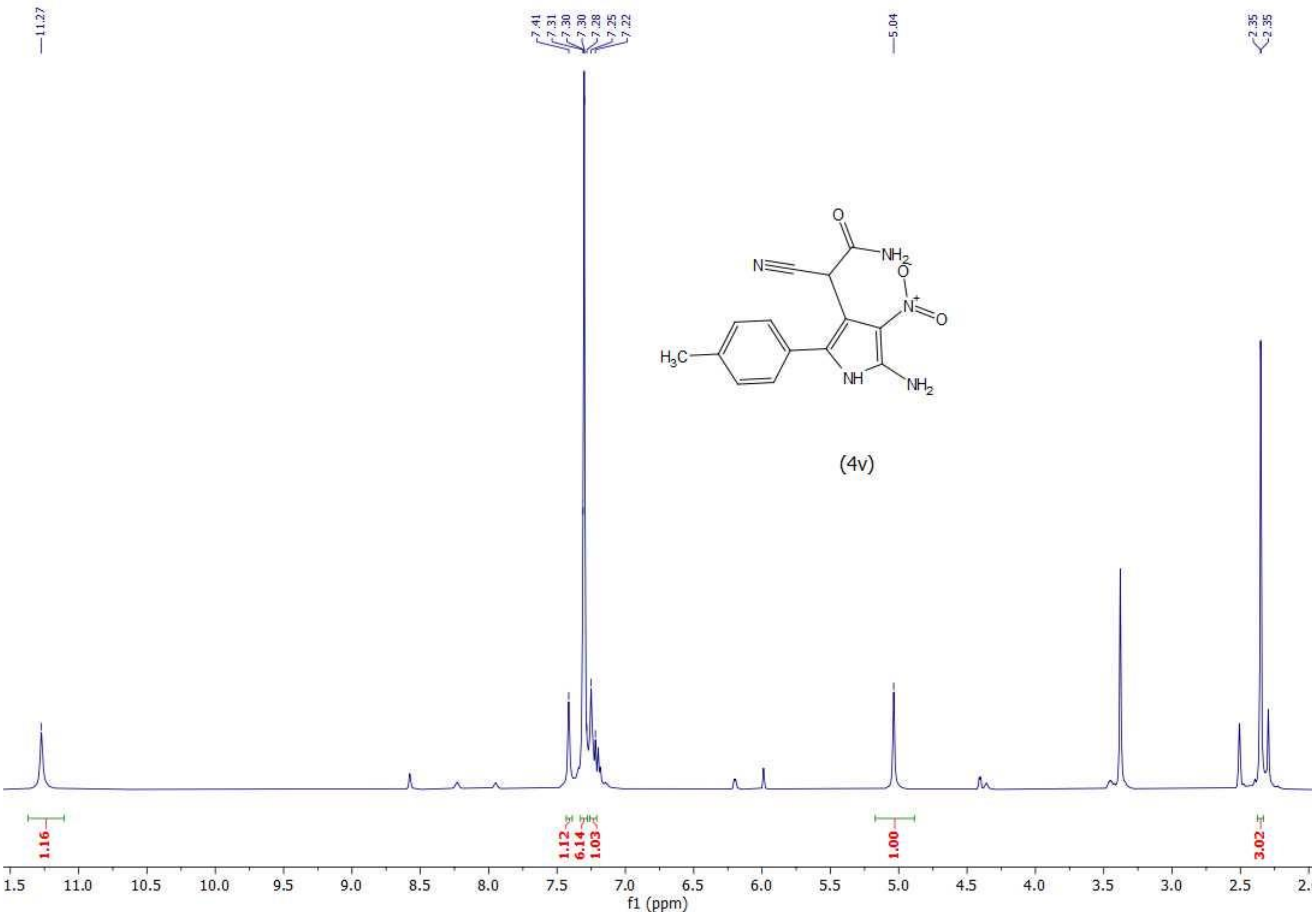
11.27
7.41
7.31
7.30
7.30
7.28
7.25
7.22
5.04
2.35
2.35
1.16
1.12
6.14
1.03
1.00
3.02
f1 (ppm)

(4v)

C4v

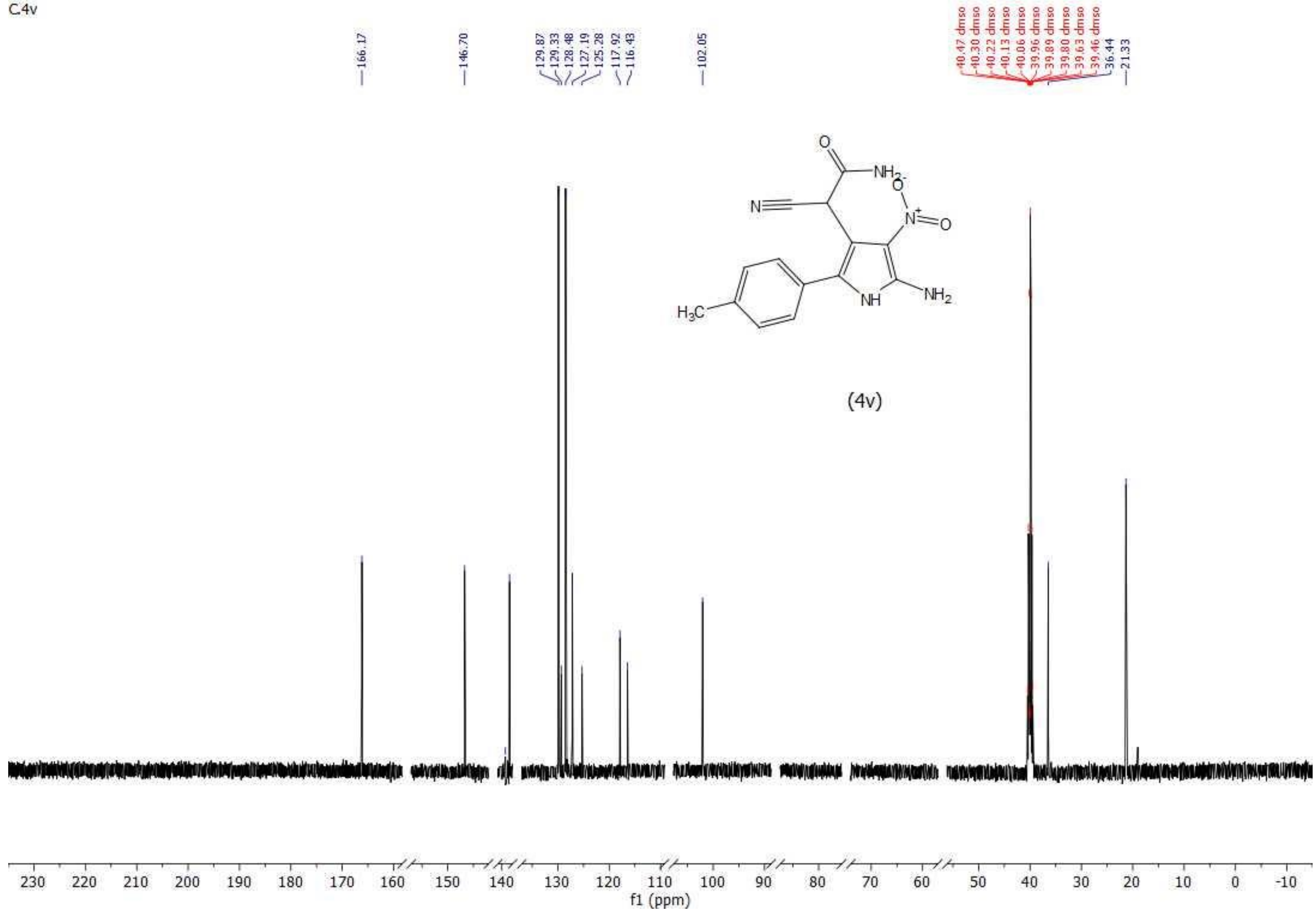
166.17
146.70
129.87
129.33
128.48
127.19
125.28
117.92
116.43
102.05
40.47 dmso
40.30 dmso
40.22 dmso
40.13 dmso
40.06 dmso
39.96 dmso
39.89 dmso
39.80 dmso
39.63 dmso
39.46 dmso
36.44
21.33
O
NH2
O
N
O
N
NH
NH2
H3C
230 220 210 200 190 180 170 160 150 140 130 120 110 100 90 80 70 60 50 40 30 20 10 0 -10
f1 (ppm)

(4v)

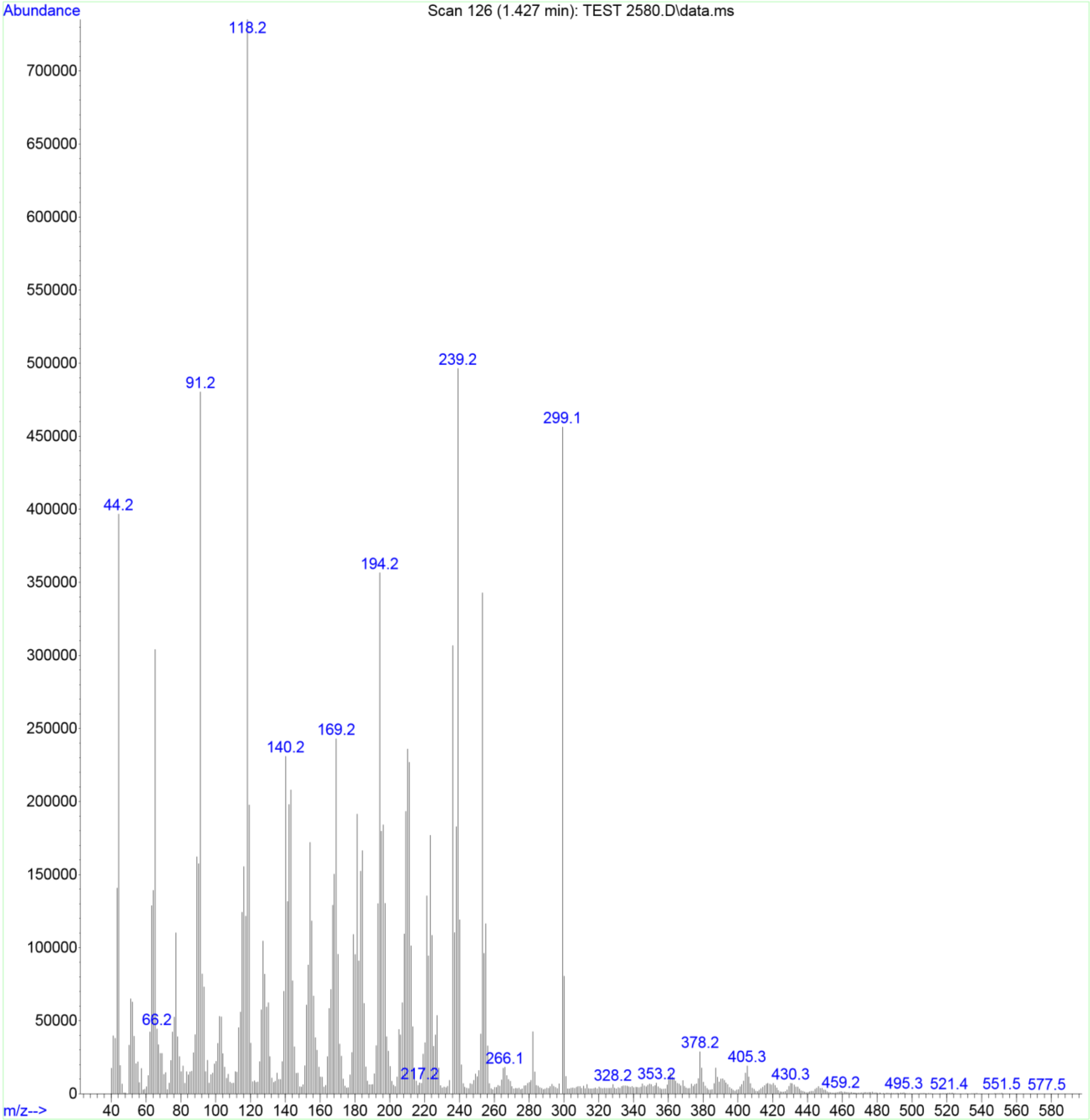

$C_{14}H_{13}N_5O_3$

(299/2)

(4v)

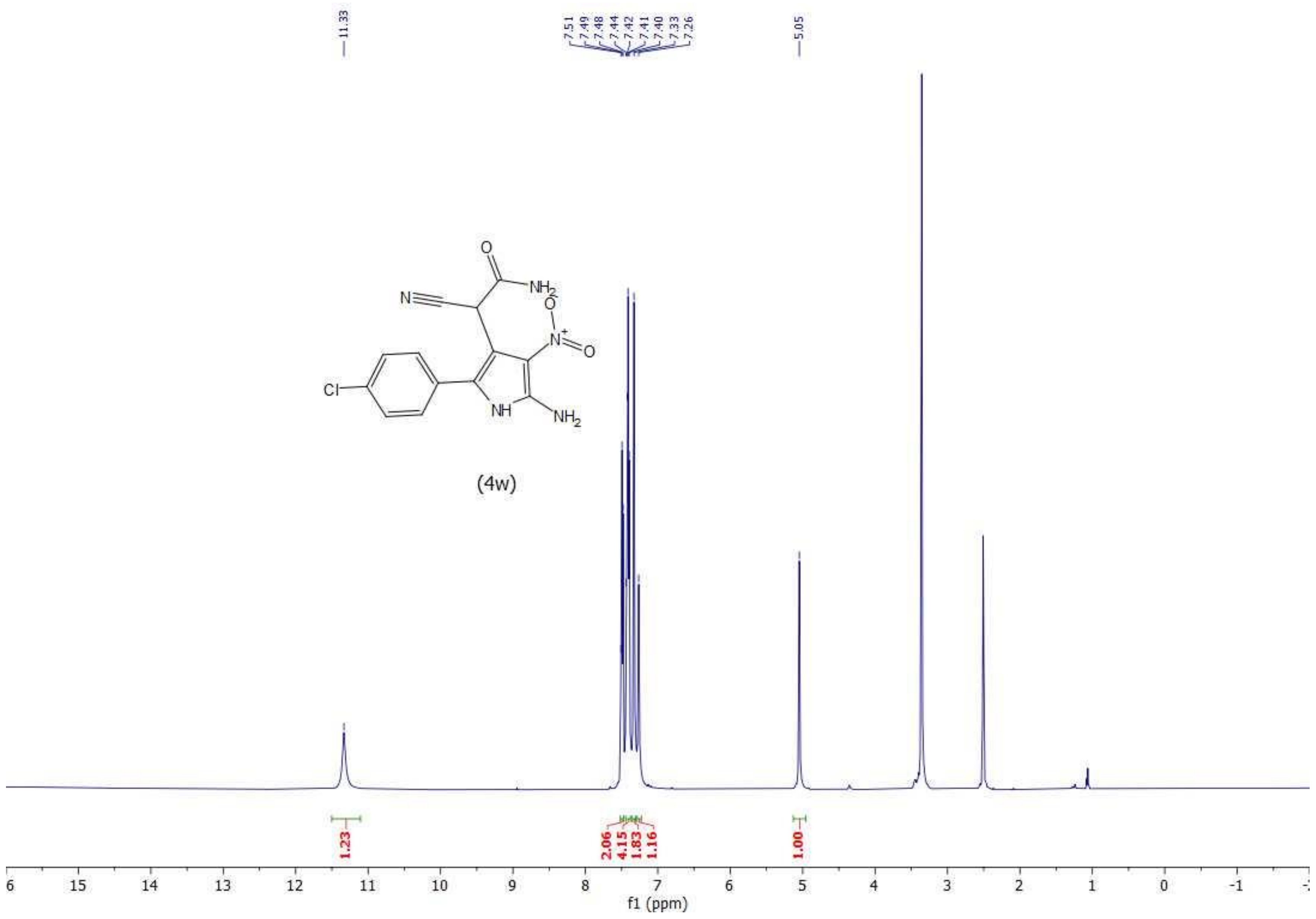
11.33
7.51
7.49
7.48
7.44
7.42
7.41
7.40
7.33
7.26
5.05
(4w)
1.23
2.06
4.15
1.83
1.16
1.00
16
15
14
13
12
11
10
9
8
7
6
5
4
3
2
1
0
-1
-2
f1 (ppm)

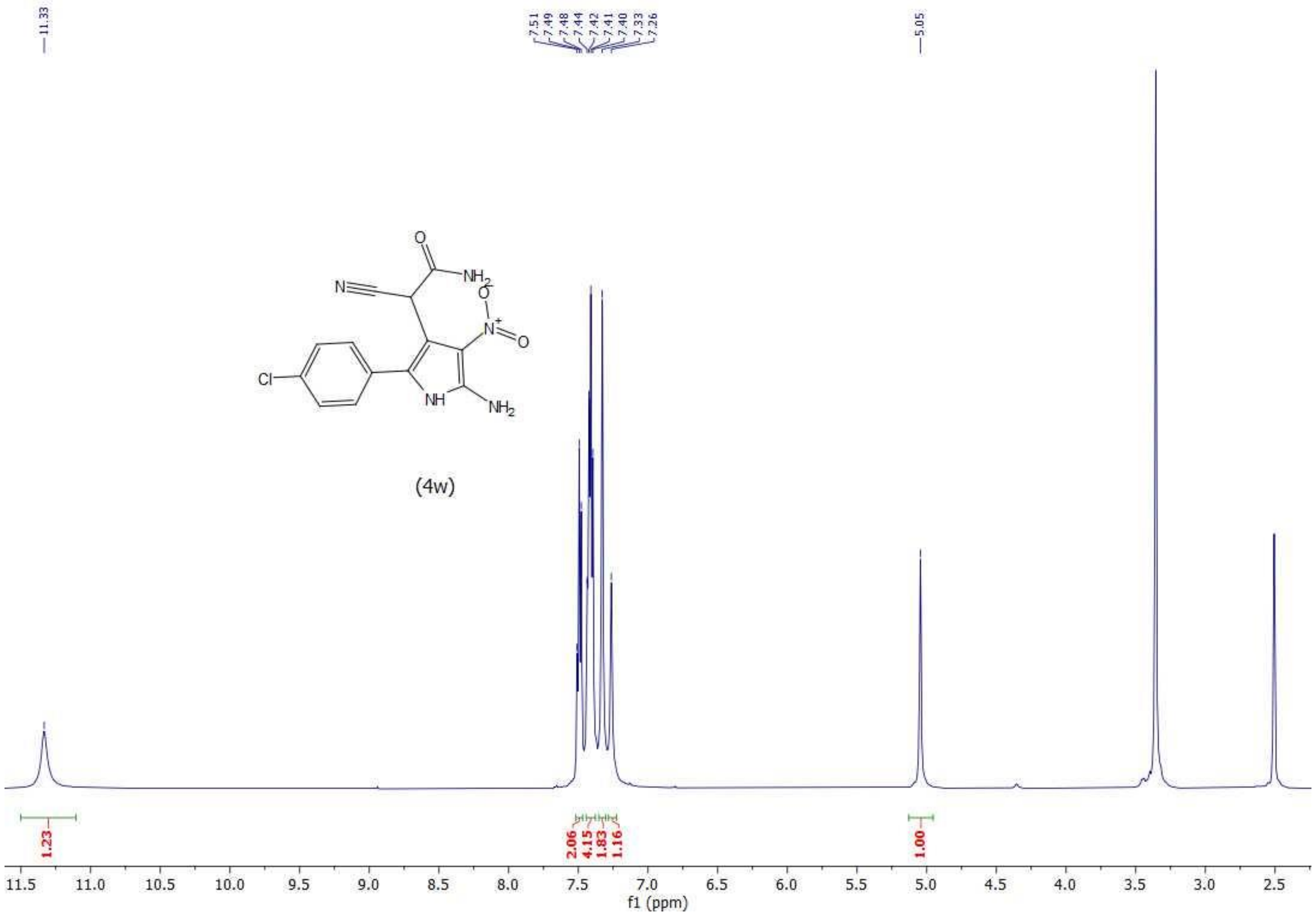
11.33
7.51
7.49
7.48
7.44
7.42
7.41
7.40
7.33
7.26
5.05
NH
NH2
Cl
N
O
(4w)
1.23
2.06
4.15
1.83
1.16
1.00
11.5
11.0
10.5
10.0
9.5
9.0
8.5
8.0
7.5
7.0
6.5
6.0
5.5
5.0
4.5
4.0
3.5
3.0
2.5
f1 (ppm)

C4w

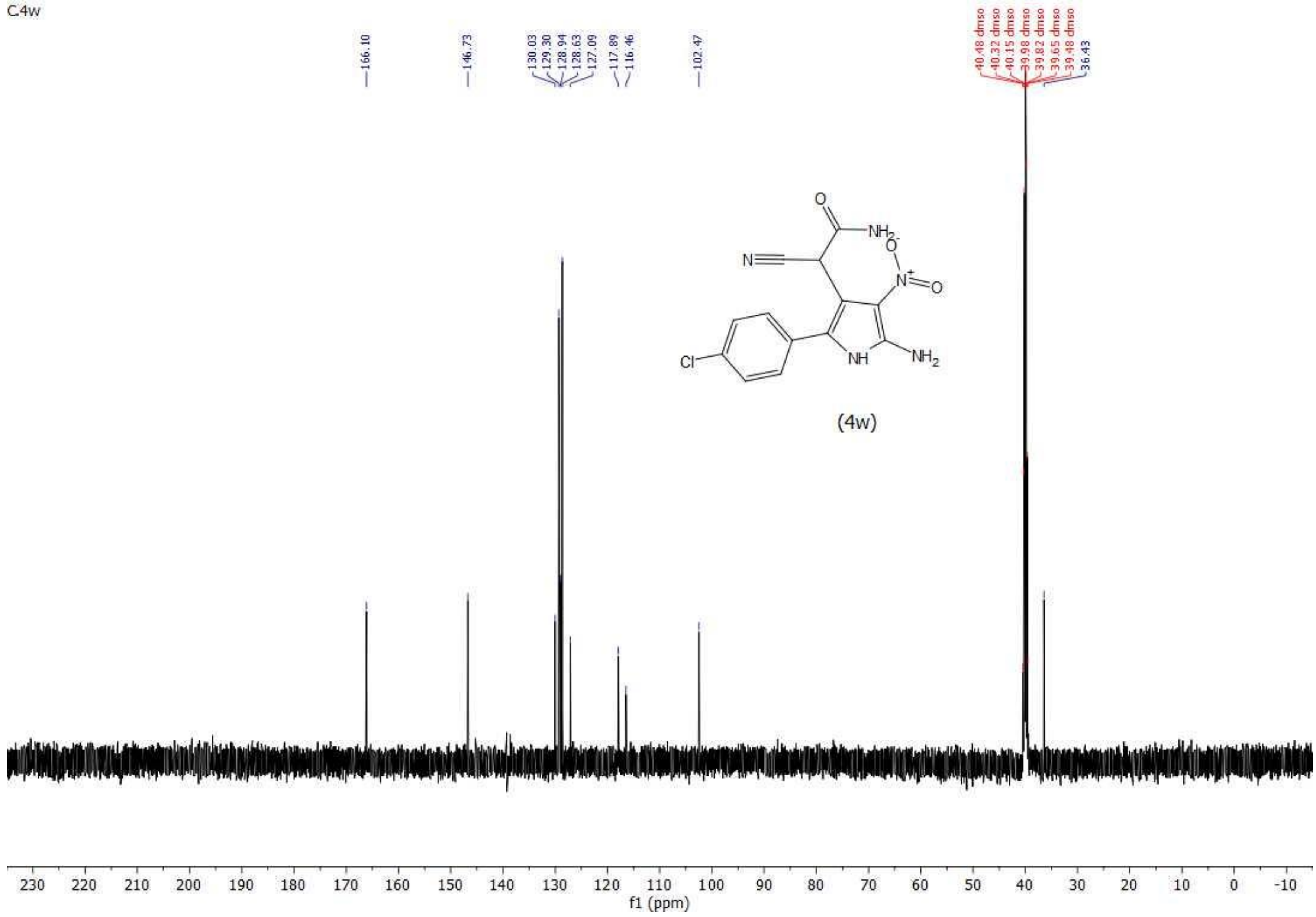

166.10
146.73
130.03
129.30
128.94
128.63
127.09
117.89
116.46
102.47
40.48 dmso
40.32 dmso
40.15 dmso
39.98 dmso
39.82 dmso
39.65 dmso
39.48 dmso
36.43
O
NH2
N
O
N+
O
Cl
NH
NH2
(4w)
230 220 210 200 190 180 170 160 150 140 130 120 110 100 90 80 70 60 50 40 30 20 10 0 -10
f1 (ppm)

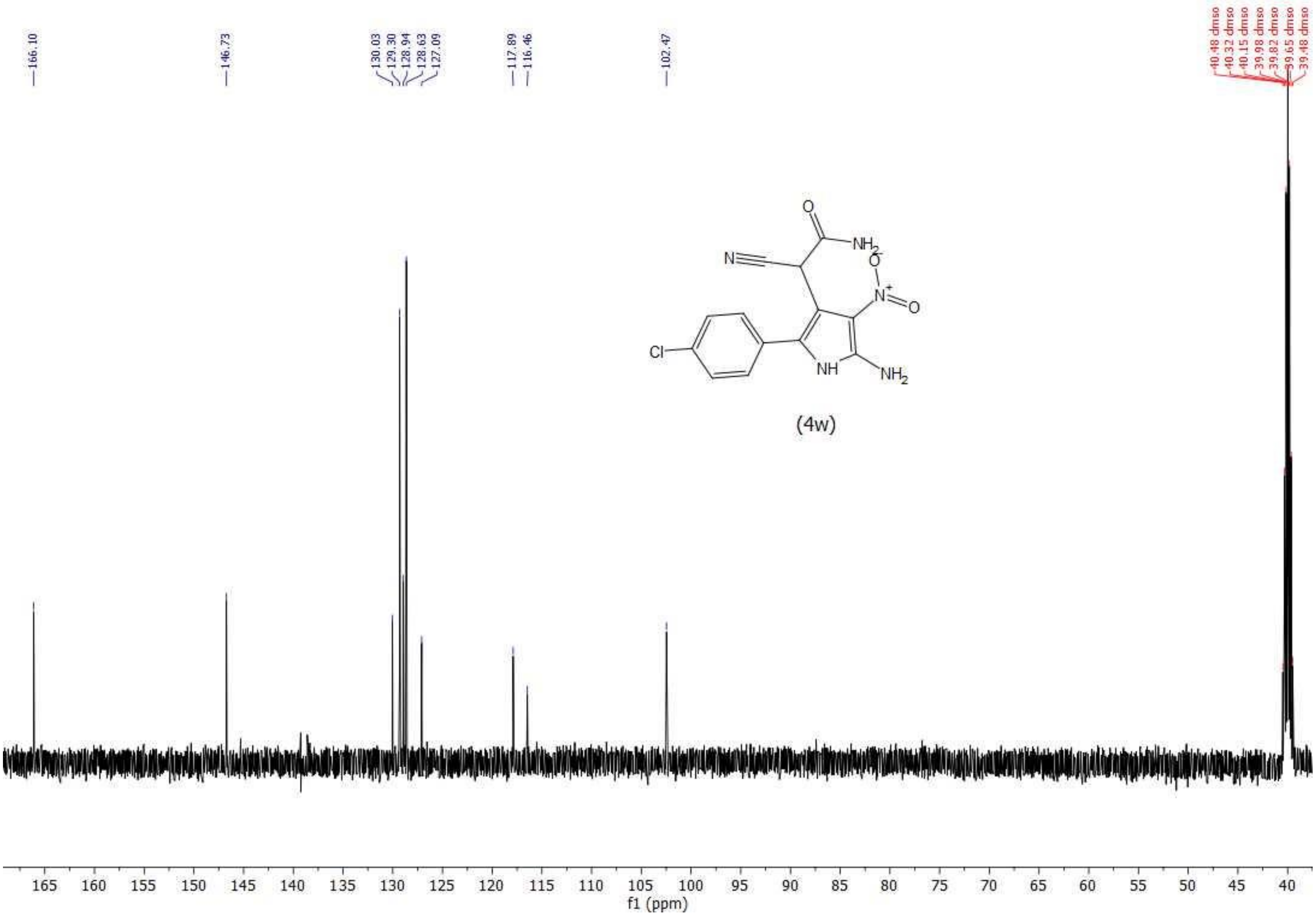
166.10
146.73
130.03
129.30
128.94
128.63
127.09
117.89
116.46
102.47
40.48 dmso
40.32 dmso
40.15 dmso
39.98 dmso
39.82 dmso
39.65 dmso
39.48 dmso
O
NH2
N
O
N
O
Cl
NH
NH2
165
160
155
150
145
140
135
130
125
120
115
110
105
100
95
90
85
80
75
70
65
60
55
50
45
40
f1 (ppm)
(4w)

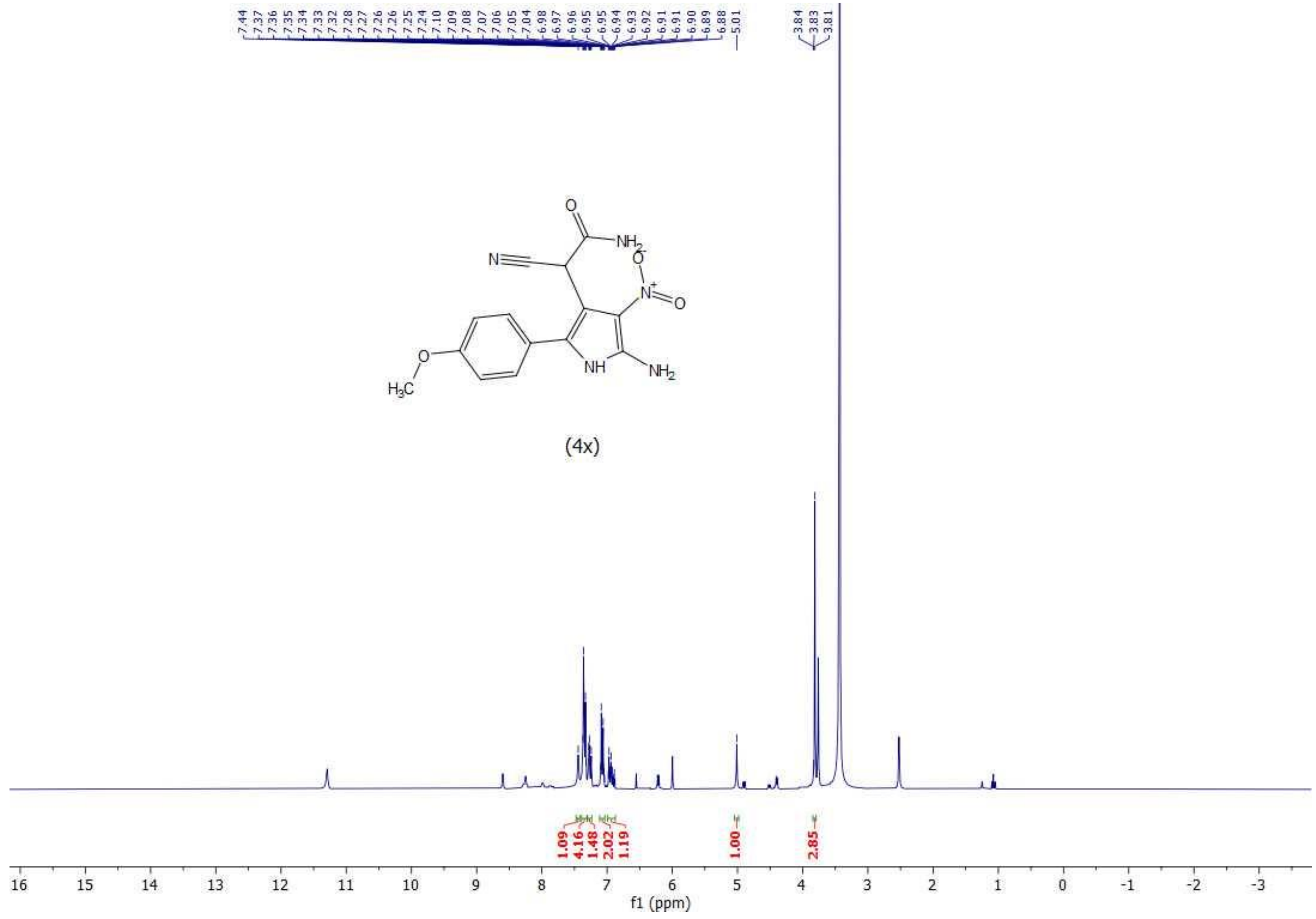
7.44
7.37
7.36
7.35
7.34
7.33
7.32
7.28
7.27
7.26
7.26
7.25
7.24
7.10
7.09
7.08
7.07
7.06
7.05
7.04
6.98
6.97
6.96
6.95
6.95
6.94
6.93
6.92
6.91
6.91
6.90
6.89
6.88
5.01
3.84
3.83
3.81
(4x)
1.09
4.16
1.48
2.02
1.19
1.00
2.85
f1 (ppm)

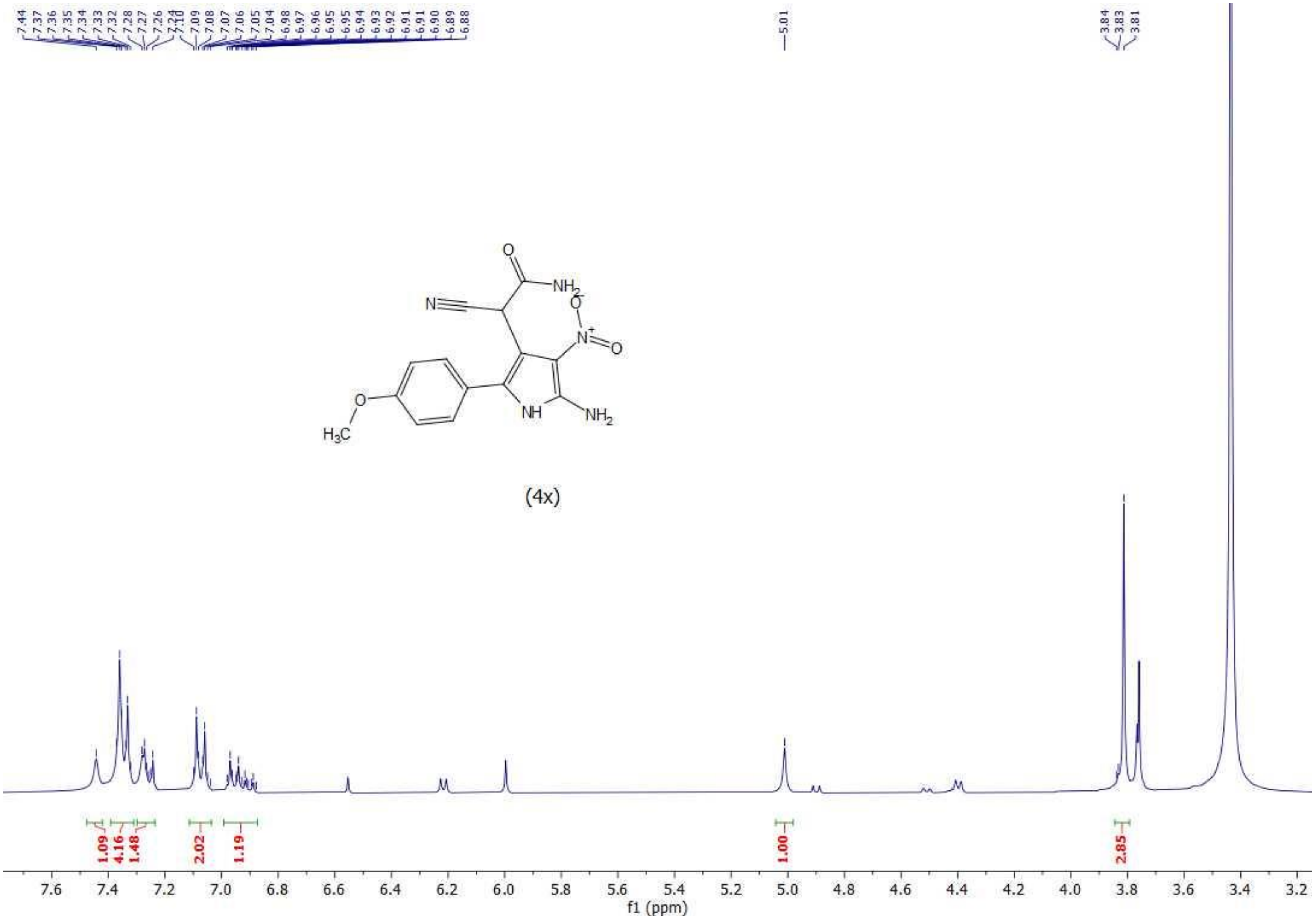
7.44
7.37
7.36
7.35
7.34
7.33
7.32
7.28
7.27
7.26
7.24
7.10
7.09
7.08
7.07
7.06
7.05
7.04
6.98
6.97
6.96
6.95
6.95
6.94
6.93
6.92
6.91
6.91
6.90
6.89
6.88
5.01
3.84
3.83
3.81
O
NH2
N
O-
N+
O
NH
NH2
O
H3C
(4x)
1.09
4.16
1.48
2.02
1.19
1.00
2.85
7.6
7.4
7.2
7.0
6.8
6.6
6.4
6.2
6.0
5.8
5.6
5.4
5.2
5.0
4.8
4.6
4.4
4.2
4.0
3.8
3.6
3.4
3.2
f1 (ppm)

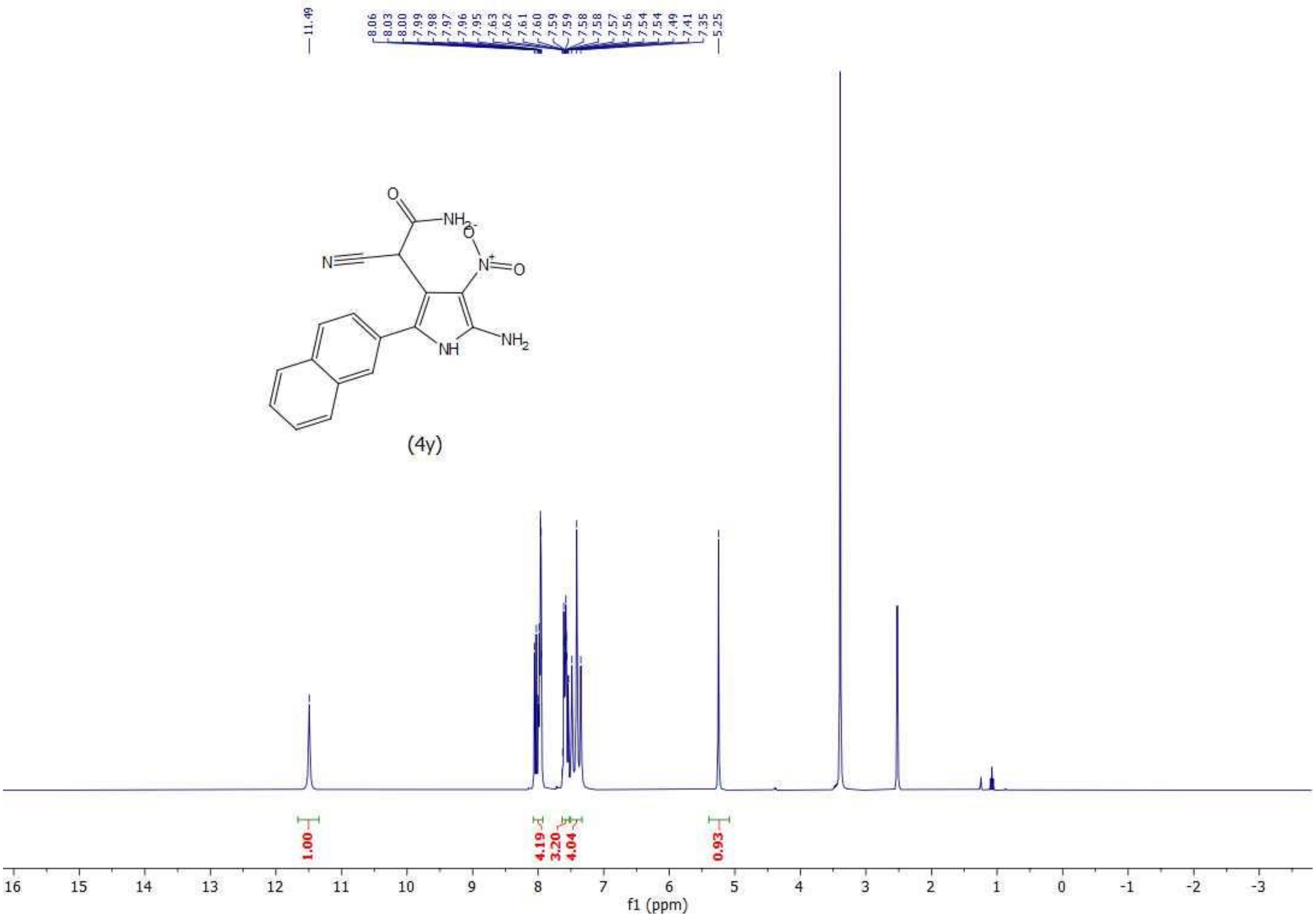

11.49
8.06 8.03 8.00 7.99 7.98 7.97 7.96 7.95 7.63 7.62 7.61 7.60 7.59 7.59 7.58 7.58 7.57 7.56 7.54 7.54 7.49 7.41 7.35
5.25
1.00
4.19
3.20
4.04
0.93
16 15 14 13 12 11 10 9 8 7 6 5 4 3 2 1 0 -1 -2 -3
f1 (ppm)

(4y)

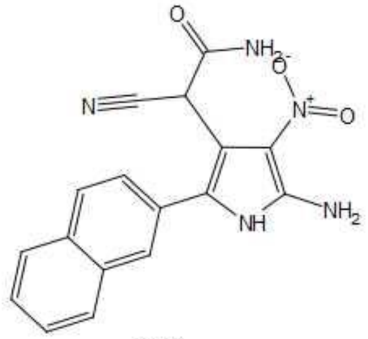

(4y)

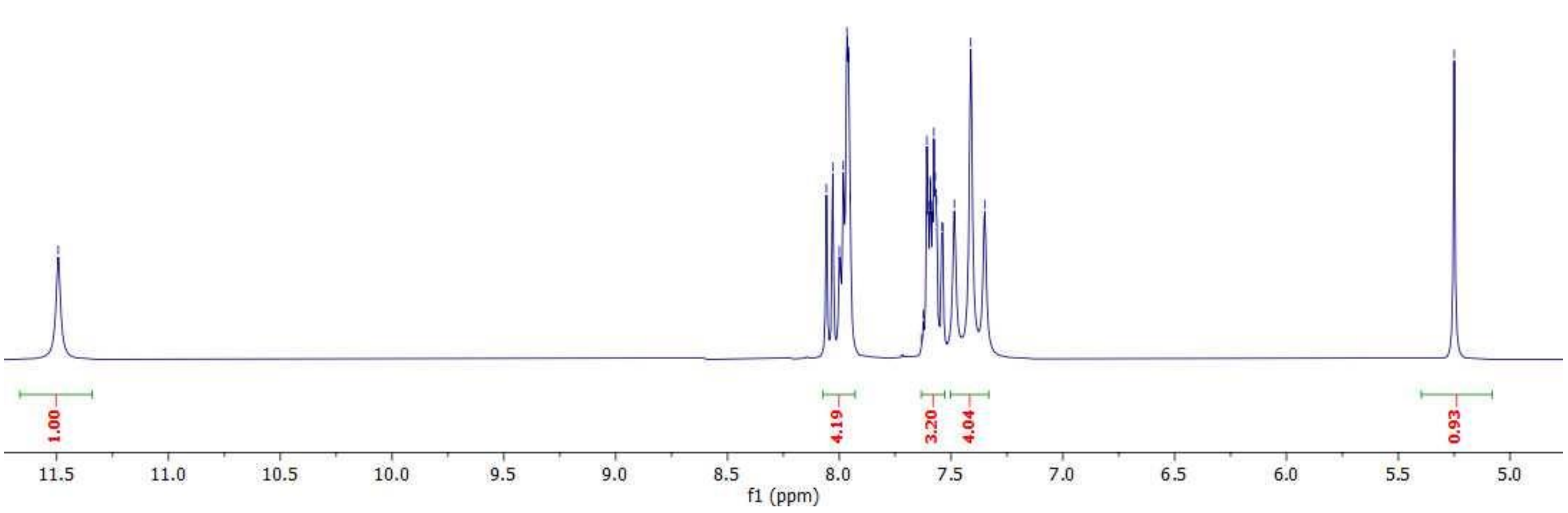

11.49
8.06
8.03
8.00
7.99
7.98
7.97
7.96
7.95
7.63
7.62
7.61
7.60
7.59
7.59
7.58
7.58
7.57
7.56
7.54
7.54
7.49
7.41
7.35
5.25
1.00
4.19
3.20
4.04
0.93
11.5
11.0
10.5
10.0
9.5
9.0
8.5
8.0
7.5
7.0
6.5
6.0
5.5
5.0
f1 (ppm)

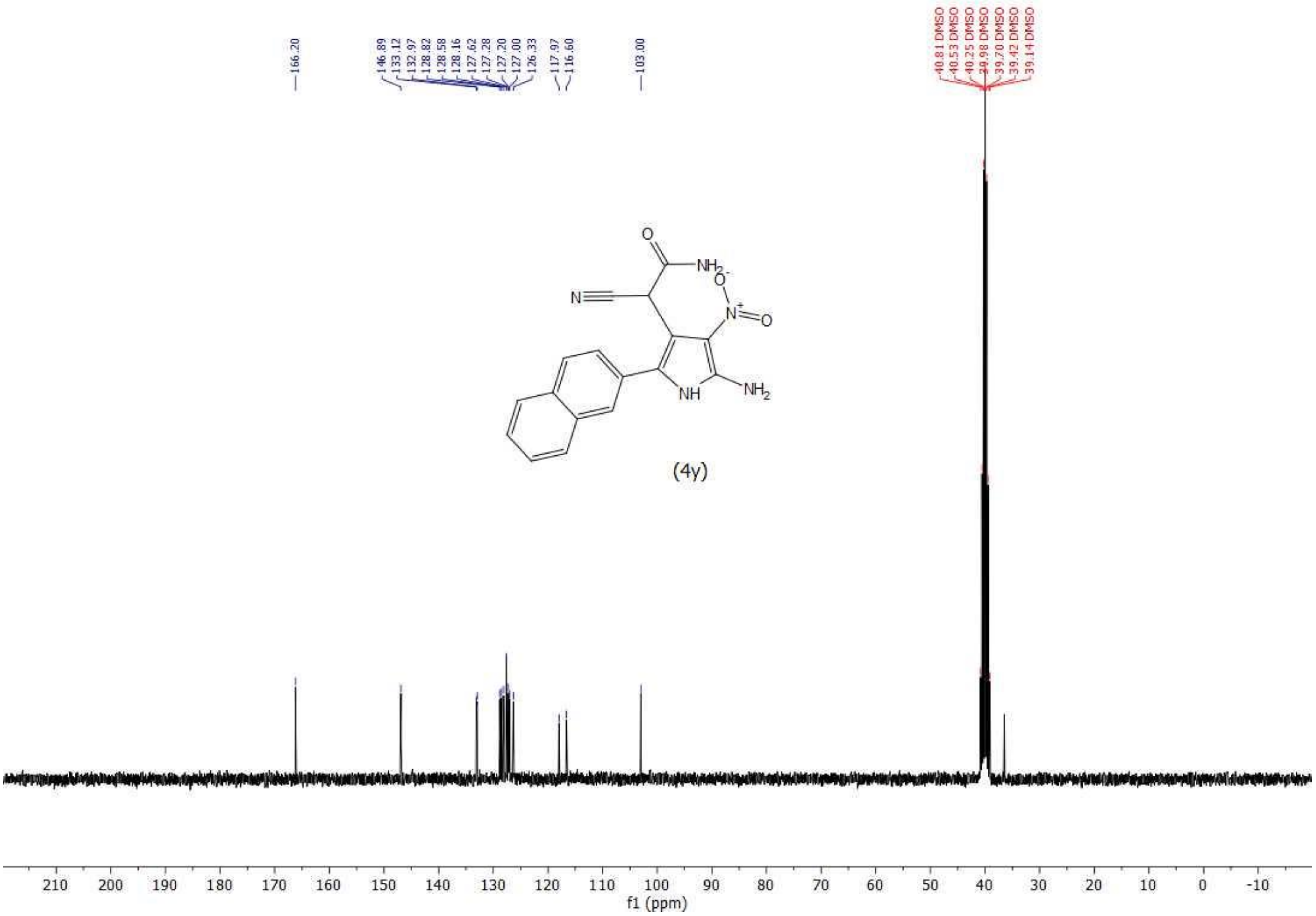

166.20
146.89
133.12
132.97
128.82
128.58
128.16
127.62
127.28
127.20
127.00
126.33
117.97
116.60
103.00
40.81 DMSO
40.53 DMSO
40.25 DMSO
39.98 DMSO
39.70 DMSO
39.42 DMSO
39.14 DMSO
(4y)
210 200 190 180 170 160 150 140 130 120 110 100 90 80 70 60 50 40 30 20 10 0 -10
f1 (ppm)

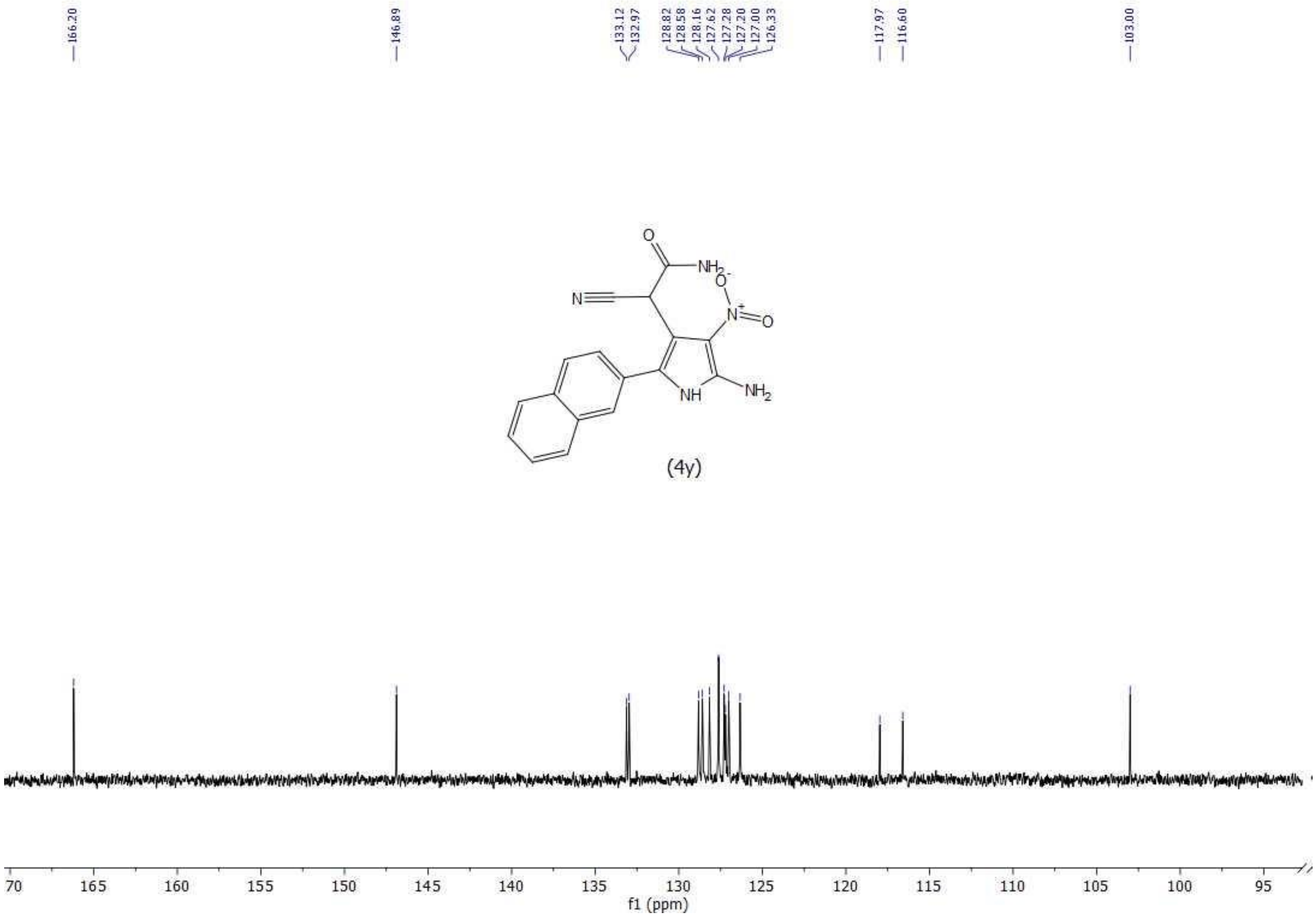
166.20
146.89
133.12
132.97
128.82
128.58
128.16
127.62
127.28
127.20
127.00
126.33
117.97
116.60
103.00
NH2
O
N
NH
f1 (ppm)

(4y)

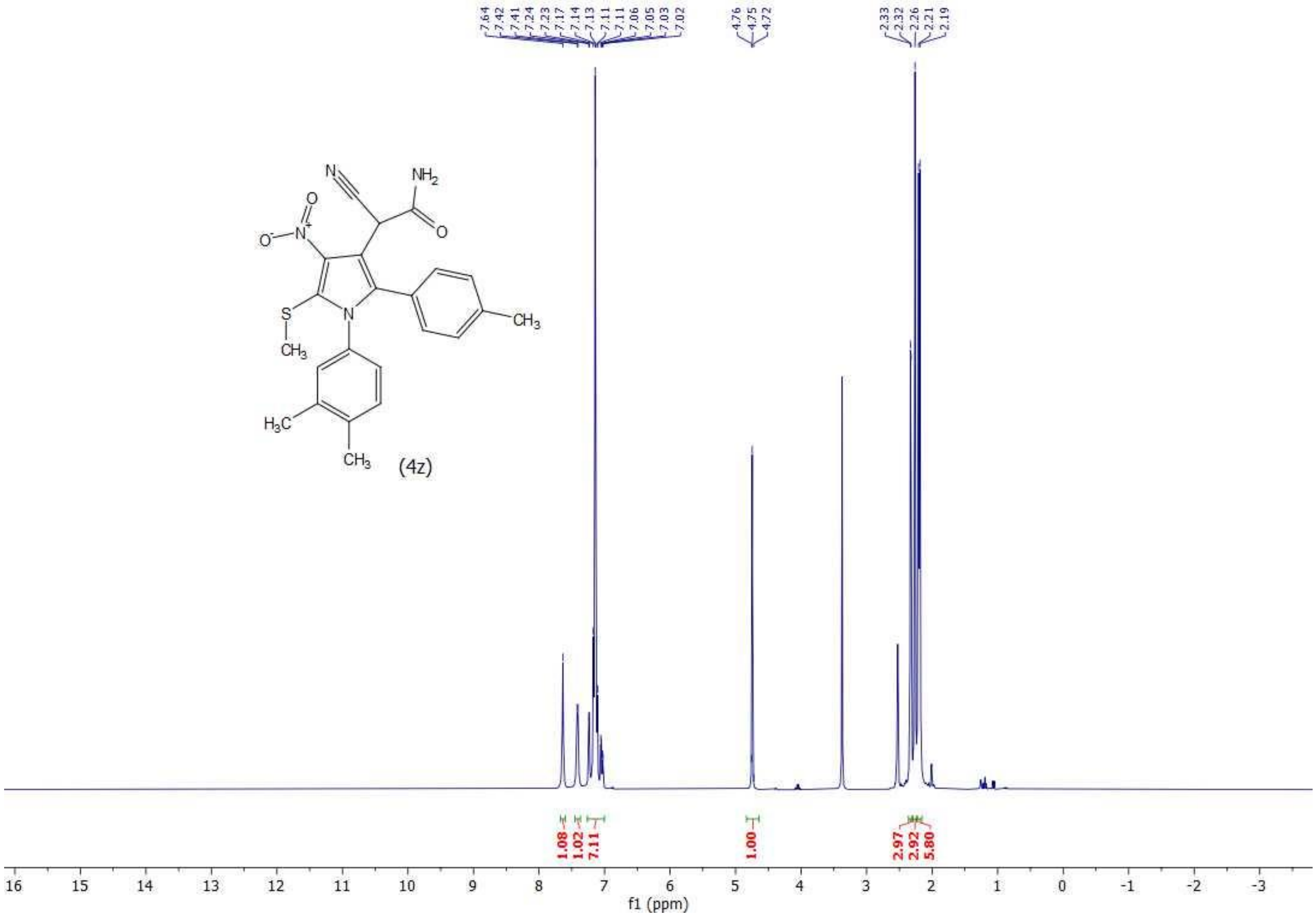

7.64
7.42
7.41
7.24
7.23
7.17
7.14
7.13
7.11
7.11
7.06
7.05
7.03
7.02
4.76
4.75
4.72
2.33
2.32
2.26
2.21
2.19
NH2
O
N
O-
N+
S
CH3
N
CH3
H3C
CH3
(4z)
1.08
1.02
7.11
1.00
2.97
2.92
5.80
16 15 14 13 12 11 10 9 8 7 6 5 4 3 2 1 0 -1 -2 -3
f1 (ppm)

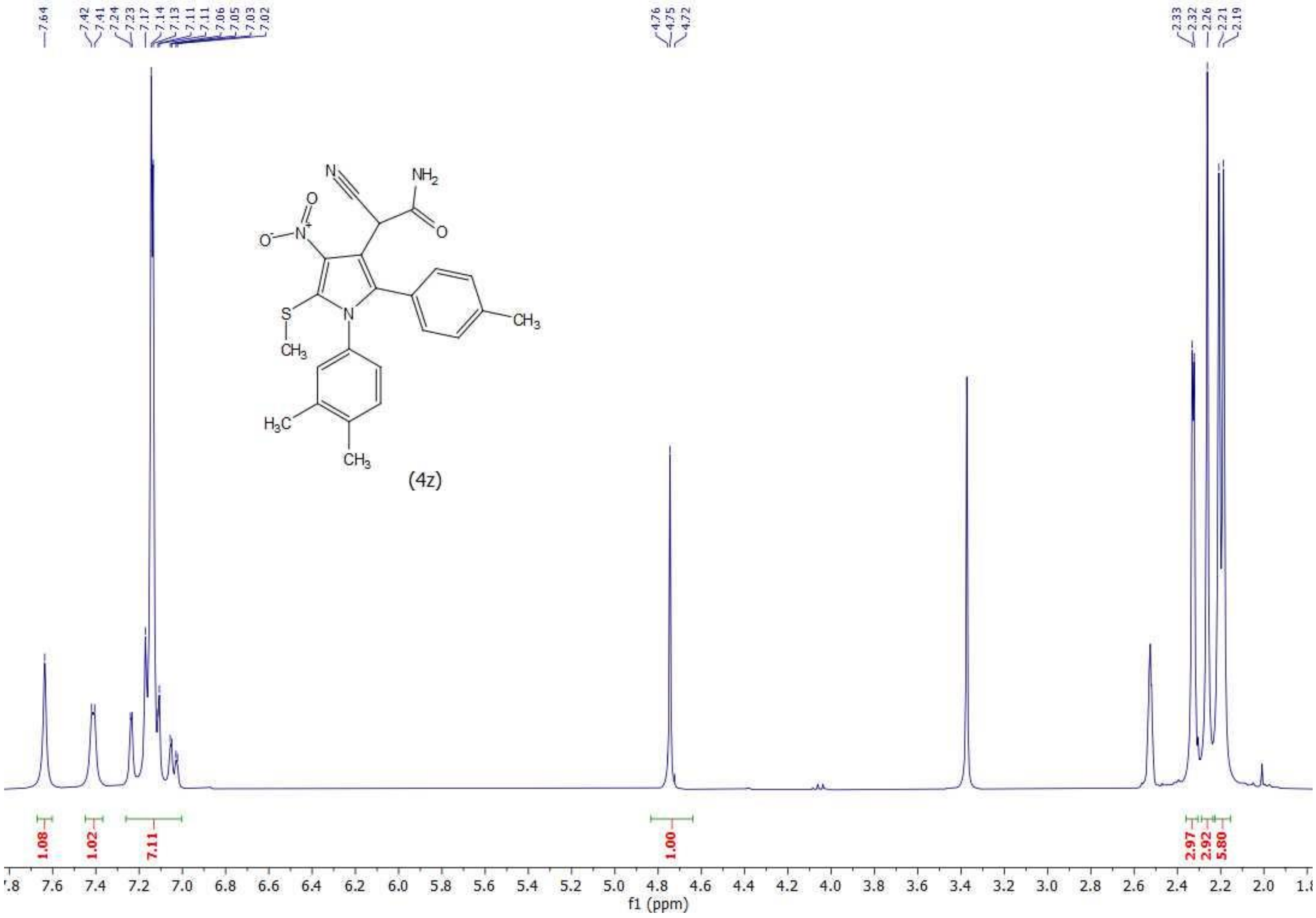
7.64
7.42
7.41
7.24
7.23
7.17
7.14
7.13
7.11
7.11
7.06
7.05
7.03
7.02
4.76
4.75
4.72
2.33
2.32
2.26
2.21
2.19
N
NH_2
O
O^-
N^+
O
S
N
CH_3
CH_3
H_3C
CH_3
(4z)
1.08
1.02
7.11
1.00
2.97
2.92
5.80
f1 (ppm)

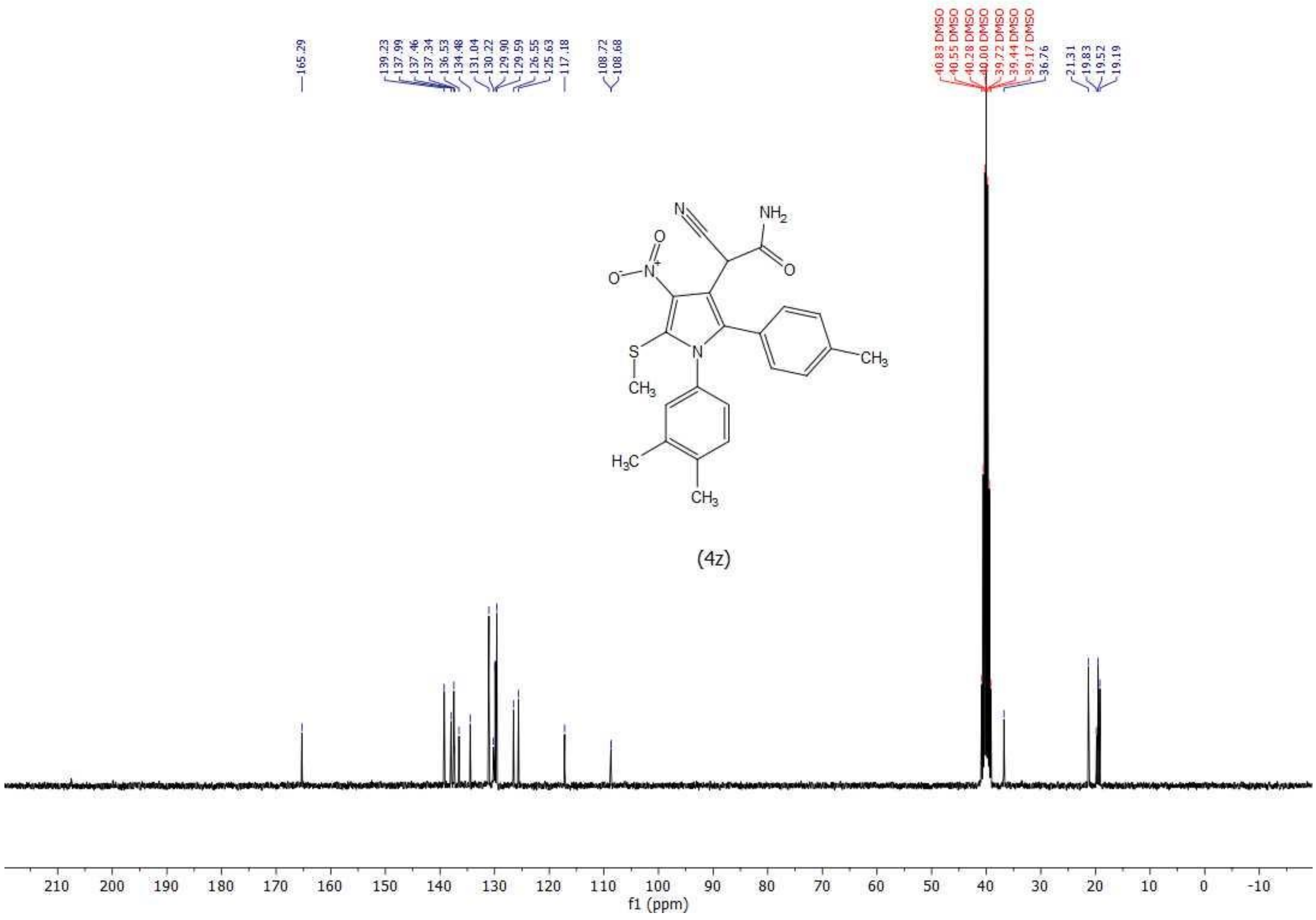

165.29
139.23
137.99
137.46
137.34
136.53
134.48
131.04
130.22
129.90
129.59
126.55
125.63
117.18
108.72
108.68
40.83 DMSO
40.55 DMSO
40.28 DMSO
40.00 DMSO
39.72 DMSO
39.44 DMSO
39.17 DMSO
36.76
21.31
19.83
19.52
19.19
NH2
N
O
O-
N+
S
CH3
H3C
f1 (ppm)
210 200 190 180 170 160 150 140 130 120 110 100 90 80 70 60 50 40 30 20 10 0 -10

(4z)

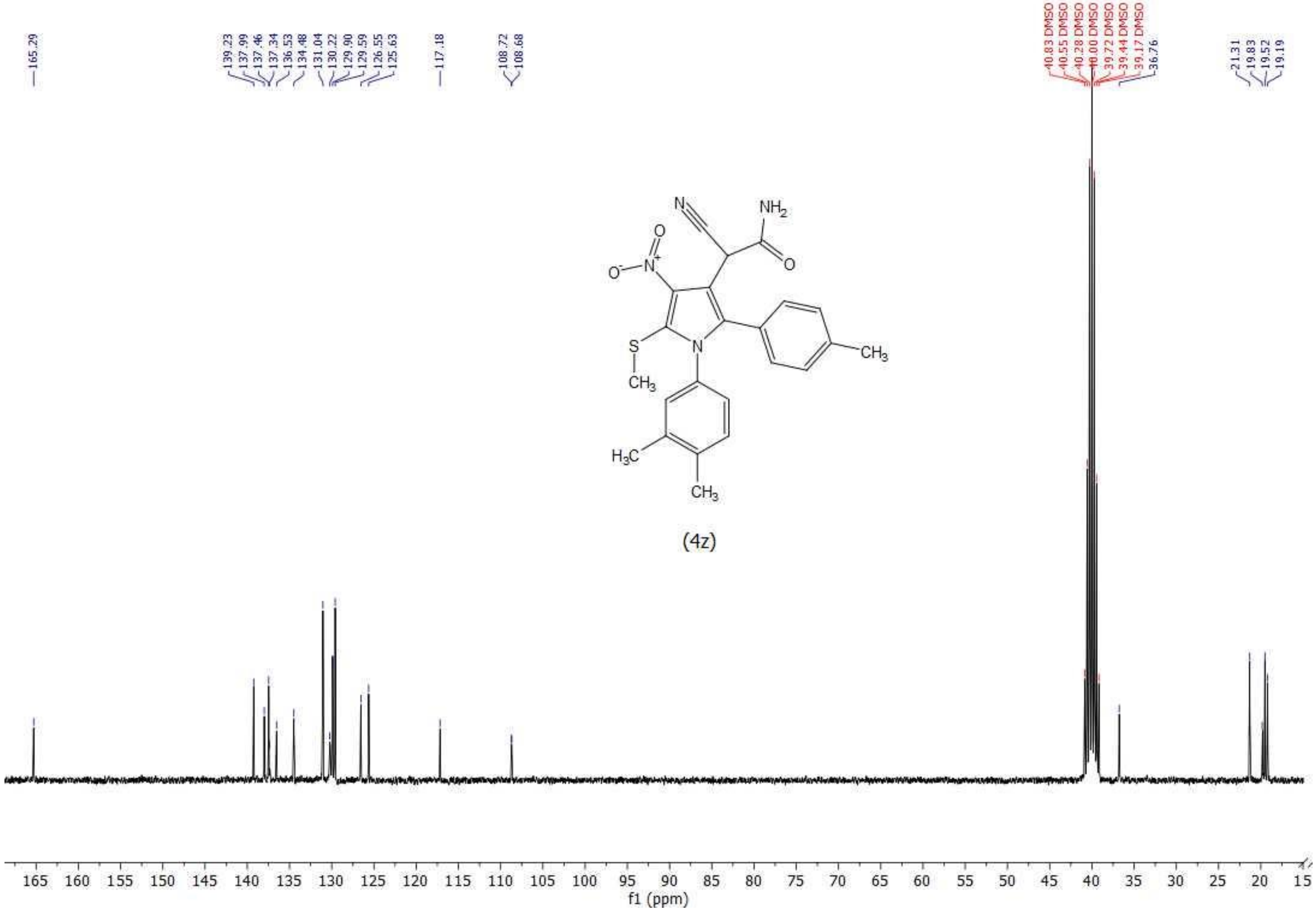
165.29
139.23
137.99
137.46
137.34
136.53
134.48
131.04
130.22
129.90
129.59
126.55
125.63
117.18
108.72
108.68
40.83 DMSO
40.55 DMSO
40.28 DMSO
40.00 DMSO
39.72 DMSO
39.44 DMSO
39.17 DMSO
36.76
21.31
19.83
19.52
19.19
N
NH2
O
O-
N+
S
CH3
N
CH3
H3C
CH3
(4z)
165 160 155 150 145 140 135 130 125 120 115 110 105 100 95 90 85 80 75 70 65 60 55 50 45 40 35 30 25 20 15
f1 (ppm)

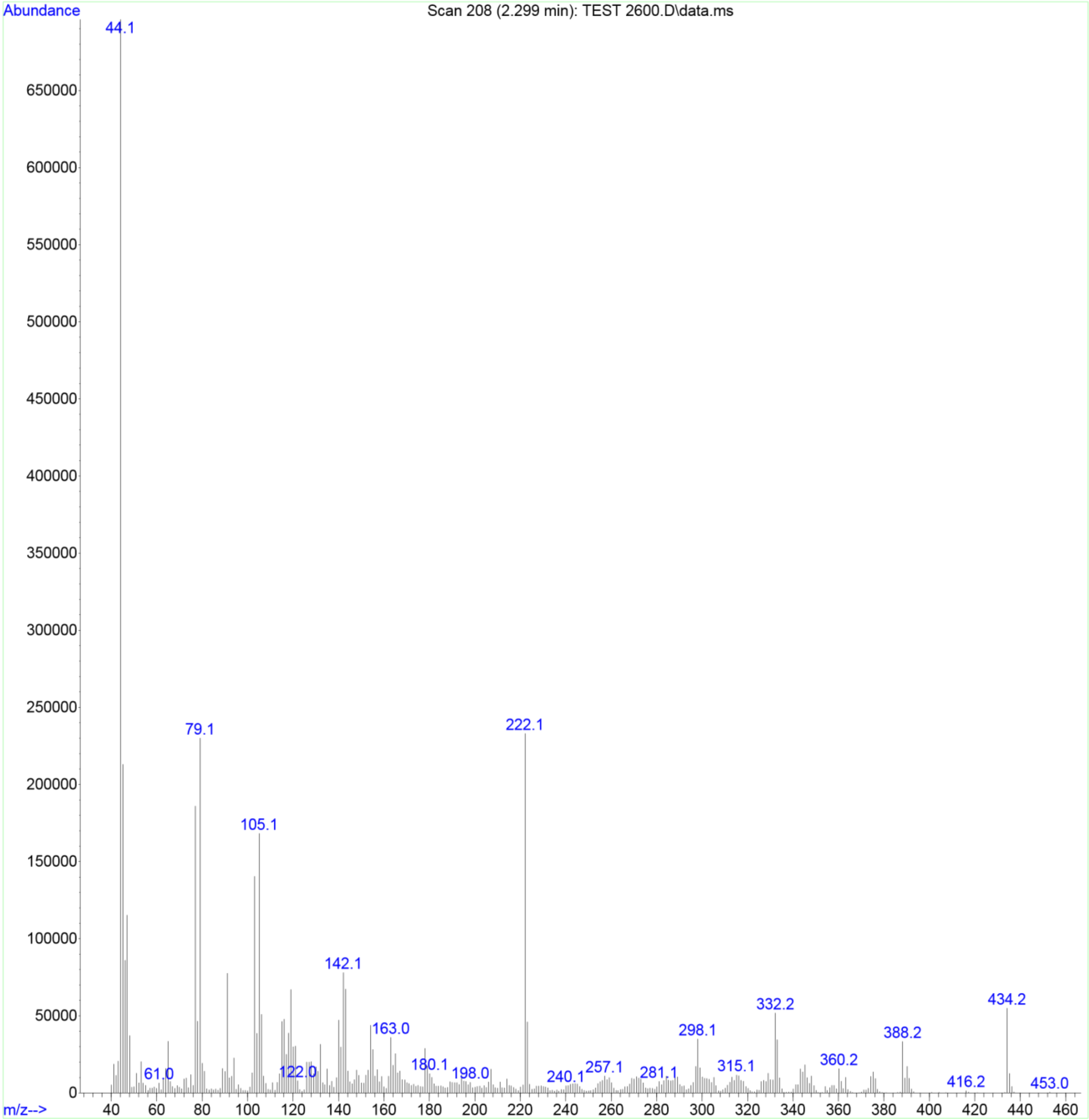

$C_{23}H_{22}N_4O_3S$

(434/5)

(4z)

4aa-H

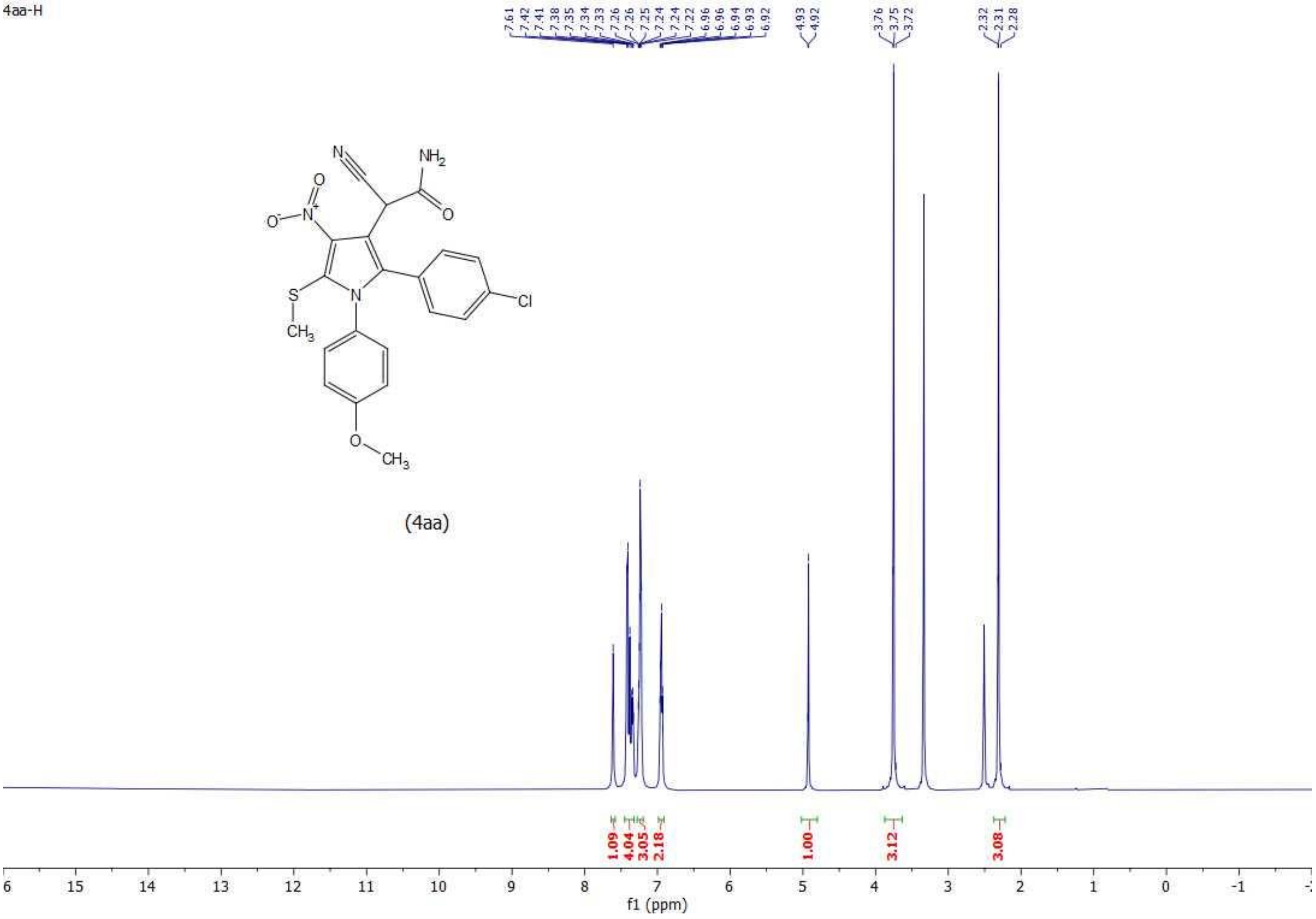
7.61 7.42 7.41 7.38 7.35 7.34 7.33 7.26 7.26 7.25 7.24 7.24 7.22 6.96 6.96 6.94 6.93 6.92
4.93 4.92
3.76 3.75 3.72
2.32 2.31 2.28
N
NH2
O
O-
N+
O
S
N
Cl
CH3
O
CH3
(4aa)
1.09
4.04
3.05
2.18
1.00
3.12
3.08
15 14 13 12 11 10 9 8 7 6 5 4 3 2 1 0 -1
f1 (ppm)

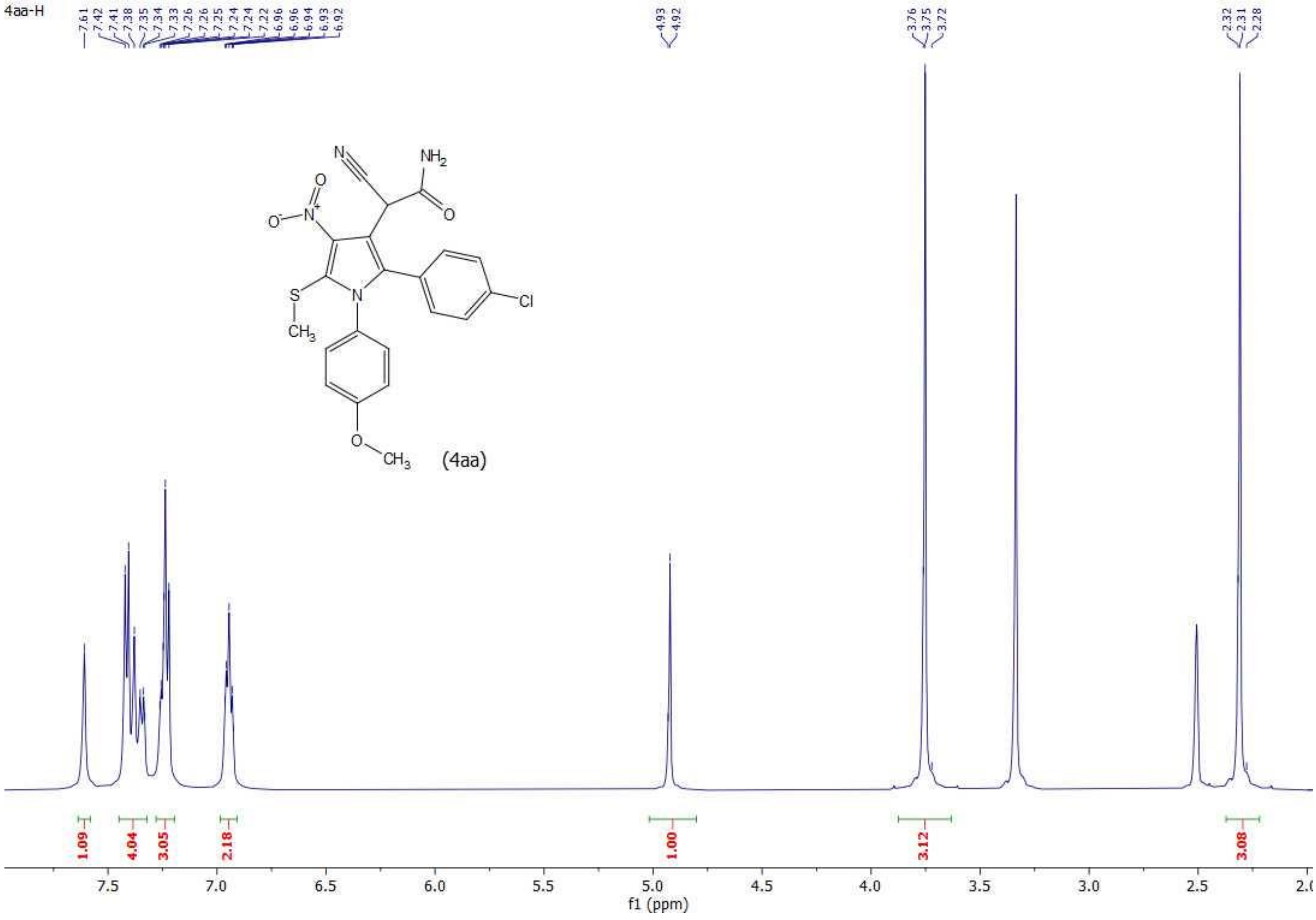
4aa-H
7.61
7.42
7.41
7.38
7.35
7.34
7.33
7.26
7.26
7.25
7.24
7.24
7.22
6.96
6.96
6.94
6.93
6.92
4.93
4.92
3.76
3.75
3.72
2.32
2.31
2.28
N
NH2
O
O-
N+
O
S
CH3
N
Cl
O
CH3
(4aa)
1.09
4.04
3.05
2.18
1.00
3.12
3.08
7.5
7.0
6.5
6.0
5.5
5.0
4.5
4.0
3.5
3.0
2.5
f1 (ppm)

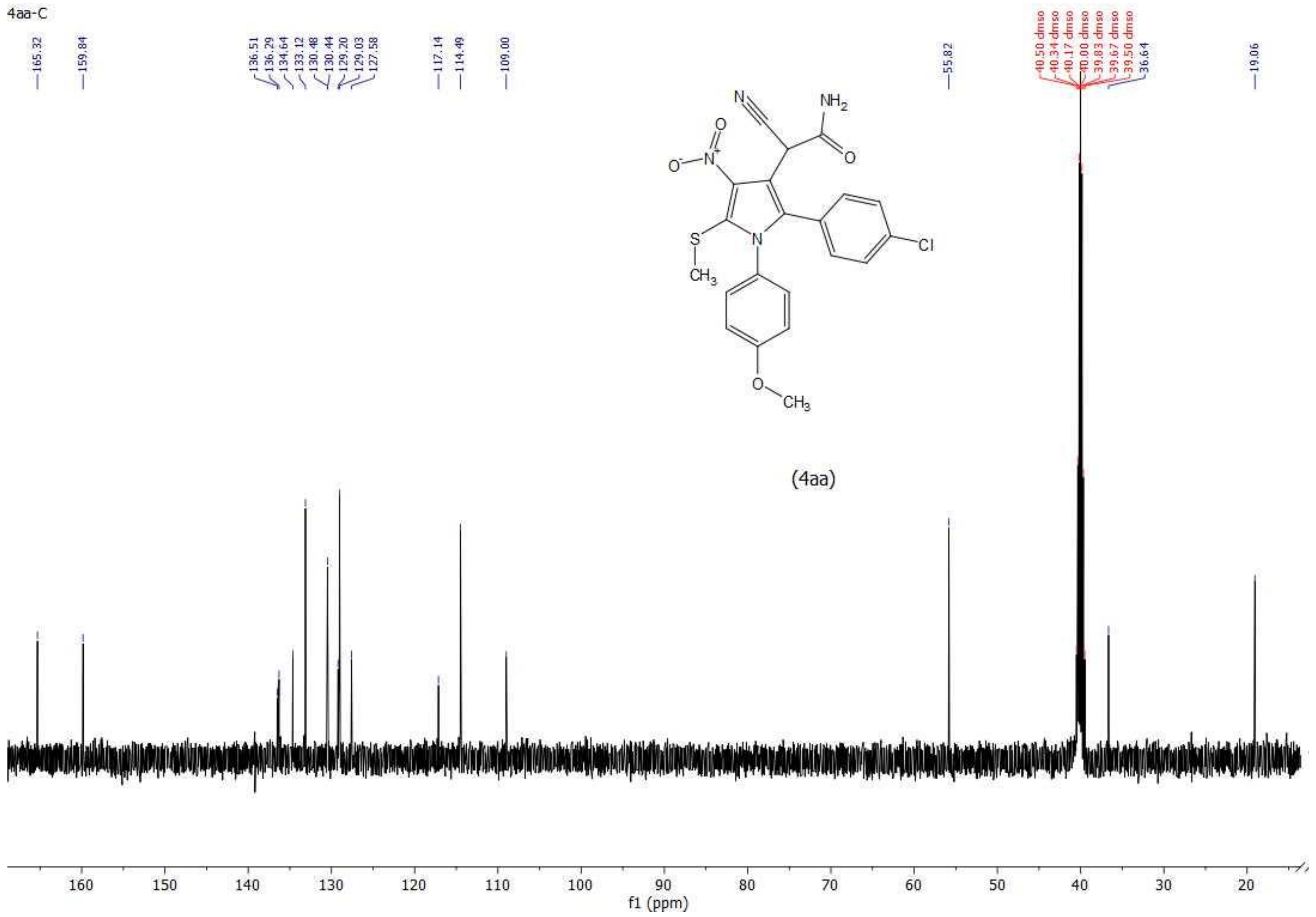

4aa-C
165.32
159.84
136.51
136.29
134.64
133.12
130.48
130.44
129.20
129.03
127.58
117.14
114.49
109.00
55.82
40.50 dmso
40.34 dmso
40.17 dmso
40.00 dmso
39.83 dmso
39.67 dmso
39.50 dmso
36.64
19.06
N
NH2
O
O-
N+
S
N
CH3
Cl
O
CH3
(4aa)
160
150
140
130
120
110
100
90
80
70
60
50
40
30
20
f1 (ppm)

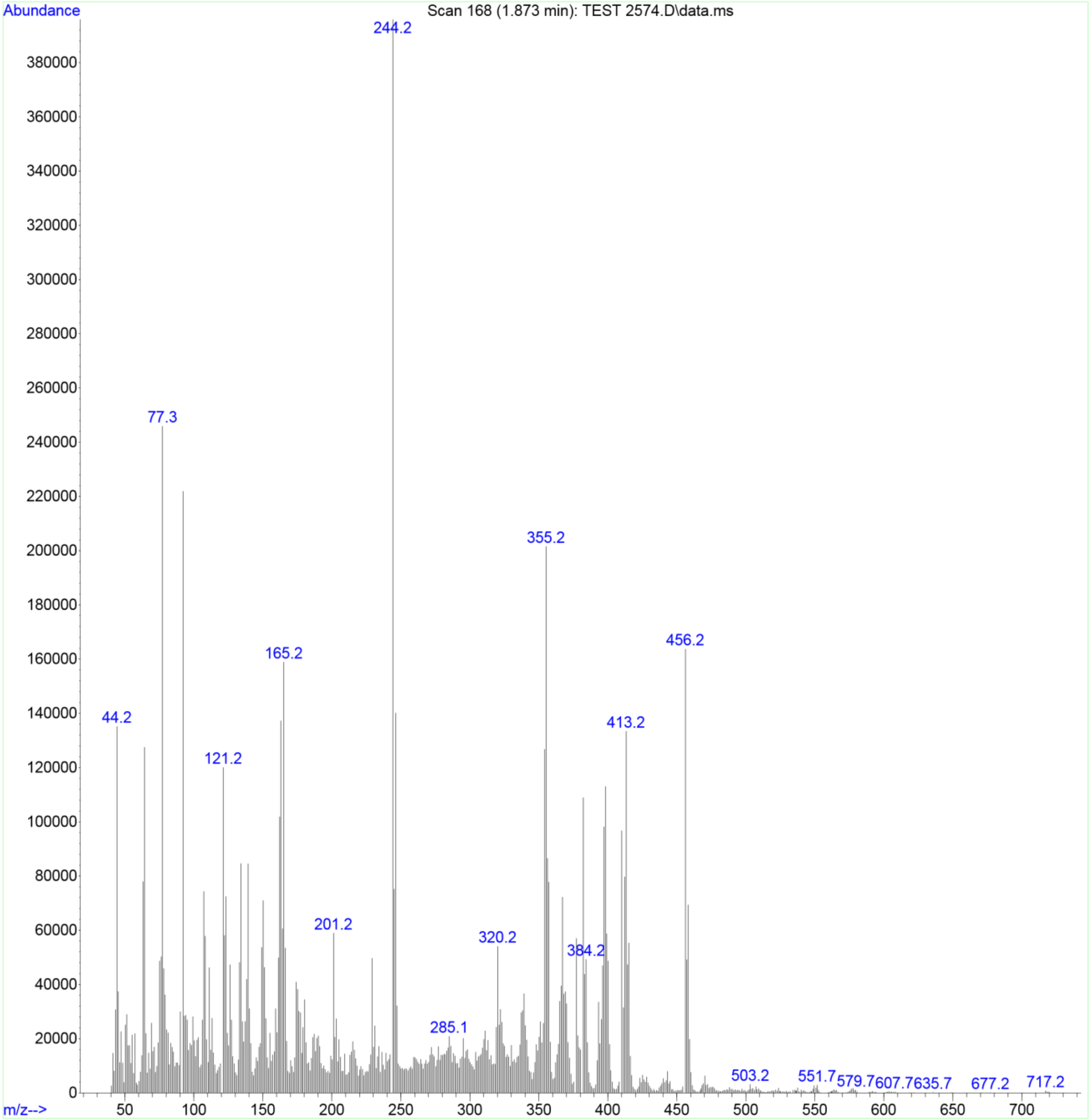

$C_{21}H_{17}ClN_4O_4S$

(456/9)

(4aa)

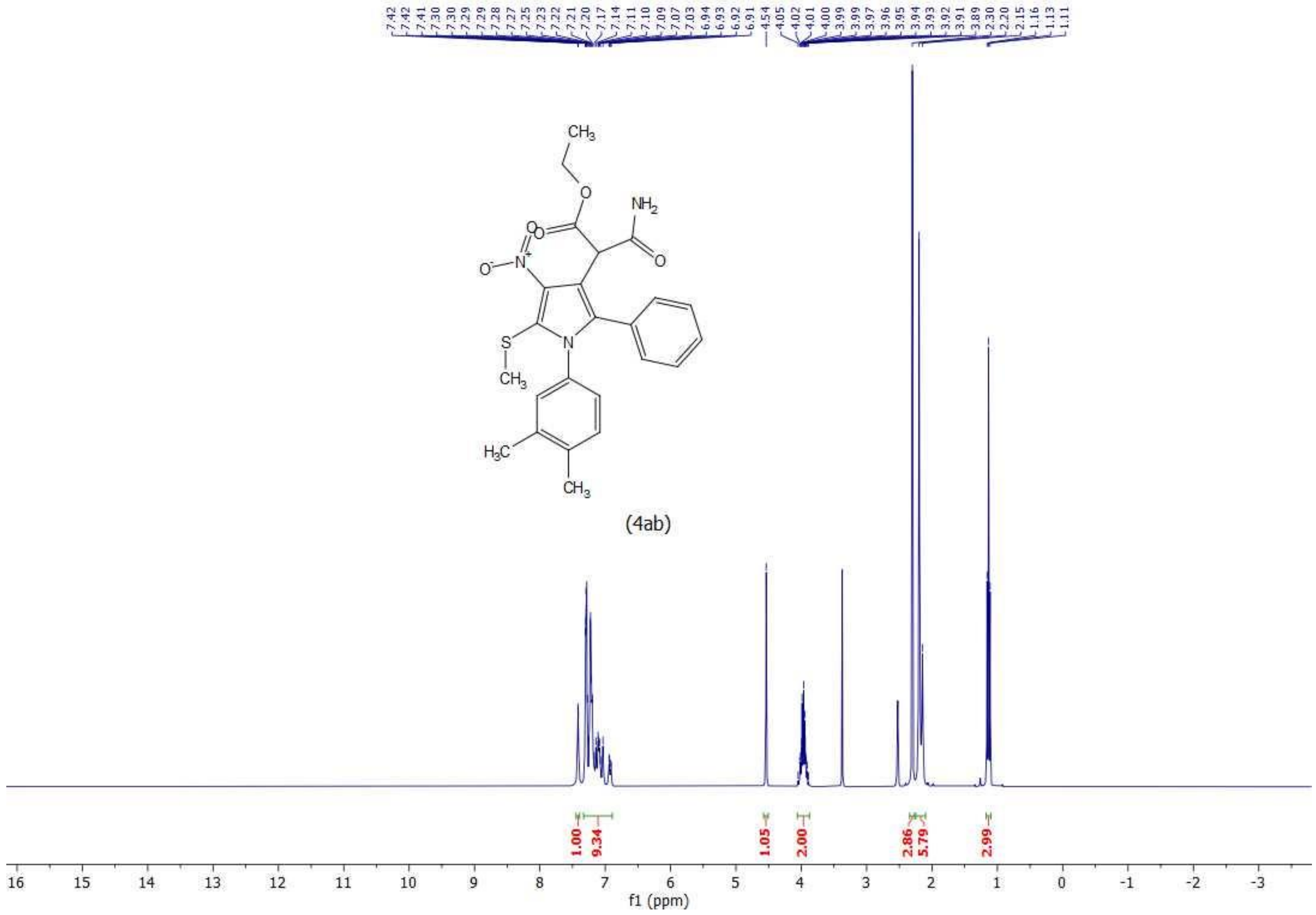
7.42
7.42
7.41
7.30
7.30
7.29
7.29
7.28
7.27
7.25
7.23
7.22
7.21
7.20
7.17
7.14
7.11
7.10
7.09
7.07
7.03
6.94
6.93
6.92
6.91
4.54
4.05
4.02
4.01
4.00
3.99
3.99
3.97
3.96
3.95
3.94
3.93
3.92
3.91
3.89
2.30
2.20
2.15
1.16
1.13
1.11
CH3
O
NH2
O
O
N+
O-
O
S
CH3
N
H3C
CH3
(4ab)
1.00
9.34
1.05
2.00
2.86
5.79
2.99
16
15
14
13
12
11
10
9
8
7
6
5
4
3
2
1
0
-1
-2
-3
f1 (ppm)

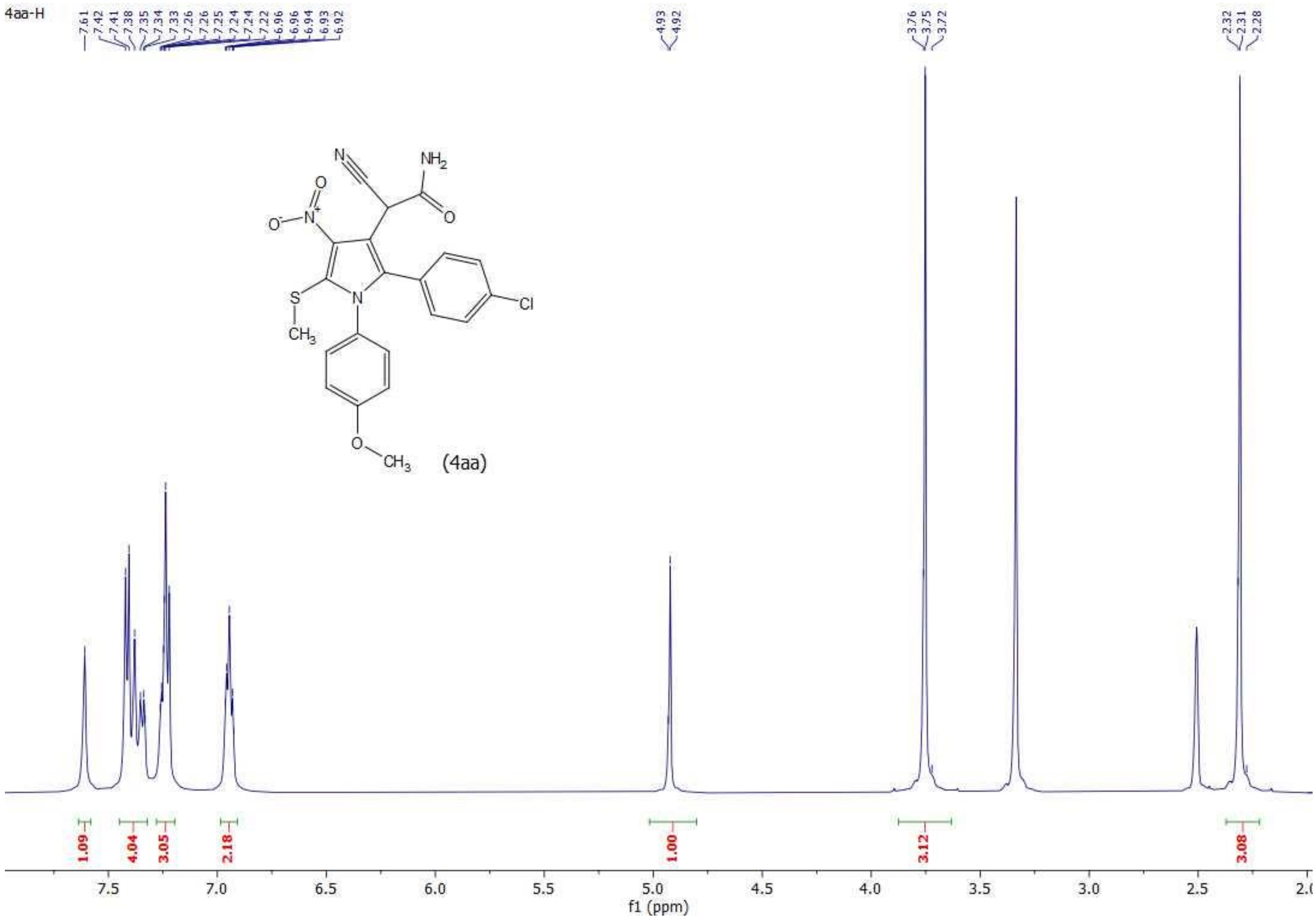

4aa-H
7.61
7.42
7.41
7.38
7.35
7.34
7.33
7.26
7.26
7.25
7.24
7.24
7.22
6.96
6.96
6.94
6.93
6.92
4.93
4.92
3.76
3.75
3.72
2.32
2.31
2.28
N
NH2
O
O-
N+
O
S
N
Cl
CH3
O
CH3
(4aa)
1.09
4.04
3.05
2.18
1.00
3.12
3.08
7.5
7.0
6.5
6.0
5.5
5.0
4.5
4.0
3.5
3.0
2.5
f1 (ppm)

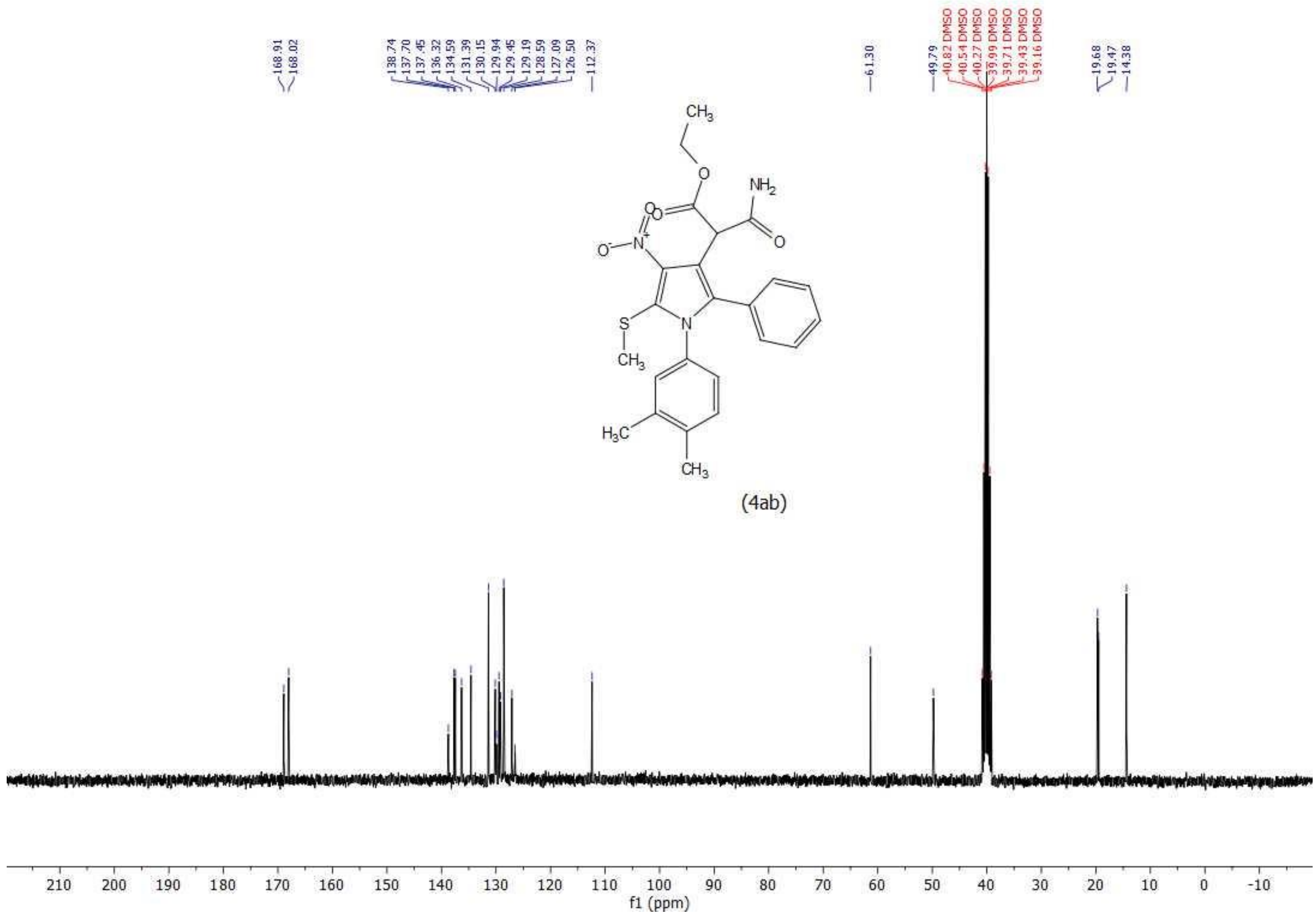
168.91
168.02
138.74
137.70
137.45
136.32
134.59
131.39
130.15
129.94
129.45
129.19
128.59
127.09
126.50
112.37
61.30
49.79
40.82 DMSO
40.54 DMSO
40.27 DMSO
39.99 DMSO
39.71 DMSO
39.43 DMSO
39.16 DMSO
19.68
19.47
14.38
CH3
O
NH2
O
O
O-
N+
O
S
N
CH3
H3C
CH3
210 200 190 180 170 160 150 140 130 120 110 100 90 80 70 60 50 40 30 20 10 0 -10
f1 (ppm)

(4ab)

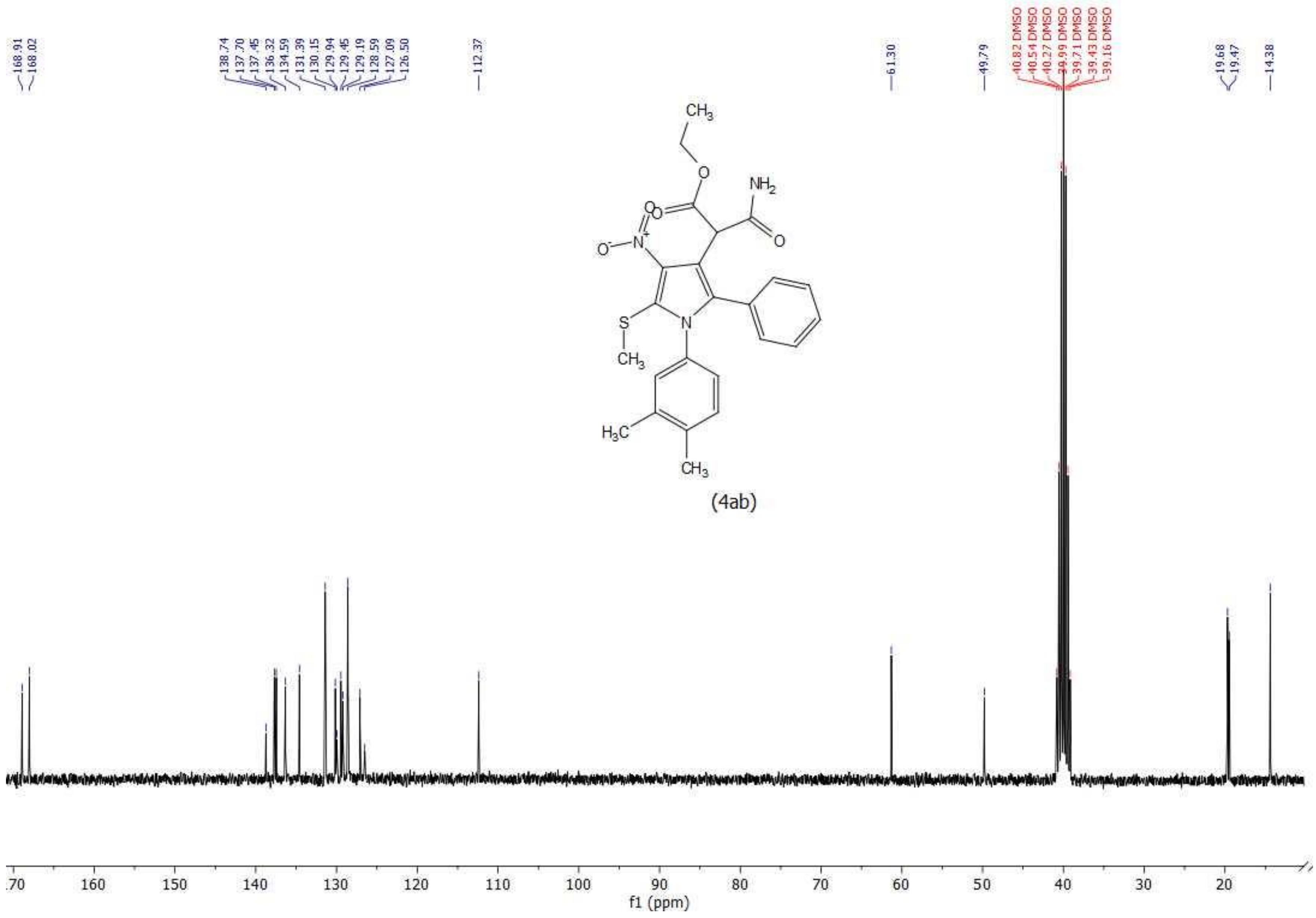
168.91
168.02
138.74
137.70
137.45
136.32
134.59
131.39
130.15
129.94
129.45
129.19
128.59
127.09
126.50
112.37
61.30
49.79
40.82 DMSO
40.54 DMSO
40.27 DMSO
39.99 DMSO
39.71 DMSO
39.43 DMSO
39.16 DMSO
19.68
19.47
14.38
(4ab)
f1 (ppm)

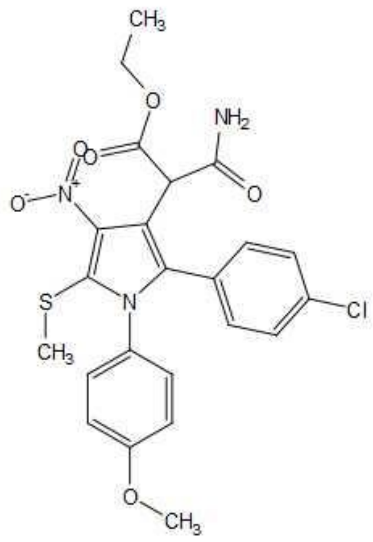

CH3
O
NH2
O
O
O-
N+
S
N
Cl
CH3
O
CH3

(4ac)

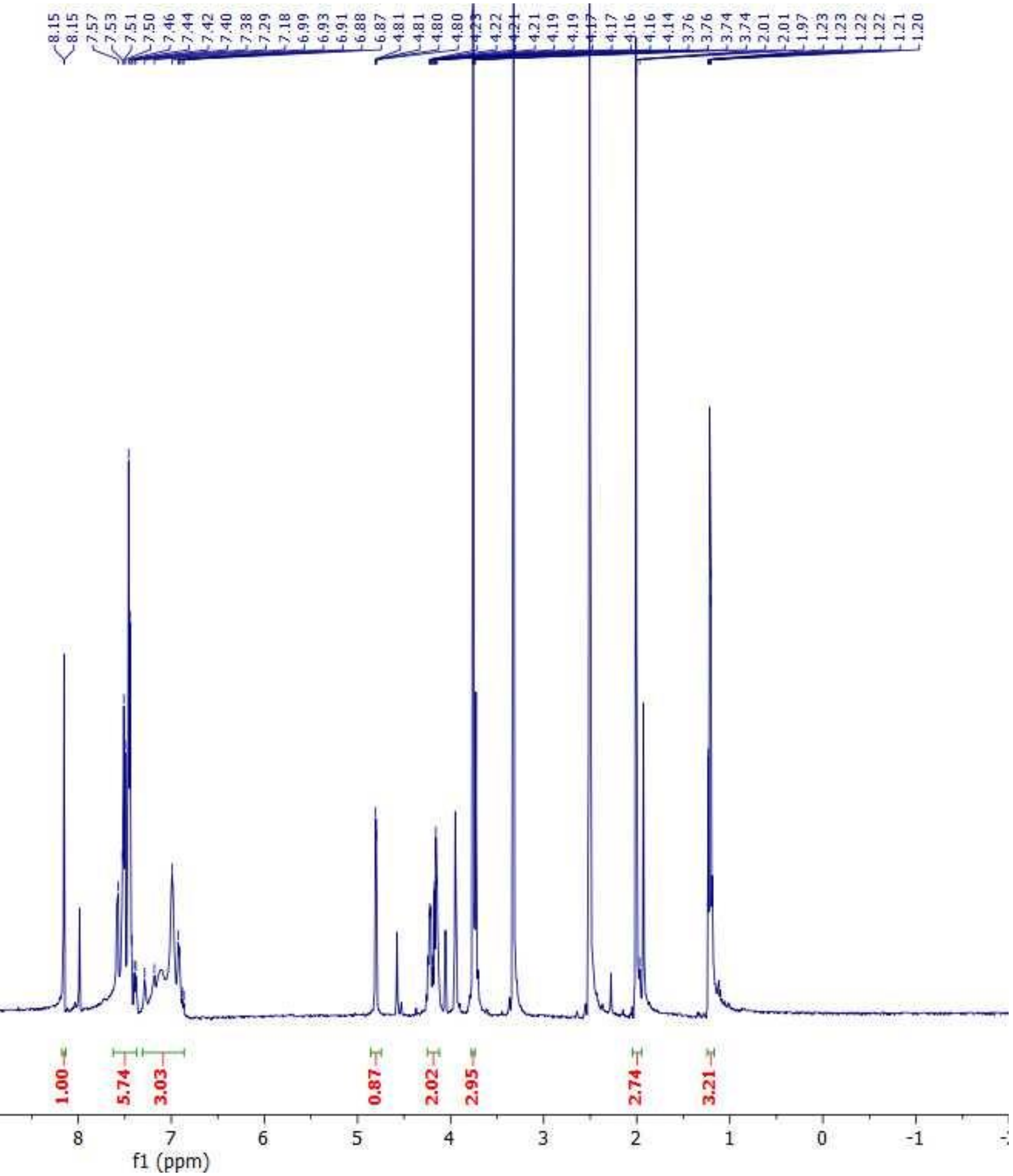

8.15
8.15
7.57
7.53
7.51
7.50
7.46
7.44
7.42
7.40
7.38
7.29
7.18
6.99
6.93
6.91
6.88
6.87
4.81
4.81
4.80
4.80
4.23
4.22
4.21
4.21
4.19
4.19
4.17
4.17
4.16
4.16
4.14
3.76
3.76
3.74
3.74
2.01
2.01
1.97
1.23
1.23
1.22
1.22
1.21
1.20
1.00
5.74
3.03
0.87
2.02
2.95
2.74
3.21
f1 (ppm)

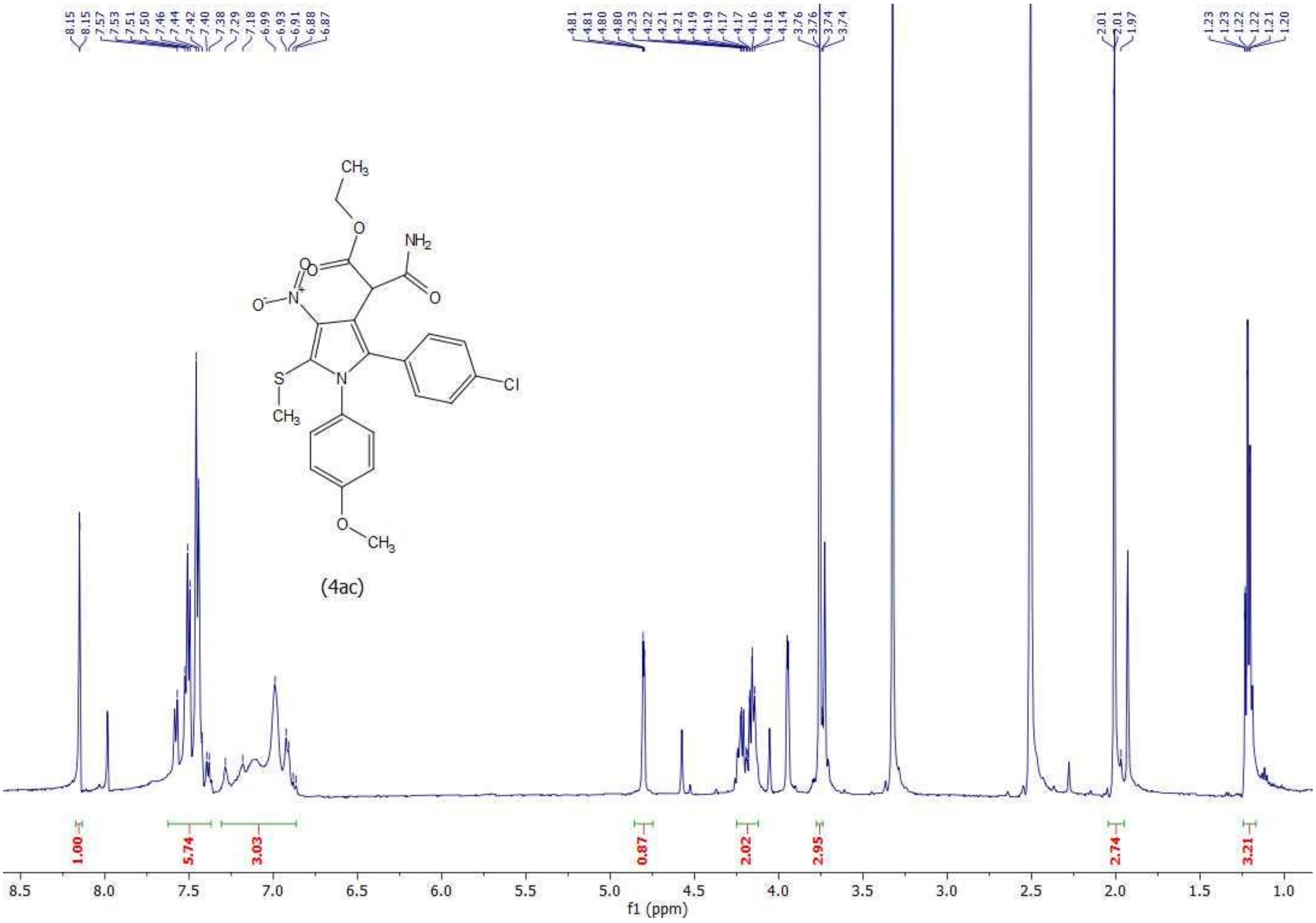
8.15 8.15 7.57 7.53 7.51 7.50 7.46 7.44 7.42 7.40 7.38 7.29 7.18 6.99 6.93 6.91 6.88 6.87
4.81 4.81 4.80 4.80 4.23 4.22 4.21 4.21 4.19 4.19 4.17 4.17 4.16 4.16 4.14 3.76 3.76 3.74 3.74
2.01 2.01 1.97
1.23 1.23 1.22 1.22 1.21 1.20
CH3
O
NH2
O
O
O-
N+
O
S
N
Cl
CH3
O
CH3
(4ac)
1.00
5.74
3.03
0.87
2.02
2.95
2.74
3.21
8.5 8.0 7.5 7.0 6.5 6.0 5.5 5.0 4.5 4.0 3.5 3.0 2.5 2.0 1.5 1.0
f1 (ppm)

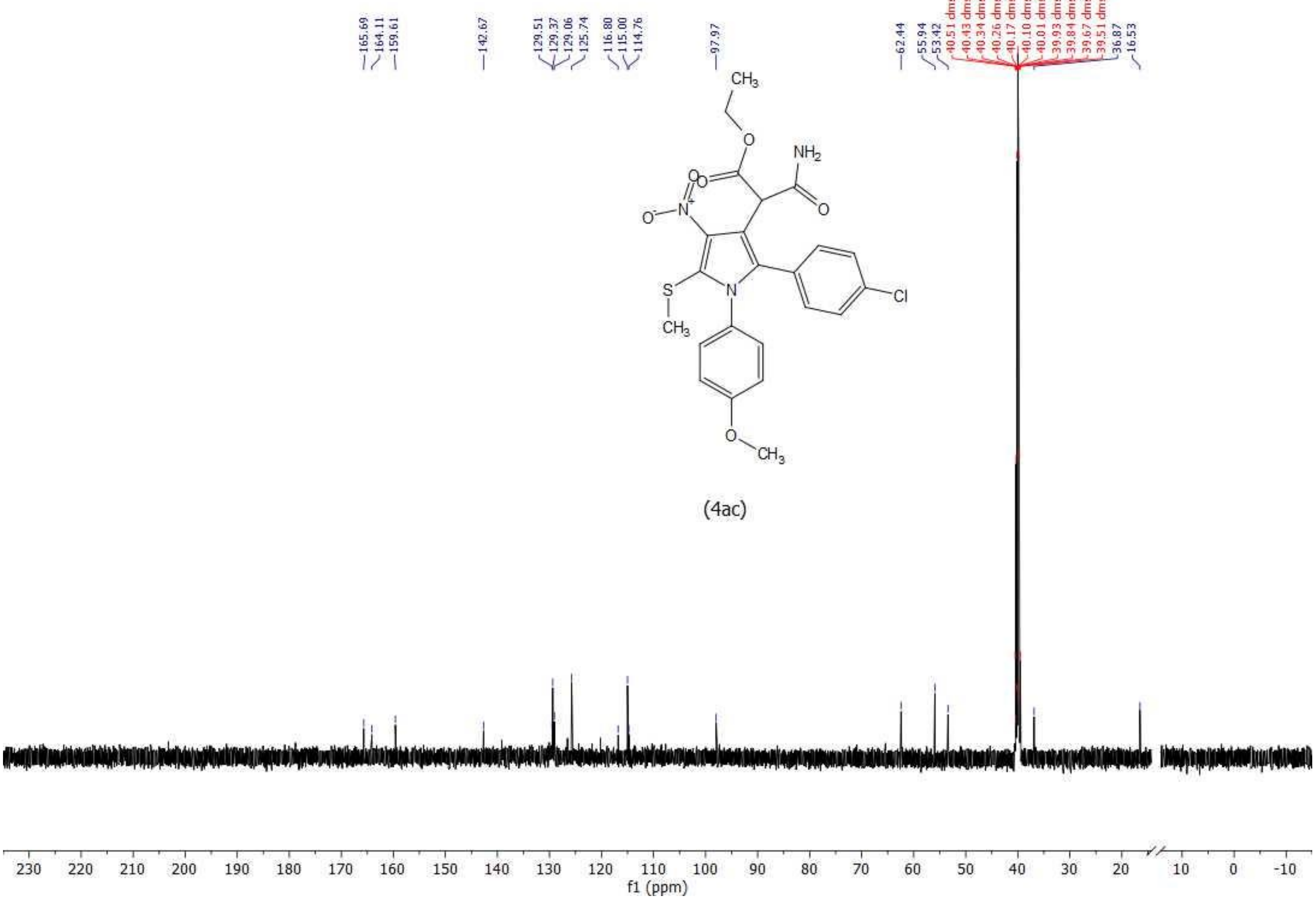
165.69
164.11
159.61
142.67
129.51
129.37
129.06
125.74
116.80
115.00
114.76
97.97
62.44
55.94
53.42
40.51 dmso
40.43 dmso
40.34 dmso
40.26 dmso
40.17 dmso
40.10 dmso
40.01 dmso
39.93 dmso
39.84 dmso
39.67 dmso
39.51 dmso
36.87
16.53
CH3
O
NH2
O
N+
O-
O
S
CH3
N
Cl
O
CH3
(4ac)
230 220 210 200 190 180 170 160 150 140 130 120 110 100 90 80 70 60 50 40 30 20 10 0 -10
f1 (ppm)

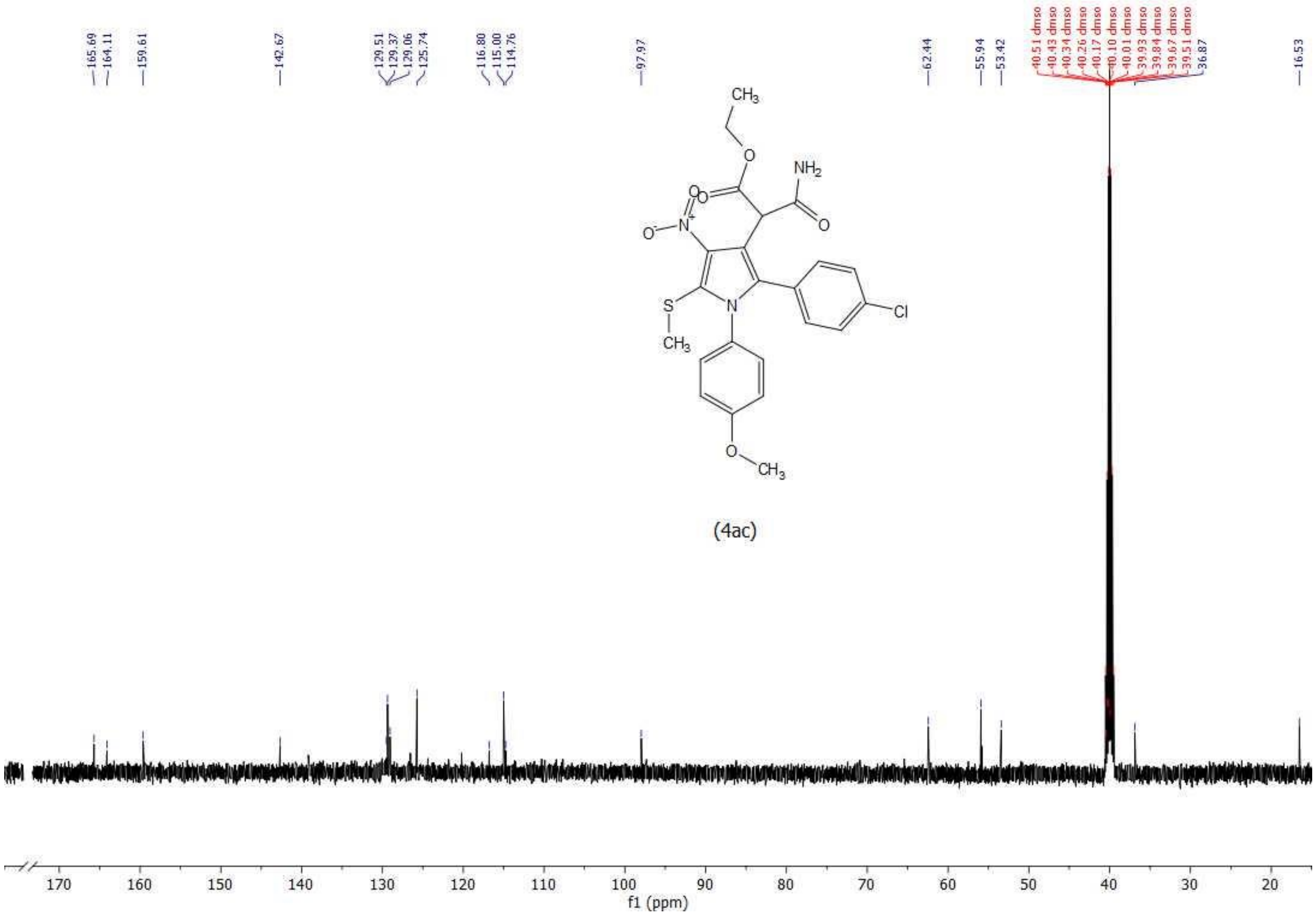
165.69
164.11
159.61
142.67
129.51
129.37
129.06
125.74
116.80
115.00
114.76
97.97
62.44
55.94
53.42
40.51 dmso
40.43 dmso
40.34 dmso
40.26 dmso
40.17 dmso
40.10 dmso
40.01 dmso
39.93 dmso
39.84 dmso
39.67 dmso
39.51 dmso
36.87
16.53
CH3
O
NH2
O
O
O-
N+
S
CH3
N
Cl
O
CH3
170
160
150
140
130
120
110
100
90
80
70
60
50
40
30
20
f1 (ppm)

(4ac)

4ad-H

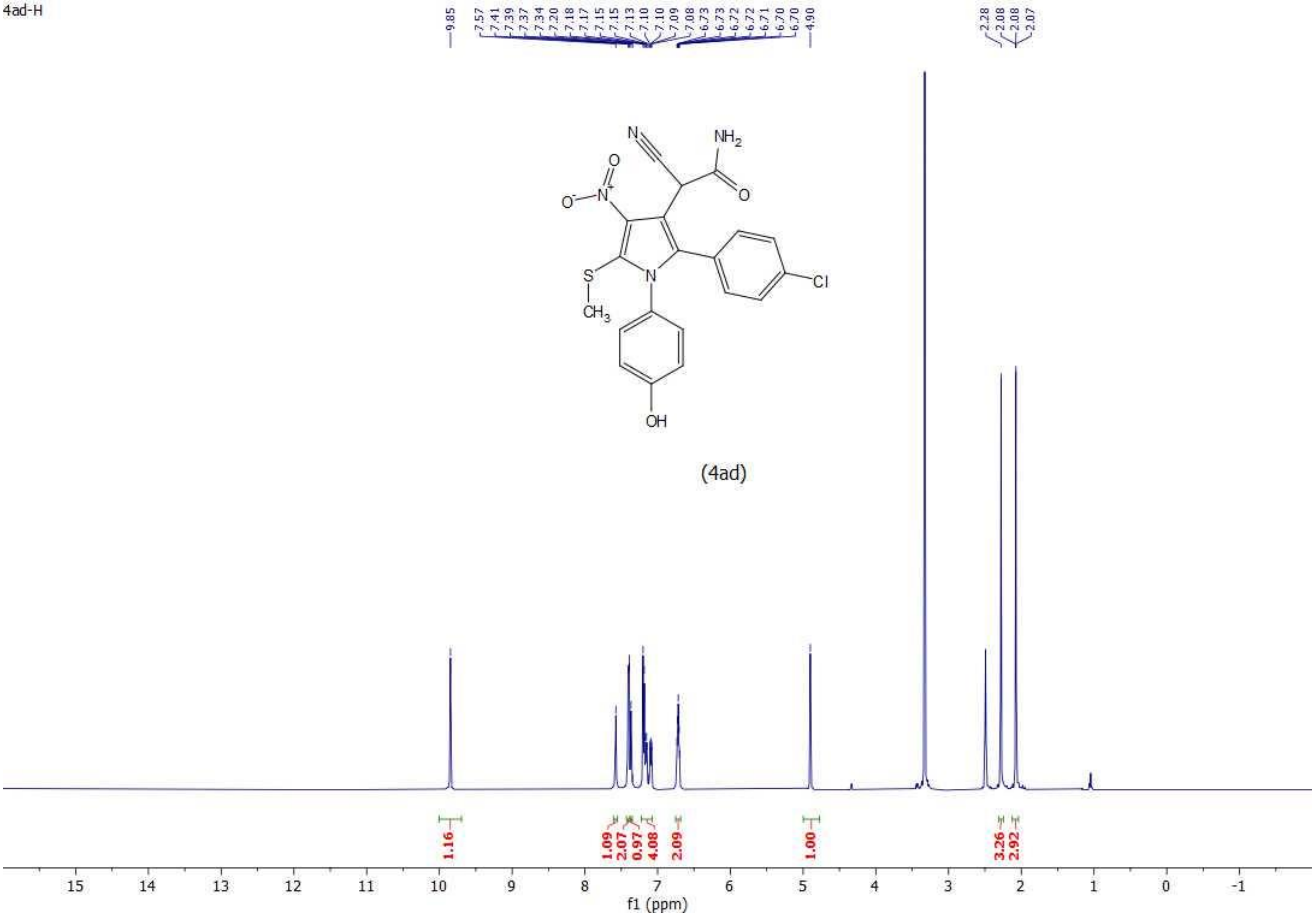

9.85
7.57
7.41
7.39
7.37
7.34
7.20
7.18
7.17
7.15
7.15
7.13
7.10
7.10
7.09
7.08
6.73
6.73
6.72
6.72
6.71
6.70
6.70
4.90
2.28
2.08
2.08
2.07
N
NH2
O
O-
N+
S
N
Cl
CH3
OH
1.16
1.09
2.07
0.97
4.08
2.09
1.00
3.26
2.92
15
14
13
12
11
10
9
8
7
6
5
4
3
2
1
0
-1
f1 (ppm)

(4ad)

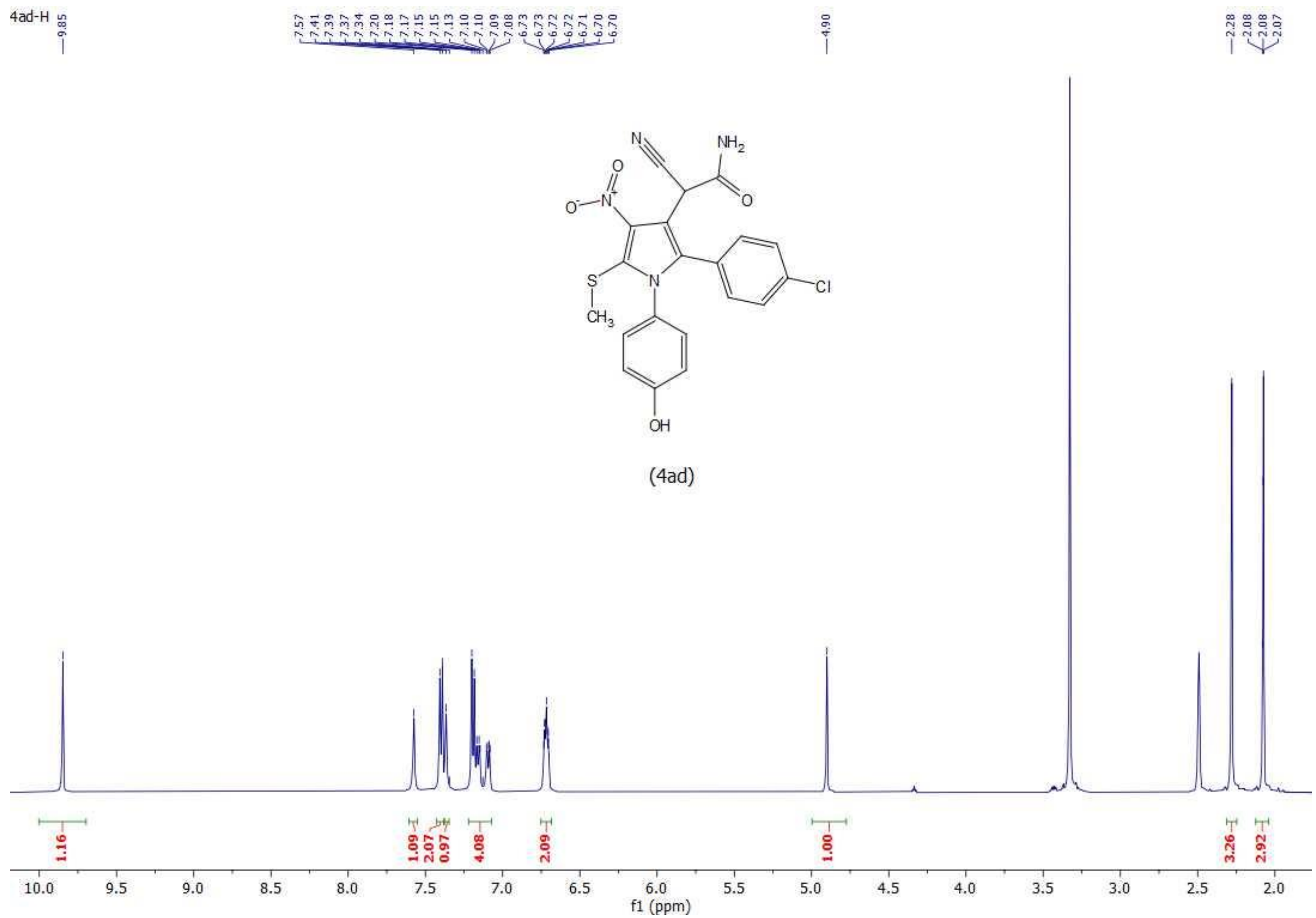
4ad-H
9.85
7.57 7.41 7.39 7.37 7.34 7.20 7.18 7.17 7.15 7.15 7.13 7.10 7.10 7.09 7.08
6.73 6.73 6.72 6.72 6.71 6.70 6.70
4.90
2.28 2.08 2.08 2.07
N
NH2
O
O-
N+
S
CH3
N
Cl
OH
(4ad)
1.16
1.09
2.07
0.97
4.08
2.09
1.00
3.26
2.92
10.0 9.5 9.0 8.5 8.0 7.5 7.0 6.5 6.0 5.5 5.0 4.5 4.0 3.5 3.0 2.5 2.0
f1 (ppm)

4ad-C

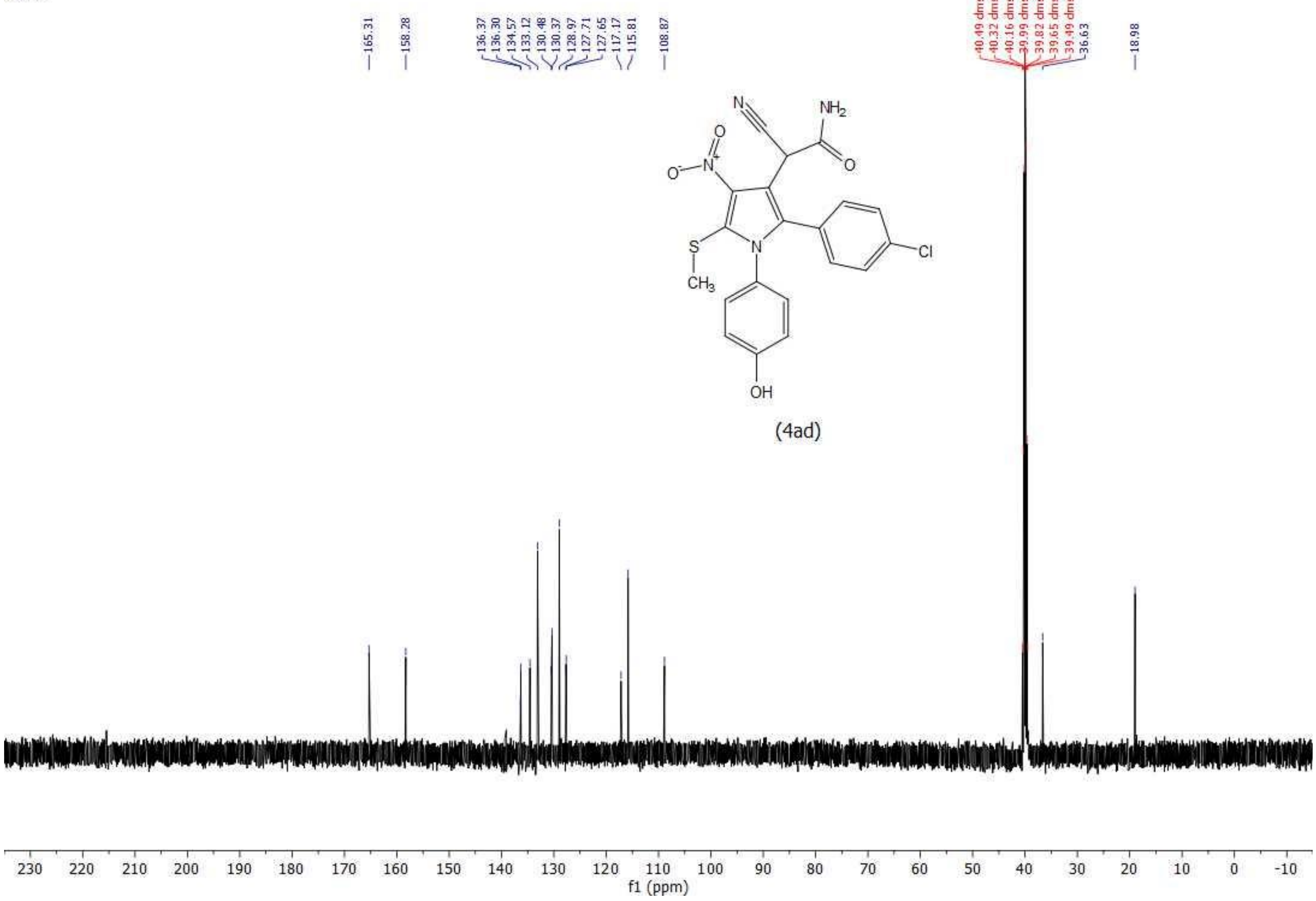
165.31
158.28
136.37
136.30
134.57
133.12
130.48
130.37
128.97
127.71
127.65
117.17
115.81
108.87
40.49 dmso
40.32 dmso
40.16 dmso
39.99 dmso
39.82 dmso
39.65 dmso
39.49 dmso
36.63
18.98
N
NH2
O
O
O-
N+
S
CH3
N
Cl
OH
(4ad)
230 220 210 200 190 180 170 160 150 140 130 120 110 100 90 80 70 60 50 40 30 20 10 0 -10
f1 (ppm)

4ad-C

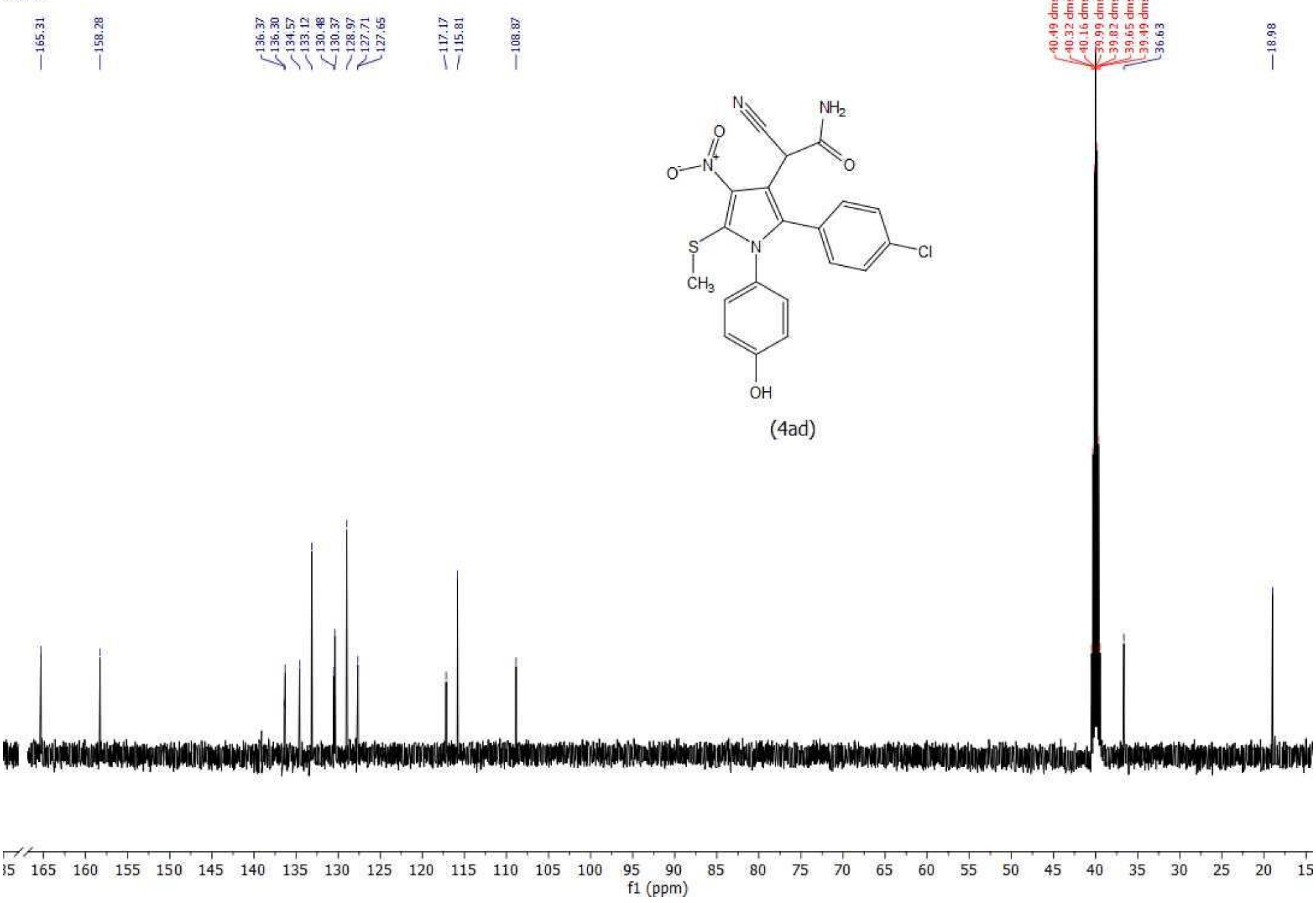
165.31
158.28
136.37
136.30
134.57
133.12
130.48
130.37
128.97
127.71
127.65
117.17
115.81
108.87
40.49 dmso
40.32 dmso
40.16 dmso
39.99 dmso
39.82 dmso
39.65 dmso
39.49 dmso
36.63
18.98
NH2
N
O
O-
N+
S
CH3
Cl
OH
(4ad)
f1 (ppm)

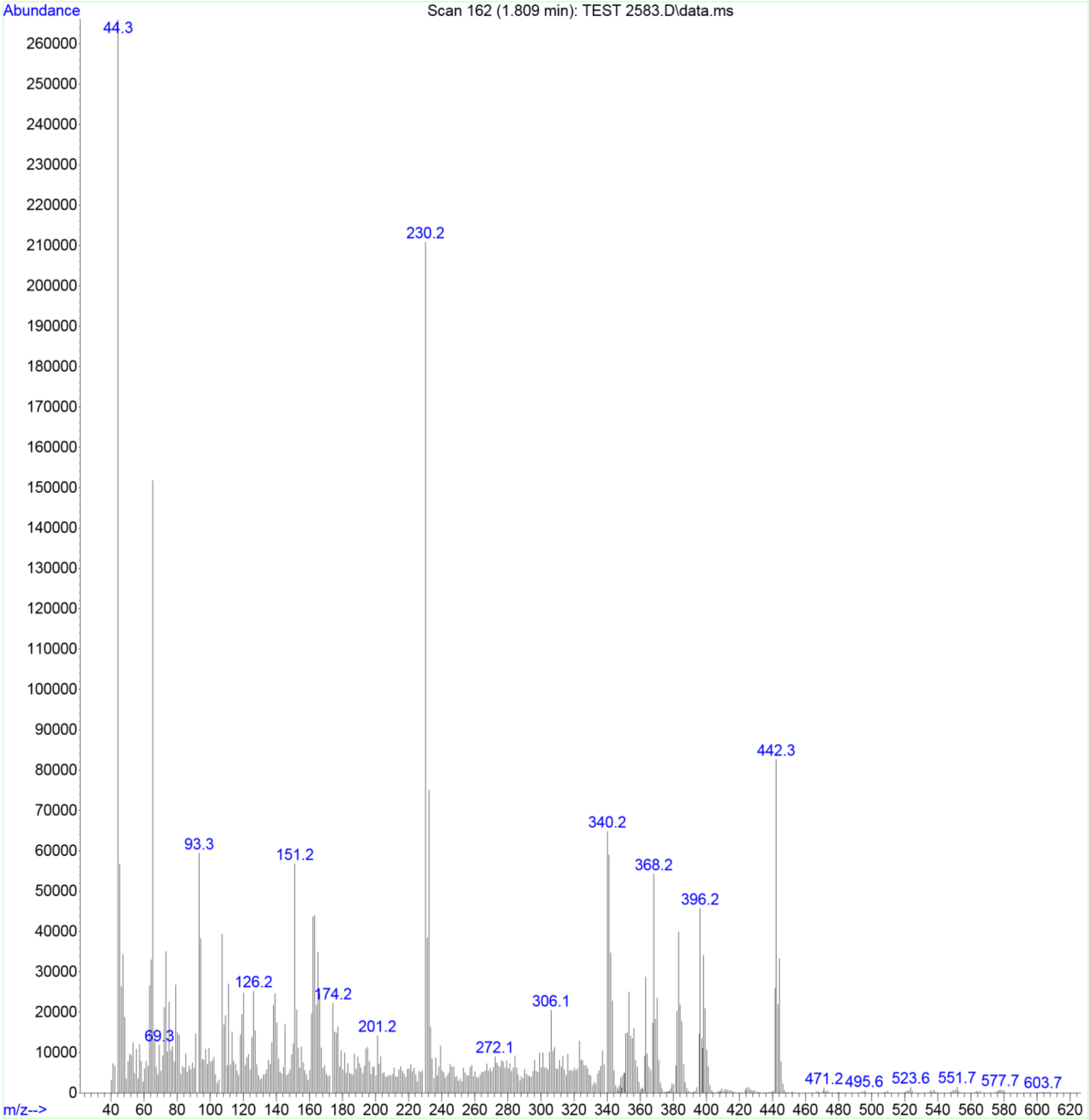

$C_{20}H_{15}ClN_4O_4S$

(442/88)

(4ad)

4ae-H

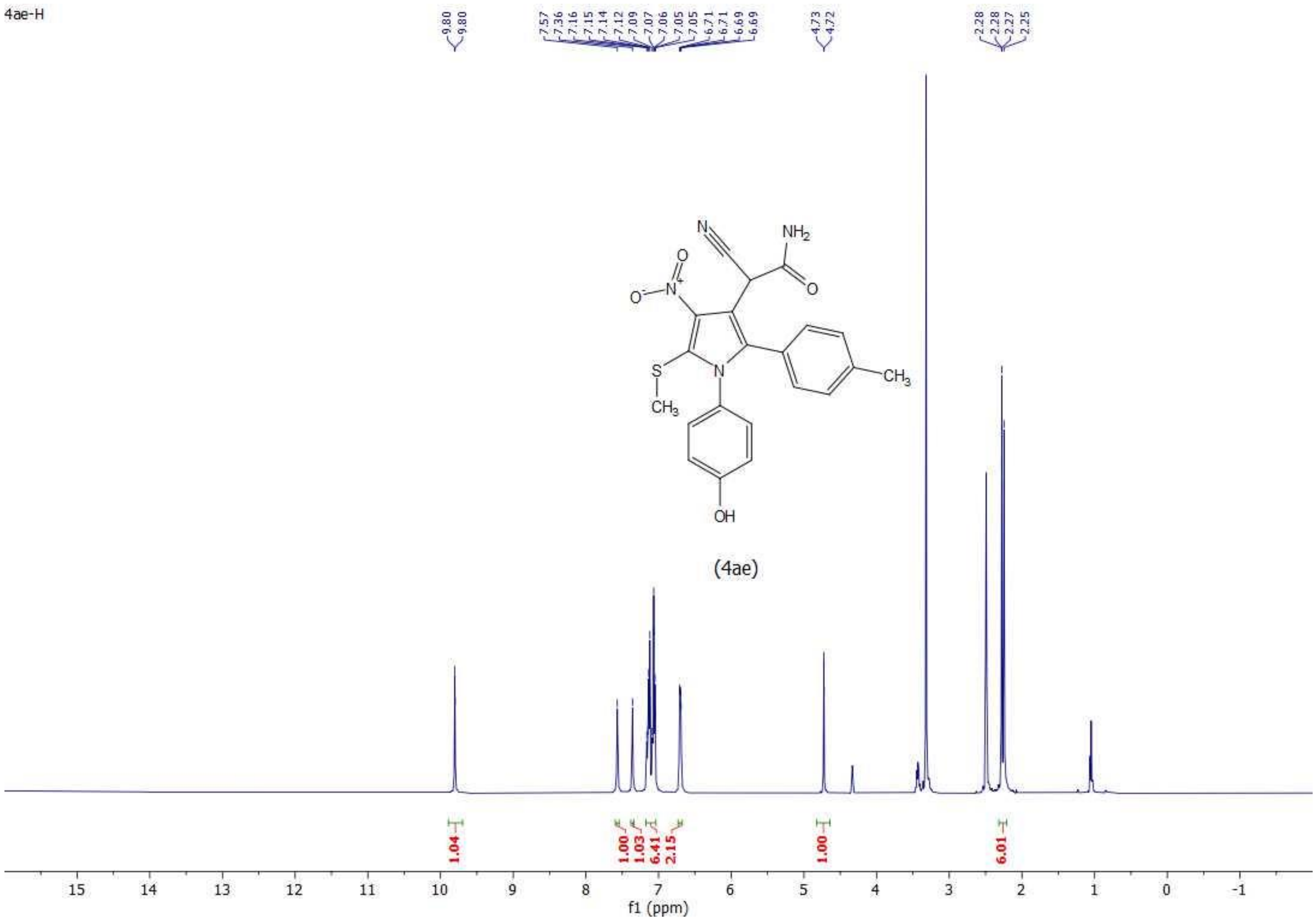

9.80
9.80
7.57
7.36
7.16
7.15
7.14
7.12
7.09
7.07
7.06
7.05
7.05
6.71
6.71
6.69
6.69
4.73
4.72
2.28
2.28
2.27
2.25
NH2
N
O
O-
N+
O
S
CH3
N
CH3
OH
(4ae)
1.04
1.00
1.03
6.41
2.15
1.00
6.01
15
14
13
12
11
10
9
8
7
6
5
4
3
2
1
0
-1
f1 (ppm)

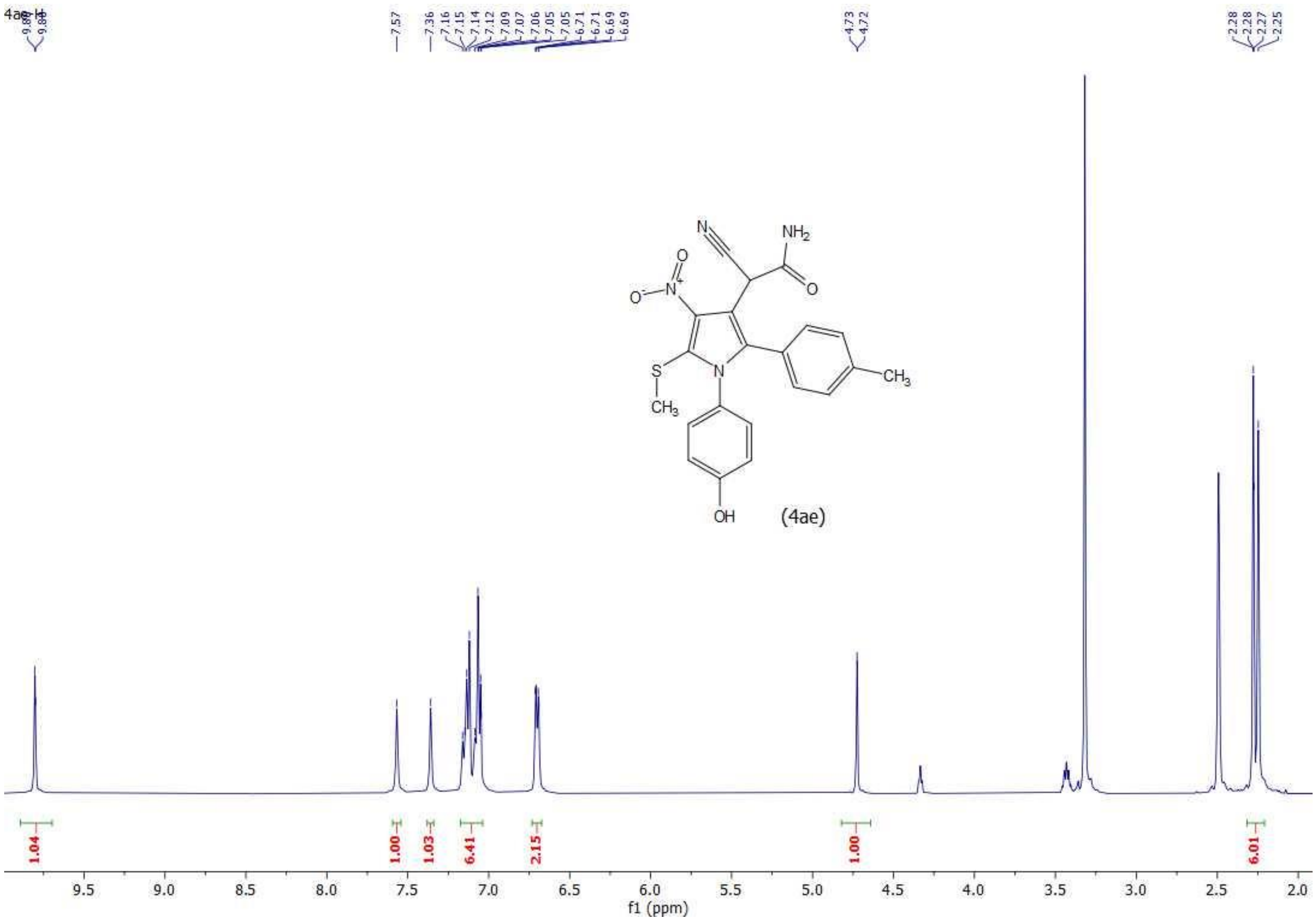
4ae H
9.80
9.80
7.57
7.36
7.16
7.15
7.14
7.12
7.09
7.07
7.06
7.05
7.05
6.71
6.71
6.69
6.69
4.73
4.72
2.28
2.28
2.27
2.25
N
NH2
O
O
N
O
S
CH3
N
CH3
OH
(4ae)
1.04
1.00
1.03
6.41
2.15
1.00
6.01
9.5
9.0
8.5
8.0
7.5
7.0
6.5
6.0
5.5
5.0
4.5
4.0
3.5
3.0
2.5
2.0
f1 (ppm)

4ae-C

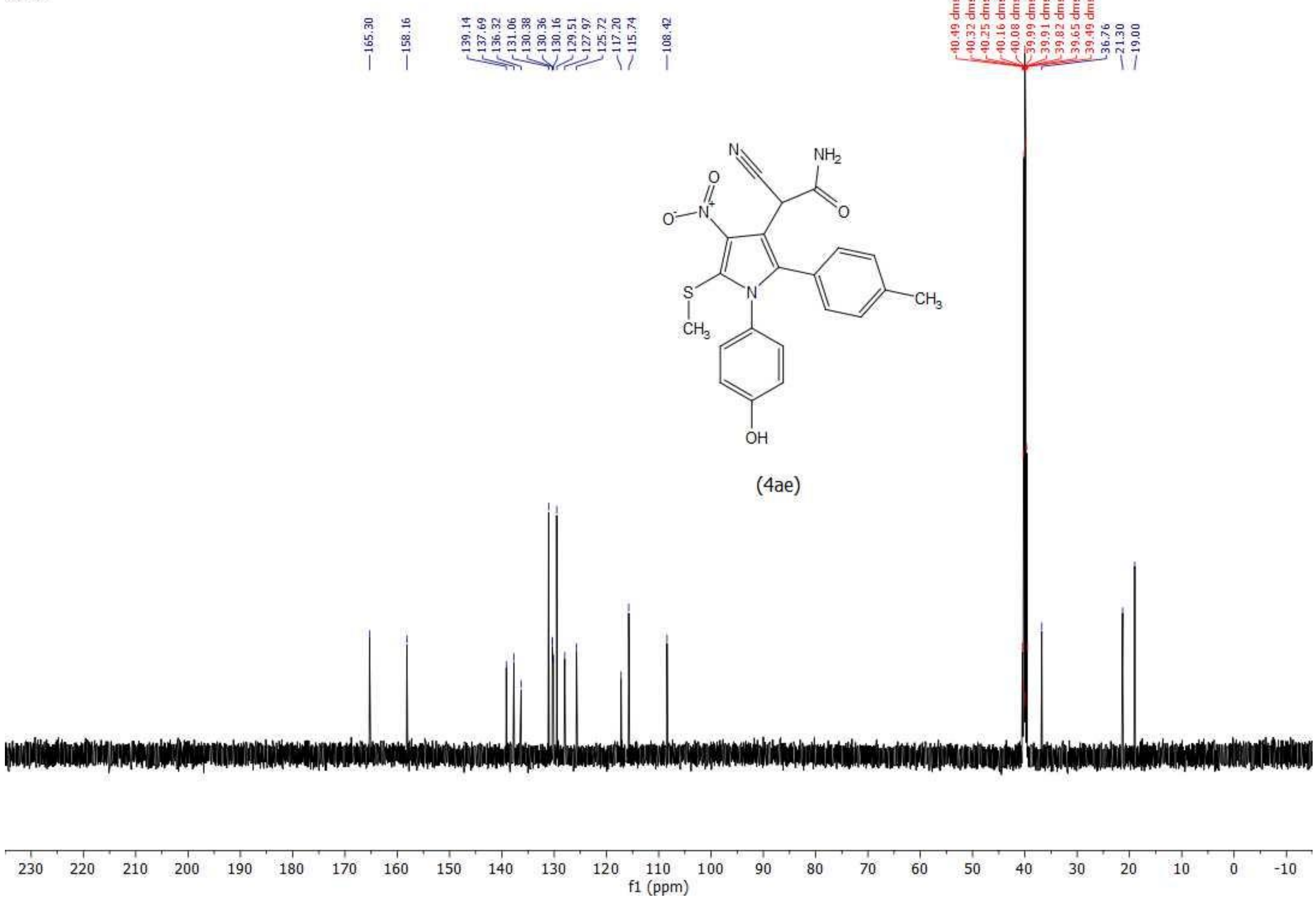

165.30
158.16
139.14
137.69
136.32
131.06
130.38
130.36
130.16
129.51
127.97
125.72
117.20
115.74
108.42
40.49 dmso
40.32 dmso
40.25 dmso
40.16 dmso
40.08 dmso
39.99 dmso
39.91 dmso
39.82 dmso
39.65 dmso
39.49 dmso
36.76
21.30
19.00
N
NH2
O
O-
N+
S
N
CH3
CH3
OH
230 220 210 200 190 180 170 160 150 140 130 120 110 100 90 80 70 60 50 40 30 20 10 0 -10
f1 (ppm)

(4ae)

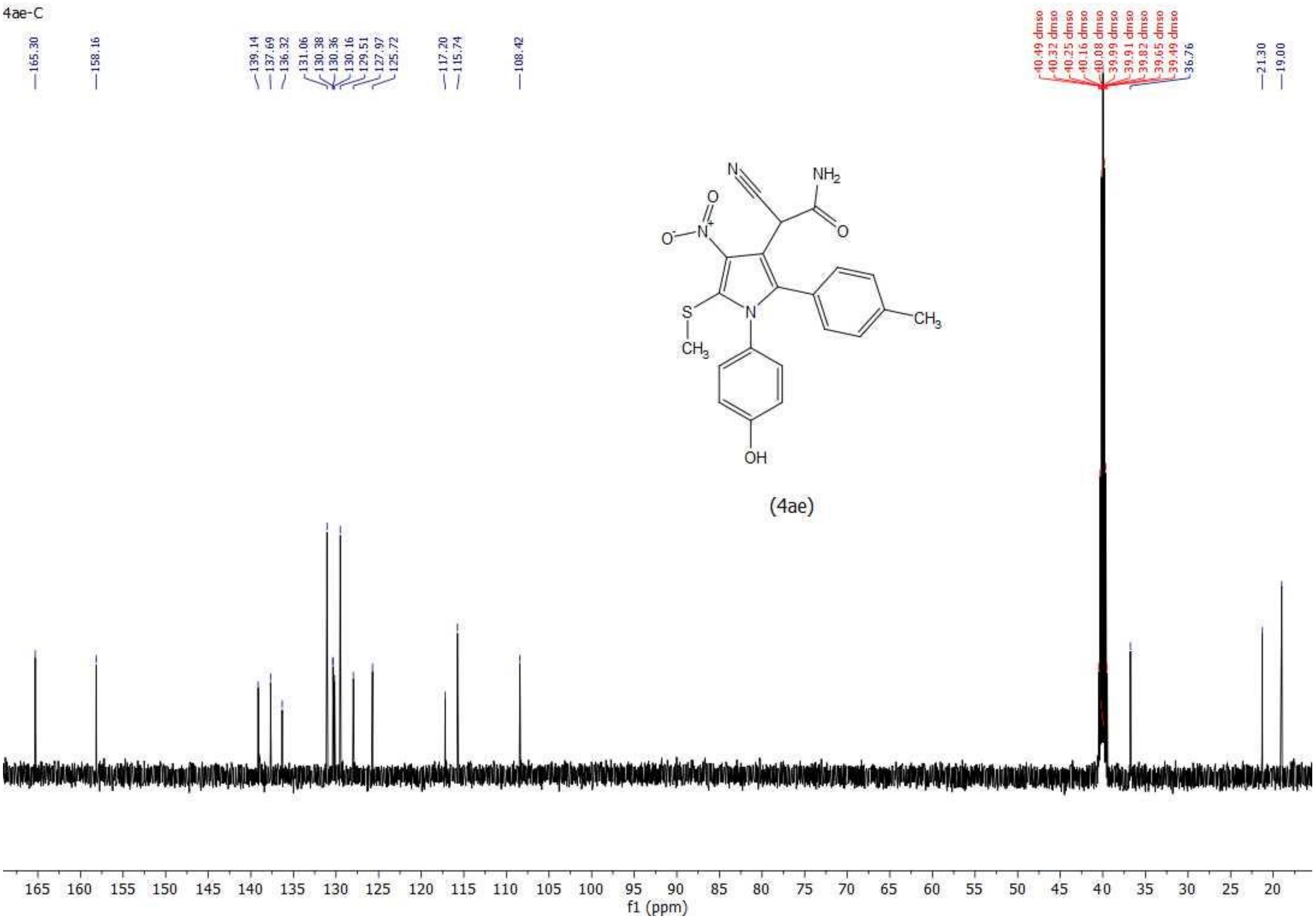
4ae-C
165.30
158.16
139.14
137.69
136.32
131.06
130.38
130.36
130.16
129.51
127.97
125.72
117.20
115.74
108.42
40.49 dmso
40.32 dmso
40.25 dmso
40.16 dmso
40.08 dmso
39.99 dmso
39.91 dmso
39.82 dmso
39.65 dmso
39.49 dmso
36.76
21.30
19.00
N
NH2
O
O-
N+
S
N
CH3
CH3
OH
(4ae)
165 160 155 150 145 140 135 130 125 120 115 110 105 100 95 90 85 80 75 70 65 60 55 50 45 40 35 30 25 20
f1 (ppm)

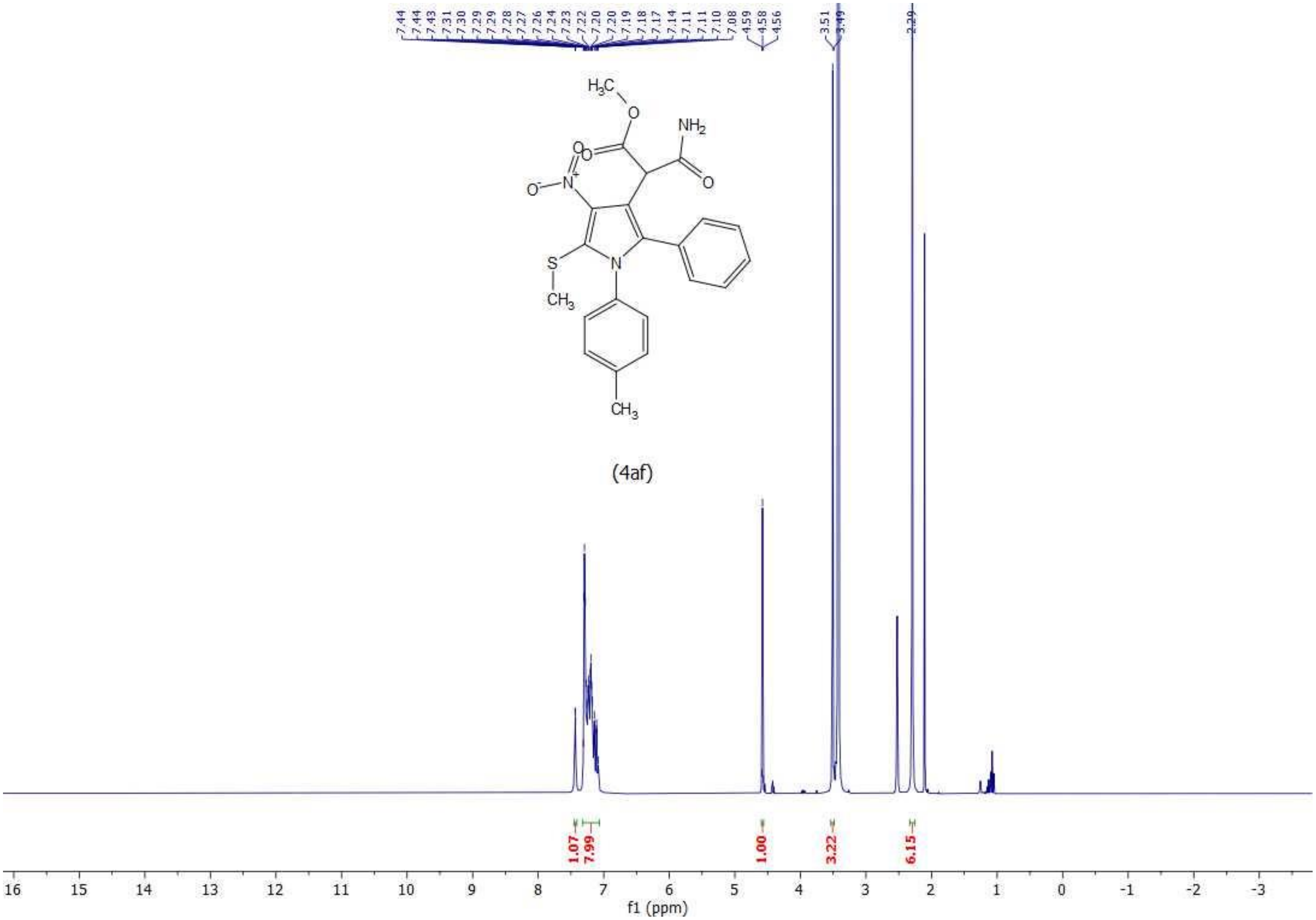
7.44
7.44
7.43
7.31
7.30
7.29
7.29
7.28
7.27
7.26
7.24
7.23
7.22
7.20
7.20
7.19
7.18
7.17
7.14
7.11
7.11
7.10
7.08
4.59
4.58
4.56
3.51
3.49
2.29
H_3C
O
NH_2
O
O
O^-
N^+
O
S
N
CH_3
CH_3
(4af)
1.07
7.99
1.00
3.22
6.15
16
15
14
13
12
11
10
9
8
7
6
5
4
3
2
1
0
-1
-2
-3
f1 (ppm)

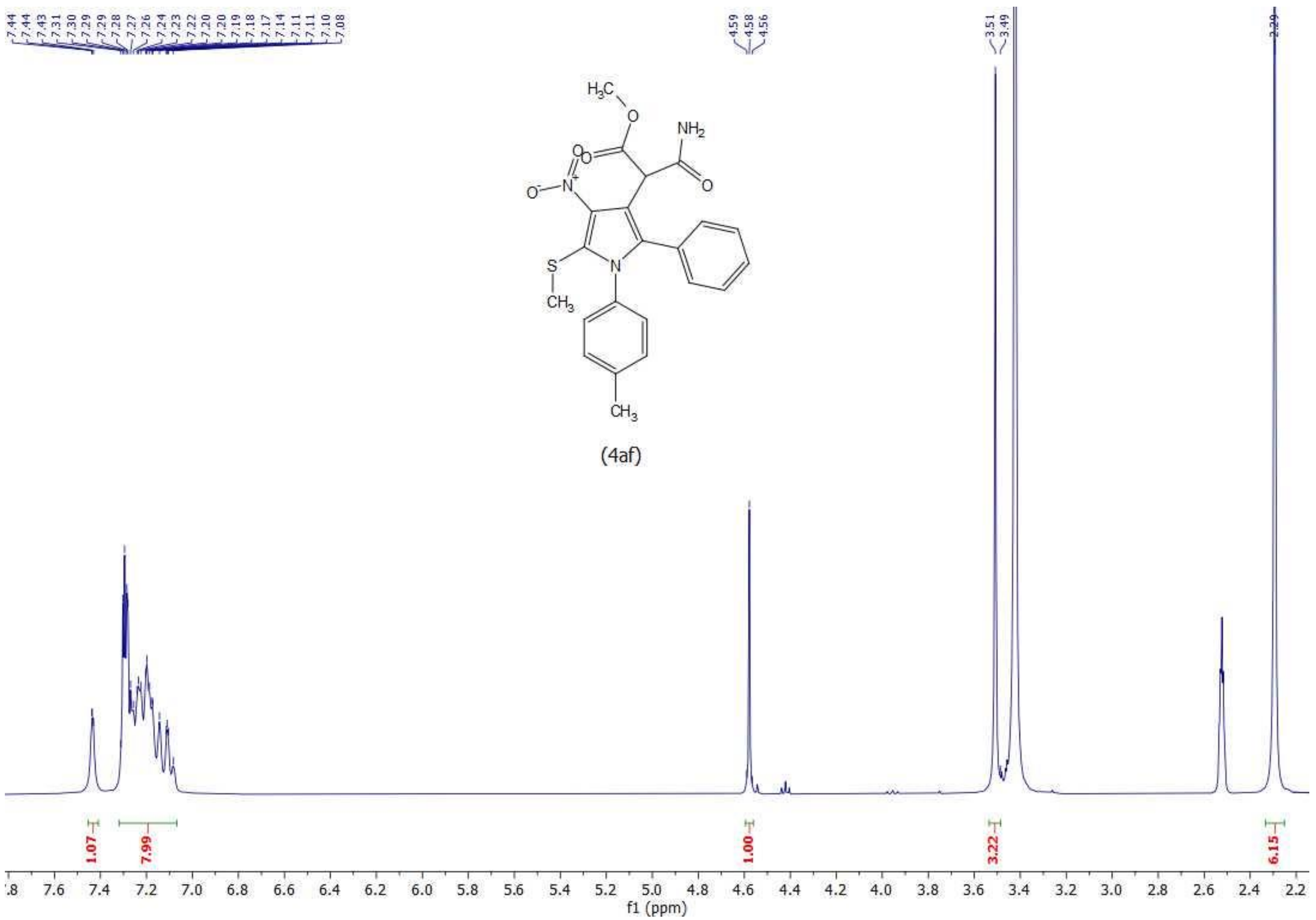
7.44
7.44
7.43
7.31
7.30
7.29
7.29
7.28
7.27
7.26
7.24
7.23
7.22
7.20
7.20
7.19
7.18
7.17
7.14
7.11
7.11
7.10
7.08
4.59
4.58
4.56
3.51
3.49
2.29
(4af)
1.07
7.99
1.00
3.22
6.15
f1 (ppm)

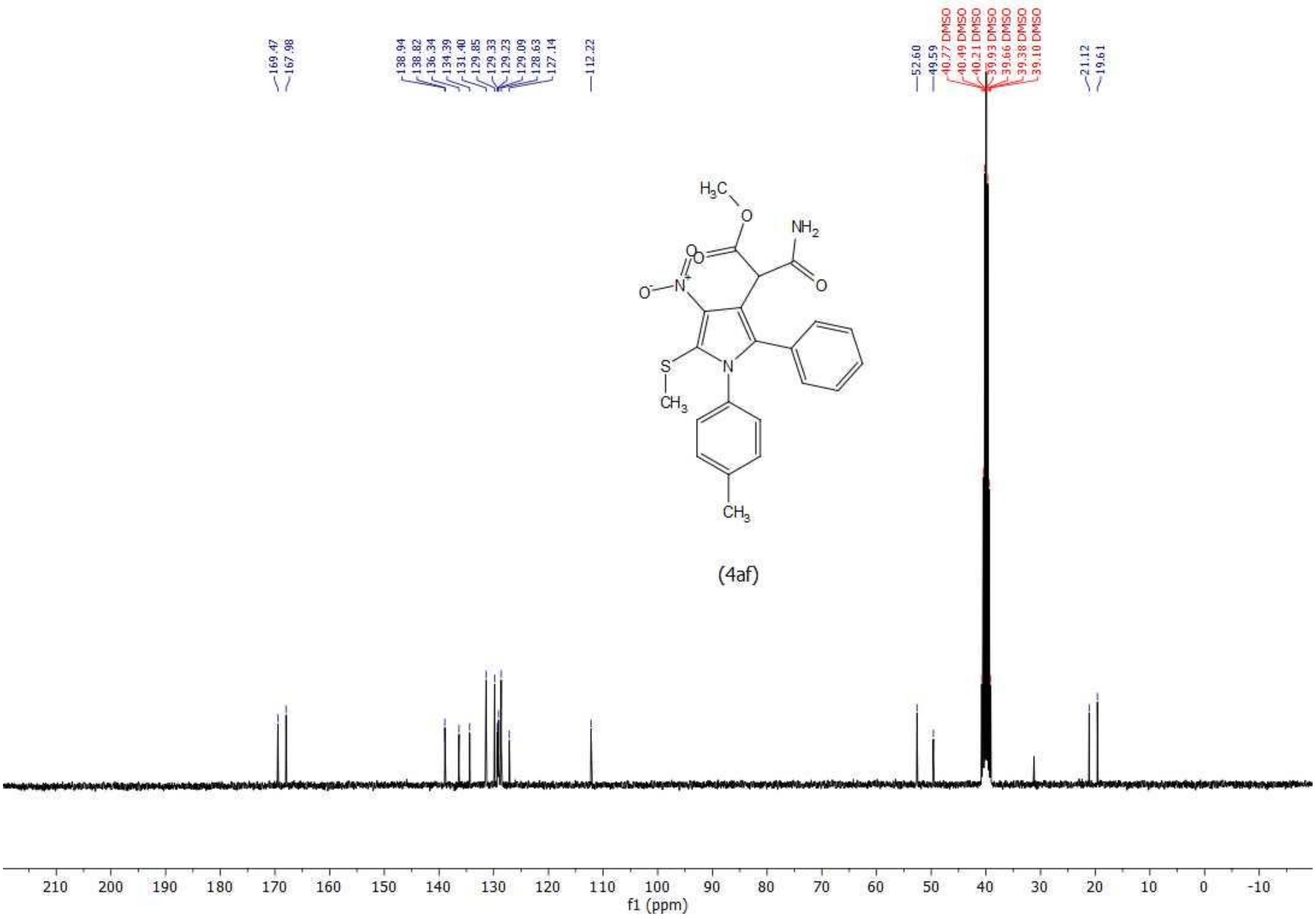
169.47
167.98
138.94
138.82
136.34
134.39
131.40
129.85
129.33
129.23
129.09
128.63
127.14
112.22
52.60
49.59
40.77 DMSO
40.49 DMSO
40.21 DMSO
39.93 DMSO
39.66 DMSO
39.38 DMSO
39.10 DMSO
21.12
19.61
(4af)
210 200 190 180 170 160 150 140 130 120 110 100 90 80 70 60 50 40 30 20 10 0 -10
f1 (ppm)

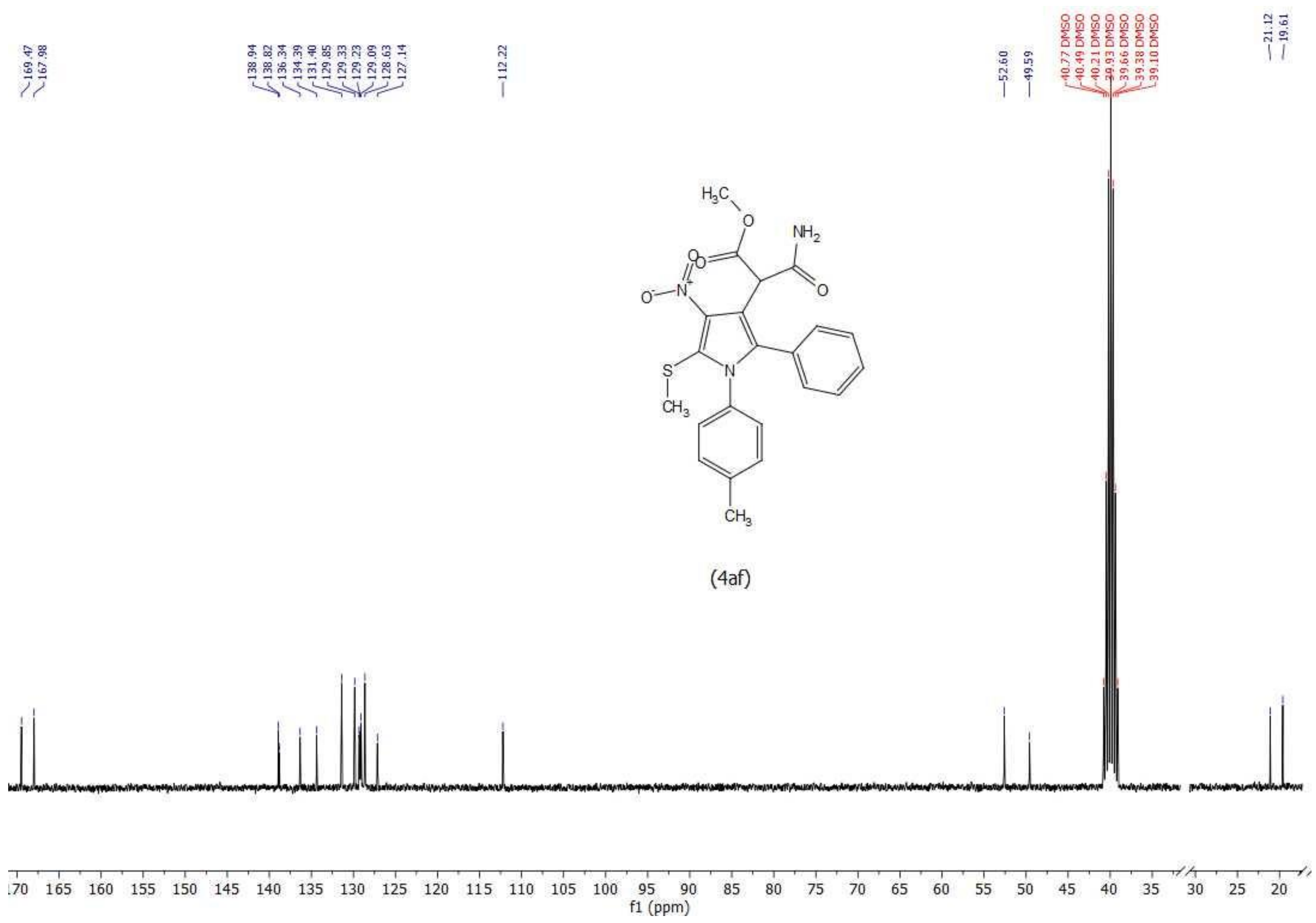
169.47
167.98
138.94
138.82
136.34
134.39
131.40
129.85
129.33
129.23
129.09
128.63
127.14
112.22
52.60
49.59
40.77 DMSO
40.49 DMSO
40.21 DMSO
39.93 DMSO
39.66 DMSO
39.38 DMSO
39.10 DMSO
21.12
19.61
H_3C
O
NH_2
O
O^-
N^+
O
S
CH_3
N
CH_3
170 165 160 155 150 145 140 135 130 125 120 115 110 105 100 95 90 85 80 75 70 65 60 55 50 45 40 35 30 25 20
f1 (ppm)

(4af)

4ag-H

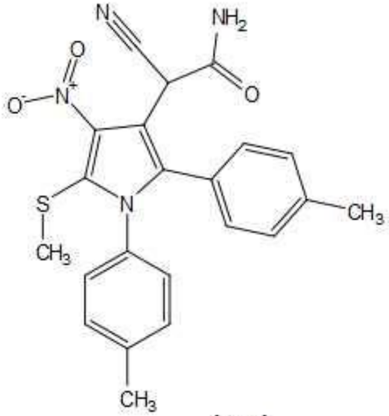

(4ag)

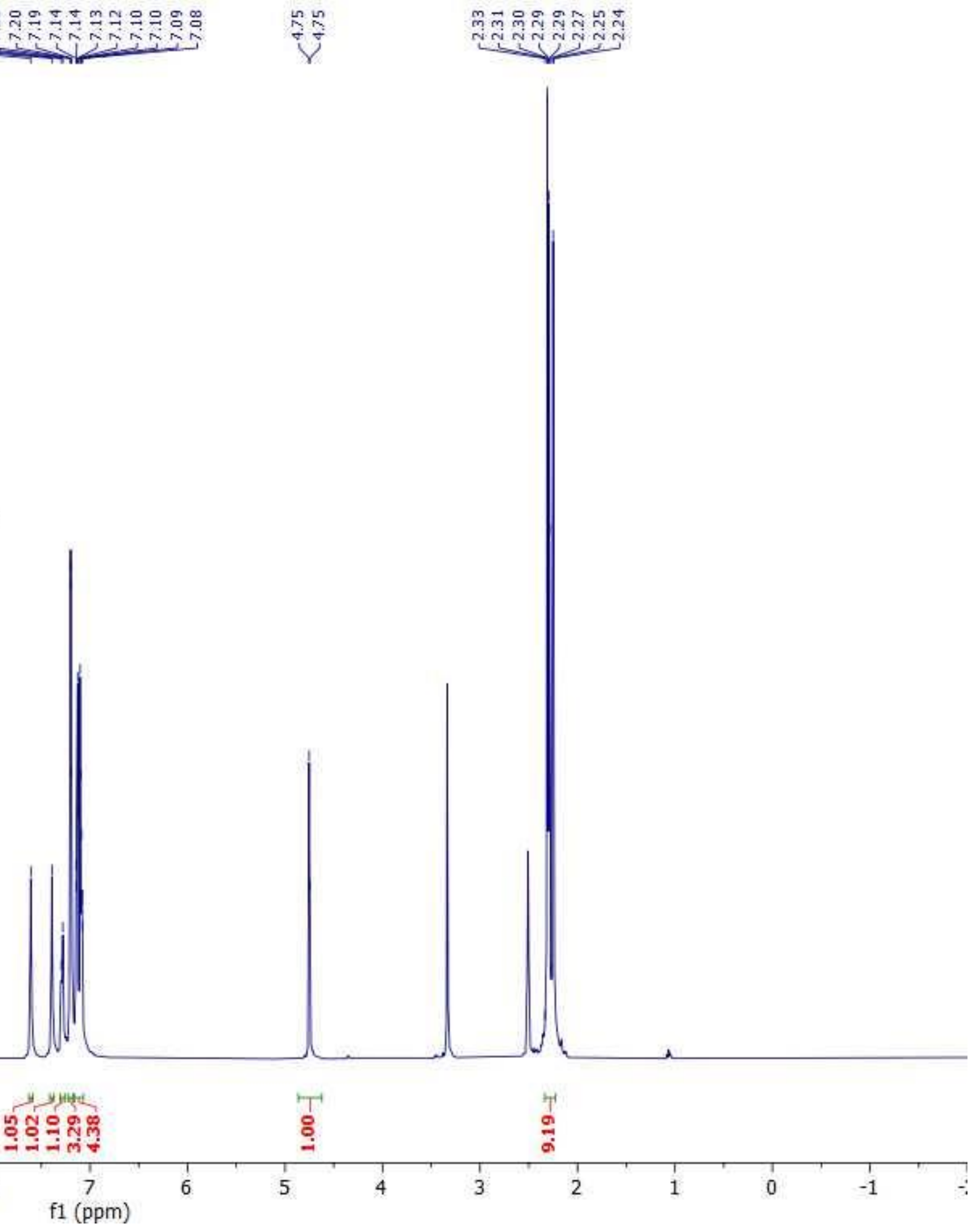
7.61
7.39
7.30
7.28
7.20
7.20
7.19
7.14
7.14
7.13
7.12
7.10
7.10
7.09
7.08
4.75
4.75
2.33
2.31
2.30
2.29
2.29
2.27
2.25
2.24
1.05
1.02
1.10
3.29
4.38
1.00
9.19
f1 (ppm)

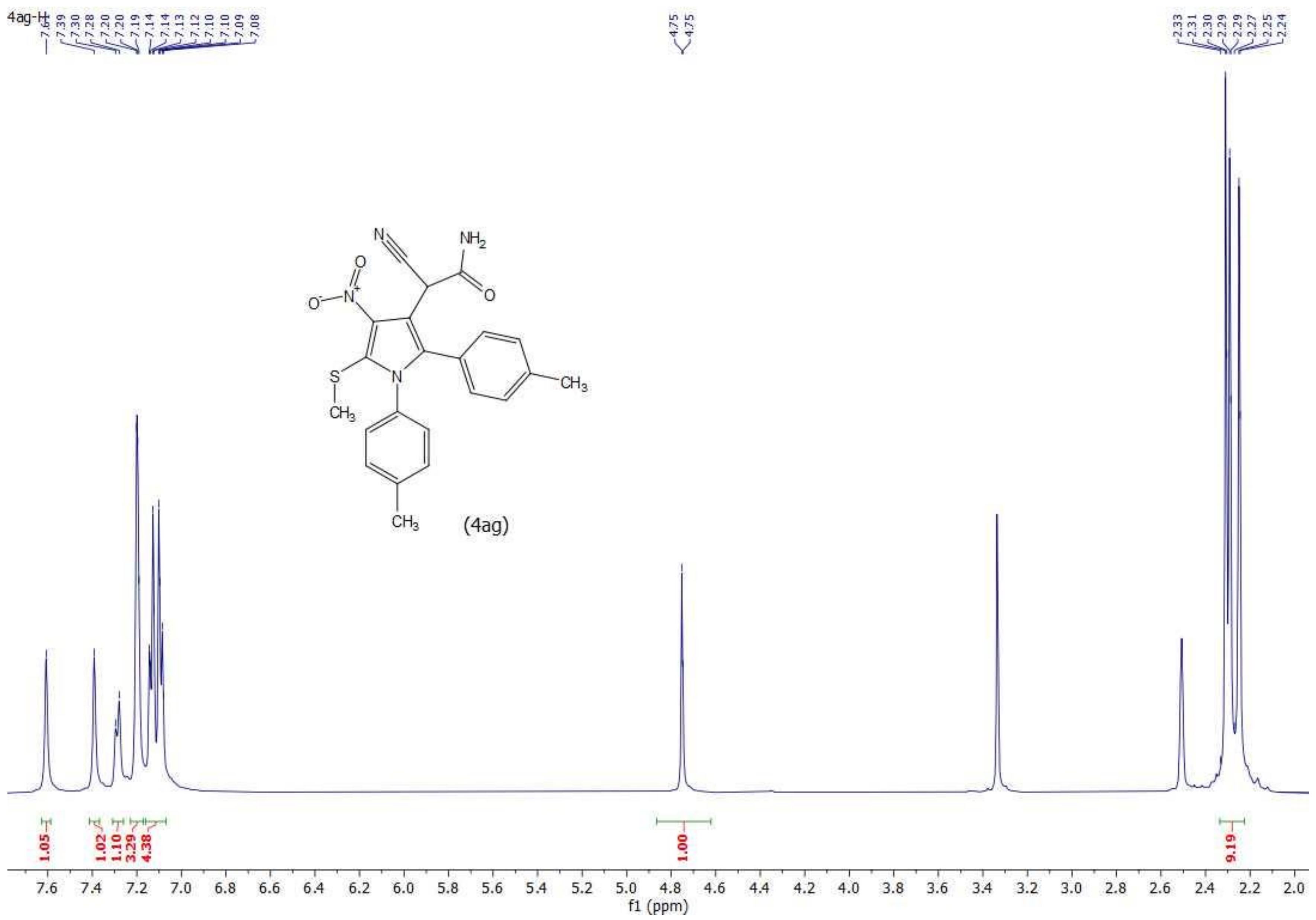
4ag-H
7.61
7.39
7.30
7.28
7.20
7.20
7.19
7.14
7.14
7.13
7.12
7.10
7.10
7.09
7.08
4.75
4.75
2.33
2.31
2.30
2.29
2.29
2.27
2.25
2.24
NH2
N
O
O-
N+
S
CH3
N
CH3
CH3
(4ag)
1.05
1.02
1.10
3.29
4.38
1.00
9.19
7.6 7.4 7.2 7.0 6.8 6.6 6.4 6.2 6.0 5.8 5.6 5.4 5.2 5.0 4.8 4.6 4.4 4.2 4.0 3.8 3.6 3.4 3.2 3.0 2.8 2.6 2.4 2.2 2.0
f1 (ppm)

4ag-C

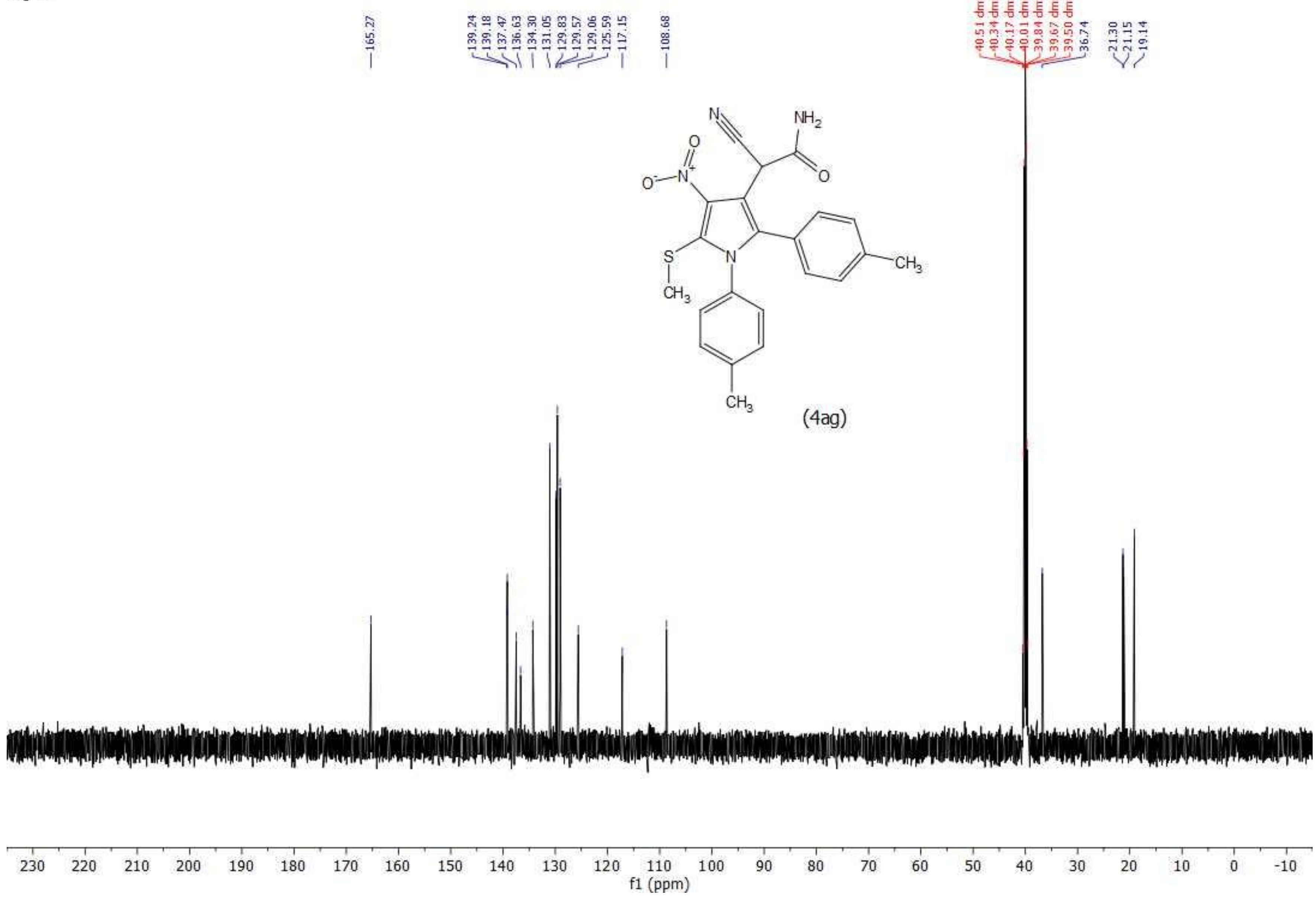
165.27
139.24
139.18
137.47
136.63
134.30
131.05
129.83
129.57
129.06
125.59
117.15
108.68
40.51 dmso
40.34 dmso
40.17 dmso
40.01 dmso
39.84 dmso
39.67 dmso
39.50 dmso
36.74
21.30
21.15
19.14
NH2
O
N
S
CH3
(4ag)
230 220 210 200 190 180 170 160 150 140 130 120 110 100 90 80 70 60 50 40 30 20 10 0 -10
f1 (ppm)

4ag-C

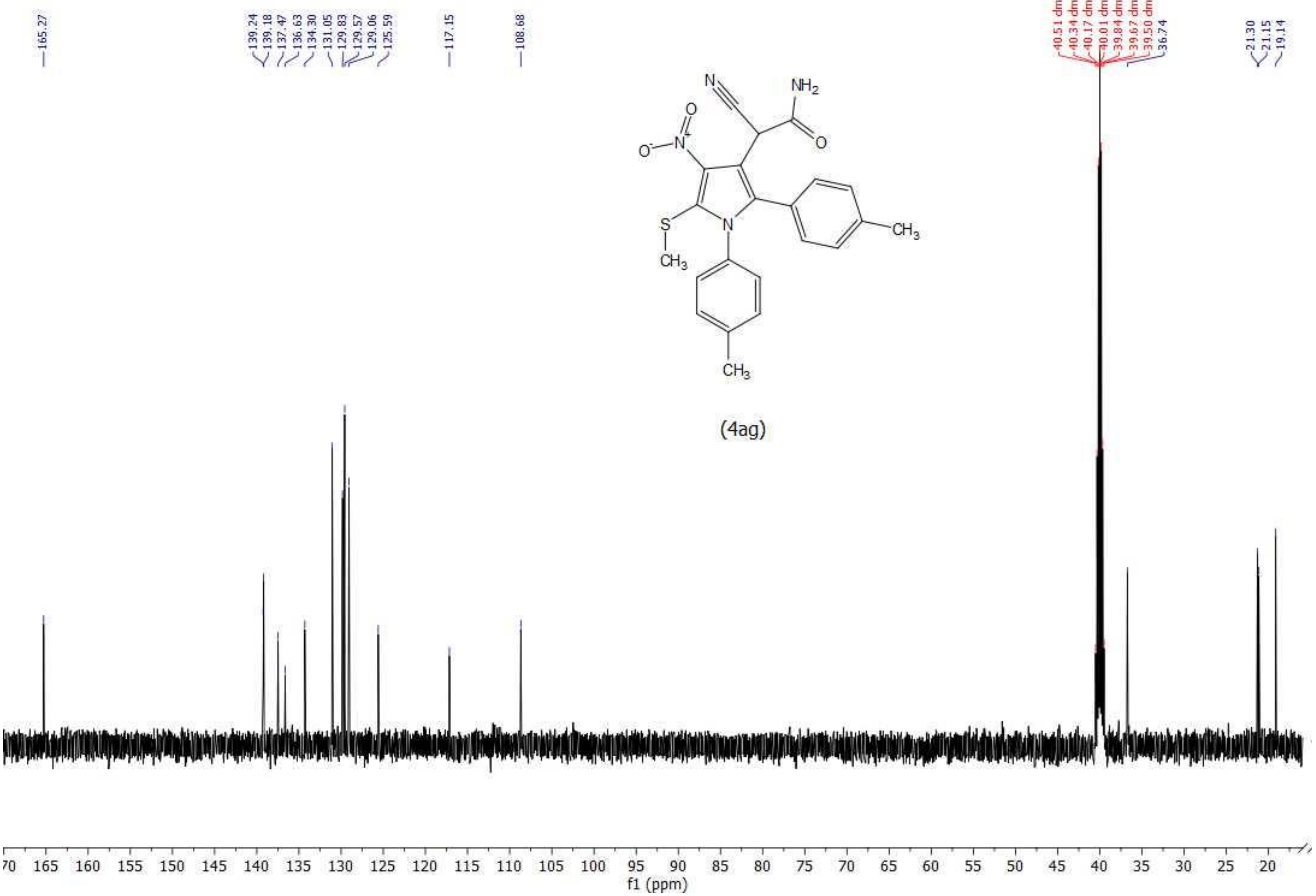

165.27
139.24
139.18
137.47
136.63
134.30
131.05
129.83
129.57
129.06
125.59
117.15
108.68
40.51 dmso
40.34 dmso
40.17 dmso
40.01 dmso
39.84 dmso
39.67 dmso
39.50 dmso
36.74
21.30
21.15
19.14
NH2
O
N
O
O-
N
S
CH3
N
CH3
CH3
f1 (ppm)

(4ag)

4ah-H

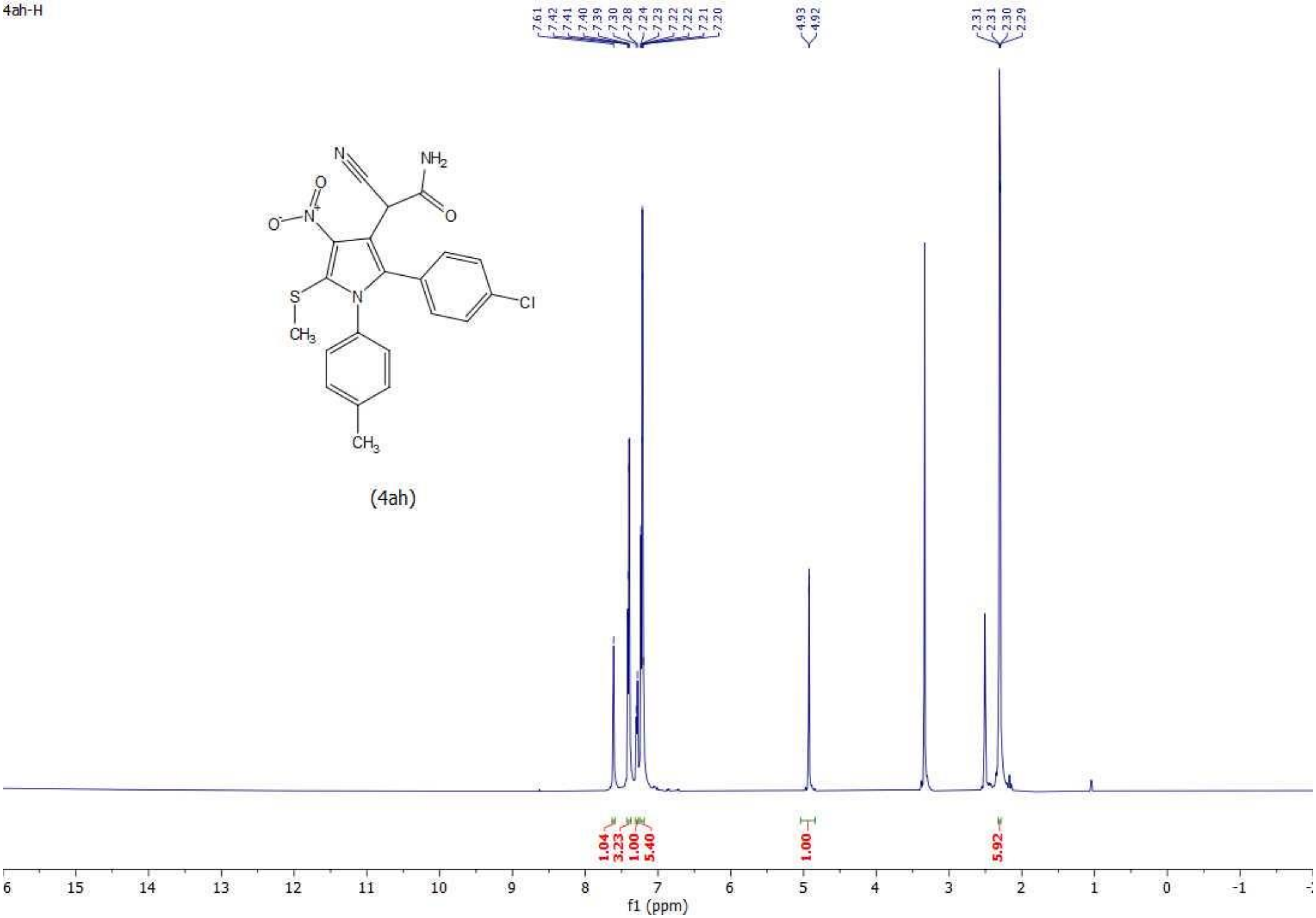
(4ah)
7.61
7.42
7.41
7.40
7.39
7.30
7.28
7.24
7.23
7.22
7.22
7.21
7.20
4.93
4.92
2.31
2.31
2.30
2.29
1.04
3.23
1.00
5.40
1.00
5.92
f1 (ppm)

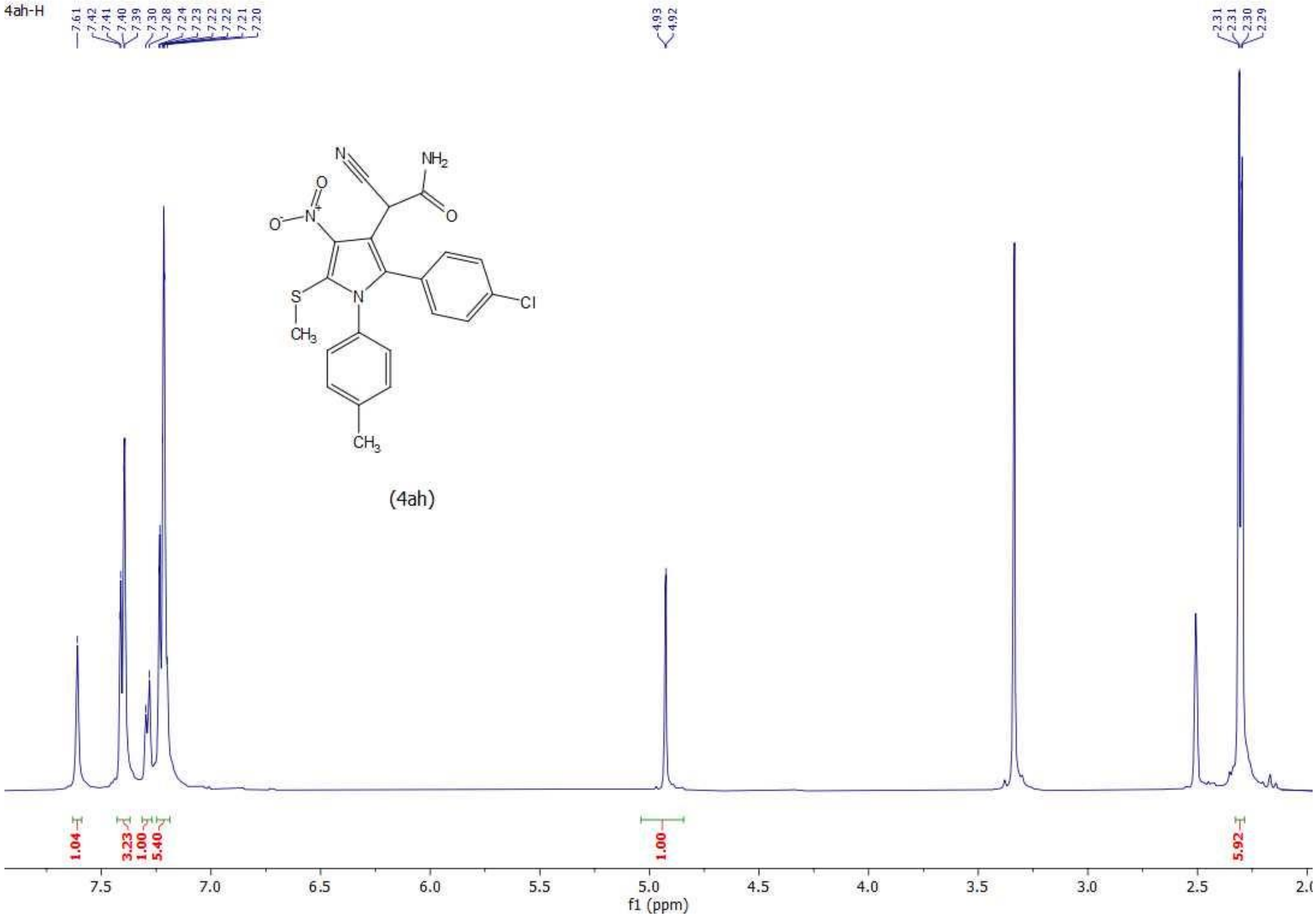
4ah-H
7.61
7.42
7.41
7.40
7.39
7.30
7.28
7.24
7.23
7.22
7.22
7.21
7.20
4.93
4.92
2.31
2.31
2.30
2.29
N
NH2
O
O-
N+
S
CH3
N
Cl
CH3
(4ah)
1.04
3.23
1.00
5.40
1.00
5.92
7.5
7.0
6.5
6.0
5.5
5.0
4.5
4.0
3.5
3.0
2.5
f1 (ppm)

4ah-C

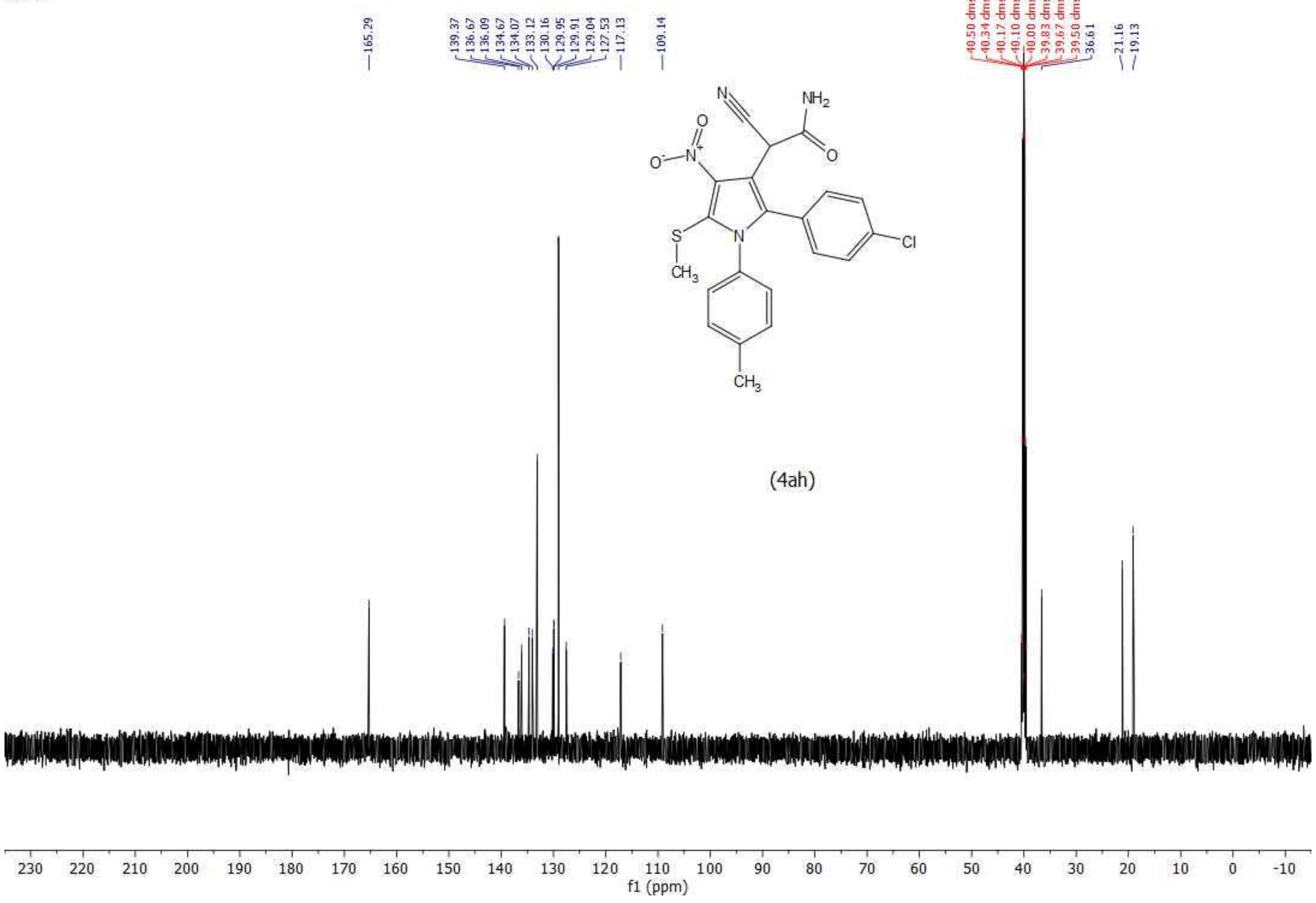
165.29
139.37
136.67
136.09
134.67
134.07
133.12
130.16
129.95
129.91
129.04
127.53
117.13
109.14
40.50 dmso
40.34 dmso
40.17 dmso
40.10 dmso
40.00 dmso
39.83 dmso
39.67 dmso
39.50 dmso
36.61
21.16
19.13
(4ah)
f1 (ppm)

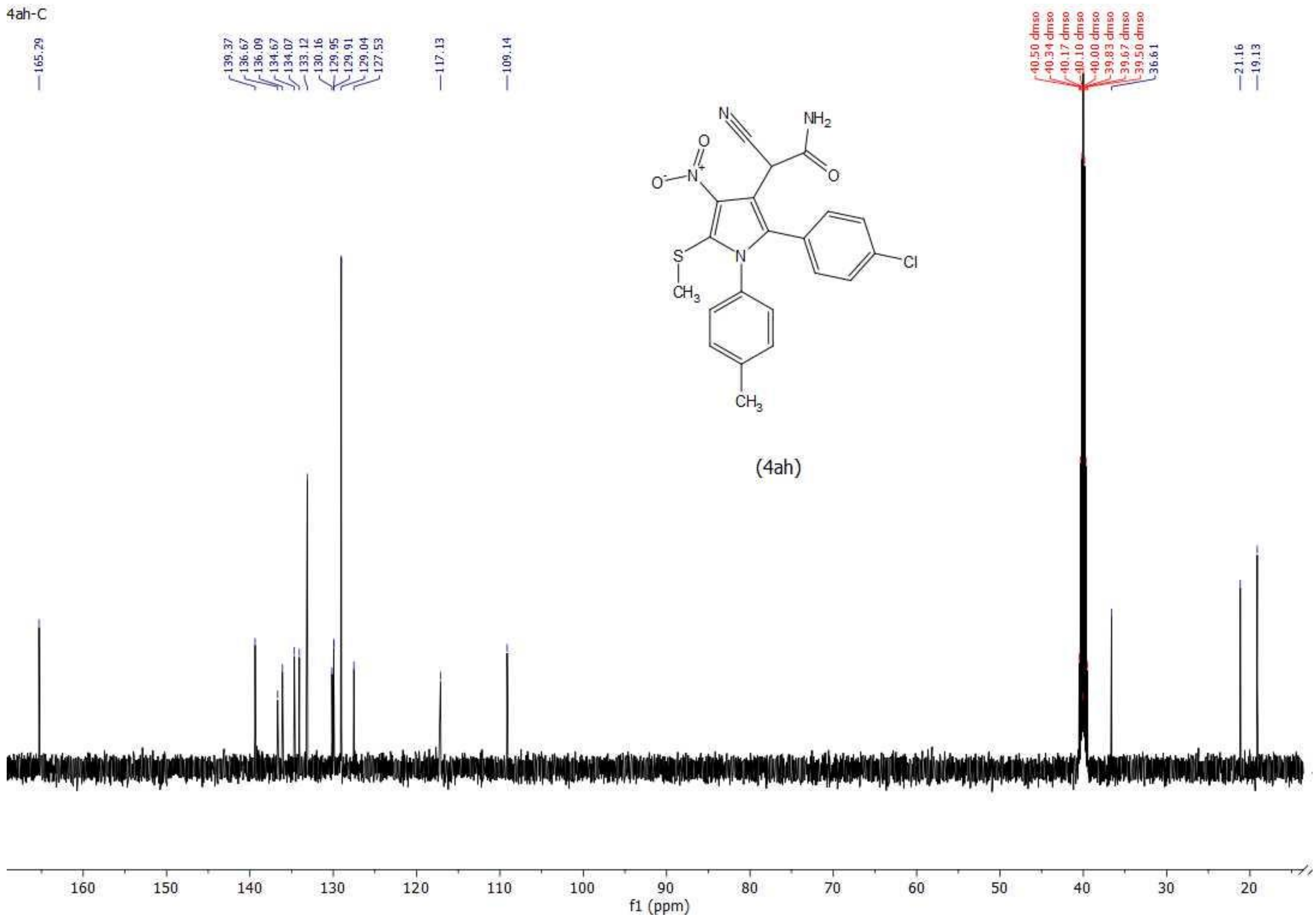
4ah-C
165.29
139.37
136.67
136.09
134.67
134.07
133.12
130.16
129.95
129.91
129.04
127.53
117.13
109.14
40.50 dmso
40.34 dmso
40.17 dmso
40.10 dmso
40.00 dmso
39.83 dmso
39.67 dmso
39.50 dmso
36.61
21.16
19.13
NH2
O
N
O
O-
N+
S
CH3
N
Cl
CH3
(4ah)
160
150
140
130
120
110
100
90
80
70
60
50
40
30
20
f1 (ppm)

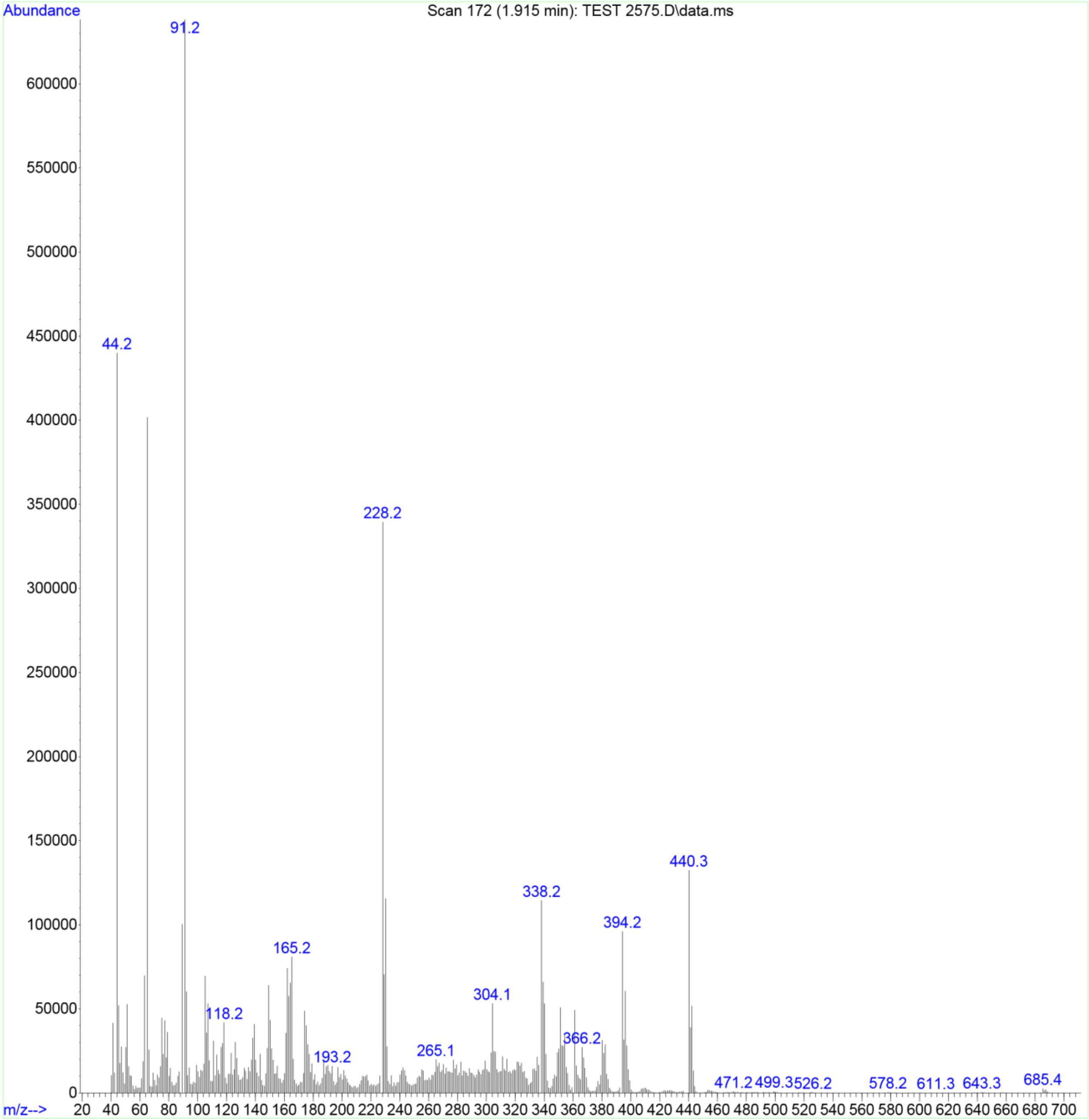

$C_{21}H_{17}ClN_4O_3S$

(440/9)

(4ah)

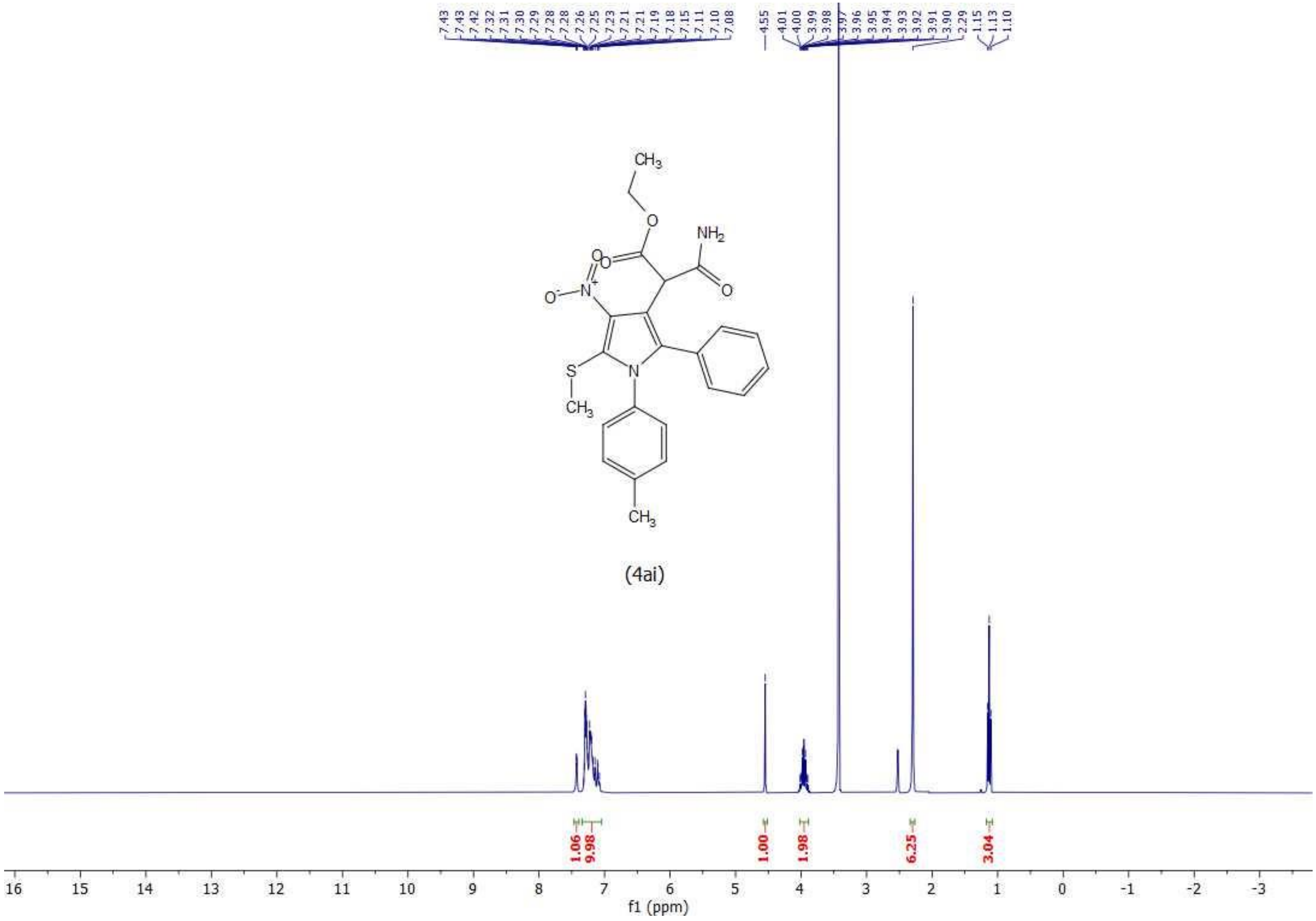
7.43
7.43
7.42
7.32
7.31
7.30
7.29
7.28
7.28
7.26
7.25
7.23
7.21
7.21
7.19
7.18
7.15
7.11
7.10
7.08
4.55
4.01
4.00
3.99
3.98
3.97
3.96
3.95
3.94
3.93
3.92
3.91
3.90
2.29
1.15
1.13
1.10
CH3
NH2
O
O
O
N+
O-
S
CH3
N
CH3
1.06
9.98
1.00
1.98
6.25
3.04
16
15
14
13
12
11
10
9
8
7
6
5
4
3
2
1
0
-1
-2
-3
f1 (ppm)

(4ai)

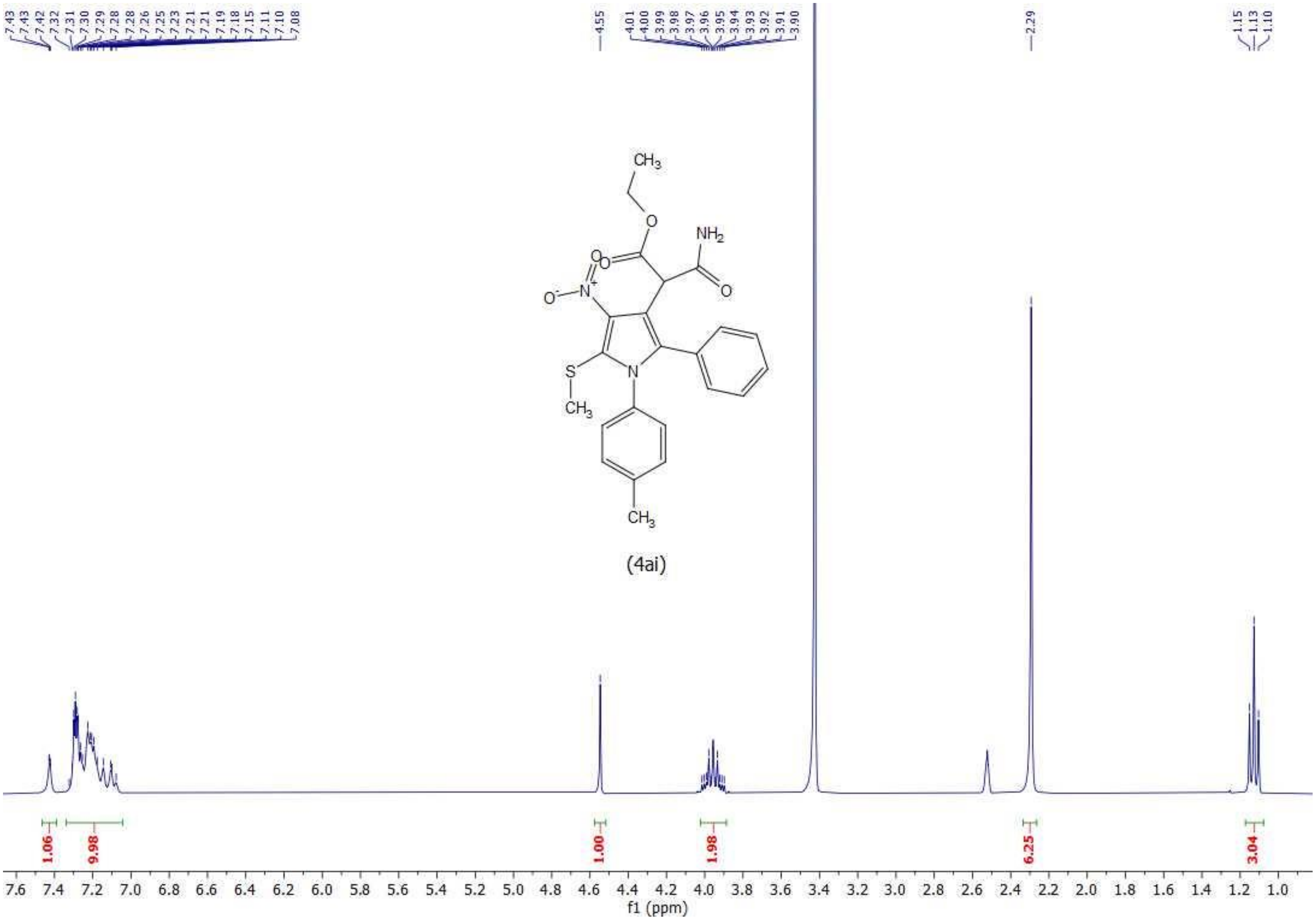
7.43
7.43
7.42
7.32
7.31
7.30
7.29
7.29
7.28
7.28
7.26
7.25
7.23
7.21
7.21
7.19
7.18
7.15
7.11
7.10
7.08
4.55
4.01
4.00
3.99
3.98
3.97
3.96
3.95
3.94
3.93
3.92
3.91
3.90
2.29
1.15
1.13
1.10
CH3
NH2
O
O
O
N+
O-
S
CH3
N
CH3
(4ai)
1.06
9.98
1.00
1.98
6.25
3.04
7.6 7.4 7.2 7.0 6.8 6.6 6.4 6.2 6.0 5.8 5.6 5.4 5.2 5.0 4.8 4.6 4.4 4.2 4.0 3.8 3.6 3.4 3.2 3.0 2.8 2.6 2.4 2.2 2.0 1.8 1.6 1.4 1.2 1.0
f1 (ppm)

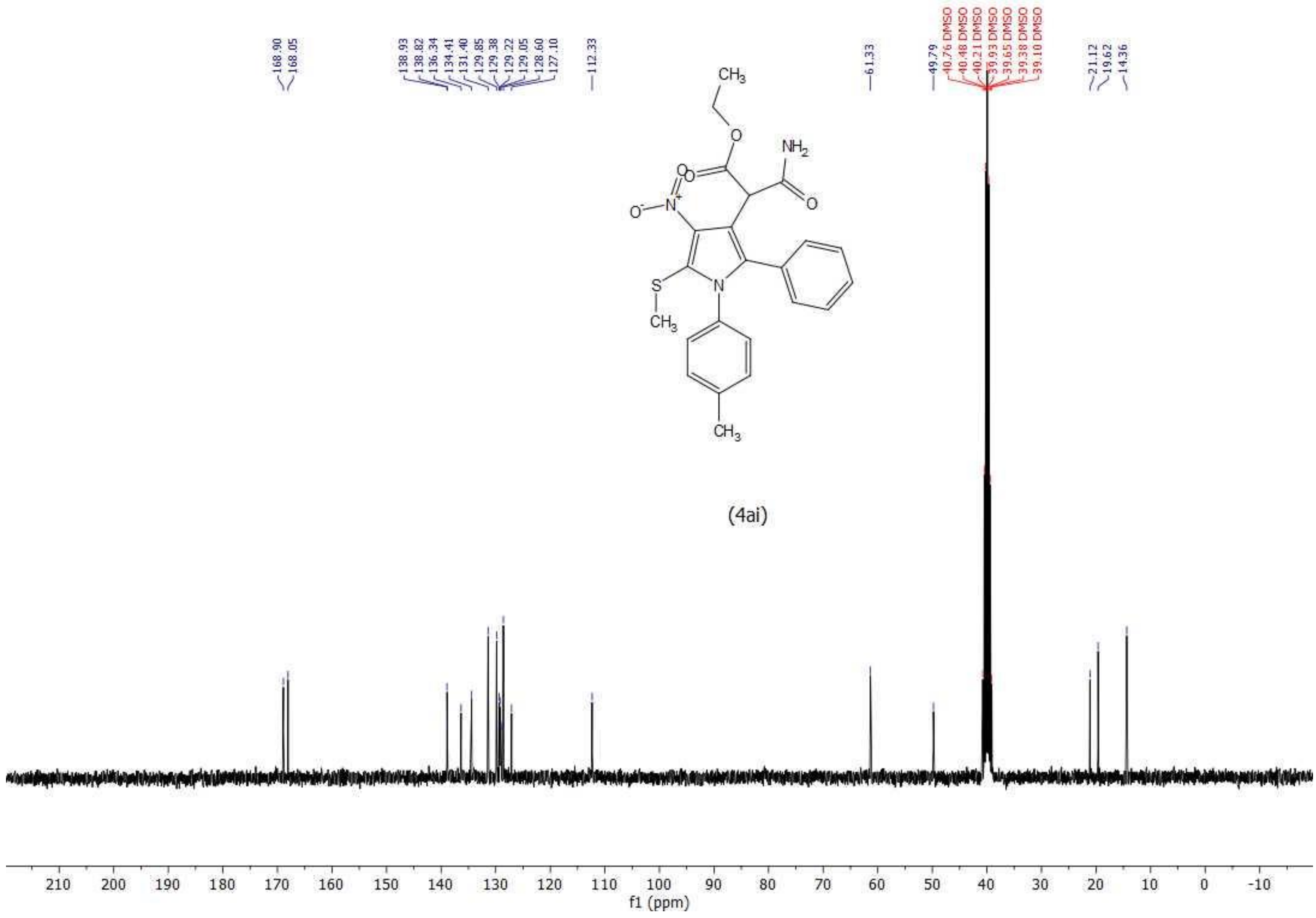
168.90
168.05
138.93
138.82
136.34
134.41
131.40
129.85
129.38
129.22
129.05
128.60
127.10
112.33
61.33
49.79
40.76 DMSO
40.48 DMSO
40.21 DMSO
39.93 DMSO
39.65 DMSO
39.38 DMSO
39.10 DMSO
21.12
19.62
14.36
CH3
O
NH2
O
O
O-
N+
S
N
CH3
CH3
(4ai)
210 200 190 180 170 160 150 140 130 120 110 100 90 80 70 60 50 40 30 20 10 0 -10
f1 (ppm)

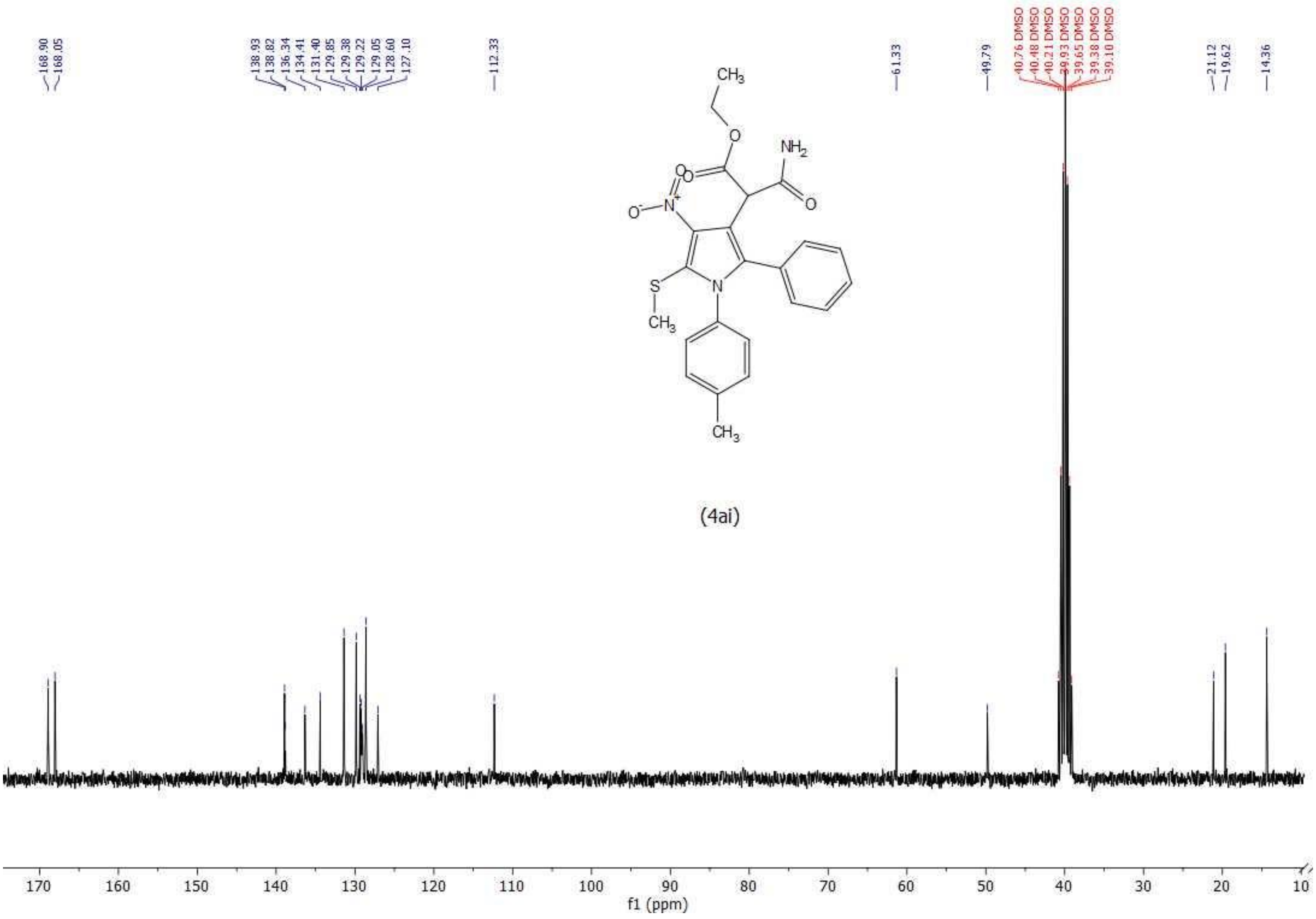
168.90
168.05
138.93
138.82
136.34
134.41
131.40
129.85
129.38
129.22
129.05
128.60
127.10
112.33
61.33
49.79
40.76 DMSO
40.48 DMSO
40.21 DMSO
39.93 DMSO
39.65 DMSO
39.38 DMSO
39.10 DMSO
21.12
19.62
14.36
CH3
O
NH2
O
O
O
N+
O-
S
CH3
N
CH3
(4ai)
170 160 150 140 130 120 110 100 90 80 70 60 50 40 30 20 10
f1 (ppm)

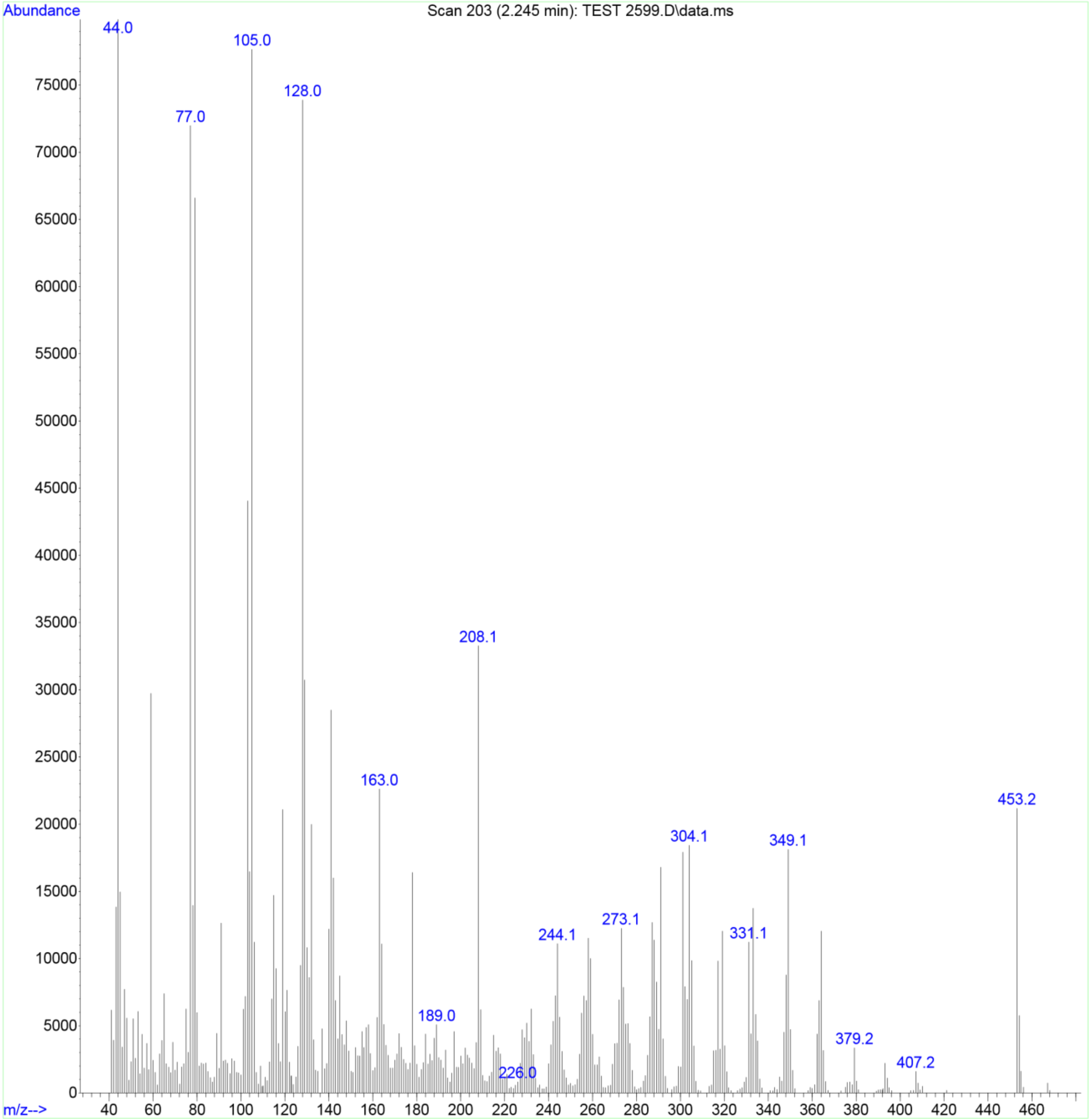

$C_{23}H_{23}N_3O_5S$

(453/5)

(4ai)

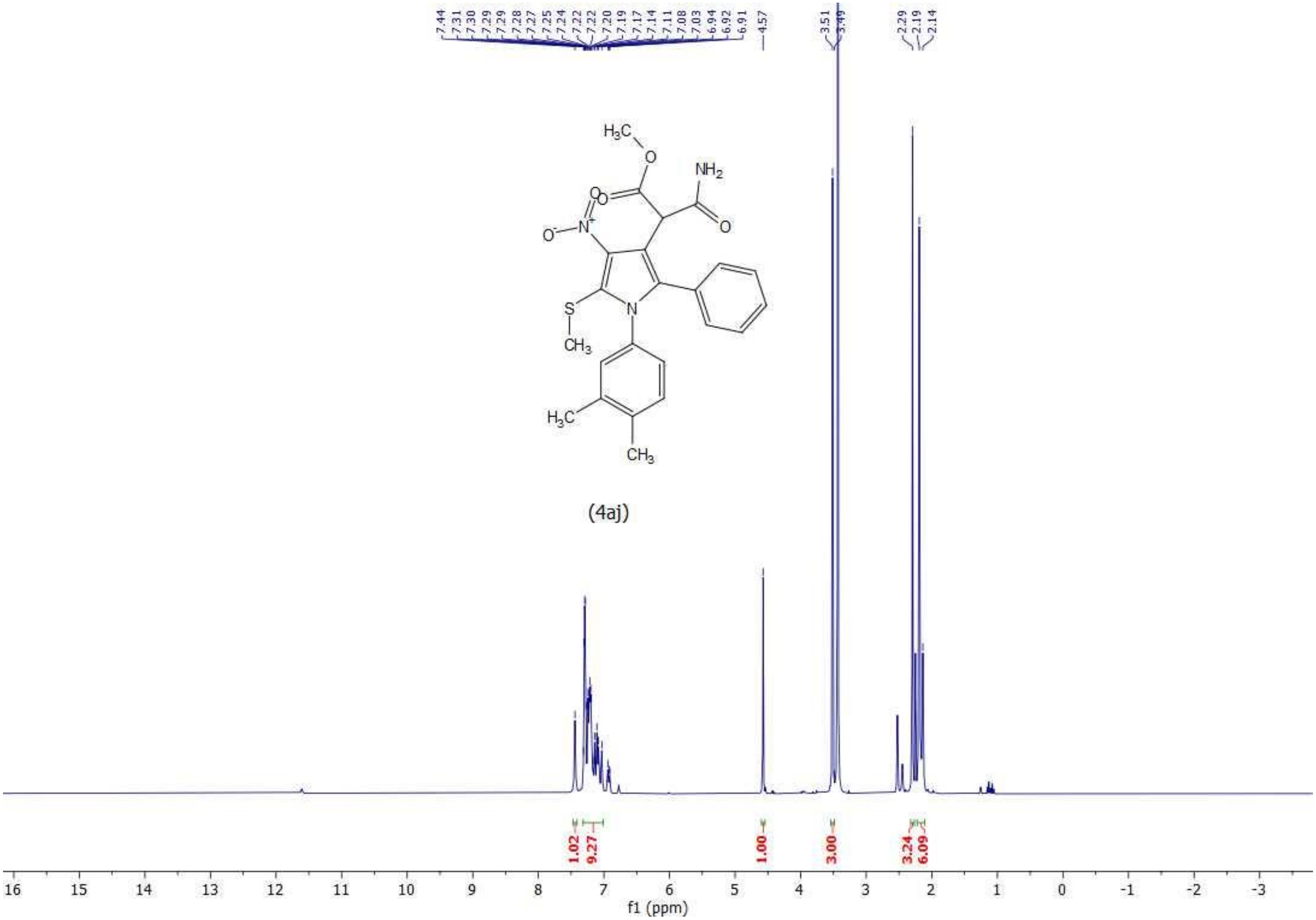
7.44 7.31 7.30 7.29 7.29 7.28 7.27 7.25 7.24 7.22 7.22 7.20 7.19 7.17 7.14 7.11 7.08 7.03 6.94 6.92 6.91
4.57
3.51 3.49
2.29 2.19 2.14
(4aj)
1.02 9.27 1.00 3.00 3.24 6.09
16 15 14 13 12 11 10 9 8 7 6 5 4 3 2 1 0 -1 -2 -3
f1 (ppm)

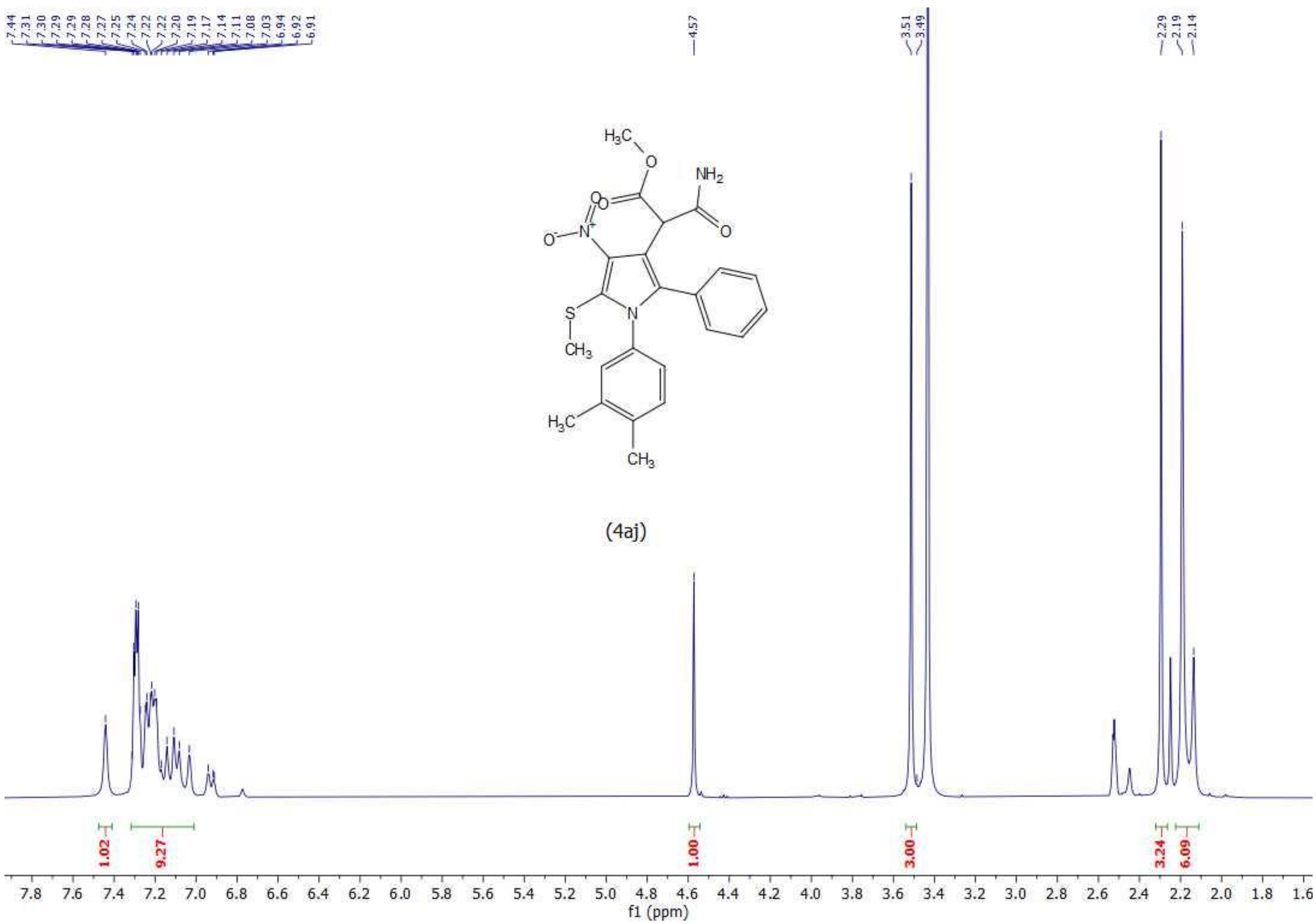
7.44
7.31
7.30
7.29
7.29
7.28
7.27
7.25
7.24
7.22
7.22
7.20
7.19
7.17
7.14
7.11
7.08
7.03
6.94
6.92
6.91
4.57
3.51
3.49
2.29
2.19
2.14
H_3C
O
NH_2
O
O
O^-
N^+
S
N
CH_3
H_3C
CH_3
(4aj)
1.02
9.27
1.00
3.00
3.24
6.09
f1 (ppm)

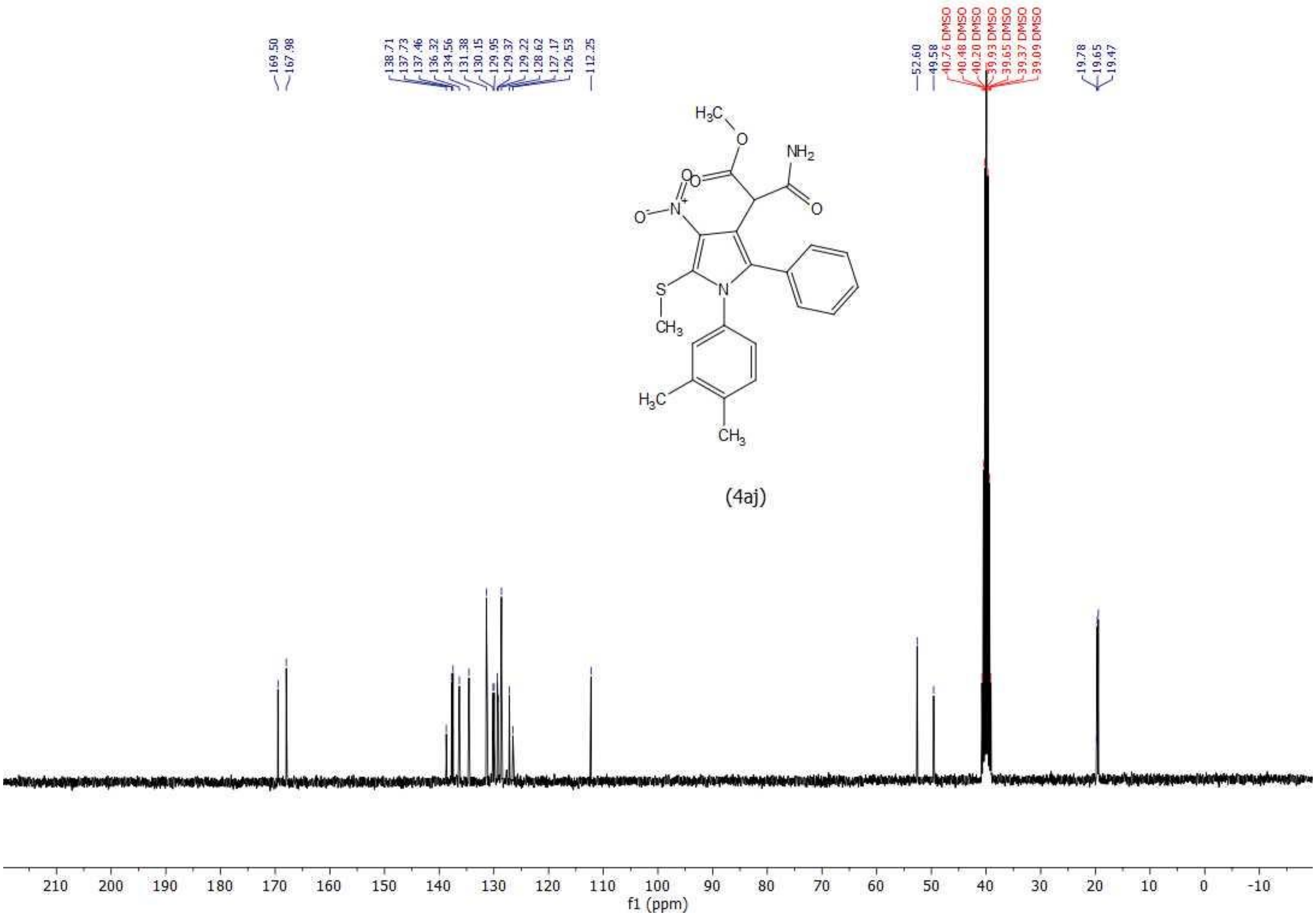
169.50
167.98
138.71
137.73
137.46
136.32
134.56
131.38
130.15
129.95
129.37
129.22
128.62
127.17
126.53
112.25
52.60
49.58
40.76 DMSO
40.48 DMSO
40.20 DMSO
39.93 DMSO
39.65 DMSO
39.37 DMSO
39.09 DMSO
19.78
19.65
19.47
H3C
O
NH2
O
O
O-
N+
S
CH3
N
H3C
CH3
210
200
190
180
170
160
150
140
130
120
110
100
90
80
70
60
50
40
30
20
10
0
-10
f1 (ppm)

(4aj)

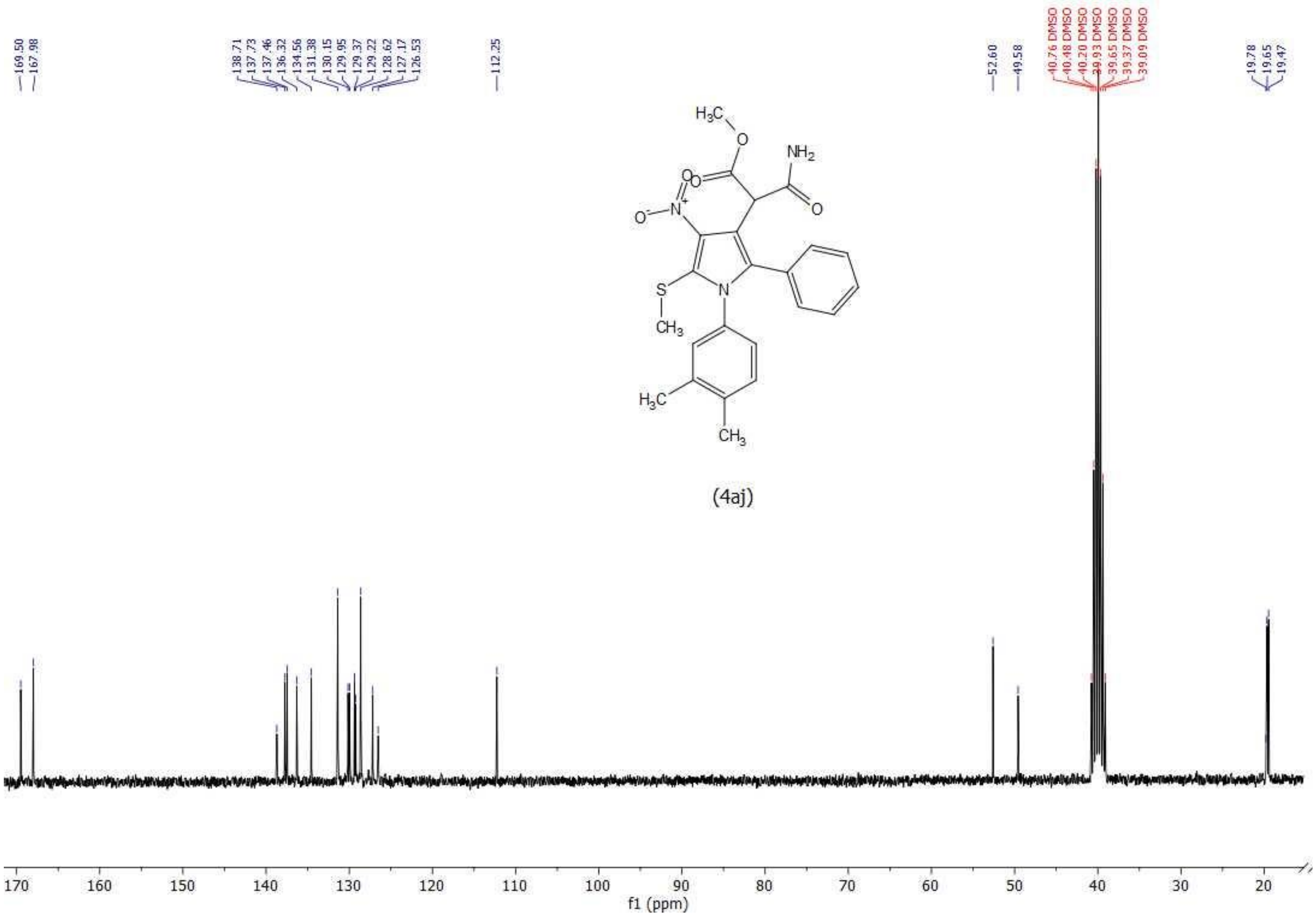
169.50
167.98
138.71
137.73
137.46
136.32
134.56
131.38
130.15
129.95
129.37
129.22
128.62
127.17
126.53
112.25
52.60
49.58
40.76 DMSO
40.48 DMSO
40.20 DMSO
39.93 DMSO
39.65 DMSO
39.37 DMSO
39.09 DMSO
19.78
19.65
19.47
H_3C
O
NH_2
O
O
O^-
N^+
S
N
CH_3
H_3C
CH_3
170
160
150
140
130
120
110
100
90
80
70
60
50
40
30
20
f1 (ppm)
(4aj)

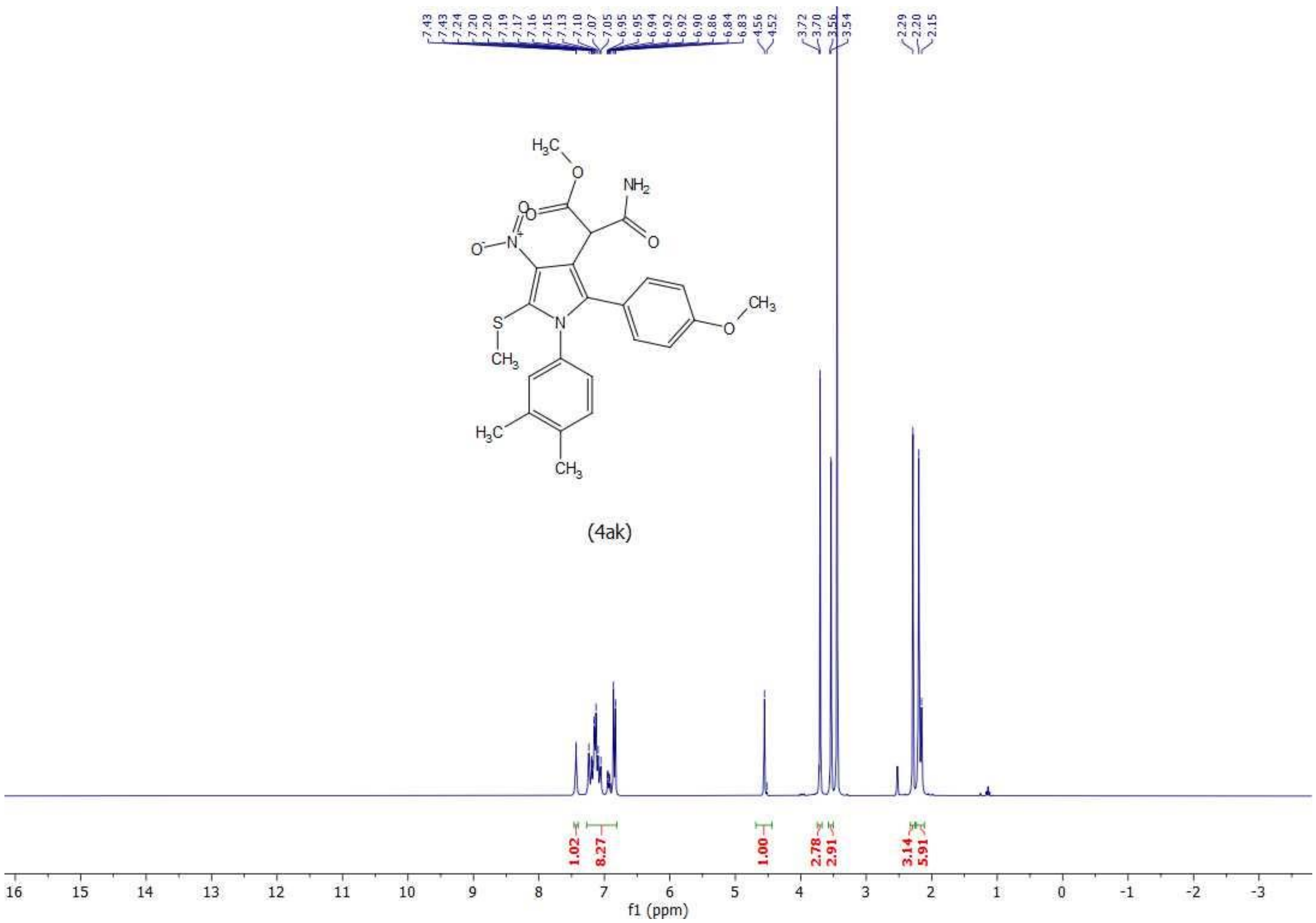
7.43
7.43
7.24
7.20
7.20
7.19
7.17
7.16
7.15
7.13
7.10
7.07
7.05
6.95
6.95
6.94
6.92
6.92
6.90
6.86
6.84
6.83
4.56
4.52
3.72
3.70
3.56
3.54
2.29
2.20
2.15
H_3C
O
NH_2
O
O
O^-
N^+
O
S
CH_3
N
CH_3
O
H_3C
CH_3
1.02
8.27
1.00
2.78
2.91
3.14
5.91
16
15
14
13
12
11
10
9
8
7
6
5
4
3
2
1
0
-1
-2
-3
f1 (ppm)

(4ak)

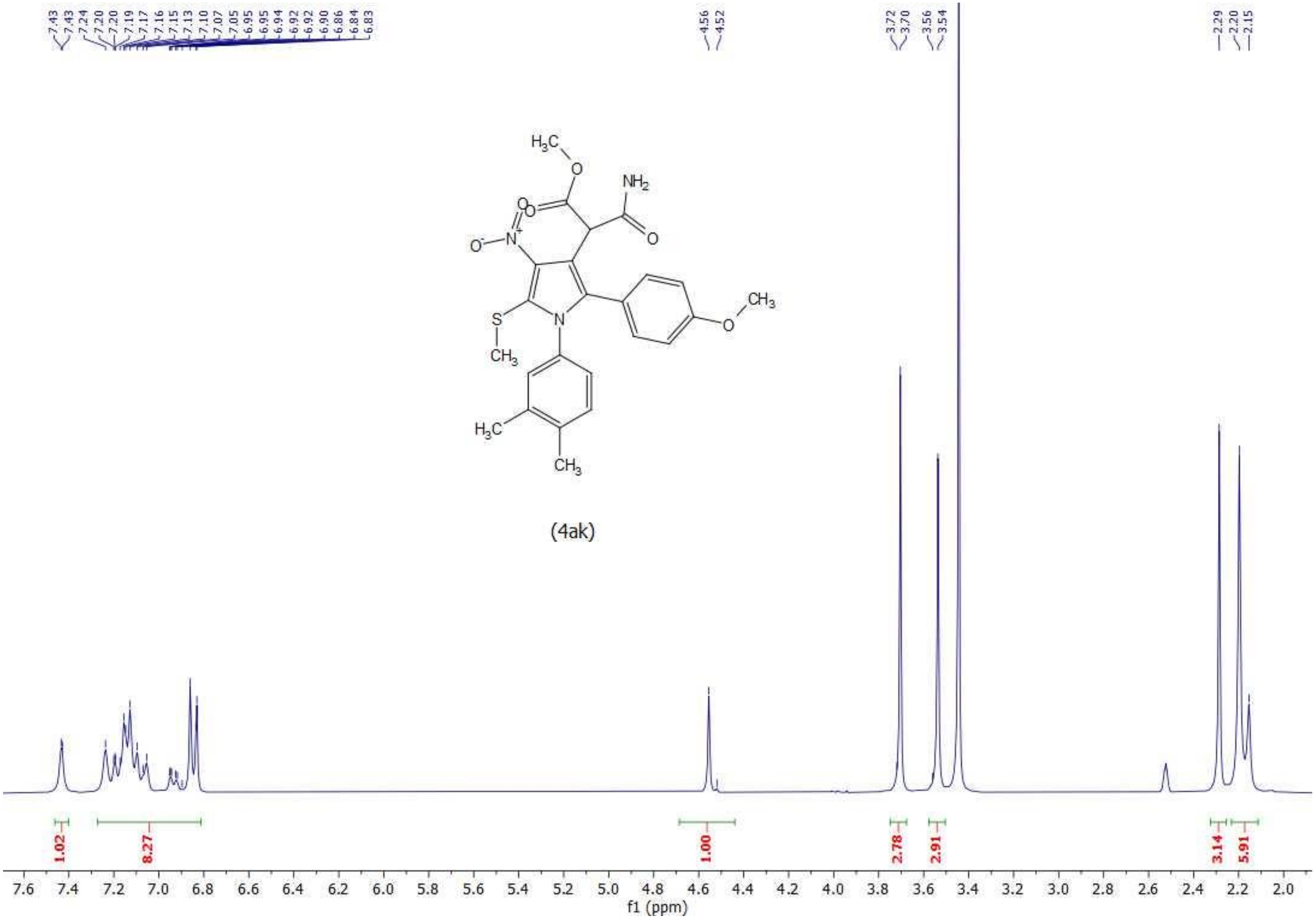

7.43
7.43
7.24
7.20
7.20
7.19
7.17
7.16
7.15
7.13
7.10
7.07
7.05
6.95
6.95
6.94
6.92
6.92
6.90
6.86
6.84
6.83
4.56
4.52
3.72
3.70
3.56
3.54
2.29
2.20
2.15
(4ak)
1.02
8.27
1.00
2.78
2.91
3.14
5.91
7.6 7.4 7.2 7.0 6.8 6.6 6.4 6.2 6.0 5.8 5.6 5.4 5.2 5.0 4.8 4.6 4.4 4.2 4.0 3.8 3.6 3.4 3.2 3.0 2.8 2.6 2.4 2.2 2.0
f1 (ppm)

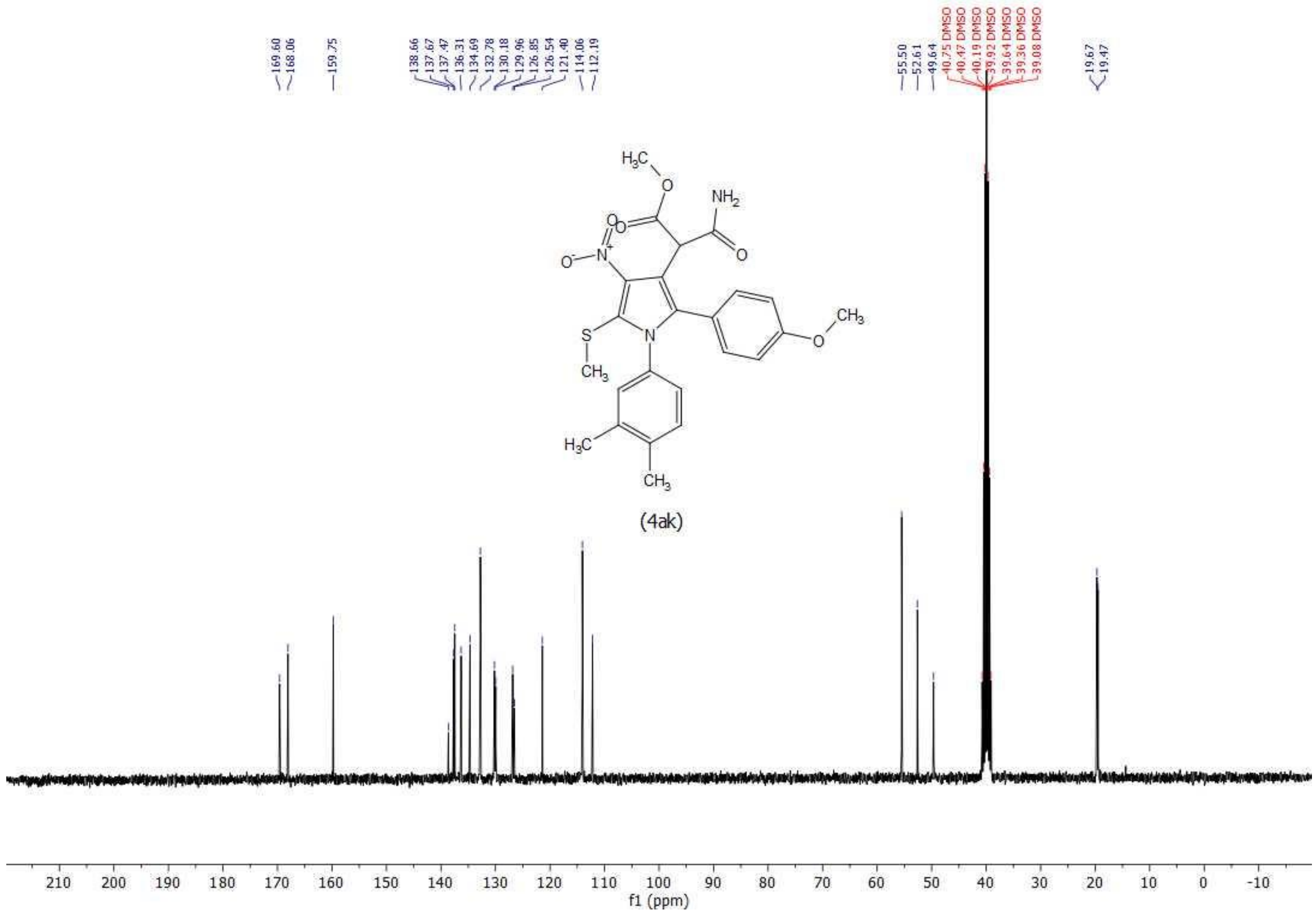

169.60
168.06
159.75
138.66
137.67
137.47
136.31
134.69
132.78
130.18
129.96
126.85
126.54
121.40
114.06
112.19
55.50
52.61
49.64
40.75 DMSO
40.47 DMSO
40.19 DMSO
39.92 DMSO
39.64 DMSO
39.36 DMSO
39.08 DMSO
19.67
19.47
H3C
O
NH2
O
O
N+
O-
CH3
S
CH3
N
H3C
CH3
(4ak)
210 200 190 180 170 160 150 140 130 120 110 100 90 80 70 60 50 40 30 20 10 0 -10
f1 (ppm)

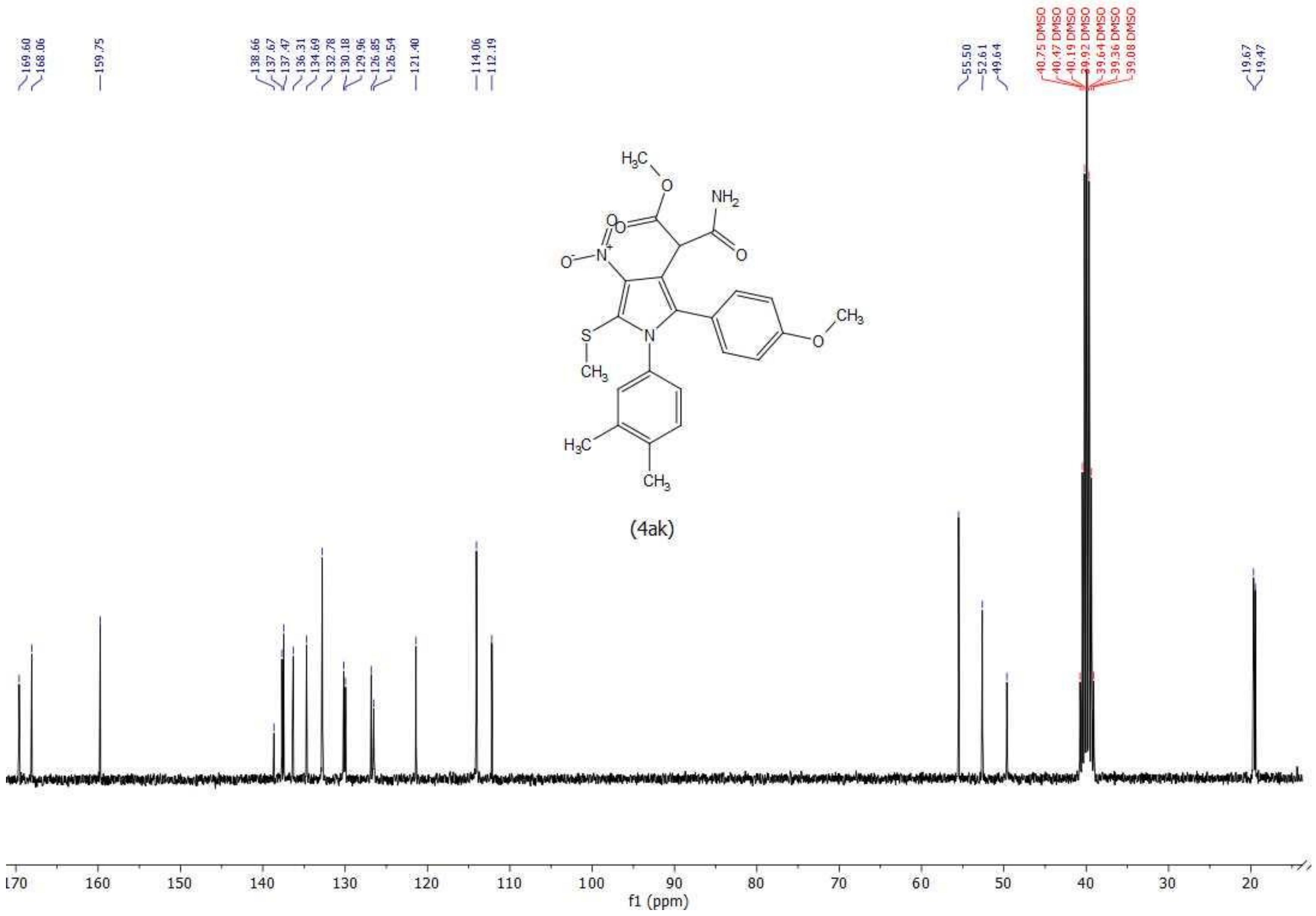

169.60
168.06
159.75
138.66
137.67
137.47
136.31
134.69
132.78
130.18
129.96
126.85
126.54
121.40
114.06
112.19
55.50
52.61
49.64
40.75 DMSO
40.47 DMSO
40.19 DMSO
39.92 DMSO
39.64 DMSO
39.36 DMSO
39.08 DMSO
19.67
19.47
(4ak)
f1 (ppm)

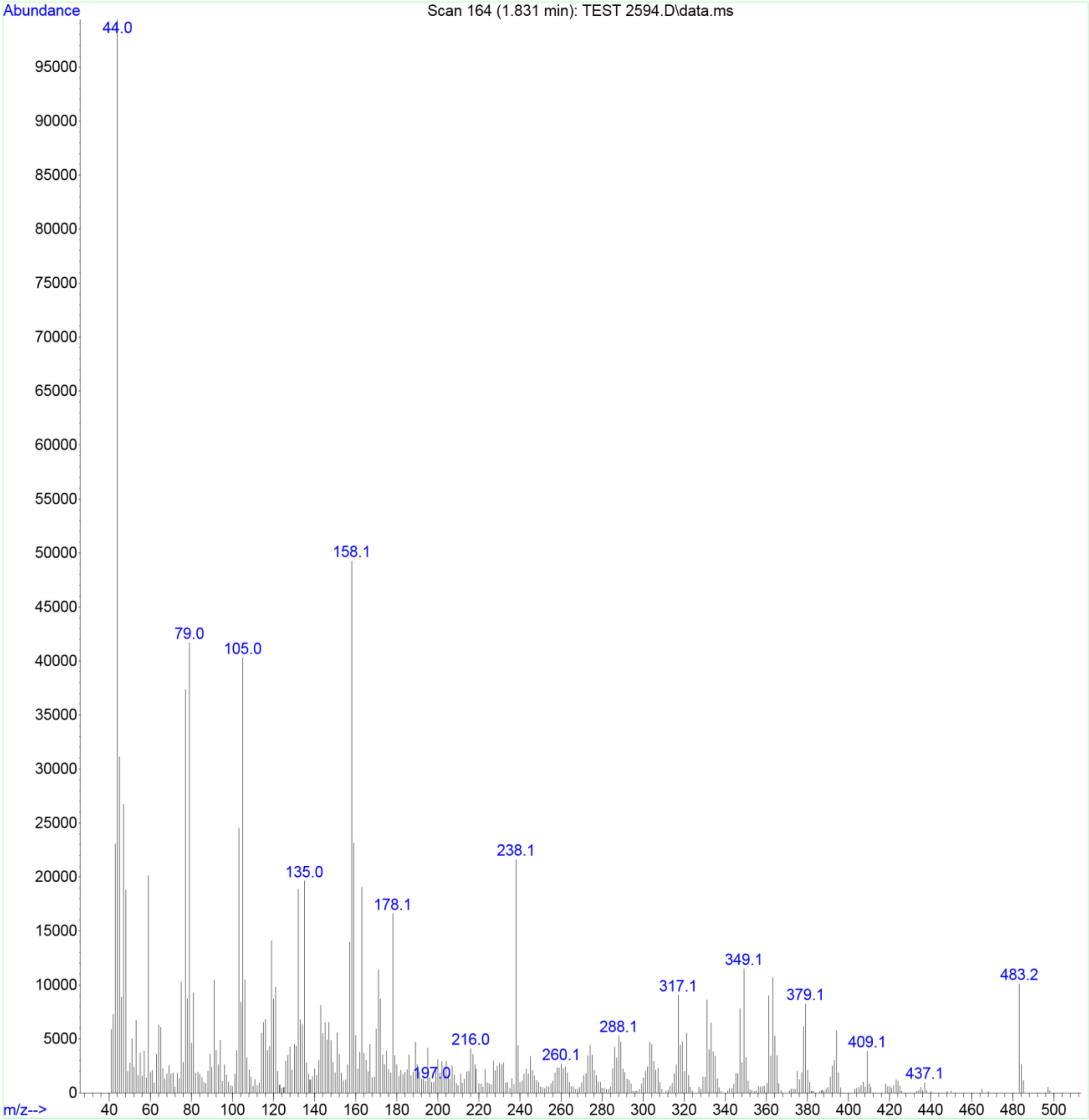

C24H25N3O6S

(483/5)

(4ak)

4al-H

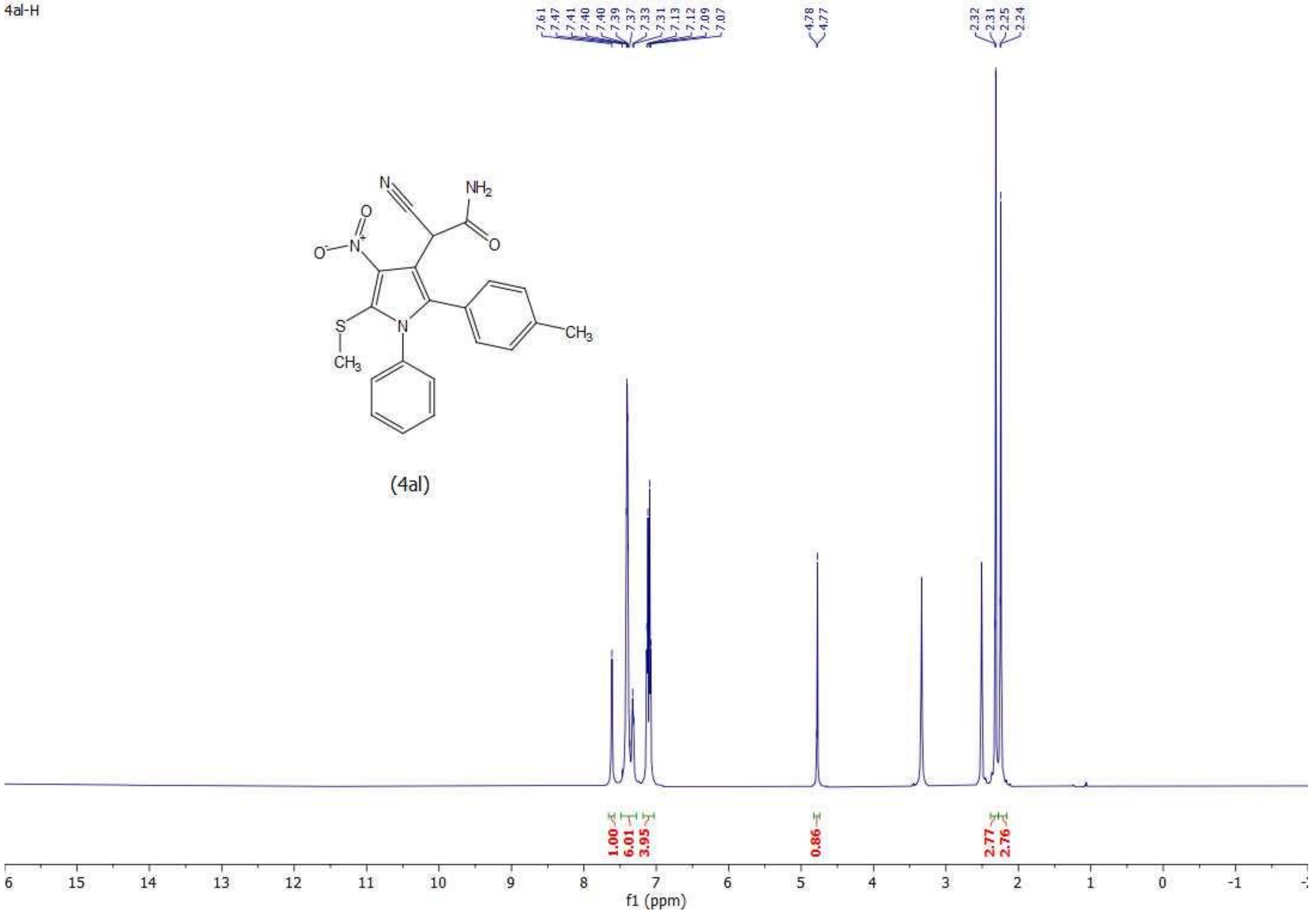
7.61
7.47
7.41
7.40
7.40
7.39
7.37
7.33
7.31
7.13
7.12
7.09
7.07
4.78
4.77
2.32
2.31
2.25
2.24
NH2
N
O
O-
N+
S
CH3
N
CH3
(4al)
1.00
6.01
3.95
0.86
2.77
2.76
f1 (ppm)

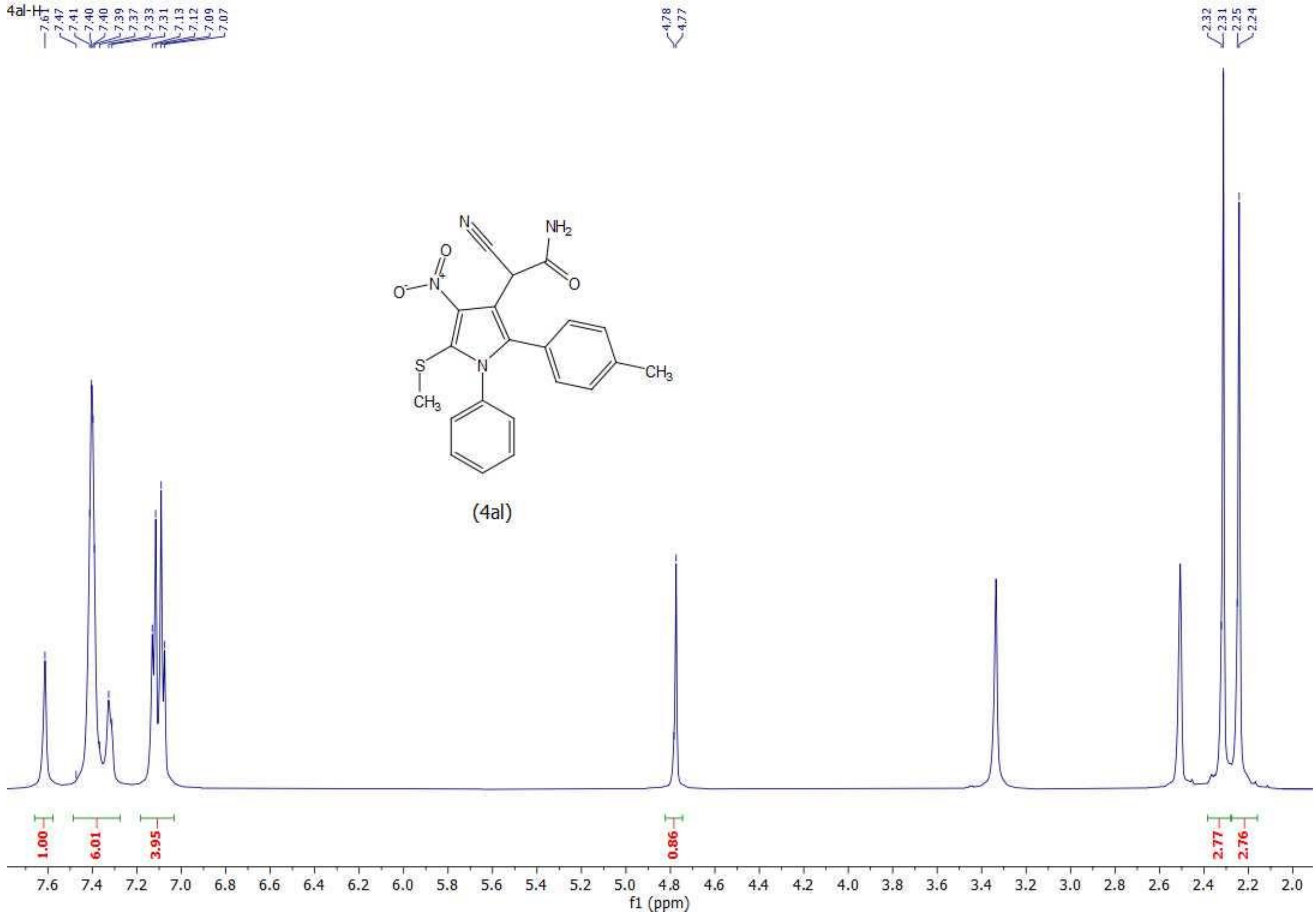
4al-H
7.61
7.47
7.41
7.40
7.40
7.39
7.37
7.33
7.31
7.13
7.12
7.09
7.07
4.78
4.77
2.32
2.31
2.25
2.24
NH2
N
O
O-
N+
S
CH3
CH3
1.00
6.01
3.95
0.86
2.77
2.76
7.6 7.4 7.2 7.0 6.8 6.6 6.4 6.2 6.0 5.8 5.6 5.4 5.2 5.0 4.8 4.6 4.4 4.2 4.0 3.8 3.6 3.4 3.2 3.0 2.8 2.6 2.4 2.2 2.0
f1 (ppm)

(4al)

4al-C

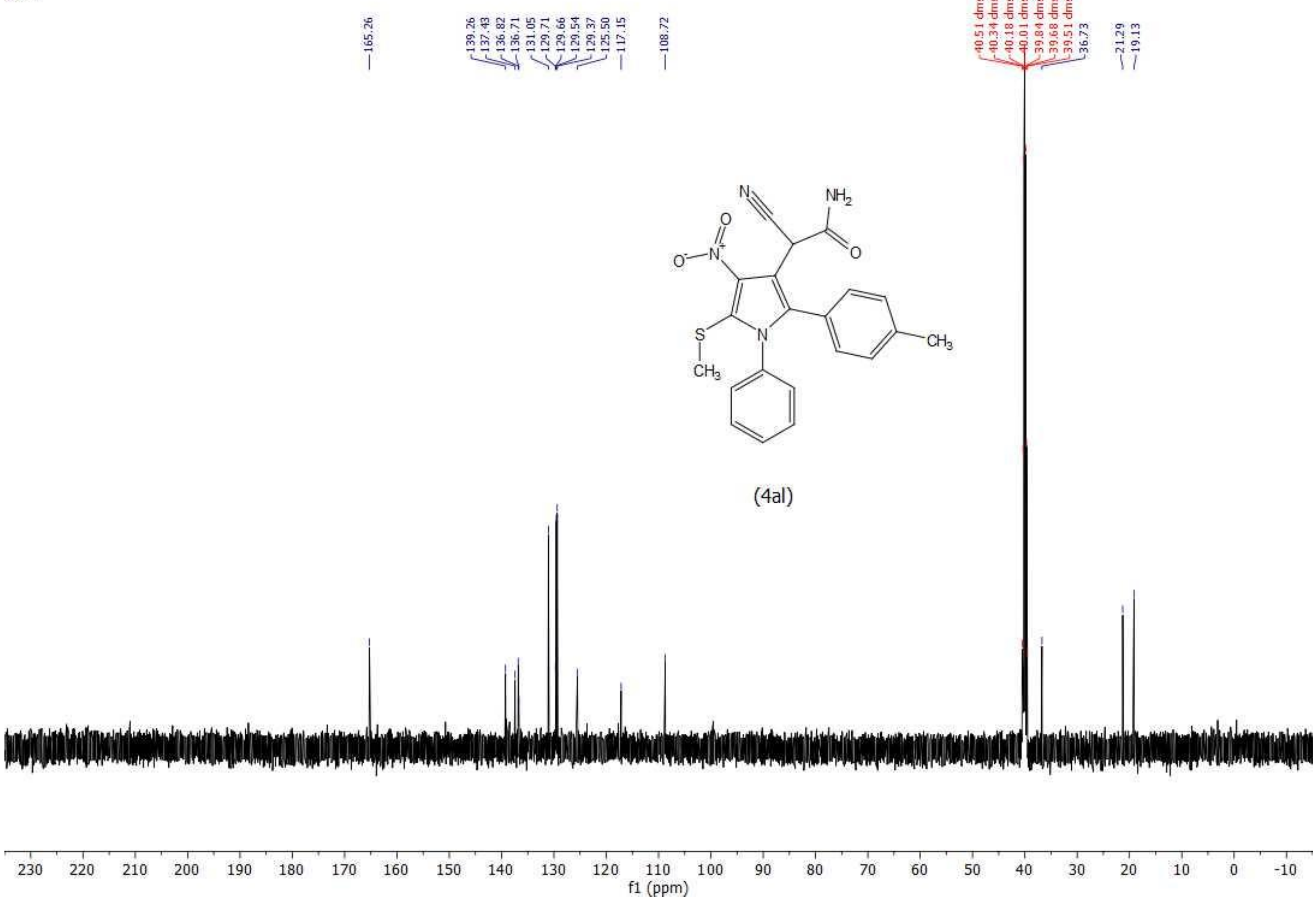
165.26
139.26
137.43
136.82
136.71
131.05
129.71
129.66
129.54
129.37
125.50
117.15
108.72
40.51 dmso
40.34 dmso
40.18 dmso
40.01 dmso
39.84 dmso
39.68 dmso
39.51 dmso
36.73
21.29
19.13
NH2
N
O
O-
N+
S
CH3
N
CH3
(4al)
230 220 210 200 190 180 170 160 150 140 130 120 110 100 90 80 70 60 50 40 30 20 10 0 -10
f1 (ppm)

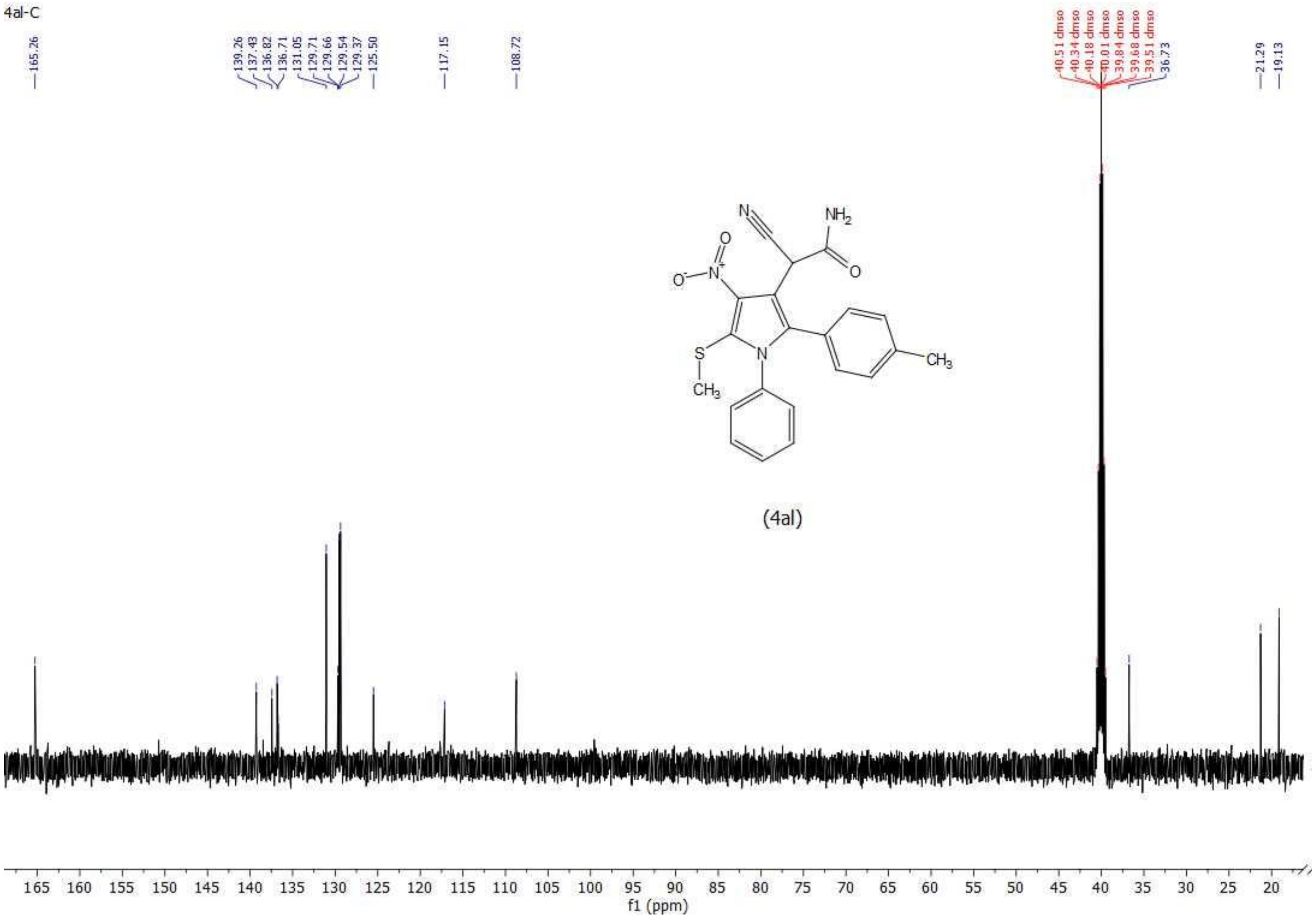
4al-C
165.26
139.26
137.43
136.82
136.71
131.05
129.71
129.66
129.54
129.37
125.50
117.15
108.72
40.51 dmso
40.34 dmso
40.18 dmso
40.01 dmso
39.84 dmso
39.68 dmso
39.51 dmso
36.73
21.29
19.13
NH2
O
N
O-
N+
S
CH3
CH3
(4al)
165 160 155 150 145 140 135 130 125 120 115 110 105 100 95 90 85 80 75 70 65 60 55 50 45 40 35 30 25 20
f1 (ppm)

4am-H

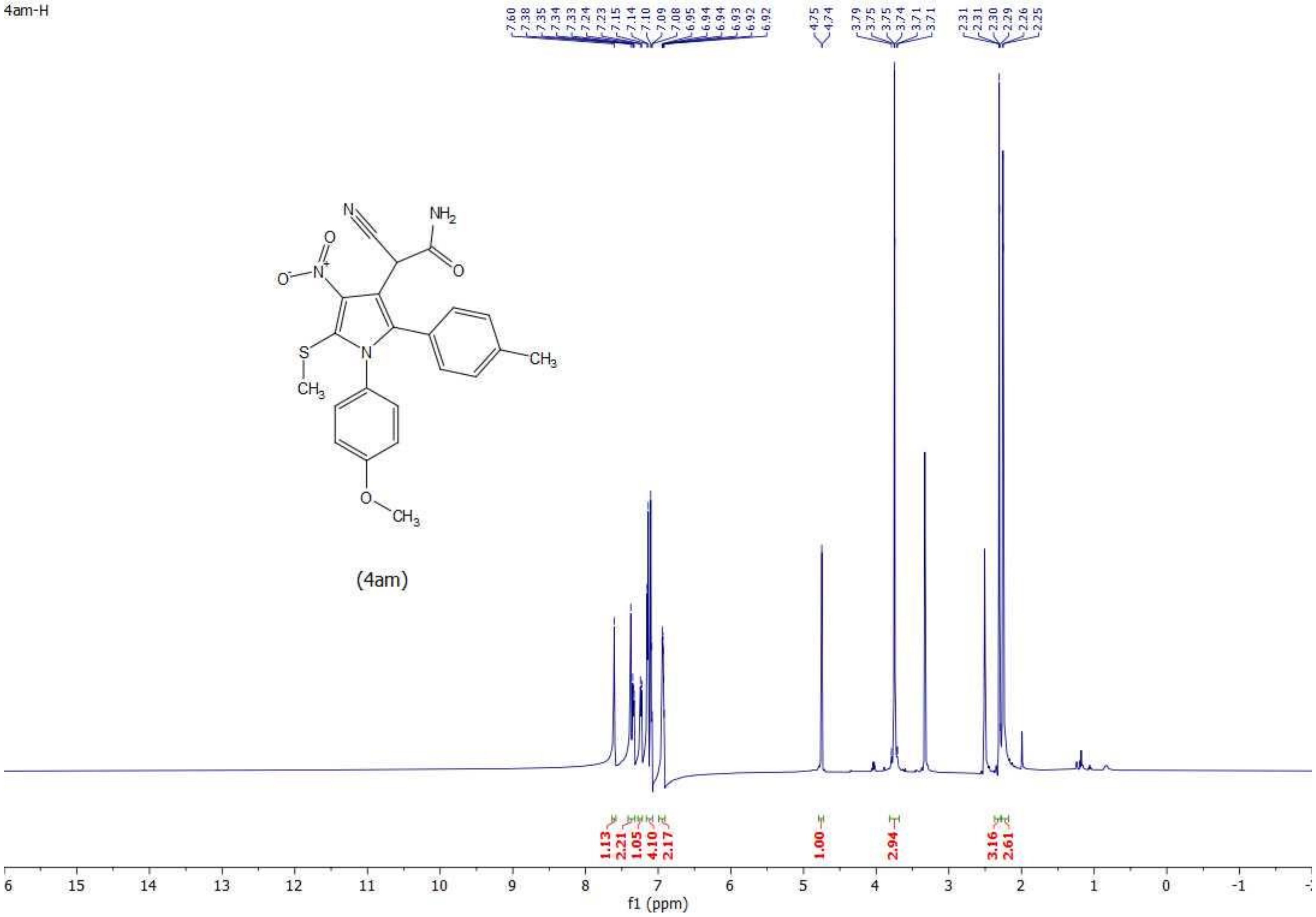
7.60
7.38
7.35
7.34
7.33
7.24
7.23
7.15
7.14
7.10
7.09
7.08
6.95
6.94
6.94
6.93
6.92
6.92
4.75
4.74
3.79
3.75
3.75
3.74
3.71
3.71
2.31
2.31
2.30
2.29
2.26
2.25
N
NH2
O
O-
N+
O
S
CH3
N
CH3
O
CH3
(4am)
1.13
2.21
1.05
4.10
2.17
1.00
2.94
3.16
2.61
6
15
14
13
12
11
10
9
8
7
6
5
4
3
2
1
0
-1
f1 (ppm)

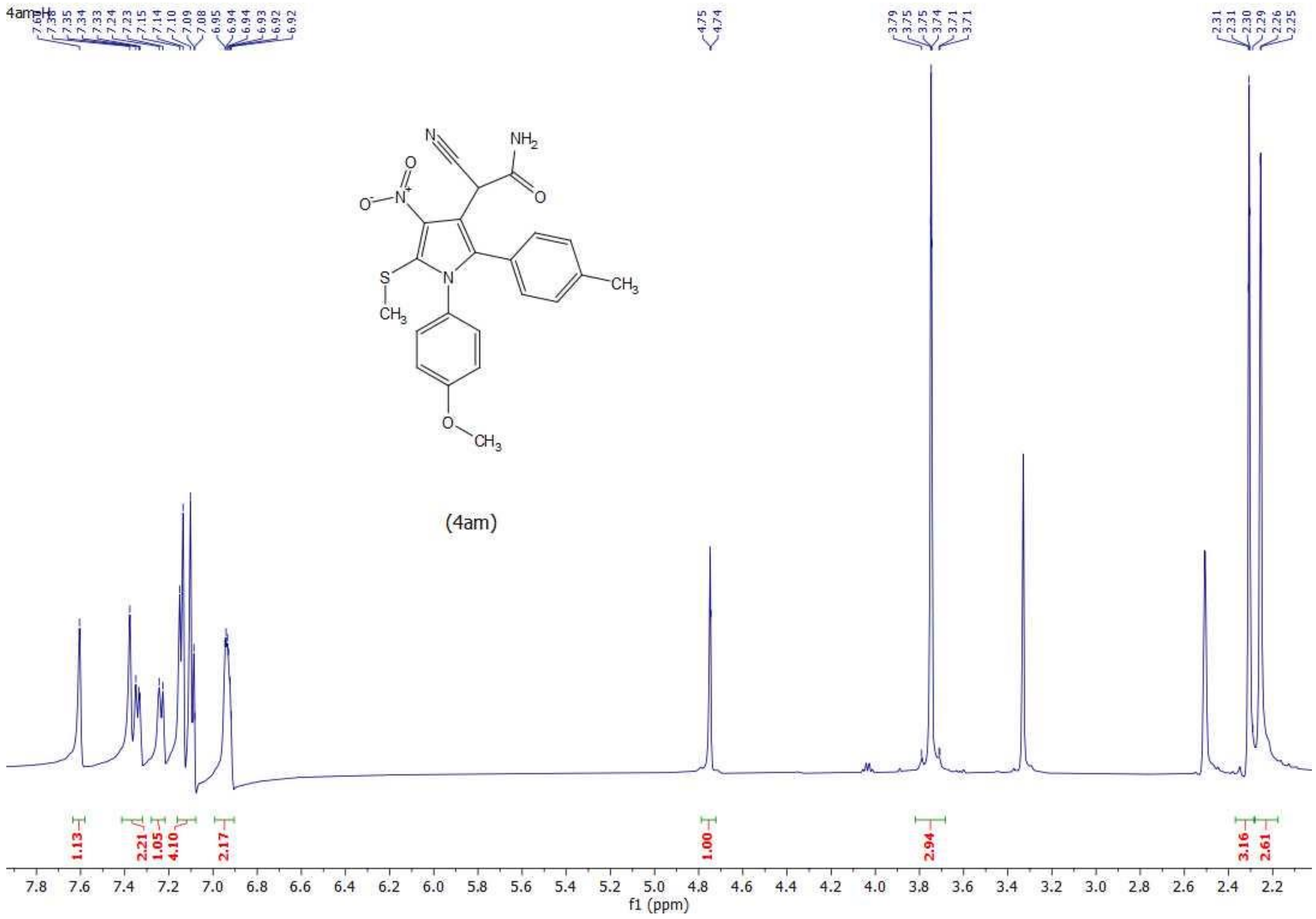
4am-H
7.61
7.38
7.35
7.34
7.33
7.24
7.23
7.15
7.14
7.10
7.09
7.08
6.95
6.94
6.94
6.93
6.92
6.92
4.75
4.74
3.79
3.75
3.75
3.74
3.71
3.71
2.31
2.31
2.30
2.29
2.26
2.25
(4am)
1.13
2.21
1.05
4.10
2.17
1.00
2.94
3.16
2.61
f1 (ppm)

4am-C

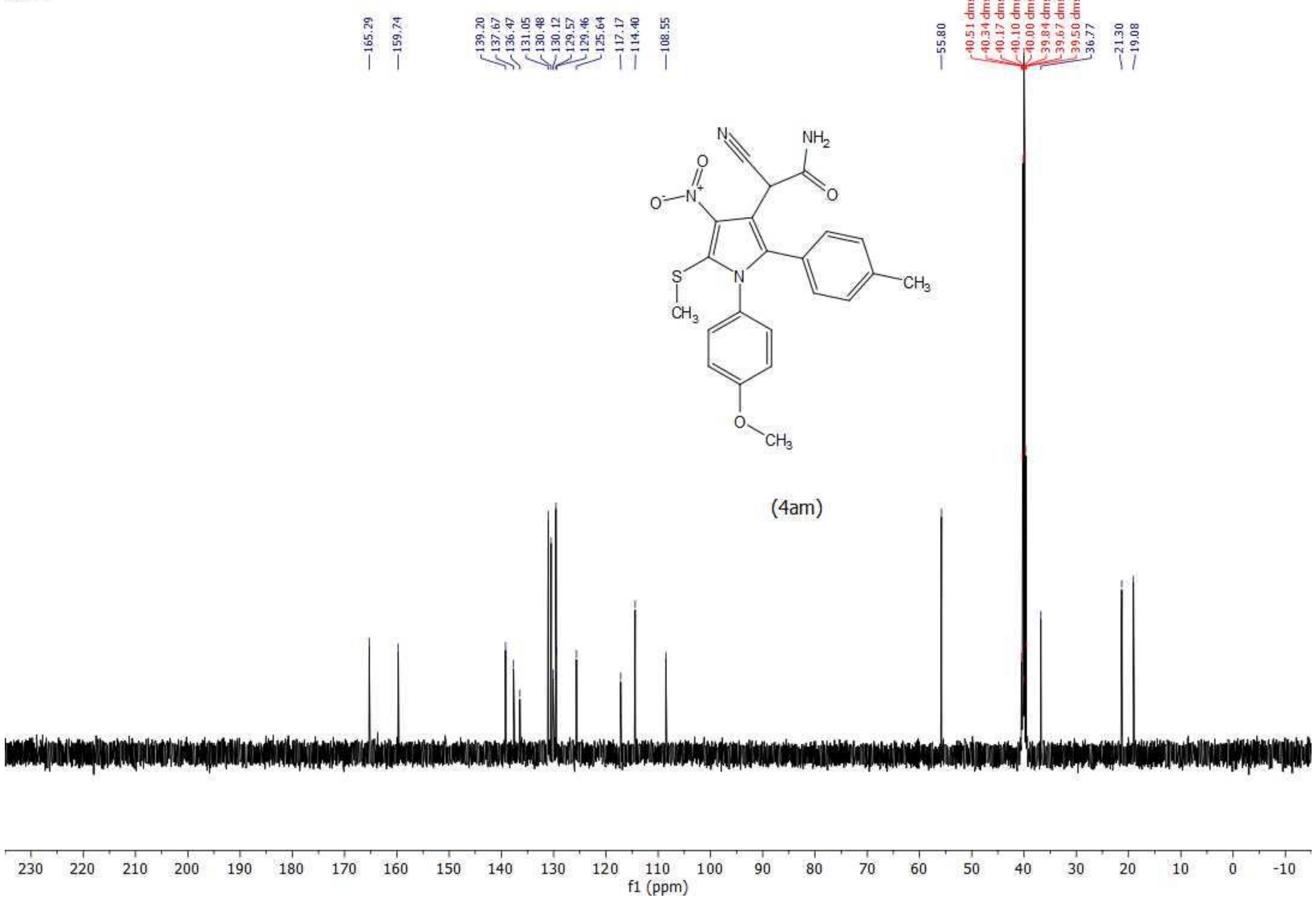
165.29
159.74
139.20
137.67
136.47
131.05
130.48
130.12
129.57
129.46
125.64
117.17
114.40
108.55
55.80
40.51 dmso
40.34 dmso
40.17 dmso
40.10 dmso
40.00 dmso
39.84 dmso
39.67 dmso
39.50 dmso
36.77
21.30
19.08
(4am)
230 220 210 200 190 180 170 160 150 140 130 120 110 100 90 80 70 60 50 40 30 20 10 0 -10
f1 (ppm)

4am-C

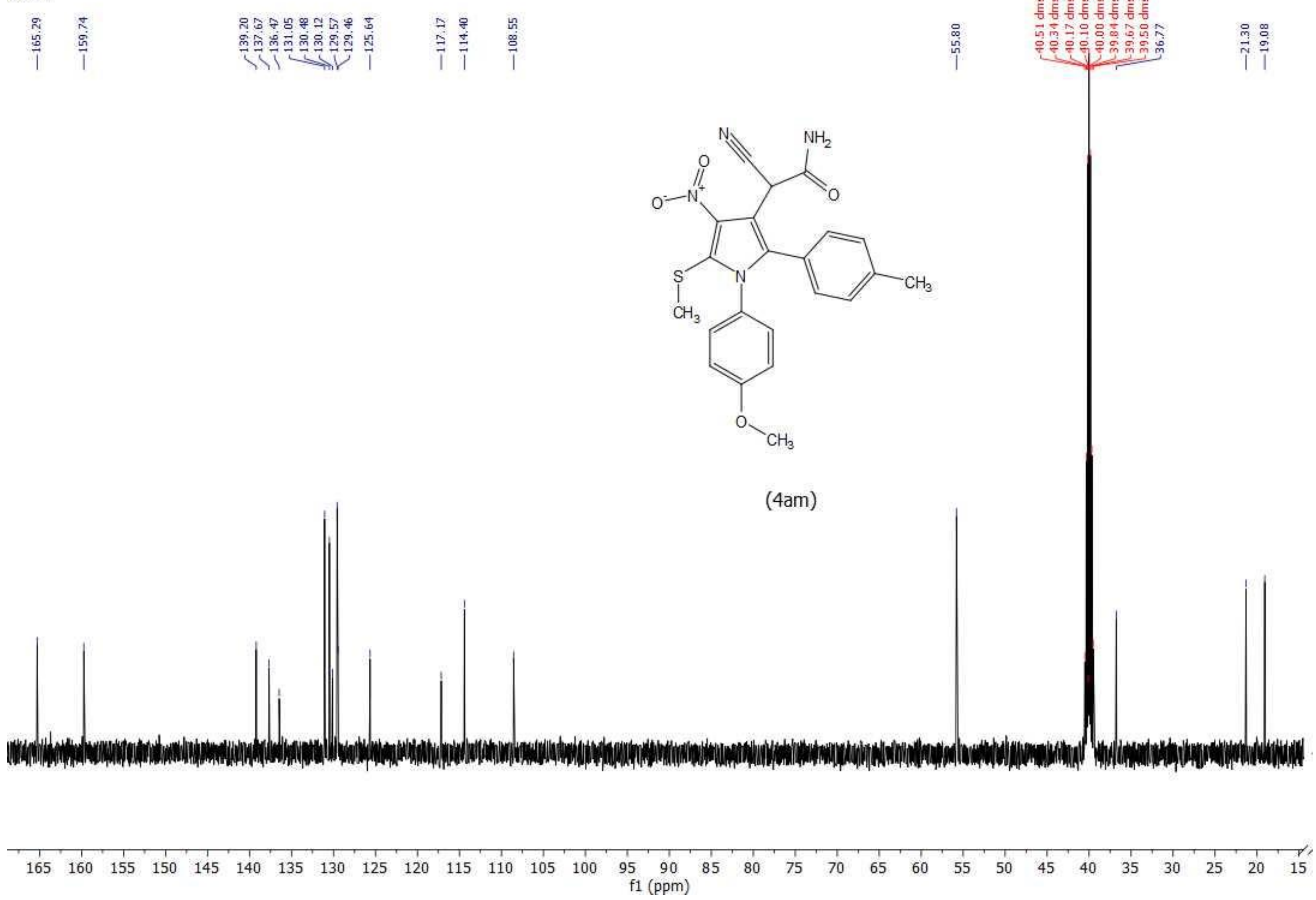
165.29
159.74
139.20
137.67
136.47
131.05
130.48
130.12
129.57
129.46
125.64
117.17
114.40
108.55
55.80
40.51 dmso
40.34 dmso
40.17 dmso
40.10 dmso
40.00 dmso
39.84 dmso
39.67 dmso
39.50 dmso
36.77
21.30
19.08
(4am)
f1 (ppm)
